# Global Analysis of Small Non-Coding RNA Populations across Tissues in the Malaria Vector, *Anopheles gambiae*

**DOI:** 10.3390/insects11070406

**Published:** 2020-06-30

**Authors:** William Bart Bryant, Savanna Ray, Mary Katherine Mills

**Affiliations:** Department of Biology and Geology, University of South Carolina-Aiken, Aiken, SC 29801, USA; skray@usca.edu

**Keywords:** small non-coding RNA, piRNA, small RNAs, mosquito reproduction

## Abstract

Malaria is a major global health problem, where the anautogenous female mosquito *Anopheles gambiae* serves as a major vector. In order to combat this devastating disease, understanding mosquito physiology is paramount. Numerous studies in the vector field demonstrate that small non-coding RNAs (ncRNAs) play essential roles in numerous aspects of mosquito physiology. While our previous miRNA annotation work demonstrated expression dynamics across differing tissues, miRNAs represented less than 20% of all small ncRNAs in our small RNA-Seq libraries. To this end, we systematically classified multiple small ncRNA groups across mosquito tissues. Here we (i) determined a new enriched-midgut miRNA, (ii) updated the piRNA annotation in ovaries with a genomic map of unique-mapping piRNAs, (iii) identified pan-tissue and tissue-enriched mRNA-derived small ncRNAs, and (iv) assessed AGO1- and AGO2- loading of candidate small ncRNAs. Continued research will broaden our view of small ncRNAs and greatly aide in our understanding on how these molecules contribute to mosquito physiology.

## 1. Introduction

Malaria continues to be a major significant health problem worldwide with an estimated 219 million cases and 435,000 deaths of malaria reported in 2017 alone [1]. Transmission of this devastating disease is tightly linked to the repeated blood feeding nature of the anautogenous female mosquito, *Anopheles gambiae.* Due to the hematophagous requirements of the female mosquito to produce eggs, an extensive understanding of her physiology is essential for the development of efficient malaria transmission blocking control strategies. For metazoans, one of the major factors for controlling physiology homeostasis are RNA molecules called small non-coding RNAs (ncRNAs) [2].

The multitude of small ncRNA groups include microRNAs (miRNAs), Piwi-interacting RNAs (piRNAs), small interfering RNAs (siRNAs), and mRNA-derived small ncRNAs, where each group plays a unique and significant role in numerous aspects of animal physiology [2,3,4,5,6,7]. While each small ncRNA group has a different biogenesis pathway, all small ncRNAs use sequence complementarity to regulate levels of their target RNA molecules [2]. For mosquito physiology, most small ncRNA research focuses on miRNAs, as loss-of-function studies demonstrate impressive reproduction and immunity phenotypes [8,9,10]. Recently, we updated and consolidated miRNA annotations and determined expression dynamics of the updated miRNAs across tissues in *An. gambiae* [11]. However, a substantial amount of small RNAs from our small RNA-Seq libraries remained unannotated. To this end, annotation and systematic classification of the diverse groups of small ncRNAs in the female mosquito is an essential step to take to decipher the physiological relevance and/or role of small ncRNAs in the transmission of vector-borne diseases.

The purpose of this study was to broaden our view of small ncRNAs in the female mosquito. Here, we systematically classified multiple small ncRNA groups across mosquito tissues resulting in (i) the discovery of an enriched-midgut miRNA, (ii) the updated piRNA annotation in ovaries with genomic map of unique mapping piRNAs, (iii) the identification of pan-tissue and tissue-enriched mRNA-derived small ncRNAs, and (iv) the assessment of AGO1 and AGO2 loading for candidate small ncRNAs of interest using publicly available sequence data. Overall, the work in this manuscript furthers our understanding and drives our appreciation of the diversity of small ncRNA groups across tissues in the malaria vector, *An. gambiae*.

## 2. Materials and Methods

### 2.1. Animals and Tissues

The *An. gambiae* G3 strain was reared and maintained in 28 °C humidified chamber with a 12-h light and dark cycle, as previously described [11]. To obtain mosquito tissues, adult female mosquitoes 3–5 days post eclosion were collected and dissected for (i) midgut, (ii) ovaries, (iii) fat body-enriched abdominal walls (fat body-Ab), and (iv) the remaining tissues comprised of the head and thorax. Dissected tissues were stored in DNA/RNA Shield (Zymo Research, Irvine, CA, USA) as previously described [11]. Each mosquito tissue group comes from three separate cages of mosquitoes, representing three biological replicates. 

### 2.2. Quantitative PCR

For validation purposes, the expression of miRNAs was quantified as previously described [11,12]. Briefly, RNA was extracted using a Direct-zol RNA Miniprep Kit (Zymo Research, Irvine, CA, USA). RNA was converted to cDNA using Qiagen miScript II RT Kit (Qiagen, Hilden, Germany). For miRNA expression, forward primers were the sequence of mature miRNA up to 58 °C Tm, and the reverse primer for all miRNAs was the universal primer 5′ GAATCGAGCACCAGTTACGC 3′, as described previously [11,12]. Quantification of miRNA by quantitative PCR (RT-qPCR) was performed using a Qiagen miScript SYBR Green PCR Kit (Qiagen) with the following PCR conditions: Step 1, 95 °C for 15 min; Step 2, 95 °C for 15 s, 56 °C for 30 s, and 70 °C for 30 s for 40 cycles; Step 3, 95 °C for 1 min; and then the melt curve analysis. Forward primers included: 

5’ CGTCAGATCTACTTCATACCCATGA 3′ for *miR-1174* and 5’ TTTCGAGACCACTGCAAACC 3′ for *miR-956*. *S7* primers were S7-Forward 5′ GTGCGCGAGTTGGAGAAGA 3’ and S7-Reverse 5′ ATCGGTTTGGGCAGAATGC 3′. 

The ribosomal *S7* gene served as a normalizer and the transcript expression was determined by 2^−∆*C*t^ [13].

### 2.3. Annotation of Small ncRNA Groups

FASTQ files from twelve small RNA libraries across mosquito tissues from our previous work [11] were probed to annotate various small ncRNA groups using the CLC Genomics Workbench version 12.0 (Qiagen, Hilden, Germany). The order for the annotation of small ncRNAs is illustrated in the flow chart in the Results section. 

Reads were trimmed of adapter sequences and mapped to the *An. gambiae* PEST genome (AgamP4.11) [14]. Reads were depleted of miRNAs, and for detailed analysis see [11]. To determine potential miRNA homologs, 20–25 nt size reads with >200 reads per unique sequence were compiled and manually queried against the miRBase database v22.1 (mirbase.org). With a >80 miRBase cutoff score, one miRNA homolog was elucidated. To quantitate abundance of the miRNA, chromosome 3L:39Mb loci was retrieved from Vectorbase (vectorbase.org) and used a as sequence reference for miRNA quantitation. 

Following the miRNA depletion of libraries, the remaining reads were queried against gene models for ncRNA groups (AgamP4.11) retrieved from BioMart (biomart.vectorbase.org). Reads mapping to rRNA and tRNA gene models were converted to reads per million (RPM). Mapped rRNA and tRNA reads over log_10_ = 1 were used to generate a heatmap using Morpheus from the Broad Institute (software.broadinstitute.org/morpheus/). Data were hierarchically clustered by Euclidean distance for metric, average for linkage method, and rows (rRNA or tRNA), and the columns (mosquito tissues) were clustered.

Following miRNA, tRNA, and rRNA depletion, the remaining reads were queried against transposable elements (long terminal repeats (LTR), non-LTR, and DNA transposons) retrieved from Repbase (girinst.org) and tefam (tefam.biochem.vt.edu, courtesy of Jake Tu at Virginia Tech University), allowing for 2 nt mismatches. Sequence strand orientation was determined using the CLC Genomics Workbench version 12.0. Sequence signature bias was determined using WebLogo 3 (weblogo.threeplusone.com).

Following miRNA, tRNA, rRNA, and TE-piRNA depletion, the remaining reads were queried against coding gene models (AgamP4.11) retrieved from BioMart (biomart.vectorbase.org). Candidate mRNA-derived small ncRNAs must (i) represent at least 10% of reads mapping to the coding gene model, and (ii) contain >1000 reads across the three biological replicates. Sequence strand orientation was determined using the CLC Genomics Workbench version 12.0.

### 2.4. Other Data Resource

To determine AGO1 and AGO2 loading of candidate small ncRNAs, publicly available small RNA datasets from the NCBI Sequence Read Archive (SRA), accession number SRP101587 [15], were retrieved. This dataset represents small ncRNAs associated with AGO1 and AGO2 through the immunoprecipitation of these proteins followed by RNA-Seq [15]. FASTQ files from AGO1- and AGO2-abdomens from non-blood fed female mosquitoes were trimmed of NEBNext Small RNA Library Prep Set adapter sequences and mapped to the *An. gambiae* PEST genome (AgamP4.11). Of note, as these libraries were made from abdomen tissue, they are comprised of fat body-Ab, midgut, and ovary tissues [15]. Trimmed and mapped reads were queried against (i) miRBase v22.1, (ii) the 39Mb loci on chromosome 3L for *miR-956* annotation, and (iii) coding genes (AgamP4.11), using parameters described above in previous sections. 

### 2.5. Data Availability

Sequencing data consisting of all small ncRNAs (15–50 nt) from all tissues have been submitted to NCBI SRA database (ncbi.nlm.nih.gov/sra) under BioProject number PRJNA630738. Sequencing data is also found at Vectorbase [14]. 

## 3. Results

### 3.1. Diversity of Small ncRNA Groups Across Mosquito Tissues

**Figure 1:**
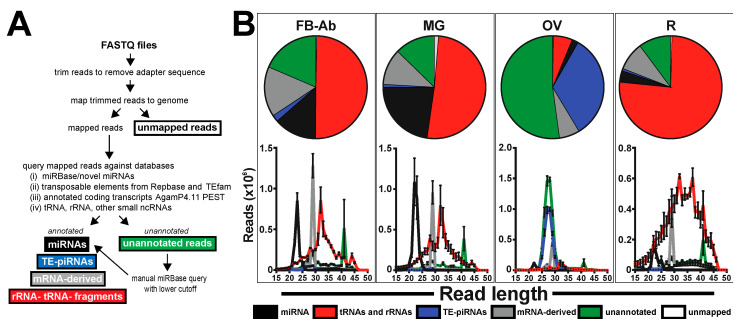
Diversity of small ncRNA groups across mosquito tissues. (**A**) Flow chart for the small ncRNA query against various databases. (**B**) Female mosquito tissues analyzed were (i) fat body-abdominal wall (FB-Ab), (ii) midgut (MG), (iii) ovary (OV), and (iv) the remaining tissues comprised of the head and thorax (R). Annotated small ncRNA groups include (i) miRNAs, (ii) tRNA/rRNA, (iii) TE-piRNAs, (iv) mRNA-derived small ncRNAs, (v) unannotated, and (vi) unmapped. For each mosquito tissue group, data demonstrate the read annotation percentages and the corresponding histograms of the nt size representing the various small ncRNA groups. All values graphed as average +/− SEM from three biological replicates.

As a means to complement our previous miRNA annotation work [11], unannotated reads were queried against a myriad of databases to determine the vast differences among small ncRNA groups across mosquito tissues in the malaria vector. The small RNA libraries represented four tissue groups, each with three biological replicates: (i) abdominal walls for fat body-Ab (FB-Ab), (ii) midguts (MG), (iii) ovaries (OV), and (iv) remaining tissues comprised of the head and thorax tissue (R). In total, the twelve small RNA libraries contained 141.3 million reads. Annotated reads were partitioned into five small ncRNA groups: (i) miRNAs, which includes an update to our previous work [11]; (ii) tRNA/rRNAs; (iii) TE-piRNAs; (iv) mRNA-derived small ncRNAs; and (v) unannotated reads (Figure 1A). Unmapped reads were removed from further analysis, but are available upon request.

In total, across all mosquito tissues, 14.5 million reads accounted for miRNAs, 64.7 million reads accounted for tRNA/rRNA, 13.2 million reads accounted for TE-piRNAs, 14.8 million reads accounted for mRNA-derived small RNAs, 33.2 million accounted for unannotated, and 0.8 million reads accounted for unmapped (Table S1). The abundance for each small ncRNA group varied across mosquito tissues (Table 1). Midgut tissue had the highest miRNA abundance with ~22.62%, while ovary tissue had the least miRNA abundance with ~2.07%. The remainder tissue had the highest tRNA/rRNA abundance with ~77.24%, while ovary tissue had the least miRNA abundance with ~6.24%. Ovary tissue had the highest TE-piRNA abundance with ~34.72%, while the remainder tissue had the least TE-piRNA abundance with ~0.76%. Fat body-Ab tissue had the highest mRNA-derived small ncRNA abundance with ~15.56%, while ovary tissue had the least mRNA-derived small ncRNA abundance with ~6.47%. Ovary tissue had the highest unannotated read abundance with ~54.33%, while the remainder tissue had the least unannotated read abundance with ~10.38%, see Table 1 and Table S1. 

The nucleotide size profile of each small ncRNA group across tissues was distinct. Indeed, (i) miRNAs were 20–25 nts, (ii) tRNA/rRNAs were spread across 15–50 nts with a prominent peak at 32 nts, (iii) TE-piRNAs were 25–30 nts, and (iv) mRNA-derived small ncRNAs possessed a prominent peak at 29 nts (Figure 1B). For unannotated reads, there was a prominent peak at 41 nts across all tissues. Additionally, most unannotated reads in the ovary tissue were within 25–30 nts (Figure 1B). 

### 3.2. miRNA Annotation Update

To update miRNA annotations, mis-called miRNAs from our previous work (Dataset S1 from [11]) were queried against transposable elements from Repbase and TEfam. We found 10% to be redundant in naming (Table S2) and 63% mapped to transposable elements, suggesting these mis-called miRNAs are actually piRNA processing byproducts (Table S2), agreeing with our previous speculation [11]. 

**Figure 2:**
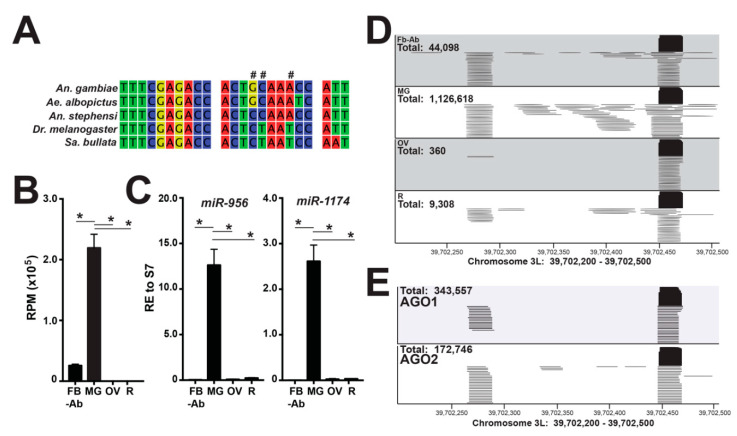
*miR-956* expression dynamics across mosquito tissues. (**A**) *miR-956* sequence alignment across dipterans; ‘#’ indicates a nucleotide mismatch between the *A. gambiae* and *D. melanogaster miR-956* sequences. (**B**) Expression of *miR-956* is significantly higher in the midgut (MG) over the fat body abdominal wall (FB-Ab), ovary (OV), and remaining head and thorax tissues (R). Reads per million (RPM) values are graphed as the average +/− SEM from three biological replicates; ‘*’ indicates statistical significance by one-way analysis of variance (ANOVA) followed by Tukey’s multiple comparisons test, *p* < 0.0001. (**C**) The validation of the *miR-956* enrichment in midgut tissues by RT-qPCR, values graphed as the average +/− SEM from three biological replicates; ‘*’ indicates statistical significance by one-way ANOVA followed by Tukey’s multiple comparisons test, *p* < 0.0001. *miR-1174* served as the positive control for midgut miRNA enrichment. (**D**) Mapping of *miR-956* reads to 39.7 Mb loci on chromosome 3L across mosquito tissues. (**E**) AGO1 and AGO2 loaded *miR-956* reads mapping to 39.7 Mb loci on chromosome 3L, using reanalyzed sequencing data obtained from [15].

Additionally, 20–25 nts unannotated sequences with >200 reads were compiled from all tissue libraries and manually queried against miRBase v22.1 (miRBase.org). Here, a substantial number of reads aligned to *miR-956* from *Drosophila melanogaster*, with a 3 nt mismatch (Figure 2A), which was one nucleotide mismatch over our cutoff in our previous work [11]. Additionally, *miR-956* has been reported in *Aedes albopictus*, *Anopheles stephensi*, and *Sarcophaga bullata* [16,17,18], but these dipterans are not represented in miRBase v22.1 (Figure 2A). Reads aligning to *miR-956* mapped to the 39.7 Mb loci on chromosome 3L with ~150 nt separating the 5p-miRNA and 3p-miRNA strands (Figure 2D). Quantitation of *miR-956* was determined by using the chromosome 3L 39.7 Mb loci as a reference sequence (Figure 2B,D and Dataset S2). To this end, *miR-956* was the second highest expressed miRNA in the midgut small RNA-Seq libraries and was statistically significantly enriched in midgut tissue (one-way analysis of variance (ANOVA) followed by Tukey’s multiple comparisons test, *p* < 0.0001) (Figure 2B). RNA-Seq data were verified by RT-qPCR, where midgut-enriched *miR-1174* served as our positive control (Figure 2C). To amend to our annotation of *miR-956*, we determined AGO1 and AGO2 loading of this miRNA by interrogating publicly available sequence data [15], as miRNAs loaded into Argonaute proteins demonstrate functionality. To this end, our analysis found *miR-956* to be the second highest AGO1-loaded miRNA (16% of total annotated miRNAs) where 343,557 reads mapped to the 39.7 Mb loci on chromosome 3L (Figure 2E). AGO2 small RNA libraries also contained *miR-956*, but at lower levels (Figure 2E). 

### 3.3. tRNA- and rRNA-Fragment Annotation

Most small RNA transcriptome studies suggest reads mapping to tRNA and rRNA genes are simply metabolism byproducts, but recent studies suggest physiological roles for tRNA- and rRNA- derived small RNAs [19,20,21], including differential expression in *Aedes aegypti* [22]. To this end, we queried our mosquito tissue libraries reads to tRNAs, rRNAs, and other annotated ncRNA genes retrieved from Vectorbase. From these non-coding gene models, reads mostly mapped to tRNA and rRNA genes across all tissues with a range of 7–40% and 69–92% for tRNAs and rRNAs, respectively (Figure S1). Our data, along with a previous report [23], demonstrated that the mosquito small RNA transcriptome consists of a copious amount tRNA- and rRNA- small RNAs in all tissues, with the exception of the ovary tissue small RNA transcriptome. The 32 nt size peak in the tRNA/rRNA group (Figure 1B) was represented by tRNA mapped reads (Figure S1), while rRNA reads did not yield any discernable patterns in nucleotide size. Unfortunately, differential abundance analysis of tRNA- and rRNA- reads across tissues did not exhibit any obvious distinct tissue expression patterns (Figure S2 and Dataset S1). 

### 3.4. TE-piRNA Annotation

**Figure 3:**
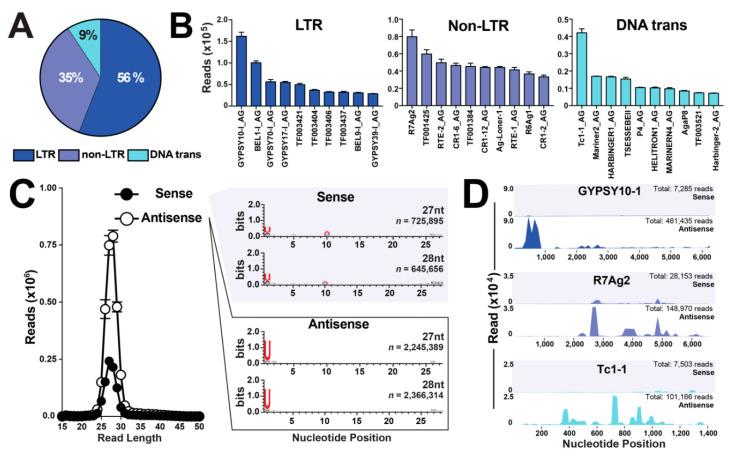
Sequence characteristics of TE-derived piRNAs in a mosquito ovary. (**A**) Percentage of piRNAs mapped to three classes of transposable elements: LTR, non-LTR, and DNA transposon. (**B**) Top ten piRNA mapping transposable elements for each class of transposable elements. Values graphed as the average +/− SEM from three biological replicates. (**C**) Histogram of nucleotide size distribution of sense and antisense piRNA sequences. Values graphed as the average +/− SEM from three biological replicates. WebLogo3 sequence bias analysis for 27- and 28-nt size sense and antisense piRNA sequences compiled from three biological replicates. (**D**) Mapping properties of sense and antisense piRNA reads compiled from three biological replicates mapped to the top LTR, non-LTR, and DNA transposon.

Previous studies in the malaria mosquito interrogated small RNA libraries against TEs from Anopheles (378) using Repbase [24,25,26]. To compliment these studies, the number of interrogated TEs were doubled by the inclusion of 392 Anopheles TEs retrieved from TEfam, totaling 770 annotated transposable elements (Dataset S3). Nearly all annotated transposons were represented, with a range of 99.3–100% and 90.1–99.5% for Repbase and TEfam, respectively (Dataset S4). Class I LTRs represented the highest piRNA annotation at 56%, followed by 35% and 9% for non-LTR and DNA transposons, respectively (Figure 3A and Dataset S4), agreeing with previous reports [24,25,26]. Also, across all classes of TEs, the top ten piRNA mapping transposable elements contained sequences from both Repbase and TEfam databases (Figure 3B).

While piRNA sequences are not usually conserved across species (reviewed in [27]), piRNAs do possess sequence bias characteristics, where a majority of piRNAs are antisense to their TE target sequence [7,28]. Indeed, 77% of piRNA sequences were determined to be antisense and 23% to be sense (Figure 3C), in agreement with reports in flies [28] and mosquitoes [24,25,26]. Additional shared piRNA sequence characteristics include a strong bias for uracil (U) at position-1 for both sense and antisense TE-piRNA sequences, and a strong bias for adenosine (A) at position-10 for sense TE-piRNA sequences, as reviewed in [7]. From three ovary biological replicate libraries, sense TE-piRNA reads of 725,895 and 645,656 for 27 nt and 28 nt, respectively, possessed a strong bias for U at position-1 and A at position-10 (Figure 3C). Additionally, antisense TE-piRNA reads of 2,245,389 and 2,366,314 for 27 nt and 28 nt, respectively, possessed strong bias for U at position-1 (Figure 3C), agreeing with reports in flies [28] and mosquitoes [24,25,26]. Lastly, the top TE-piRNA mapped LTR, non-LTR, and DNA transposons were analyzed for mapping properties along their TE-target sequence. Across all TE representatives, a vast majority of piRNA sequences were antisense with no obvious mapping preferences along the TE-target sequences (Figure 3D), agreeing with [29,30]. 

**Figure 4:**
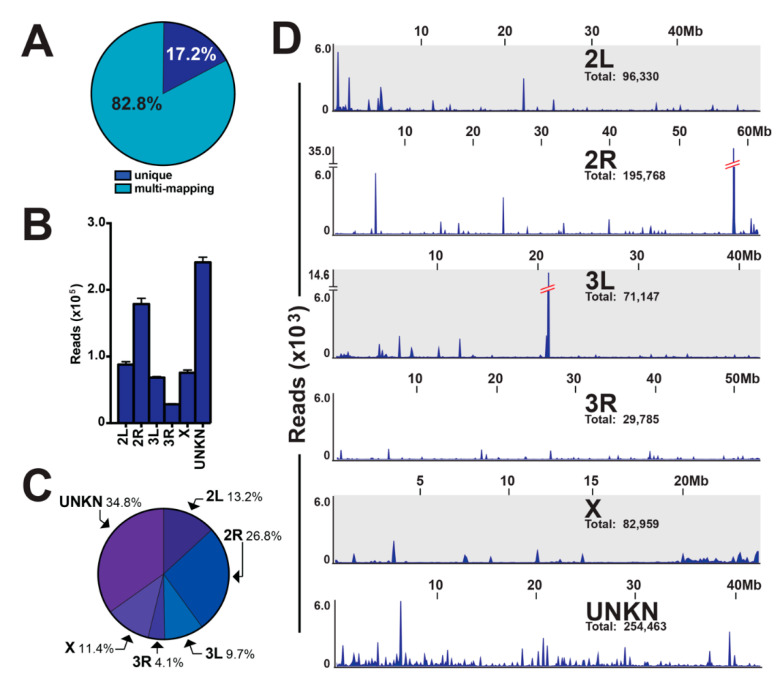
Genome-wide mapping properties of piRNAs. (**A**) Percentage of piRNA sequences with unique or multi-mapping properties. (**B**) Read intensity and (**C**) chromosomal contribution across chromosomes for unique mapping piRNAs, with the values graphed as average +/− SEM from three biological replicates. (**D**) Genomic loci map of unique mapping piRNAs across chromosomes, with notable 58 Mb loci on chromosome 2R and 21 Mb loci on chromosome 3L accounting for approximately 20% of unique mapping piRNAs.

A majority of piRNA sequences map to multiple chromosomal locations in a variety of organism genomes [4,5,6,7,31]. To this end, 82.8% of piRNAs from our mosquito ovary small RNA libraries were found to be multi-mapping, and 17.2% of piRNA were determined to be unique mapping (Figure 4A). For the unique mapping piRNAs, we determined: (i) read intensity across chromosomes with a range of 28,262–241,335 reads for chromosome 3R and chromosome UNKN, respectively (Figure 4B); (ii) chromosomal contribution with a range of 4.1–34.8% for chromosome 3R and chromosome UNKN, respectively (Figure 4C); and (iii) a genomic loci map of unique mapping piRNAs (Figure 4D). The number of unique mapping piRNAs varied across the mosquito genome with two notable piRNA clusters that accounted for ~20% of all unique mapping piRNAs. Indeed, the 58 Mb loci on chromosome 2R and the 21 Mb loci on chromosome 3L accounted for 17.7% and 3.3% of unique mapping piRNAs, respectively (Figure 4D).

### 3.5. mRNA-Derived Small RNA Annotation

Multiple small RNA studies across disciplines, including mosquitoes, report a substantial amount of small ncRNAs derived from coding genes [32,33,34,35,36,37,38,39], suggesting these mRNA-derived small ncRNAs are not mere RNA metabolism byproducts but rather hold physiological significance. Thus, to determine mRNA-derived small ncRNAs for the mosquito, we interrogated our small RNA libraries against Vectorbase gene models, AgamP4.11. Initial analysis identified many mRNA candidates. However, some possessed a low number of small RNA reads mapping to the associated mRNA candidate (<1000 reads), suggestive of mere RNA metabolism byproducts, which should be excluded. Thus, to obtain a list of *bona fide* mRNA-derived small ncRNAs with physiological relevance, we focused on candidate small ncRNAs with (i) one unique sequence accounting for 10% of total mapped reads to a candidate gene, and (ii) one that contains >1000 reads across the three tissue biological replicates.

**Figure 5:**
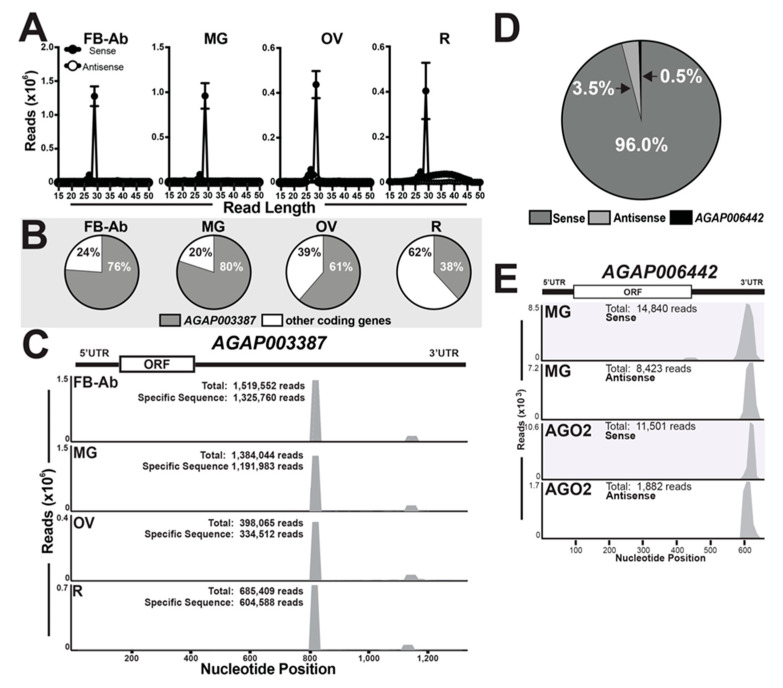
mRNA-derived small RNAs across tissues. (**A**) Nucleotide size distribution histograms of sense and antisense mRNA-derived small ncRNAs across mosquito tissue groups, fat body abdominal wall (FB-Ab), midgut (MG), ovary (OV), and remaining head and thorax tissues (R). Values graphed as the average +/− SEM from three biological replicates. (**B**) Percentage of mRNA-derived small ncRNAs mapping to *AGAP003387* and other coding genes across tissues. (**C**) *AGAP003387*-derived small ncRNAs mapping to 3′UTR across mosquito tissues. ‘Total’ refers to the number of reads mapped to the transcript, and ‘specific sequence’ refers to the number of reads mapped to a specific 29-nt sequence. A representative for *AGAP003387*-derived small ncRNAs is shown, and other biological replicates are found in (Figure S4). (**D**) The percentage of mRNA-derived small ncRNAs according to the target orientation: sense, antisense, or sense/antisense (*AGAP006442)*. (**E**) Mapping properties of *AGAP006442*-derived small ncRNAs from (i) midgut (MG) and (ii) AGO2 small RNA libraries. A representative is shown for midgut tissues, and other biological replicates are found in (Figure S3). Reanalyzed AGO2 loaded small RNA sequencing data obtained from [15].

From our mRNA-derived small ncRNA list (Dataset S5), reads ranged in nucleotide size from 15 nts (from *AGAP002104*) to 37 nts (from *AGAP001546*) (Dataset S5). Reads mapped to mRNA transcripts in (i) sense, (ii) antisense, or (iii) sense and antisense orientations (Dataset S5). At ~96%, a majority of mRNA-derived small ncRNAs were mapped in sense orientation across all tissues (Figure 5A,D, and Dataset S5). Further, 60% (8,827,997 reads in total across all mosquito tissue small RNA libraries) were mapped to 3′UTRs of their target mRNA (Dataset S5). Of note, *AGAP006442* was the only candidate gene with both sense and antisense orientation reads mapping to the 3′UTR (Figure 5D) and was only present in midgut libraries (Figure 5E and Figure S3, and Dataset S5). In agreement with previous reports on *An. gambiae* [24,25,26], the gene *AGAP003387* accounted for the highest amount of mRNA-derived small ncRNAs (Dataset S5) with a range of 38–80% for remainder tissue and midgut tissue, respectively (Figure 5B). The most impressive data point is the 3′UTR mapping 29 nt sequence, 5′ UUCGGAUGUAACAUCUAGUAUAAAACCU 3′, which on average of all the total reads from all small RNA libraries accounted for (i) ~10.52% (~1,228,435 reads) in the fat body-Ab, (ii) ~7.93% (~924,497 reads) in the midgut, (iii) ~3.45% (~410,795 reads) in the ovary, and (iv) ~3.12% (~369,519 reads) in the remainder tissue, (Figure 5C, Dataset S5, and Table S1). Reads mapping both sense and anti-sense to *AGAP006442* resembled siRNAs, which is in agreement with [25]. Thus, to gain evidence for physiological relevance, we questioned if mRNA-derived small ncRNAs to *AGAP006442* were loaded into AGO2 by interrogating publicly available sequence data [15], as loading into AGO proteins is highly suggestive of functionality. Here, we found *AGAP006442* to have the highest number of mapped reads (9.4% of mRNA-derived ncRNAs) with 11,501 and 1882 reads mapping in sense and anti-sense orientation, respectively (Figure 5E). Additionally, *AGAP006442*-derived small ncRNAs from (i) AGO2- and (ii) our midgut- small RNA libraries exhibited similar 3′UTR mapping properties (Figure 5E). 

## 4. Discussion

To broaden our overall understanding of the abundance and diversity of small ncRNA groups in the malaria vector, *An. gambiae*, we systematically classified these small ncRNAs across tissues resulting in (i) finding an evolutionarily conserved and midgut-enriched miRNA, (ii) updating the piRNA annotation in ovaries paired with a genomic map of unique mapping piRNAs, (iii) the identification of pan-tissue and tissue-enriched mRNA-derived small ncRNAs, and (iv) a demonstration of physiological relevance for candidate small ncRNAs by the assessment of AGO1 and AGO2 loading using publicly available RNA Seq datasets [15]. While no tissue enrichment patterns for tRNA- and rRNA- fragments were determined, this small ncRNA group should not be overlooked as they are (i) abundant across all domains of life [19,20]; (ii) loaded into plant, Drosophila, and mosquito AGO complexes [15,19,20]; (iii) regulators of translation during stress in halophilic archaea [21]; and (iv) differentially expressed across life stages in the mosquito, *Aedes aegypti* [22]. 

Manual interrogation of our mosquito tissue small RNA libraries identified the midgut enriched miRNA, *miR-956*. As there are 3 nt mismatches between *An. gambiae* and *D. melanogaster miR-956* sequences, it missed our previous miRNA annotation work, which used a routine 2 nt mismatch cutoff [11]. Of note, the >150 nt hairpin for *miR-956* is larger than most miRNA hairpins. However, it is worth noting that *miR-1174* in mosquito and *miR-989* in mosquito and fruit flies also share similar miRNA hairpin sizes and are both tissue-enriched [11,24,40,41,42,43,44]. Additionally, *miR-956* has been reported in small ncRNA studies in *Aedes albopictus*, *Anopheles stephensi*, *Sarcophaga bullata*, and *Lucilia sericata* [16,17,18], but unfortunately these species are not represented in miRBase. To add physiological relevance for *miR-956*, we used publicly available RNA-Seq data from [15], and found this miRNA to be the second highest loaded miRNA in AGO-1 in sugar fed female mosquito abdomen tissue, which are comprised of the midgut, ovary, and fat body tissues [15]. In Drosophila, *miR-956* is proposed to control circadian rhythms [45] and suppress virion levels of Drosophila C virus by targeting an evolutionarily conserved gene, called *Ectoderm-expressed 4*, proposed to modulate Toll signaling [46]. As *miR-956* is midgut-enriched, loaded into AGO-1 complexes, and evolutionarily conserved, more work is warranted to determine role(s) for this specific miRNA in reproduction and immunity in Anophelinae and Culicinae mosquitoes. 

Most of the ovary small ncRNA transcriptome were represented by piRNAs, which agrees with [24,25,26], as this small ncRNA group safeguards the host genome from mobile parasitic DNA elements, called transposable elements (TEs). piRNAs (i) range in size from ~23–36 nts, (ii) are directly complementary to target TE sequences, (iii) are mostly antisense and minorly sense to TE sequences, and (iv) reside as clusters in the host genome [24,25,26,31]. Our work added to the current piRNA annotations for *An. gambiae* [24,25,26] by adding the TEfam database (courtesy of Jake Tu at Virginia Tech) to annotate piRNAs mapping to LTR, non-LTR, and DNA transposons. All three groups were accounted for in our analyses, and piRNA sense- and antisense-signature sequences agreed with literature across metazoans [4,5,6,7,24,25,26]. Further, each species contains hundreds of unique TEs in their genome that require repression; thus, each species has a unique piRNA transcriptome. While we added to the piRNA transcriptome in *An. gambiae*, future work is needed. Indeed, a high number of ovary reads were not annotated, but were ~23–36 nts, and sequence probing found the classic U at position-1 signature sequence. Thus, these unannotated ovary reads hold promise for annotating additional TEs within the *An. gambiae* genome. Additionally, these unannotated ovary reads could lead to the discovery of endogenous viral elements for the malaria vector. Lastly, the payoff for studying piRNAs is their use as a mosquito population management strategy. In fruit fly populations, hybrid dysgenesis occurs where a male fruit fly carrying a novel TE with associated piRNAs is mated with a naïve female lacking the TE and associated piRNAs. When these flies mate, their progeny have un-repressed TEs. These un-repressed TEs then damage the fly genome with the end result of insect sterility defects, as reviewed in [5,7]. In 1988, vector biologists noted hybrid dysgenesis as a novel mosquito population management strategy [47]. However, basic research on how mosquito piRNAs protect the genome is a necessity before implementing this biology into a vector control strategy.

Surprisingly, across all tissues, we found a substantial amount of reads from our small ncRNA libraries mapping to coding genes, mostly to the 3′UTR. While similar in size to piRNAs, these small RNAs are unique as they mostly map sense to their mRNA target [32,33,34,35,36,37,38,39]. For simplicity, we call these reads mRNA-derived smalls ncRNAs, previously termed genic-piRNAs or exonic-piRNAs [32,33,34,35,36,37]. Even though mRNA-derived small ncRNAs are noted in numerous animal small RNA transcriptomes [24,25,26,32,33,34,35,36,37,38,39], they are the most understudied small ncRNA group. This small ncRNA group regulates translation through the regulation of ribosomes in yeast [48], and have roles in mRNA decay in ovary development in fruit flies [33]. Further, in Drosophila, numerous studies report genetic interactions between mRNA-derived small ncRNAs and piRNAs [34,35,36,37]. In *Aedes aegypti*, histone mRNA-derived small ncRNAs regulate the cell cycle [38]. Our study found multiple examples of pan-tissue or tissue-enriched mRNA-derived small RNAs. *AGAP003387*-derived small ncRNAs accounted for a substantial amount of reads across all tissues. For comparison, in fat body-Ab tissue, the total annotated miRNAs accounted for ~13%, and the one 29 nt *AGAP003387*-derived small ncRNA accounted for ~10% of all reads in the small RNA-Seq library. Further, in ovary tissue, the total annotated miRNAs accounted for ~2%, while the same 29 nt *AGAP003387*-derived small RNA accounted for ~3.5%. Our data agree with three independent research groups reporting a substantial amount of reads map to *AGAP003387* [24,25,26]. Our study confirms these works and adds perspective on small ncRNA abundance since we compared all small ncRNA groups across tissues. As mRNA-derived small RNAs are preferentially loaded into AGO3 [35,36], this sequence was not found to be loaded into AGO-1 and AGO-2 after querying the publicly available RNA-Seq data [15]. In contrast, a substantial amount of small RNAs mapping to *AGAP006442* were found to be loaded into AGO-2. Additionally, *AGAP006442*-derived small ncRNAs were both sense and antisense, and were only found in our midgut small RNA-Seq libraries, suggestive of physiological relevance for midgut tissue in the female mosquito. Overall, due to the vast abundance and physiological relevance for mRNA-derived small ncRNAs across metazoans [24,25,26,32,33,34,35,36,37,38,39], these understudied small ncRNAs hold potentially new biology that is relevant to vector biologists. 

## 5. Conclusions

Given the continued burden of malaria on the global population, there will always be a benefit from basic mosquito physiology research. Numerous studies clearly demonstrate the importance of the small ncRNA group, miRNAs, in terms of reproduction and immunity of the female mosquito [8,9,10]. Here, we expand the view of small ncRNAs through systematically classifying small ncRNA populations across female mosquito tissues, which will pay dividends for future mosquito physiology research. 

## Figures and Tables

**Table 1:** Average percentage of mapped reads to small ncRNA groups across mosquito tissues.

Tissue	miRNA	tRNA/rRNA	TE-piRNAs	mRNA-Derived	Unannotated	Unmapped
Fat Body-Ab	13.43	49.89	2.21	15.56	18.63	0.29
Midgut	22.62	52.80	0.94	11.45	13.09	1.35
Ovary	2.07	6.24	34.72	6.47	54.33	0.26
Remainder	3.71	77.24	0.76	9.07	10.38	0.45

## Supplementary Materials

The following are available online at https://www.mdpi.com/2075-4450/11/7/406/s1, Figure S1: Fragments of tRNAs and rRNAs across mosquito tissues. Figure S2: Differential abundance of rRNA- and tRNA- fragments across mosquito tissues. Figure S3. Mapping properties of *AGAP006442*-derived small ncRNAs from midgut small RNA libraries. Figure S4. Mapping properties of *AGAP003387*-derived small ncRNAs across tissues. Table S1: Bioinformatic data for read abundance per small ncRNA group across tissues. Table S2. miRNA annotation update. Dataset S1. Conversion of Raw Reads to RPM Across Tissue Llibraries for miR-956. Dataset S2. Conversion of Raw Reads to RPM Across Tissue Libraries for rRNA and tRNA -fragments. Dataset S3. Transposable Elements retrieved from databases. Dataset S4. Reads for piRNAs across Repbase and TEFam annotated transposable elements. Dataset S5. Reads for mRNA-derived small ncRNAs across tissues.

## References

[1] World Health Organization (2018). World Malaria Report 2018.

[2] Aalto A.P., Pasquinelli A.E. (2012). Small non-coding RNAs mount a silent revolution in gene expression. Curr. Opin. Cell. Biol..

[3] Hirakata S., Siomi M.C. (2016). piRNA biogenesis in the germline: From transcription of piRNA genomic sources to piRNA maturation. Biochim. Biophys. Acta.

[4] Saito K., Siomi M.C. (2010). Small RNA-mediated quiescence of transposable elements in animals. Dev. Cell.

[5] Aravin A.A., Hannon G.J., Brennecke J. (2007). The Piwi-piRNA pathway provides an adaptive defense in the transposon arms race. Science.

[6] Han B.W., Zamore P.D. (2014). piRNAs. Curr. Biol..

[7] Senti K.A., Brennecke J. (2010). The piRNA pathway: A fly’s perspective on the guardian of the genome. Trends Genet..

[8] Lucas K., Raikhel A.S. (2013). Insect microRNAs: Biogenesis, expression profiling and biological functions. Insect Biochem. Mol. Biol..

[9] Lucas K.J., Myles K.M., Raikhel A.S. (2013). Small RNAs: A new frontier in mosquito biology. Trends Parasitol..

[10] Lampe L., Levashina E.A. (2017). The role of microRNAs in Anopheles biology-an emerging research field. Parasite Immunol..

[11] Bryant W.B., Mills M.K., Olson B.J.S.C., Michel K. (2019). Small RNA-Seq Analysis Reveals miRNA Expression Dynamics Across Tissues in the Malaria Vector, *Anopheles gambiae*. G3 (Bethesda).

[12] Bryant B., Macdonald W., Raikhel A.S. (2010). microRNA miR-275 is indispensable for blood digestion and egg development in the mosquito *Aedes aegypti*. Proc. Natl. Acad. Sci. USA.

[13] Schmittgen T.D., Livak K.J. (2008). Analyzing real-time PCR data by the comparative C_T_ method. Nat. Protoc..

[14] Giraldo-Calderon G.I., Emrich S.J., MacCallum R.M., Maslen G., Dialynas E., Topalis P., Ho N., Gesing S., Consortium V., Madey G. (2015). VectorBase: An updated bioinformatics resource for invertebrate vectors and other organisms related with human diseases. Nucleic Acids Res..

[15] Fu X., Dimopoulos G., Zhu J. (2017). Association of microRNAs with Argonaute proteins in the malaria mosquito *Anopheles gambiae* after blood ingestion. Sci. Rep..

[16] Reynolds J.A., Peyton J.T., Denlinger D.L. (2017). Changes in microRNA abundance may regulate diapause in the flesh fly, *Sarcophaga bullata*. Insect Biochem. Mol. Biol..

[17] Su J., Li C., Zhang Y., Yan T., Zhu X., Zhao M., Xing D., De Dong Y.-, Guo X.-X., Zhao T. (2017). Identification of microRNAs expressed in the midgut of *Aedes albopictus* during dengue infection. Parasit. Vectors.

[18] Jain S., Rana V., Shrinet J., Sharma A., Tridibes A., Sunil S., Bhatnagar R.K. (2014). Blood feeding and Plasmodium infection alters the miRNome of *Anopheles stephensi*. PLoS ONE.

[19] Dou S., Wang Y., Lu J. (2019). Metazoan tsRNAs: Biogenesis, Evolution and Regulatory Functions. Noncoding RNA.

[20] Lambert M., Benmoussa A., Provost P. (2019). Small Non-Coding RNAs derived from eukaryotic ribosomal RNA. Noncoding RNA.

[21] Babski J., Maier L.-K., Heyer R., Jaschinski K., Prasse D., Jäger M., Randau L., A Schmitz R., Marchfelder A., Soppa J. (2014). Small regulatory RNAs in Archaea. RNA Biol..

[22] Eng M.W., Clemons A., Hill C., Engel R., Severson D.W., Behura S.K. (2018). Multifaceted functional implications of an endogenously expressed tRNA fragment in the vector mosquito *Aedes aegypti*. PLoS Negl. Trop. Dis..

[23] Arca B., Colantoni A., Fiorillo C., Severini F., Benes V., Di Luca M., Calogero R.A., Lombardo F. (2019). MicroRNAs from saliva of anopheline mosquitoes mimic human endogenous miRNAs and may contribute to vector-host-pathogen interactions. Sci. Rep..

[24] Castellano L., Rizzi E., Krell J., Di Cristina M., Galizi R., Mori A., Tam J., De Bellis G., Stebbing J., Crisanti A. (2015). The germline of the malaria mosquito produces abundant miRNAs, endo-siRNAs, piRNAs and 29-nt small RNAs. BMC Genom..

[25] Biryukova I., Ye T. (2015). Endogenous siRNAs and piRNAs derived from transposable elements and genes in the malaria vector mosquito *Anopheles gambiae*. BMC Genom..

[26] George P., Jensen S., Pogorelcnik R., Lee J., Xing Y., Brasset E., Vaury C., Sharakhov I.V. (2015). Increased production of piRNAs from euchromatic clusters and genes in *Anopheles gambiae* compared with *Drosophila melanogaster*. Epigenetics Chromatin.

[27] Serrato-Capuchina A., Matute D.R. (2018). The Role of Transposable Elements in Speciation. Genes.

[28] Lau N.C., Robine N., Martín R., Chung W.-J., Niki Y., Berezikov E., Lai E.C. (2009). Abundant primary piRNAs, endo-siRNAs, and microRNAs in a Drosophila ovary cell line. Genome Res..

[29] Malone C.D., Brennecke J., Dus M., Stark A., McCombie W.R., Sachidanandam R., Hannon G.J. (2009). Specialized piRNA pathways act in germline and somatic tissues of the *Drosophila* ovary. Cell.

[30] Li C., Vagin V.V., Lee S., Xu J., Ma S., Xi H., Seitz H., Horwich M.D., Syrzycka M., Honda B.M. (2009). Collapse of germline piRNAs in the absence of Argonaute3 reveals somatic piRNAs in flies. Cell.

[31] Brennecke J., Aravin A.A., Stark A., Dus M., Kellis M., Sachidanandam R., Hannon G.J. (2007). Discrete small RNA-generating loci as master regulators of transposon activity in *Drosophila*. Cell.

[32] Wen J., Mohammed J., Bortolamiol-Becet D., Tsai H., Robine N., Westholm J., Ladewig E., Dai Q., Okamura K., Flynt A.S. (2014). Diversity of miRNAs, siRNAs, and piRNAs across 25 *Drosophila* cell lines. Genome Res..

[33] Rouget C., Papin C., Boureux A., Meunier A.-C., Franco B., Robine N., Lai E.C., Pélisson A., Simonelig M. (2010). Maternal mRNA deadenylation and decay by the piRNA pathway in the early *Drosophila* embryo. Nature.

[34] Klein J.D., Qu C., Yang X., Fan Y., Tang C., Peng J.C. (2016). c-Fos Repression by Piwi regulates *Drosophila* ovarian germline formation and tissue morphogenesis. PLoS Genet..

[35] Robine N., Lau N.C., Balla S., Jin Z., Okamura K., Kuramochi-Miyagawa S., Blower M.D., Lai E.C. (2009). A broadly conserved pathway generates 3’UTR-directed primary piRNAs. Curr. Biol..

[36] Saito K., Inagaki S., Mituyama T., Kawamura Y., Ono Y., Sakota E., Kotani H., Asai K., Siomi H., Siomi M.C. (2009). A regulatory circuit for piwi by the large Maf gene traffic jam in *Drosophila*. Nature.

[37] Hirakata S., Ishizu H., Fujita A., Tomoe Y., Siomi M.C. (2019). Requirements for multivalent Yb body assembly in transposon silencing in *Drosophila*. EMBO Rep..

[38] Girardi E., Miesen P., Pennings B., Frangeul L., Saleh M.C., Van Rij R.P. (2017). Histone-derived piRNA biogenesis depends on the ping-pong partners Piwi5 and Ago3 in *Aedes aegypti*. Nucleic Acids Res..

[39] Adelman Z.N., Anderson M.A.E., Liu M., Zhang L., Myles K.M. (2012). Sindbis virus induces the production of a novel class of endogenous siRNAs in *Aedes aegypti* mosquitoes. Insect Mol. Biol..

[40] Liu S., Lucas K.J., Roy S., Ha J., Raikhel A.S. (2014). Mosquito-specific microRNA-1174 targets serine hydroxymethyltransferase to control key functions in the gut. Proc. Natl. Acad. Sci. USA.

[41] Lampe L., Levashina E.A. (2018). MicroRNA tissue atlas of the malaria mosquito *Anopheles gambiae*. G3 (Bethesda).

[42] Mead E.A., Tu Z. (2008). Cloning, characterization, and expression of microRNAs from the Asian malaria mosquito, *Anopheles stephensi*. BMC Genom..

[43] Jain S., Rana V., Adak T., Sunil S., Bhatnagar R.K. (2015). Dynamic expression of miRNAs across immature and adult stages of the malaria mosquito *Anopheles stephensi*. Parasit. Vectors.

[44] Ruby J.G., Stark A., Johnston W.K., Kellis M., Bartel B., Lai E.C. (2007). Evolution, biogenesis, expression, and target predictions of a substantially expanded set of *Drosophila* microRNAs. Genome Res..

[45] Goodwin P.R., Meng A., Moore J., Hobin M., Fulga T.A., Van Vactor D., Griffith L.C. (2018). MicroRNAs Regulate Sleep and Sleep Homeostasis in *Drosophila*. Cell Rep..

[46] Monsanto-Hearne V., Tham A.L., Wong Z.S., Asgari S., Johnson K. (2017). Drosophila miR-956 suppression modulates Ectoderm-expressed 4 and inhibits viral replication. Virology.

[47] Curtis C.F., Graves P.M. (1988). Methods for replacement of malaria vector populations. J. Trop. Med. Hyg..

[48] Pircher A., Bąkowska-Żywicka K., Schneider L., Żywicki M., Polacek N. (2014). An mRNA-derived noncoding RNA targets and regulates the ribosome. Mol. Cell.

