# Supplementary material for: Global Analysis of Small Non-Coding RNA Populations across Tissues in the Malaria Vector, Anopheles gambiae
Source: Insects. 2020 Jun 30;11(7):406. doi: 10.3390/insects11070406 (PMC7411766; doi:10.3390/insects11070406)
Supplement: Supplementary file 1 [file insects-11-00406-s001.zip › insects-827855-suppl/Dataset S3 2020.docx]

**Dataset S3. Transposable Elements retrieved from databases**

Transposons from databases (1) Repbase and (2) Tefam

**LTR from Repbase**

>aara5_aa bel

gagaaaccaagctcaagggcggccgctacgaaactggactcttatggcgttatgatgatcccgacctacc

ctgtaacaaggctgcggcgatgaaacggtatgtatgcctaaaacaaaaattaagtaaagatcccgttcta

gctgaagccgtgcaggccaagatggaggagtacttagctaaaggctacatcagaaagctcgctgacaccg

tttcgatcgcgcgccagaagaacgatggtacctccccatatttccggtaaccaaccccaacaagccgggg

aaaattcgcatggtcttcgatgcagcggctaaggtaggcggggtaagcctgaactctcgtctgcttcccg

gtccggatatgcttgccggactgttagcggtgctgctgaaattcagagagaatcgtgtagctattgctgg

agatatacgagagatgttccatcaagtggccatcaaagaaacggatcaacggagtcagatgatcctttgg

gacagtggacgtcccggcaccggaccggcaacttacgtcgtgaccgtaatgactttcggcgcagcgtgct

cccccagtagcgctcaatatgttaagaacttgaacgcggacaggttttcagatgcatttccaagagcagc

agaatgcattaagcatgaacat

>adg_copia-70_aa-i copia

tgttaaataacaacgtttatttaactcgcgtcaggcagcactgccaaaaccggcagcactggtcagtact

tgttgtgtcaagtcttaacaactgtgcggtcagtctttgcatagttctagaataaagaccgtgctctgta

gagccaagtgttttccgcgtttgatttcgacctaaatattaacaggttatgggcccagcagtgctaacgt

gaaaacaaaagatggattcggtacgagtaatagtgccaaggttgtcgaacgacaactacgcatcgtggag

tttcaaggtacaaatgctcttgcagcgtgaggacgtgtggcatgcagtggaaggcggtaagccagagaca

gttacaacagcatggacaaaagcaaacacgaaggcgaaagccataattggtctgcatattgaggatgacc

aaatcacgcttatcagagattgtgaagatgcaaaaagtgcgtgggaagcccttaaaaagtttcatgataa

cgtatcggaggtgtatttgttaaagaaattaacatccctcacgctggcagaaggccagagcatggaacag

cacctgtccactttcagcgagttgattcaaagaatcggtgcctcgggcgaatccattcccagaaagtggc

aggtggcgatgcttctttgttcacttcccccctcgtacgacccgctcacaacggccatcgagctcacgaa

tataaacgagctaacagtggagagcgtgaagtcgaagttgctggcagaagccgaaaagagaaaagagagg

gcgggaaacatcgagagcgagaacgagaaagcaatgaggagtgaagtttttcgcagaaataagaaaccgg

cgggagtcgtttgttataattgtaacaagccgggacacctgaagagaaattgccgtctgctgagaaatga

tgaagcaaaacgagtggagaacaacaacgaaaagcccgcagaagagtgtgcatttacaataagccaggaa

gaagctaccgaatggtacgtagatagtggggcttcaaggcatatgaccggtaacaaggaatttttcaatc

aattctcagcaacaaacatgggcacagtgacactcgcgaacggccaaaagaccagaatcaatggaactgg

tagaggcaccctaatatgcaccgacagcgaaggaaaaccaagatcgatccttctctctgatgtactttat

gtaccgaaactaacaagtggactaatatccgtaagatctctcacgcgaaaaggaatgaccgtgctattta

caggtgaacagtgcaggataaaaggagaaaataaagaagttatagccacagctagtagtggaagtttata

taaacttcatacaacagaaaaggcactcaagggtttatttcaaacacaccacgaaaaatgtattcacagc

ctgcacaggagattcggccaccgagaatgtggtgtaatagaaaagatgtgtaaagaaagtggattaaagg

taaaggactgcagaataaaatcaaaatgcgagatttgtctaaaaagtaaaatgtcaagaaaaccctttcc

gaaaaagtccttttcagagaccagcgagatactgcagttgatccacaccgacatcagtggaccttttggc

tgtacagtgagtggatacaagtactatatgtgcataatagacgattatagcagaatgacgttcctatact

tgttaaaacacaaatcggaagcagaggacaagattcgtgagttcgtagccttctgcaaaacgcaaattgg

gaaaacaccacgagtgatacgttccgacaatggaggagagtatacaggaaatcagttgaaaaatttcctg

aaacgcgaaggtattgtcagccaactaacagcaccatattcacctcaacaaaacggagtcgctgaaagga

aaaatagatacatacaagaaatggtgcggtgtatgctagcagatgcaaaaatggaaaagaaattttgggg

cgaagcggctttaacagctacgtacatacaaaaccgcttacccaccacagcgacgggaatgacgccttac

gaaagatggtatggacaaaaaccatcttatgatcattttcgcatatttggatgcgaggcgtgggtgcata

ttcccaaagagaggcggaacaaaatgaattccaaagcagaacgcctaagatttgttggatactccaaaga

acaaaaagcatatcgattcgtcaacgaagatacaagtaaaattgtgatcagccgcgatgcagttttcaaa

gaaaactttaatcacccttcgtggaatgaagacatcttgaagaagaacaaaatgcgaaatcaagatcatg

atatcggagaaaccgacgacatcagtgacgttgattcgtgtgtttcaaacgaatcagatgctgtgagtga

tgatgctttcgaagacgcaaatggtgtttttccttcggaatctgaggatctaccatcaaccacagatcga

ccaacacgagaaaacagagggaagccaccagcgtacttggacgactatatagtcgggattgccaagagcg

aagatatagaaccgacatcctggcaagaagcgatgaagtcatcacatcatgacgaatggaaagaggctat

ggacgcagaaatgttttcacacgagaagaacagcacatgggaggtaaccgagctacctcctgacaagaaa

gcgatcggatgcagatgggtcttcaaattaaagaaagatgaactaggaaatgtggtacgttttaaggcca

gattggtggcccaaggtttcaatcaacaatacggtgtggactatgatgaaactttcgcaccagtgacgcg

tttcaacaccattcgtgtattcctgacgatatgtggaagagaccgtttgatcgccaaacaccttgatgtg

gctacagcatatctgcacggggacatcaaagaagaaatctacatgcgtcaacctcctggatattcactga

ccggacaaggaactaaagtatgtaagttgaaaagaagtatctacggattgaaacaggcagcgaggtgctg

gaaccagaagttatgtggtgttctggaaagtattggattcaaggctagtgtaactgatccatgtttgttc

atgcgtattaaagataattcaaaaatgtatctggtcacatatgttgacgacttactagtagcatgtaaga

atgaaaatgaaatcgataacgtatatacagaattgaagaaacactttgagataaatatgttaggtgatgt

taagaattttttaggagtacaaatcatcaaaaaccaggacggcattcttagtttgagtatgacacgtcat

atagatgaacttgccttaaagttcggtttagagaaggcaaaggtggcgcgtactcctatggattcaggtt

acctcaaatgcacggataggcaagatatgttcgaagacaataacaaataccgtagcatagtaggttcact

tatgtacatagcaaattgtggaagaccggatatagcagcaagcgcatccatactaggtagaaaacttgaa

tgtccatcgaaaacggattgggtggcagcgaaaagagtagtgcgttacttaatagggacacgcgactggt

gtttaagattaggtgacgggaaggctcgatgcgttttagaagtatatacggatgctgattgggcaggaga

tcatgccacgagaaaatctacaacaggctacgtccttttttatggaggaggagctgttgcgtggggtagt

cgtcgacaaaattgcgttagtctttcgtcgatggaggctgaatatgtagctataaccgaatcatgccagg

aaactttatggatacgtagattactgagggacttaggtgaagaacagttaggcgccacttttatacacga

agataatcaaggctgtataagtttcgcgcaatctggaaaaataagcaagcggtccaagcacatagagaca

aggaattctttgtccgtgacttaatcgacaggaaggaaattgaactaaggtactgtcctacggaggacat

gatagcagacatattaactaaagccttagggaatataaaacacaagaagttctcattatcattaggacta

acggatccagttaattaaacgttgaggaggagtgttaaataacaacgtttatttaactcgcgtcaggcag

cactgccaaaaccggcagcactgccaaaaccggcagcactggtcagtacttgttgtgtcaagtcttaaca

actgcgcggtctgtctttgcatagttctagaataaagaccgtgctctgtagagccaagtgttttccgcgt

ttgatttcgacctaaatattaaca

>afun1 gypsy

gcgttgttgaggccaaagggcatcttaagaaggattgtcgtagtgcaagcaaagcactcacacttaaaaa

cacaccaatgaatgtagtggacaataaaagtgtgtttgtaaaaacggtcagagtggaaggtgaaaccatg

atggcgttagtagattcaggagcacgtgtttcaacaattcagcaaaaatgggcagataaaataggaaatt

tgaagcctactcataaaattttaagagggtttggtagaaaagaaatacaggtgaattcaaaagttgtcac

ggaattactattagacaacgttaaactagaaatcgagcttcaagtagtgccaaattgggtacaggatacc

gcgataataatcggtgaagaagtgttgggaaaagaaggattagtantaacagtgcgtgcgggatcagtaa

ccttccagatggaaccaaactgtcgctcagaggaaaaagaaagagaagatgagaatgatagtgcgaaatc

agtaatgcacatgttcaccattaaagaagggtcaagacaggtcgtggatcgcaaaatggtgaattcagat

ggaagttacgaacacacggtcagattgataaaggttgtaaatgaataccgtgatgtgtttgcgttgaata

ttaaagagttagggtgtgcaaaatcgacggcaatttcactaaaattgacccaaatcgaaccagtgtatgt

gaaaccacggaagttggagtatgcgcgagaaatcatgttaaatgaaatggtcgaagaactgttagaggcg

gaggtaataaaggaaacagattcgccttataatagtccgattgtgttagtgccgaagaaaaatggacgat

ttaggatggcgatcgactatcggatgcttaacgcaaagacagtaaaggataaattccctatgcccgacct

cgaacagtgcttacagagattaagtggtgcgaaaatgtatataacgctagatctattcagtgggtattac

caaataccagtacatcaggatagccaagattacactgcgttttcgacgcccagcggacattttaaattca

cgcgtatgcccttcgcctcaccaacgcaaccgaatc

>agm1 bel anopheles gambiae

tcgttgctaaccaaaaccatccaacgaaaaacacattcaaattgttcgatctcaaatgcatgcggttcta

ccacagtgccggattatggcagctgacgttttgtcattcttgtgggtctagggacgtaccgctgtgaaag

gggaaccgcctttttgtggcaattgtcacgagccgaagaaaagcgaagcacgcataaatggtgtgtgaat

aaagaagtgaacttacttgccaacaaagaacaaccgactgattatttcgtgtaaaaaaaaagtaaaaata

tcacaacgtagggactttcacggaacattttggtgcaagtgaccaggataagtgcctgattatcggtgtc

aattgtggtaatcataattagtgcaaaagttctcgcttgttctttgctgtgtgcgtgtgagtgtgtgctt

ggagctggctaggattttgtgtgcgtgtgcgcgtgtgcaacgtattaaacagtgtgtttaacgcaagtgt

aacaatggcgacggcgcgagaagacaaaattcgtggcaaagagcaaaaaagaaaaaacattatagattcg

atgcggcgaatcgatttattcgtacaaacgtacactgtggacaagattcatgaggtgaccacaaggctcg

agaggctcgagaaagtgtggtatggttttgaagaagttcaagaagagttagacaagctaacccttgaggg

agacaacgctgaaaatgaaaagacgcgtgcagaaatggaggagctatacatgagtgttagatccaatcgc

tacggctgaagccatcgtctaatccaatagtcagcgacgttaaaccggtacccattgtgtcccaaatgaa

attaccagtaatacaattgcctgagtttggaggtgattttaatgattggttaccatttcatgacacgttt

gtgtccctgattgataaatcagatgagctttctggtgtgcaaaagctacattatttgaaagctgcgctca

aaggtgaggcagcacggctaatgagccaattctcactacagatgagaattacaaaatgcatggcaaatgt

tggtagaccgctatggcataaacatttgcttaagaaacgtcatatccaagccattttgaggctaccgaag

attatcaatagcaatctagatttattgcgacgcactgtagatgatttccagcgacacacattggtgttag

aacaactcggtgaaccaataaaacatcttagctcattccttgttgagcttcttagtgagaaattggatag

tgcttctcttgcggcgagggaagaagcacaagcggataaaagttacacatacagtgacatggttgagttt

ctgcgtaagcgtgtgcgtttgttggaaacgcttgctaacgatacgggtgaaacgagtaagcgacaaccgc

gtgtaaaagtgagtgtgaacactgctgcagcggccgagaaaaaagtggatatgtgtgttgtgtgtggaaa

gcaggggcatacaatagtgaattgtcggcgtttcaatgaattcgatgcaaagaagcgccacgaagttgtg

aggcagcacaaattgtgttggaattgcttgcagggcagccattttgtaaccagttgtacgtcaaggtatg

ggtgtcaaacgtgtggcaaacggcatcataccttgctgcatgctgaacgaagtagtagtgtaatagcaga

tgattcggtgggttctgttagtacgatggtgcttgctaatattccaatgcagtgcaactctactgaccga

agctcatattctaatgttatgctgacaacggtggtgttgtttgtagttgacgcaaatggtacgcaacatc

cagtacgtgcgttgttggataacggtgcgcagcccaatgcgataagtgagcgattgagtcagcttttgtg

cttgcgcgtatgcgtacccatgtatccattacaggtgtggatggaacgacgactcaggcgtcatgtgaaa

tgaaggtggaaatccgttccagatttacgcaatttgctctgaaactaaatttcttggttttgagcaaggt

aacagcaaacactccagccacatctttctccacatcatgttggaaattacctgctgggttggcgctcgca

gacccagaattccatcagtctggacgagtggatatgctaatcggtgcatcgcatttctacacgtttctga

gggaaggccggctcaaacttagtgaacatggtccattgttagttgaaacagtgttcggttgggtggtaac

aggtgaagtgctccgagaagaagctataattcagcaacaagcagctcagtgtcatgttatgttatcgtcg

gaaaacattagcgatcaacttgagcggttttggaagatcgaagagctgcatgtttcgcatttctcggcgg

atgagcagagatgcgaagcttattatgagcaaacggtatcacgagacgaaacaggcagatatatcgtgaa

actgcctaaacatcagcaacattctaccatgattggaaaatcagaaacgacatcacttaagaggtttgct

ggattagaacgtaagttatttgctaactcacaacttcgtcagcagtacaatgagtttatgttggagtaca

tacaactcggtcatatggttcctgtgtcgcctgacaacctggatgcagcaacctgctgctaccttccaca

tcaccctgtgtttaaggagacaagttccacgactaaaatgagagtggtatttgatgggtcagcaccaact

agcacaggacactctctgaatgatgcgctattggttggaccagtcatccaagatgatcttttgagcttaa

taatccgttttcgtaaatttcaggtcgcgctagttccggacttggaaaaaatgtatcgtcaggtacttgt

gcatccggaggatcgaccgttacagagatatggtggggcgtatgagttgcggacagtaacatacggtttg

gctccttcatctttcttagctacaagaacacttcagcagttggctgaagatgagggtgacgcgtttccta

ctgccaaggatacgctgaaaaaacaactgtacatggatgaccttattgctgggtcaaatagtgttgatgg

agctatacagctacgtgaggaattgagtgcactggcgcagagaggcggttttacttttcgaaaatggtgt

tcgaattcattagccgttttatctgacgttcccgctgaacaattggcaacaaaatcatcgttaaggttcg

acgacaaggagacaattagtacgcttggtatatgttgggaaccagaaattgatacgttccagttcaacat

ttctattactacgaaatcagagagagacaccatgcgtacgatactatcaatgattgctgaactgtatgat

ccattgggattgatttcacctgtaattattacagccaaagttttgatgcaatcgctctggcgtttgaagt

tgagttgggatgatacggtgcctgaggaacttcaaaggaattggattagatttcgagcagaattaccaga

acttaaagattttagtatccctagattcgctttcgctcatcaatatcggcaagcagaaatacattgtttc

acagacgcgtcagagcttgcatatggcgcgtgcatctatattcggtctgaagctgaggatggaagcattc

atgttaatttgctagcatcaaaatcgagagtggctccactgaaggcattgacgattcctagacttgaact

ttgcggtgcattattaggagctcgattgcatgagaaggtaatggctgcaatggagattaagttcgttgct

catcgattttggaccgattctaccgtggtgctagattggctaaatgctgaatcaaagacttggaaaacat

tcgtagcaaaccgagttgctgagatccaagcaattcgagatgctgtttggcaacatgtatctggacaaga

aaatccagctgaccttatttcacgcggagttttaccgcatcagctcatcaacaatcaattgtggaaacaa

ggtccacaatggttatcagagagaaaggagaattggccccaacaaaaagagagaacaggtcaaattacaa

cagacgaaataagatcgaatgtcgttttaacaacgcaaatacaagaaaaaaatgaaatatttacaagata

tgggtcataccagaagctaatcgacgtggttgcatattgttttcgttttgttcataatgctcgtcgtctg

caatcaagaataagcaatagtgcattgacggtaaaggaacttgctgatgctaaaaagcgacttgtaaaac

ttgtgcaagctgaagaatttaccaacgatctttataaaatccacaaggggattcctgttgctcgaaattc

aacgttgaagcttttaaacccgtttatagataatgaaggaataatccgcgtaggtggccggctcagaaat

tctgatttgaattataatattaagcatcaaatagttcttcctggattccatccatttactcaactcctca

tcatggacaaacatgtaaaagcaatgcatggaggaatatcatcaactcttaacgcagttagagatgagat

ttggccaatcaatggaaaaagagctgtacgtaaggtcatacgaaattgttttcgatgttgcagagcaaat

ccacaacctataattcaacctgaagggcaactaccagcagaacgtgttacagttaacgaggtgttcagtt

gtacgggtctagattattgtggacctttgtatttgaggccaacacaccgcaaggccgcaccaaataagtg

ctacatttgtgtatttgtctgcatgagtacgaaagcagtacatttggaattagtcggagatttgagcaca

aattcgtttctgatggcacttgatcgctttgtttatcggcggggtaaaccaaagcacatttattcagaca

atggtaccaattttattggtgccaaaaatgaacttcatcagatctacaaaatgctgttcaacgattctgc

agatagcaaaatagcaaaacatttggcaaaagaagagatacaatggcatttgatacccccacgcgcccca

aatttcggaggcctttgggaagcggcggtgaaggtagccaaaacccatttgattcgtcaactaggatcat

cgcgtttatcatcggaggaaatgactactgttttagtaaaaatagaaggttgcatgaactcgcgaccatt

agttccgctttctgaagatcccaatgatttgacggcattaactccagcgcactttcacattacaaacaat

ttgaaggttattctcgaacctgacttgaaagaggtgcctatgaatcgtctgggaagataccaactccttc

acgggtacacgcaaaacttctggatacactggaaacaggattatctgaaaaatcttactgttttgcatcg

gtcagctaagcaatctaagcaattatctgtcggagatatagttattctgaaggatgagcagcttccagca

gttcaatggccattggcacgtgtcgtcgaaatacaccctggagctgatggaatttcccgagttgcaacac

ttcgtacggcatccggtattgtgaagcgagcagtatctaaaatctgtccgttacaatgcagtaatcaaag

aatggattgaaaactgagtgtttcaaggtggccggtatgttcgatctcaaatgacctgcggttctaccac

agtgccggattatggcagctgacgttttgtcattcttgtgggtctagggagctaccgctgtgaaagggga

accgcctttttgtggcacttgtcacgagccgaagaaaagcgacgacgcataaatggtgtgtgaataaaga

agtgaacttacttgccaacaaagaacaaccgactgattatttcgtgtaaaaaaaaagtaaaaatatcaca

acgtagggactttcacggaacacaaattaaccagcaatcaaatcaggtgagacagaaaacctaccatgca

ataaatgcttagcacacacaaaccgtagacagg

>bel-20_ag-i bel anopheles gambiae str. pest

tggtaaaagtgaaaatgacgcaagccaaagcctcaagcgaagaaagtttccgtggtttctcggactcaga

aacggaaaatagcacgattatagacaacagtgcaatggctcttgctctcgcaaaggaggaacgagatggc

ttgattgaatgtctagtaagactagaaaaatttgtggaaaattcgggaatagttgagttggaacaagtcg

aagctcgattaaaacgtcttgagaggtgctgggagcattttgtaaagatttcaaaggaaataaggcagtt

tgacgaccagcacaacaagaaagaaaattttgctatatttgccgacttcgatgagcgtgtgtgtgtgcta

acaggcaaacttaagagaatgcagcgtagcagcaacgtgccaaagaaggaagagaatatgagcaacgtta

ctaacacatccacaggcggtgtgaagctccccaaaatggcgctaccagaatttcaaggtaaattcgatga

gtggctacagtttagggacatgtatgagcaaatggtgcacaacaacataactctttctaaagtagaaaag

ttatactatttgaagagttcactaaagggtgaagcattaaaagtgatcgaagcttttccgatatgcgcag

caagctatgatgctgcatgggacgctgtggtaagtcgttttgctaacccatacatacaaaagaaaagaca

tacgaatgagttgctaaactggccgagaatgaaaaaaccaacagccgcaaacatcaacgctgtaatcgat

ggttttgaaaggcacacaaagttattgcaacagttaggagaaacgccgtcaacatggggtatcatgctca

cgcagcttttaacatcaaagctcgatgaaggaactcagcgtgagtgggagcgaacagttgaggcacaaga

tgatgctaattacaacgatctaataaaattcttgcgaggacaagtacgaattttggaagctttgggcgaa

gataaaatagaacagcgaaaccaaccgttgccaacatcaaaagctccgaaattggcgattcacgtgacag

caggtgagaaaaagcttaaatgcaatgtttgcggtgcagagcattcaacagcaaaatgtgacgagttcat

ttcaatggcggtgaaagaacgaatcaagattgcgcgagcaaaagagctttgtctaaactgtttaggaaaa

ggccattttcgaaaccaatgcgcatcaaaggtacgttgtcgtgcatgtaagggtgctcatcattctcttt

tacatatgtggttaccgaaagaaaacactatgcctagcacatcagctcaaagcaacaacaatgttgatga

aacattcgtcaatgaagcacaaggtgcaaatcatggaaatgctacgctcactatggctgttacaagatca

ggtgtttcttgtggcgtgctttctacagctctagtaaatgtgaaagcaaagaatggtatgttcattcaaa

cgcgagcactattagatagtggctcacagctcaacgtcttgactgagaaactttgtagaaagcttggttt

acaacggcgagctagcggaatcaaactaacaggtataggaaagcatgaggtcagtagtgacatagctgta

acggcggaagttgcgtcgcagagcaacaattatgcgagaagaatggaattcttagtgatggacggcatca

ctcacaatcttcagccagtacatcttcctaacgcatgcattccgaatgaaggtaaaatagcagaccctgg

atggcatcagggtggagagatcgatatgttgttggggtcggagcatttctttgaatttctagcgctagac

ggcggccggcctggagtctatagaatgaacgattctcatccatattttgttaatactgtgttcggctggg

tattaactgggccaaagcaaacgcagcagaaaacatcagcgatttgttctcatgtgagtattgcggagca

aattgagcgattttggtcgatcgaagaggtgtgtccagagaacagcttgacccaagaagagaaagattgt

gagcaaagtttcgttacaacacattctcgtgacgaaacgggtcgatacatagtaaaacttcccataaagc

tcaaaggttgggaaaggattggagagtccttaggaacggcaaagaaaagatttctacaactggaaaaccg

attgtcgagggatgtggcactatacgaaaattactgctcaacgataaagcagtatcaagagctgggatat

ttagtagaggtatctcctgacatgtcacaaggtgacaacgtgaagccatgctatttaccacatcacccag

ttgtgaaattatccagtgctagcaccaaagtgcgcccggttttcgatggttccgctaaaacctcaactgg

ttttgcactaaatgattgcctcctaaatggacctgtgttgcaagacacactctttgatctaattgtgcga

tttagatcgtatgccatcgcgctcattgcggacatcgagaagatgtacttgcaggtaaaggttcatgagg

aacacacaccattgcaacgcatcttatggcgatcttctcaacaagaggagataaagcatttcgaacttca

gagagtcacatttggcctgacgtcatcatcctttttggcaactcgagtgctctttcaacttgcggcggat

gaaggagagcgatttccattaggtaaaagggctttacaagaatcattctacgtggatgactacatcggtg

gagcaaatagtgacgctgaagctgtccagctagtaagtgagttgcggcaactattggagaaaggaggttt

ccatttacggaagtggaactcaaataacaaaatggttttgcgaaacctgtctccagaggagaaagataaa

tgcaagcttgtgagtattggaccggaggatcaagtaaaaacgcttggcgtatattgggatccaactactg

attcgttaggagtagcggcagatttcgacgatgtgtcaaatgaacctcctaccaggcggaacgtgttttc

gttcatagccaaattattcgacccgcttggcataattgctccaattatcgcctgggcaaaaatcatgatg

cagcgtttgtggatagccacaaaggaatgggatgatccaatccctgtggatttggctgaccaatgggaat

tgtttaagaaacaattgtacctggtaaaggagatgcggattccaaggtatgtgatgcttcttgatcatac

caatgtgcaaattcattcttttgcagatgcttccgaagtagcctatggcgcatgcgtttacctccgagta

acaaatcgcgaaggcacagtaaaaataggtctccttgcagcaaaatctaaagtagcgccacttaagaagt

tgagcttaccacggttggaactctgtgctgctgtgctgggagcaaaactttggaaaactgcatgcatcgc

cttgaagcaacaaataacagaaagttatttctggagtgactctaccattgtgctgaactggttaagagct

ccatcgtatacgtgggcgactttcgtggcaaatagagtcgccacgatacaggacttaacccaaggaaatc

attggcaacatgtgaaaggaagtgaaaatccagcagacatcctgtccagaggtgctttacctaatcaact

cactaaggaatggttccaggggccacactggctctcgaataatgagtgcacatggtcatttcctgaggag

aaatctgacatcgatgaatcgcaactcgagcgaaagcgacaagtggctgtaatgacgatgggcgttgcag

aacatcctttcctggatcgctattcctcctactggaaatgtggtagagtggcagcttattgtatccggtt

tgctcaaagatgcaggaatgagccattagtcagcggtccgattacatataacgaatttattagagcgatc

cgacgtttagtcgtcgatttacaacgaattgaattccatgttgagatgcgggaattagaagaaaagggag

aattgcttagcacatcaaaactaaggaagttgcgaccgtttctggatgatgaaggtgtcctacgtgttgg

aggacgtcttaggcaaacaaacatcagatacaacacaaaacatcccatgattcttccggcaaagggcaac

tttacgcgaattgttgcaaaggcataccatgagatggcgctgcatggaggtcctcgcatcaccctggcaa

caatgaggcaagatttttggcctttaaatggcagaatattggcaaactacatacaccgaaactgtttaac

atgtttcaaggctaattcaacaccagttgcgcagccaattggccagctaccagttaagagagtgacacca

gccaggccatttctgacgactggtatcgatttctgcggccctgtgtatttaaaaccagttcaccgtcgag

caacatctcagaaggcatacatagcagtttttgtatgtttttgtactaaagcagtgcatctcgaactggt

tgaggatatgacaacatcagcatttttggcggcatttagacggttcatttcgcgtcgtggttatcccagt

gatgtctatacggataacggtttgaattttcgtggagcgcagcgtgaacttaatgacctttttcgtttgt

taagcgacgactctttccaggcaactacaagggaagaaacgatgaagtgtggaatcacctggcacttcat

acctccgagagcacccaactttggagggctatgggaggcggccgtgaagtcgactaagaagacgcttacc

aaactgtttggatcccagcgattgtcatttgctgacatgtctaccgtgttgacacagatcgaggctcagc

ttaactcgcgcccactcacacccttatcggaggatccagaggagctcaacgtattgacacctggccattt

tctcattggagaagctctattgacgttaccagatgctaatcacgtgacaacacccgagaatcgtttgaag

cactttgagcagctgcagcagctcgtgcaacggcattggcagcaatggtcgaaggagtacatttgtgagt

tgcataatgtcagtcaaaagggattacgaagcaagaagatcgagataggtcagatggtaattgtgaagga

ggatttaccaccaaacgaatggtgtttaggcagagtaataggagtgcatccaggtagtgatcaggtagta

agagtagtgacgattaagacgtctaagggaacttacaagagacctgtgtcaagggtgtgtctgttaccag

atagtgaagattagattgttgaaaatgtttttattgaaacattacttcaaggcggccggta

>bel-20_ag-ltr bel anopheles gambiae str. pest

tgttgggaatatgtaacgatcaaccgtcaaagttagcgaattcaaaatattaacgaacttaatgacattt

aacgaacgaaatgtcaaaggtaggaagacggctgcttgggaagtgtcagattgaaaaccgcatggaacag

aacacaacgtagggcagacaacacaacaacacattcctgaataaaagaattctattttcacttaaatcac

cac

>bel-21_ag-i bel anopheles gambiae str. pest

tttcaaaaaattagcattcgcgcgaaaaagccaataaagttgatcgaaaactcgtttcgcagagcagaaa

gcacagtaagtcgcgcgcgcaagtgatatatagtggaacgggaaaataaaccaacgtaaaatgcgtcgta

cgcttcgggtggagcaaaacctaccagcgagtccggaaacgccgcaggaaagcggtaacgtctctggcgg

gcccaggtacgatccatcctccccgggaacatcgagcactgtaacgacggaaggaagcaccccgaggagc

gatgccaccacaacgaacgttgcggtactttgcaaagaggggcagagtaaggggataagttcaccgttcc

acgcattaaaccacggtgcgaaccagggccgcgatgacattcgcgaatcgcgaagacgacgattgcagga

gattgagcacgagatgcagatgctacgggatgcagaagacgaaaatggctcggcggccatcgctgagcat

cccaaccacggcggcactggggggacacattatccggagtttgctgggtgggtgaagagtttcgaggaat

ccttgcgtcgaatatacccggcgggaaaagcccttacgcaaggccaaattccagggggcaacgacctcgg

gcaacgcgccgctgctgagcaagcccaactccctcaaccgagccccgcgattgcgtcgacctacgctaca

ctaccgtcacaaaggatgcatcctcacgcaacacaaactcttcctcacgcgacacattctcactcagcgc

acactatacagacacacccgacgtacacttcgcatacacattcggctcacacgatactaccacaacacga

atatgtgcaaccacatttcaacaccagcacactgagtcagagccagatagctgctcgtcaaccggtgccc

cgggaccttccgatcttttccggggaaccagaagcgtggtcgtttttcatagccacgtataaccgcacta

acactgcatgtggttacaccgatgacgaaaacattggccgtctacaatatgcgttgagaggagcggcgtt

cgaagcggtaggccaccttttgtccttcccggacgggttgaatgaggtgatggcgactctaaaggcgcgt

ttcggcagaccagatctgattgtggagtccatgaccgagaagatcagaaaaatggcaccaccgaagattg

aaaggctgtcaacggtcgtagagtttggatatgcggtgaagcggttggtaggaacgatgacggcctccgg

gttacgagggtacatgtacgacgtggcgttactcaaggagttagtcaggaaactaccccccgtcctatgc

atcgactgggcgagaacgaggagtaaactttcggaagtgacgcttttggagtttggaaaatgggttggtg

acttagcagaagatctctgtggtgttgtcgacgtgatgtccatcatggacaatagcgactcaactcatca

acaaccagaacccccaactcatcacagccgcacccagcctcagcggtttcaaccccatcgtgccccgcca

gcgcaactcgatcgacggccaccgatccgtactgggcgggttcatctagcctactgcaacgcaacggtat

tgcaggatgagaatcttggtgatccatcttcatcgcaaaatacaccagaattgctgactgtctgtccctt

gtgcggcagggattgtcccactctggtccagtgtgagcagttccaaaggacaacggttgccgccagaaga

tcgtttgtaggtgaacgcaggatatgccggaaatgcctcggataccatagaggtggatgcagcgtaagag

caccgtgcgcggtgaacggatgcaacagacaacaccatgagctgctccacgtaaatgaccagacaccagc

cagccctcaccagcgcgatcatctaaatggctcaggtaaggtcgtgtacactggtgtcgctaacgttctt

acccattcaggaccgaaagactcttcgcttttaaaatacgtacccgttacgctgcacgggcccagggggc

gaatagatacgttcgcctttttagatgacggctctacttccacgtttatggagcacggactagcacagga

gcttggagttacagggacaccgtacccgttgtgtctacaatggacgggagacgtgacaagagaagaacaa

gactccgtaaggctatcggtgcgcatatcgggtagaaacatgtcgcatgcgatatacaaactatcagaag

tacacacggtcaaagagcttgcccttccggaacaatctgtaaacgtagcacaactgactgcacgctatac

tcatctgagagatctccagttcgaatcctacgcttccgccgtgcctcgcatcctcataggcatagacaac

tgcaacataacgcgaactctgaagaccgtggatgcaagctgcaacgaaccggtcgcttcgaaaacccgcc

ttggatgggttgtctatgggccttgctcggttgcgagtgcaaagccgagtccttcaaaccggtccttctc

gggccgcccgtttcacgacctctcttgtaactccgcgtccgacaaagcagtgcggccgttgcggcctagg

gacgacgaaagagcgttggcgatattggagcgggaaacgaacggacagcgatacgaaatgggacctttgt

ggcggtacgacagcgtcaatttgccctacaacaaaaaggcgttcaagcgtgatgggtttctaaaacagaa

gatggcaaaatattcgaagttagcaaacgatgtgaatagaacaaatgtagcagagtttttacctacactt

caccacagaaccgagcggtccgaaccggtgcggccgacagaaataggcgatatagttgtagtggcgtgta

acaattcctaggaattgttggcctaagggaggaatcgtggtagtaaaccctgataggaatggacaagtta

gtcaagccaccgtcaaaacggcccattaaacgtacgataggtctacgcacggatgtgcaatttccttaga

gtaattggggtaaaaccgcgtggttacgacgcaacagatcgaacagtgagcagacagtaaacaaaccaat

gcacatacacatacacgcaaaacacctgacagataataggaagtgtagccacggttgatagtgtcgcagg

ctaggatggcgaaaaaagaggttgcaccatggttaagcgggattgacgtttttgtgtaacgggctataga

gcttaagcgggttacacgatcagcgtttacgggctataagctcaagcgggttacacgttcagcgtttacg

ggctactaacaaattcaaccgcctcaaccgtggtacactggggggacaa

>bel-21_ag-ltr bel anopheles gambiae str. pest

tgttggaaactcgtgagtgcccaacagtctgtcaggtgacaacgctagagaagagagtgagagggaggat

aaaaacggtaaattaggagataaggagaagggttggtccgctcctaaccagttagttgaaattcgacaat

cggtacaagtggactaattttttgatttaacctcataccacagtaaataaagtgttttctctaaaaaatc

caccgcgtttttcaaca

>bel-22_ag-i bel anopheles gambiae

tatggtgcttcgtgacacaggattgtgattgtgatcgtttagcgtggttttsgwttwgttgtttggagtt

tttttgcgttagtgaagtgawatggcaacgtctaaaggccactcggacgagtcggatgaggagttgcgtg

gtttcgcagatgaagatctggaaaccgcaattaatacagtgttggtgaaagaagcatccttacttcaaca

ccactctacggagtcggataagaagatggtcaggggagcctcgggggagggccctaagacaggagcattc

gttgagcccaacgcggtgttagaagtgaaaaaagctgaacgtgatggcataatccggtgtttgaagcgtc

aggaagtgtttttgcaaagttacgcacaaagtgacgcaatgtatgttccaacaaggttgaaacgtgttcg

ttcgtgttgggaacaattccaagaagtaacccgtgaaattcgttcgatcgagggaatagtccaagcaaac

gaagagctggtggacgaaatcgacgaacagtgcatgttgttgatcgggcaattcgaggaaaagttgaagc

gtccagaaagtgacagtgcagtggcagtacgtgttttggacgaacaaaaggttcggctaccgcctcttgc

gctgcccgaattttcgggaaattatgatgaatggttgcctttttacagtttgtacaaaacggcagtccat

gaaaacaatagtttgacggatacagaaaagataatgtatctgaagcgtgcgttgaaggaggaggcgttcc

gggtagtggacgcttttcctacgtgtggttcggcgtatgaagccgcttggaaggctctggaaaaaagata

tgccaacgaatatcttttgaaaaagcggtacgtaaacgaactgttgaatatgccgaaaatgaaaacgcgc

aaaacaaaagacatccacagcgttgtggatagtttcgagcgaaatgcaaagcttttggaccagcttggtg

aaaaaaccagtggttggggtatgcttttgactcagcttcttttgtcaaaattggatgatgaaactcagcg

gaattgggaacggcagttagagagtgatagcgagtgttcggtgcaaagtttgttcgattttcttcgagca

gaaacgcgtgttctggatgccatggcggttgaccaacacagtttgggtgttacgaagaaatctgatcgtc

gctttgttaatcttgctctcaaaggagaaacgaaatgtgcgcagtgtagcaaacagcatagcattgtggt

gtgcaggtcgtttggagagttaacaatcgatgacaggttgaaggtggctatgcagaaaaggctttgttta

aattgtttgtcgccaggccattttgcgagcaagtgtttttctaaaggtcgatgcggcacgtgtaaaaaac

gccatcattctttgcttcacaaagaagacatgcctgcaatcgaacaagtgcaacaagtgtcaggtgaaga

agaagagcaagaagtgtccgcgggggctagcagtatgttagccgcgctacctgcgctgaaatcgtctacg

catgggcaagtgatgttatcaaccgcgattgttcgtattcaaggaaaaggtggaaaatggttttccgtta

gagcccttttggacaatggttcgcagatcaacatcatgacagcagatctttgtcgacgtttgtgtttgcc

caaagtgcgcggttcggtaacagttactggtataggaaagatcggtgtgcaaaatacccaaatggcaaag

gcgttagtatcatcggaaggaaggacgttcatggaacaagttgattttgtagtgctcgaacatattacgg

aaaatcagccttgtggtgatatgcgtttcgatgtgaaaaggttgccgggtaacatggtgttagcagatcc

taatttttataagaagggaccgatcgatctgttattgggtgcggagtattttgtggacattcttcagcac

gagtgtaaaattgttccagcaagtagtgatcacccgggtttcgtgaagactgtgtttggttgggttgcgt

ctggcagaacaagttttccgaagaaccagcaagccgcatgtcaccttgtcaccgctgagccgtcgtcgga

aaatttgacggaatgtttggagcgtttttggaccattgaggagctaccagacaaacctcgattttcgcaa

caggagaaggattgtgaggagagttttgcacaatttcatcatcgtgacgacgaaggcaggtatgtagtta

atctgccttttaagattggcgagcgtgagccattaggcgagtcgaagcagtgtgcgaccaaaaggtttct

gcagttggagcggcgtttgaaaagggatccgtcgttgggacgagattatgcagcagtgattgccgactac

ataaatcaaggttttttgaagaaagtagctatcgacgaaaccggtgatgaccgtcagcagggtttctatc

ttcctcatcacccggttatcaaaagagctagcaccaccacgaaggtaggccagttttcgatggtccgcca

aaacatcgaatggtgtttcgcttaatgagccttactgaaagggccgtcattcaggattcttgttggacat

attgctacgatttcgatgaagcatgcattggcggcggatatgagacaaatgtatctgcaggtaaaagtag

atccgaagcatacgcgttaccagcgtatattatggcgcgaggatccagcattgccgatagaagtatatga

gctacagcgtgttacttttggtctgactccatcgtcattcttagcaaccagggttttaaagcagctggcg

attgacgaaggggacgcctaccctagagctcagcaagcgttgctacaagatttttatgtggacgatttct

tgggaggagcagacaatgaagaagaagcgcggcagctggttgatgaattgttaaaactcatggcgaaagg

aggattccattgcaaaaatggagttcaattcatctgctgttctggcacaagtggcatctgatgattggtc

acgtgcaaatgtagtgaatctgagctctgaggagcckgtaaaaaccttgggaatagcctggcaaccccaa

gatgacgaattgtttgttgaggccagcattcctgtatctgatgagccatggacccggcgacgcgtatact

ccatggtggctagattgttcgaccctcttggccttctagcgccagttaccgcatgggcgaagatccagat

gcaggctttgtggatagctaccgacgattgggacgaaccaataccgacgcaaatggaactcctgtggaac

cagttccaggatcagctaccccgtttgaaggaaatcaaatttgcgcggcatgtggttatcagcaatccgg

tgagtgtccaatttcattgtttttccgatgcttcagaggcggcgtatggtgcctgtgtttaccttcgatc

aatggacggcgatggtaacgtcaaagtggagctagtagcagcgaaatccaggccagcccctttgaagaaa

attagttttgccagactagagctgtgtggagcagtattagctacgaacttgagaaagtgtgtcggcagag

cgctcaagatggaggaggctgaaaccttcatgtggacggattccacagttggttttgcatggctgcgatc

accttcttacacgtgggctactttgtggcaaaccgcgtctcggcggtacaggaaaacggaaagggctatc

atggctacacgtcagaggcaggagaatccggcggacatcgtttctcgggggcgcttccgagtgaagtgat

agcatcagttcgtggttccacggcccttcttggttgatatcttcagaggacaagtggagaaagtcgatgc

cgttggatccccctgacgaaggcgaccttgaaaggaagcgaaaggtcttggtagcagttagcagcgaaag

gactgaggaatgggctgagaggttttcgcagttttggagatgcctcaaagtgacagcctactgcctcaga

tttgtaaaaggatgcagaaagtccggcgaagtgtatccaagtaagtgtctaactatgtttgaaatcgttg

cagctaagatggctttagtgaagatgcttcaacaagaacacttcgtagcggagataaaggagctagcgag

tgggcgtgtcattccaccgagctctaaattgaagaaattgggagcatttctggataaagaaggattgttg

cgggttggtggacgtttgtcacaactagcaattccttatgcggagaagcatcctctgattctgcctggca

gcgcacatctgacgaagttactggcacgtgtgtaccaccttcgagctatgcatggtggtccgagagcaac

gttggcagctatgagacgggaattttggccaatcaacgggaggagcgtggtgaacggcgtttgcagaagc

tgtgttatttgtttcagagcggcacccacgacagtggcacaaccttcaggacaactaccggaaccgcgcg

ctactccttctcgaccgttttcggtggttggcatggactactgtggaccattctatttgagaccggtgca

tcggcgggcggcggcgcaaaagagctacatggcagtcttcgtttgcttttcggtgaaagcaatacatctg

gagttggtggaggacttatcgacggcagcgtttatggcagcgtttcgtcggttcgtttcccgkcgggtct

tccatccaaggtctactcggataatgggctcaactttcgtggcgccagcagtgagctgaaggagttgtat

gaactcctgaacgaaccagagaatcaacagcgtatccagaatgcagctctccaagatggcatcgagtggc

acttcatcccgccacatgctccgaacttcggtggtttgtgggaggcggccgtaaagtctgctaagcggat

cctgcggaaagtgctgggcaaccagagactttcttttcctgagatggcaacggtattaacgcagatcgag

gcgcagctgaacagtcgtcctttgacgccactatccgaagacccgtcggaaatggatgtccttacgccag

ggcatttcttgattggggcacccctgacggcgctacccgagaaggatgtctccaatcaacctgaaaatcg

tctgcgtcgttatgaactgcttcagcgattggtccagctgcactggaagcgatggcagcgagaatattta

agtgaactacataattatgggcaacgtgtatcgcctgttaaaagagttgaagagggacaggtagttcttt

tgaaggaggataatgtgccagtttgcgaatggccaatgggaaggatcgagaagacttttgtagggccaga

tcgggttgtacgagtagttaaagtgcgtacgcagaaaggcagttataccagaccaacttcaaagatttgt

attttgccaattgaggggtaaatttatcctaaatttagaggggcgcca

>bel-22_ag-ltr bel anopheles gambiae

tgtttggatttgaatttatcgtacattttgtttaggagaatttgctgcttgggtcatatttgacagctca

gtgagggatacagttccgatccgcacggaatgatcgattccgttgtgacgctcatcactatcaatttttt

gtaaggagaagacagacgcttcgtgaactcgagacaagaaagtcgtacttgtgcgcaacacttaaacacc

acggattttgttcagcccttttttcattaacttcactaaaactttgtaactttgtaacacaacgtaggac

taaaataaaaactagcaaacgtaatcaaacccgtctaaaca

>bel-23_ag-i bel anopheles gambiae

ttttaaaaaaatactcgagtgcaagtgccatttcagtgcttcgttagcgtgccggaaagttcttgaggca

agtcagaacattaagaaccgtgcaagtgaagttttgtgaaatggcgcccaacgatattctatgcgtaatc

tgcgccgcgcaaggcgaagaagattgcgtactttgtgacgagtgtcgtgaagcctatcatcccaagtgtg

ttggtgcggacgattcggttagaagtcgcgagtggttctgtccgaaatgtgccgcaagatccagccagaa

ggagacaggcttgccaacgacgtcattccccagcggtgataacccggggagctccacgcccacgcatcag

agggatatgatgcaggaaatcagcctgcttttcgaccaaatgaaggcacgtatggagaaaagcagtgagt

ctcctcagaccccgcaggtcagaacgcgttgctacgcttgtgagaagacggacgcaggaaacaagatact

gtgcaatccttgtggtagatggtgccacatctcctgtctctccgaggacgaggcggcatcgatcgcatcc

tggaaatgcgccctttgcaccgtcgctaggatgagggcatccaccaacgttatggatcgtctagtggatc

ggttggaaagactggagcagaaaatggtggtttcagccatggacccgcgtccctcttatggcattccgcc

accaagttcaaccgtctttggacgttccgctgagaccagccaagagctgtcccaaagccaagcctcggcg

aggcatacaggtgcgtccaagttgccacccttttctggcaatccggaggaatgggacatgttcatctcag

cgtatgaggaaaccacccgcctttgtggattcacagacggcgagaacatcattcgcctccagcaggcgtt

gaagggtcaggcgctgaaggctgtgcaactacgccttcgtaaggccgaaaacctggaggaagttctagac

accttacgcagcagttacggacgaccggaactgatcgtcaatactctgctggatcaaataaaaaatgcac

cggtgccgcggatggaaaagttggacacccttgtgagctatgccctgatggtagaggagatcagtgcggc

agtgcggaccggaggcttagaaaaccgatacgatggtccattgctagaggaacttgtcggacggctgcct

cccattatcgcattcttatggggaatgcagcgtttgggaaaacctctagccagcttatccgattttggaa

cctggatgaaaagtgctaaagaagctgcgcttatagcttctccagcagcggcgaaaacggacgatagacg

gagggcgagaaacatcaacgtgcatatcccggctgagcggactgctcaacatgaaccagataaatgtgca

tgtgatcatcgatgcaatcaattggagtattgcgaggccttcctgcagctgactcccaacgaccgatgga

agcttgtaaagggtgaacatctgtgcagcatatgcttgaggcgccaccggaccacctgccgaatcacgag

gtcttgcaacaagaacggctgcacgaagagacatcatcctctgttacactcaccaccctcaccggaaggg

caaaacggtaccaggtatgagtctaatctttcccattctacaggaagtgctgacgaagtgttgttgcgct

atattcctgttgtactacacgggccgaaacgaacgttgaatacggtagctctactcgatgaggggtcgtc

cgtcaccttgatggaacaccgcttactaaaggagctcggactagaaggagaatcgaaacccctttgccta

agctggactgggggtcaaaaccgcgaagagagcgattccgtagagacatcgatcatgatttccggtgcgc

gtaagggagaccgtcgcttcgatatgcacgccgtgcggacggttcgcaccctgggtcttccaccacagtc

tgctgatggatgcgacatatcagcgcgttataaacacctgcagtctctgcccctgccatcatacgtatct

gccgtcccaagactactcatcggcatcgataattacagcttgtctcgacccctgaaaaccgttgaaggag

aggtaaacgagccaacggcaacaaaaactcgattaggctgggtagtgtccggttgtttcggcaaagcaac

tcattctgcgaaacaacaagttgtatcgatgcatagacttcatccgtgcgattgtcgggacctggaagct

agggtagaagctgcaatcaagggaagctttgcgttagaagatgttcactccagcaacgcttcggcattta

tgtccaaagaagatgaacgagccttactcctactacgcacgtatacaaagcaggtagaggggcgatacga

aacagggttgctatggcgcttcgataggtgagatgcctgataatagaggaatggctttaaaaaggctgga

ctgtctagaacgacgcatgacgcgcgaagtccaactacgaacgaccttaaacgagaagatcaaagagtat

ttagaaaaaggctatgccgaaaactgtcactggaagagaaacaagcgatcggtcccagaacgtggtatct

tcctatctttcccgtggtaaaccctaacaagccgggaaaggtgaggttggtttgggacgcngccgccaaa

tttcatggggtctcgctgaattcgatgctgctaacaggtccagatctactgtcctcccttccatcagtac

ttcaaagattccgtgaatatcgagtggcggtggccgctgacatccgggaaatgttccaccaggtacgcgt

ttgkgaagacgaccaccatagccaacggttcctatggcgatggaatggagacacaaatgcagagcctgag

gagtatgtcatgctgaggatgacctttggngcggcctgctctccaagtacggcgcagttcgtgaagaacc

agaacgcggagaaatatgccacntcgcatccccgtgcagtcggctgtatcaatnaagaacactacgtgga

cgacatgcttacnagtgcggaaacggaancagaagcgattgagctggcgtatsaggtgagcttaatacac

ggtgatgctggattccctctccacaactggctgtctaactctccagaggttttcaattatgccacaggaa

gcacatctactcaaaaggagctcaacatcaacgaatatctgcgacacagaaaatattaggtatgtggtgg

gacatacaaaccgactcattctgcttccgatcgcctatgctaaactccgagcttctgcaagggaatatgg

tcccgacgaaacgtcaagtcctcgggtgctaatgaggatttttgatccsttaggcctcatcgcaggacta

ttactgtacctgaaggttactgctccaggaggtctggagaagtggtactggatgggatgaggcaatcccg

tatgagcttaaggaaaaatgggatgaatggctagaacgtctgccggaactggaaagaataaccatccccg

atgctaccgaaagccggcgtcgctcacagagaccatctaaactacagctacacgtgttcgtagacgcggg

tgaagatggtttcgccgckgtcgcctacttccgatttcaagcaaggacgatgtcgaggtggcgctmgtcg

gcgcgaaaaccagagtggcgcctctgaagtacctgtcagtcccccgactagagctgcaggcagccgtgat

gggtgccagactggccaagactataaccaatgctcaccgagaaaaagtcacacggcgcttcttctggacc

gactcgaaagatgtaatatatatggatcaactccgatcaccgaaggtacagtacattcgtagcacacagg

gttggtgagatgctggaaacgacggaggtcgacgaatggcgatggattccaaccaaamgcaacgtsgccg

acgagggtaccaaatggacgaagctgagccgacatctcacgkccagcgagtggttcagtggaccccagtt

cctacgacgacccgaagcagaatggccaacagacaccgtagactgccataggacaaatccgaggagctgc

ggaaaaacgatatgctgaagctggtcggcgtccacatagtgcgagatccggttttcatcgactacgagcg

attttcaagatggtcgcggctggtacggtcgatggcttacgtgtgtcggttcgtaaacaattgcagaata

ccgaaggcagataaatctcgatcgagaggtgggaccgctaacaccgcgacgaaatccagcgggccgagac

ggtaattctgcgagacgttcagaggaaagctttccacgacgaatatgccatacttctggaaggcgaggga

acacccaacgacaccgtcgtagacacgtctgactcccaggagtagtttgttgtacaaacgaagcccctac

atggacgagaacggaatgctgagattgagcggaaggatcgacgctcgttgcctttaccagccaggagacg

aagcgccctattatccttacctaaacgacaccgcattaccgagttgatcgtcgaccgatgtcatagaagc

acggccatatgagccacgaaactgtagtgaacgaagtgcgtcaamgcttcgatatacccgctctgcgagc

agcgtgtcaccgagtaagaaacgggtgccwgcaatgccgaacatgcaaaatgctatacgcagagccagcg

tccccgatgatgggcgaatctacccgcagctcgcctcgctgccttckccaggccgtttwcgcatacaggc

atcgactatttcggcccgatggtagtcgtgaacggccgaaagacggaaaaacggtggggtgttttgttca

cctgcttgacggttcgagccgtccacatcgaactggcgcaatccctgtctacgagcgcctgcatgatggc

agttcgaaacttcatggcacgccggggaacaccaatcgaaatcgtatccgatcgtggaacgaatttcgtg

ggcgccggtcgcgaacttaaggaagccgtggaacgaatagatgtcggcaagatcgtagatggaattagcc

tccccmgatccggtgtggacgtttaaccctccmgcagctccgcatttcggaggagcatgggagcggctcg

tccggtcggtaaaaagaacgttgacgaacatccagttcactaagtatccgaccgatgccgtactcaacag

ttggttgatcgaagttgagaatatcataaattcacgcccattgactgatgttccggttgatcgcgaggaa

gacgccccsttgacaccaaaccactttcttgtgggctcatcatccggtgccaaaccgatcaccacagtgg

acscctcgcccgaagtgttaaagaacacttggaaaacatcacaaatatacgctgacgtgttctggcgaaa

gtggttggccagctacctgcccactttaacacggcgcacgaagtggcacgcaccggtgaaaccgatcgca

gagggtgacatcgcactaatagtcgacgagactctcccccgtggatgctggccgaaaggacgcattgtaa

atgtgatcgaatcaaacgacggtcaagtacggcgggtgcacgtgcagacagcgtccggtaaagtgctcga

gcgcccagctgtcaaaatagcagttatcgacgtatcggacaaatgaagtgttgtttacaaacactggggg

gac

>bel-23_ag-ltr bel anopheles gambiae

tgttgacaaacatcaacggtccgttcatcgtgatgctagtttgagcaactaaacatagagggcgctgact

tcgtagagaggatgccatcgatgccacgatcgtagggcgaaatatttcaacatgactcagccatatttgc

aactgttagcacgaagattaaccgaagaaccaccgaagggaaagggcatcgaccgtagcgccgagcggga

acgagcgaaagggtacttaaggaacgacggctgaagaagcgcccttttctcgcgtactaaagtcccggaa

gaaagagtatttttttaacccttccgtttgaataaagtgttaaaagaactttttgcgtttactattcgcg

gacttttcaagttgagaaaattctgttgctcgaggcaaca

>bel-24_ag-i bel anopheles gambiae

ttggtccttcgaaccggatgttagaaaagtgttagaagtgtgcatcatgatgactagaacatcgaagtct

gcgaaggagaagcccgacgaggaacatgcggaagaggtgcaatctattgagattgatccggacgcgcatg

gttttatgcgacaagcagcaatcgacgaggtggtgagaataagggacactttgctaaaggccgctgacga

agggagcgaattctccaacgcacggttgagaatatttcaaaggtcagtggaaggtgcaagtgtacagttt

ctcgatcaccatcaacgtttgttgaaaagcttatcagcgagtgatcgtcagagtgaaaccgcgacttacc

aggaattccgtgttctatacgaggacacaatgacggaattggagtcaacgttggaaggccgatcgccgcc

tgtggttgaaaagaaagaaccagaaacaatacccgtgcaagcgagtgcgcctgtagtggtgcacacatcg

ttgccgcgtgccattccatcgttcagcggacgatatgaggattggcaaaagtttaaggtaatgtttcggg

acgtggttgataaaaccaacgaagaaccacgaatcaagctctatcatttggaaagagctctaacgggtga

agcatcaatgataattgatgcaaaaacgatcagtgatggcaattatgatcatgcatggaaattgctcgaa

gaaaagtacgacgacaaacgacggatagtggacttgcacatcaagggacttttggatacggaaaagatga

agcaggaaaattatgagtgtttgcgtgaactggtgacgggtatagagacacatgttgcgaatctgaagtt

tcttggtgagagttttgatggtctgagtgagaaatttgtggtgtacattatttcacagtgcttggacaat

gaaacgcgcaaacaatgggagtcaactgtatcacggaacgagttcccgaaatacgaagacactataacgt

tcctgaagagtcgtgtatcagtgcttgagcgttgcaagtgtagtggtaaatcggagaaacaaaagaattt

cgtcgataaacccggtcgttctggatacagggcaaatacggctatcactgagcaaaggtgtgcggtttgt

aaggctgcacatgaaacggaaaagtgccccgaactaagcaaattgaatgtgaaacaacgaggagcgttgc

tcaagaaaaaagggttgtgttttgtttgcttgaagtatggtcattggaaaaatcggtgcaagtcacagat

caagtgtgaaaggtgttcgggttttcatcacacaatcatgcattacgatcaagcagagagtgtagtgaat

caaacagcatccgatgaagtgccagattccgatgtggcagtgtttagtgtgagccaacactcaagggttt

caacgttgccgagagtgcgtgtaatcttacaaacagctctagtgaacgtaatagccaatggcggaaggtt

agtgcgttgccgagcattgttggatagtggctctcagataagctgcataacacggcagctcgtgaataaa

ttggggtgtcgaatggtgaatactaacatgccagtgtcagggatcgggaacgttaagtcaccagtaaaca

agtgttgttcggtgattgtaaaatcaatgtgtagaaactttactgttgacttgcgttgtcttgtgtatga

tgaaataacgggaacccttccagcgtcttatttcagtattgataatctgagaataccaagtgatcttgac

ttagctgatcctgactttaacaagccggcgtcaatagatttgcttattggcatggatcgtttacctttca

tcatgaggtcagggttcgtcaaactctcgaatgaactaccagttgtgatggacactgaattaggatgggt

gattggtggctgtatcgacagcagtgacattggtgaaggtgttcatacgaacatagcagtttctgataat

ttagatcagttattgcgaaggttttgggaaatagaagagatttctaacgaacagtgcgttcaaacggaag

cgcaacaatgtgaagaacattttaagaaaagtcatcatcgcgatgtggacggtaggtatatcgttcggct

tccattaagggaaacagttgacaaacttggcaactcacgttcgatggctttgcgtagattttttgctttg

gaagctcagcttagtcgccgtccagaagtgaaggagcagtattccatgtttatggaggaatacaaggcta

tgggccattgtcgcgaggtggatgagtcgctcgatttagctgaagttaaaaggtggtaccttcctcacca

tgcagttttgaaccccagcaagaataccaccaaatgccgggttgtatttgatgcatcggctaaagtgaat

gatatttcattaaatgatgttatgatgacgggtcccactgtgcaggctgatttattctcagttcagctga

ggtttcggatgcacagatacgtgctgagtgctgatgttgccaaaatgtttaggcaaatcaaggttgaccc

gagggatacgcccttacaaagaatattttggcgagctgagtcatcaagtcctatccgtgtttttgaatta

acaactgtaacttacgggactgctgcagcccctttcttggcaacaagaaccttgctacagttggctgacg

atgaacagctacggtttccgttggctagtcgtgtagtaaaggaaaacttctacgtggacgatggactttt

cggtgcgaaccatgtggaagagttgttagaggtacagcgtcagattgttcaattgttcgattcagctggg

atgcatttgcacaagtgggcgtctaattctgaagtgttgttgagcgaagtgccgagagaagatcgagatt

catttacggtgttgggcgagagcaaggcaaatgaaataataaaaactttgggtatcaagtggaatcctgc

tacggatgaatttattttcatcactccacagatatcggaaaactcggccccaacgaaaagagaagtgctt

tcggcattggccaaaatttttgatcctcttggtctcatttcaccagtgattgtgattgctaaaatgttaa

tgcaagagttgtggaaggcacagcttggctgggatgacaaggtggatgattctcttttgcatggatggac

gaaatttcttgctgcgttaccaagagagaatcaaatacgtattccgcgtcaaatggtgaacgatgactca

ggcttcttcgagttgcatgggtttgcagacgcttcggacaaagcatacggtgcgtgcgtgtacattcggt

cggttaacggacatggtagtgctgaggtacgtttagtgacaagcaagtctaaggttgctcctctgaaacc

tacttcgataccacgaaaggaactgatggctgcggtgcttctgtgccgattgacgatcaaggttgtggat

gctttgtttagaacaacgtttagaagcatcaacttatggtccgacagtcaggtggtgcttgcctggttga

aaaaacctttagatcgtttggaagtgtttgtacgcaaccgagtagcagagatcacgtctcatcgcgagtt

tacatggcggtacattaacacagcaatcaacccagcggacatcgtgtcaagaggacagttggcttctgaa

cttgccgacaacgagctttggtggaacgggccagcattcttgaaagaatgtacggtagaagctgagccga

caacaccaggcgtctgaaggagaatcgtatcaaggactctaccttgcgaattatagaactacgagattcg

tcaagacggattgtaggattagttcagcagatggaactatcgagcatcataacggcagtcaaaaacggta

gccgcaatcatcgttttgcgaatttgaacctgtttctaaaagatggtttgctaagagtgggtggacgtct

gcaacattctcagctaccatacgaaagcaagcatcaattgttgctgcctaatacgagtgtgattacgagc

agaatcatcactatgatacatcgtgagcatctgcatgtggggtgctcgggcgttatcaacattctacgac

aacaatactggttgacgaatgcgcggtccacggtgcgcaaggttcttcgaggatgtgtaacctgttttag

agtcaatccaacgactgtaagccaacagatgggggaccttccttcgtatcgtgtgaatgcagctccgacg

tttgaacgtgttggtctggactacgcaggaccaattttgcttcgatcaggaatacggcgggtttcctcat

cgaagggttacatttgcatatttgtgtgcatggtaaccaagggaatacatctggaggctgtcgaaaatct

aacgacggaagcgtttctggcggcttttaaaaggttcgtgtcacgtaggggaataccacaggaggtattt

agtgacaacggcacgaactttgtgggtgcgaatgcggaaattcgagagatgcgaaaggcctataagaaag

ctgttgcagctcagggatttgttgaattttgtcaagcaaaggagatcagttggaaacacatccctcctaa

cgctccacatttcggtggaatttgggagagtggagttaaaagcgtaaaggcggtactcaaaaaggtgtac

aagacagcgtcgttagcaatctttgaattctataccttactatgtcagattgaagcgattctgaattcta

gaccattgtacgcgcattcaagtgatccacatgatcttgaatgtttgacaccagcgcactttatgatcaa

tcgacccttggtaggagttgcggagcgctcgtatatagacgttgcggaaaatagactaaacagatggcag

cgaattcagcagttgagggatcaattttggacaagatggtcaaaggaatatttaaatgaattacaagtgc

gaagtaaatggatcacaaaacaggctaatgttaaaccagatatattagtgctgataaaagaggataatat

gccaccacagttttggaagatgggtcgaattgttaagacatatacagggcctgacggtttagtaagggta

gcagaggtgttcacgaagggtggaacatacaaacggccaatacataagttagcgcctatcccaggattag

aagagtagttagacacttcttgtgtgggagta

>bel-24_ag-ltr bel anopheles gambiae

tgttccgttgataacggaagatgaagaggagagagagagagggaggcatcgtgccacacggaacgacatt

tgtagttgacggtaggcacgtacgtatgtatcgatataataagtcgagcgaaacgccattcggtgtattg

gtgtacgtgtagtcttgtttggtacgtgtagcgtagcaggtacgtgtacgcgtagcaggtagaaacgcgt

atgcgatgaatttatactggtagtggtaagagcgagcggtgaatttcgaattgaaacggcattctgtttg

tagcaaccggcttagccagacgcaaacgcagcggtgttaagttagtcaataaagcgacacaaaaaaccaa

acttttctttcgtatccactgcttcccagagcaaactatcca

>bel-25_ag-i bel anopheles gambiae

ttccaaaaaattacttgtttaacagtttacaagctaattcgtcgcgagtcgtataaccgaatcgataaag

tatcgcgtaagtgagttacacccgcgtccgcgtagtgaaaacaataaaacttagtgggaagtttacgaag

gttgagaaccgaacaatggatcaagcacggaatttgcattacgatgtacaggaggcttattccactcctg

tgacgagtcatgcgacagggcacaacggcgttcaaactccaagagcgtcgtcaagctcggttccggaacg

cgatgctgtgaaaaatattccgctggaagcaggcacaagccggtgcgctacgggcggtaacgcaataccg

caaaggcaggagcgggacgagacagcgttggatgcaagatgggtttgatgagctgggaatgtacgaccga

gagggtaagcgcacactatgccacaggacgctcttcgtatgcgccacacaccactgcacacacttacaca

caacgaatcaccagcgaacacatttcttaaccaaagccaagtaacagctcgaaacggtttcccacgggat

ctgccgagtttctacggatccgtagatgagtggccacgattcatatcgacatataatcgaaccactattt

cctgcaactacactgatggcgaaacccttgagaggctgcagcactgcctcaaaggtactgcgttggatat

agtcggtcacttgttactgtttccggaaggtgtgaaagcggctattaacactttgaaagctcgtttcgga

aggccggatttgattgtggatcggacggtggagaaaataaaaatgatggcagcacccaaaatggaacgac

tagataccgttgttgagttcggttttgcggtgaaaaggttgatagcgacggtgcaggcttctggattggt

ggattacatccacgacgttatgctgctgaaggagctggtaaaaaagttaccacctgtactgtgtatggaa

tgggcacgactccgaaagcgattgcgatcagtgaacttgtaccagttcggaatgtggatcggcgagcttg

cggatgatctgtgcgcggttatcgacatgcccgaagccagcacggagcaagctgagagaagcgcaccacc

cgtgcgtcataatgtgcaacggcacgcgccacatcgacccacggcaatgcagcctcaccgtaccgttcca

tatgtgcgtactactcctgcttactgtaacgccgtggtactccaggaggagtccagcagcggtgtacctt

catcgagcagccacccggagtcatcgtcgtgtatcctctgtggggggacgtgcgtgtcgttgagcgcctg

tgagaagtttctcaggactacggtagctgccaggagggcgttcatcaacgaacgcaaactgtgtcgcaga

tgtctcgaataccacaacggaagatgcaacatcacaactccttgtggcgtagatgggtgcagtatcttgc

ataattcactgctgcaccaggaaggcacccatggtaactccggtatgtctaatgaaacaaataataatta

ttttaatacacattcaggagtgaatgagagcgtgctgttgaagtacgtaccagtgacgctgtacgggcca

cgaggaaaggtggaaacattcgccttcatcgacgatggctcaacgtcaacgtttatggaccatgggttga

tggacgaacttggtcttgaaggcacgccccaccctgtccagcttcattggaccggtgacgttacgagaga

ggaaagggactcggtacaactctcggttaaaatatcgggtagacagggttcggccacgccgtatctgcta

accgatgttcataccgtgaaggaattggctctcccgagacaatcggtcaacacttcccagcttgcggcaa

aatatcagcacctacggggacttcagctgccgtcgtatgcggatgcagcaccacgcatcctcatagggat

agacaattgcatccttgggcgttcgttgaaatgcgttgaagggaaatgggggcagccgatcgtctcaaaa

actcgcctaggatgggtagcgtatgggccatgtccgatagcgaccaatacaccaggagtttccttgcaag

ctttccacacacgggtacgcgagagatccgtcgatctcgtggctgttgagttgaatgcacgatcttcgcc

gcgaatcattgacgtcaaaagtgcagagcgtagaaaggtcgaaattagggaaccaattcgagtgacagcg

gtaccgcgtatagaaatgcgtttcatagaaaacacggtatttggtacttcgccgcgttgtacatatctga

ggaaaggtaggattgttgtcttagacggttcaccaggatgtagtagaaaacactatactcacttgccaat

agaaacggccaaagaagacggtaaacaggataaacaccggagcgccgataacaatgcggatttgatgaat

gtggtgcggttgattgaagatagttacacgcatcgaaatcttactcgaataggcagaatagattcggaaa

ggtcgaagcgaattacgaaaacaccagctgaaaagaattagcgagataattaactgaaccgcgtatgaac

ttacctttagaagcgtcggccaggtagaaccgcgtgtatgcacatttcattgtagcgcggaagtaaacaa

agtaacgaaacaagagtctgacagatatgcgagagtgcgacagcgatagcttcacactcggggggacaa

>bel-25_ag-ltr bel anopheles gambiae

tgttggaaacatttgtaaggttccaacatctgtcagaccaggcgatgttttgatccggtttcaaaacata

taaaaagggagaaaaaccgcgaaatcagcccttaacgacaaaagcgagcaagtgtacaaacgattataag

agagagaataaaaaacactaattttttggcactgcgttgaagtagcctaaagaatttaattctctcactt

ttttacacgggaaacaaacgttttccaaca

>bel-26_ag-i bel anopheles gambiae

ttatggtccttcgtagaaccggatagaattggctatcgagataacgacaattcgttgcacacgggtgcat

tagtggcagtgtattgtgcgtttgctgtttcgatactcgtagagagattgaggaagtgtgcataacggca

gtgtattgttcgcgcgagtgtcacagtgtgagttggacactaggacattcagtgtgcgtgtaaaatagct

gcgcgcgttcggacaggctcatacttgagcatacgcatgcgttagatcgtgcacttcacaagtggcttaa

ttgtgaagtccagatacgcaacatttggtgcatcgagcgttcaaagtgtggaacgttttcggtgagaagt

gttgtgtagcttcagtgcgggagtgcaagtgtccccacaaaccgagtacctgacttccaattcgtggaac

gtcgttggtggtatcgtttgttccagtgtaggtgcgcaagttacctacacacgcacatcgagcgttttca

atctgtggaaacgtggtcggtgcgatacattattgtgtgtcgtcgaaccgctgccgttcgaattcacaag

aaattaccagtgcaggtttgcaagttcctgtacaaagcatatcgaacgttttcaatacgtggaaacgtgg

tcggtgccagttattgtttctgacggtcaagcctcagtcttttggacgcttgttgaacaatacattgttc

aagtgttagtgaacattgaattcaaagttgtatgtttcaagtgtaggtgagcaagttccctatacacgta

catcgaacgttttcaatctgtggaaacgcggtcggtacggtatctttttgcttgtgatcgatcatcagtc

gttcgaatacgcaagaagtgttttcagtgcaaggaggcaagttccctgcttaaggcatatcgaacgtttt

aattcgtggaaaacgtggtcggtgctaacaaacgtattgtgaacaatacggttcgcgcgcgttattcaaa

cggagtgagcgtgtgcgcgagtgaacaattctctcttttcgggttaagataaaggttcgcgcgcgttatt

caaacggagtgagcgtgtgcgcgagtgaacaattctctcttctcgggtaaagcaaagttccgcgcgtgtt

aatccgagtgtgtgcgtgtgtgagtgtatgcgaagaatacgatccgcgcgtgttaatccgggtgtgtgcg

tgagtgtgcgaacaataaaaccgcgtggtttgaactaattctacgggtagtgtacgggacaaattgaact

attttacgaacattcagtgttgtgaaaagtgttttttgttacaagtgaaaatgcctggttcaccagttcc

aacgacgtcggcggcgtttgaggaacaagacgcatcgcatcgtgtatcacatgtgcaagctgcatcagac

agcgatcatgtccaagccaatccaatactgagggcagccgtggcaagagaagcttcggagagtgagtttg

aaggttttgaatcagtaaattcaagtcctacttttccaacagcagcaatgctggcggagaaaagaaagcg

aatgcgtcagcatttcgaccaaaggctggaccgcattgatcaagcactacagcatgcaacaagagcggat

gccgcttttctgaaaggatgtgcgtcacgcttgtcgatgctatccacggaatatgaaacatggcacacca

acaatttggtttcttcttccaatgcacagttcgaacaagaagaagaagagtacgcctctttcgagaaccg

ccattttgagctttcattggcaattgaaagaaacttgtcatcaagtgtagtttccacaccaacacgcgat

accatgccacacacaaaacttcccgaaatgcgcttgccaacttttgatggaaagatggaagattggttac

cgtttcgagatgcctttctcagtcttattgatcgcaataagaatttagccgacgtagacaagctacgata

cttaaaaggctctcttcaaaaggatgctcttaacgtagtaggagacatcgaaatcaccgacgcaaattat

tctgttgcctgggaagtattgaaatcgcgattcgaaaacaaaaagctagtggtgaaacgatatcttgatg

gattatttgcgataccacacatgaaaaaggaatcatacgaatcactgatttctttaattgacagttttga

gcgtggtgttaaaatgacgactagaatgggagtggccacggaaggatggagcgtgctcttagctcacatg

gtctgctctaaattagacctggccacacttaaacaatgggaaatacatcatagctcaaccaatgttccca

cgtatgacgagatcatgaaattccttcgcagccaagcaactgttttgcaagccatggctccagacaggaa

tcgaacaagtgaagcaacaacagctggaggcagaatgaaaacccgcaacgaagtgtacagcgtggttaag

ccggccacaaaggtttgttcattctgcaaaagggcttcacatgctgcgtacctgtgcgaagtgtttcgaa

atgcatctgtggaaaagcgacacgagctagtgaggaacggacagttgtgttttaactgtttgtcggcggg

tcatcagcgaaaggattgttcgtccggatcttgccgtgtatgtcagcgaaatcatcatacgatgcttcac

aaaccgaattctccaattgatcagtcgtcattagcatcagcgtcatcactgccgtctacatcaaatgcag

aagcagctacaccggcttctattgttcaacattgtgcgcgcaacaatttcgaaaaaataatcctgttacc

aacagcaatggtacaagtagtgaatgttgacggaacacgtatctgggctcgagctttactggatggaggt

tcacagatcaacattgttacggaacgattggtacagctgttgagagtccagaagaggaacgagcatcatc

tgcttggtggaatagggaaggcacagcattcgtctcatcattctgtgagccttgctatccactctcactg

ctctaccttcaaggcaagctggaagttccatgtgctgcgagaagtgacgtgggaccagccagcacatgca

gtcaatccggaaggattgaacctaccgaagggagtgacgttggcggatcctcatttctatgagccagggc

caattgatctacttattgggcgagaaggttacaacgatctacttttgggtaacattcttcggttgaatag

cccaaaactactactgcaaaacacggagctgggttggattgtctcaggtcaggtaagccaaggtctaaca

cactcttccttagtgctcaacgtcatgacgctgaatgaccaactaagccggttttgggaactggaaggct

gctactcccctgcgatgttatccgtcgaggaagctgagtgtgaggcagcatttgtacaaacaacttcacg

agaccacgaaggccgtttcgtggtagccttaccaacgcgtactgaaaggcttaatcaactaggtgactct

tatgaagctgcatcacggcggctacaatcgctctgtaggcgattaaatatcgactcgaaattgaagcagg

aatattctaaattcctacaggagtatctcgatctaggacacatggaagaaattccatctgaccgccatga

cattggaaagacatattacatgccgcatcattgtgtagtacgacccgacagcctcacgactaagctaaga

gtcgtatttgatgcatccagttctactgatacaggggtatccctcaacgatgcactgatggtgggacctt

cagtgcaagatgatcttttgtcgttgttactccgatttagagtacccagatttgcgattttggctgacat

tgaaaaaatgtatcgccaaatttgggtgaaggaatccgatcgtccacttcaacgcatcctgtggaaagaa

tctgctgatgatagaattcgcatctatcagctgaaaaccgttacgtacggcacagcctgcgcaccttact

tggccacccgttgtctgcaagcgttggctaaggaaggagaaacaagatatcccagggcatccaaggtcct

cgagcaagatttttacatggatgacatgatcaccggtgtggaatctgttgaagatggccggattatttgc

agcgaaataaatcagcttctacaatcggcaggatttcacctgcgcaaatgggcttcaaactcgtctgagt

tactggagcaaataccagctgaactacaaatcgagggagatgttttgaacatcgatcgcagcacatccat

caaggctttgggtttaagatggatgccatcatcggaccagctagggtttagtgtgcctggttggaaggaa

tcagaaatcatcaccaaacgcattgcgttatcagacgcagctaagttgttcgatcctcttggactgttag

gaccagtcattgtagtagcaaagtgtttcatgcaggagctctggaagaacgaaaatacctgggatgaacc

tttggagtctgaacggcaacaattctggttgcgcttcagagaacaactcgctgatctgactagcttgaca

attccacgacgagcaattggtggaacagtgcgaattgaagctcacggatttagtgatgcttccctacggg

cttatggcgcctgtatctacatacgagcagaatcgtccgatggccaagtctcagtacggttgttatgtgc

taagtcaaaggtggcaccgatacctaataccaaacggaagaaaaacgtgtcccttccacggctcgagttg

tcgggagcgttgttactatcacacttatggcagaaggtgaagcaaggggtgaaattggagcttaaaatca

acttctgggttgattcgacgatagttcttcattggctttcaagcagtccttcccgttggaaacagttcgt

tgcgaaccgagttgcggaaatccagcaccaaacctacaagatgccttggagttatgtggccagcgagcac

aatccagctgacattatttccaggggaatgatgcctttgcagctcatggattcccaactttggtggcaag

ggccctcatggttgcatcttccaagtcaagcttggcctacgaacaaacccaatactgaaatttcctcgga

agatttggaagagcgatcgattgtagccacatctcaacaggtaccgccaaattttctattcgatctttct

tcatcttacgaaaaattggtgcgacttacggcatatctgctgcgattcatgtacaattgtcaaccgacac

accgcggaaatctacgaaaggggtttctaaaaatagatgaattagtgtcagcgtctctaacgctcgttcg

tctggcccaacaagaaacctttgctgaggagcttcgggatgttcgtcaaactggagcggtcaagccgaac

tcaaaactgaaaacgctaacacctattctgaaagagggtatcctacgtgtgggtggtcgtttgcgaaacg

cgcctgtttcgtatgaacgcaaacatccgataattttagcgttttctcacccattgaccctactgattgc

gcgttcttatcatcgacaatatctgcatgcgggacaacaagagctcatatctagcctgcgcgagagattc

tggccccttcgcgtacgcaacctggcacgaaaggtcgtatatgagtgtgtaagttgtttccgatccaaac

caacaacggcagagcaaatcatgggagatttgcccagcgaacgcgtaaacccagttctcccattctacaa

caccggtgtggatctgtgtggtccattattctatagacaaaccaacaagaaggctgctccaatcaaatgc

tatgttgctgttttcgtctgtcttgtgatcaaggcagtacatgtggagcttattgccgatttgtctactc

cagccttcatttctacattgaagcgtttcatagctcgtcgcggtaaaccatccgtaatccagtgcgacaa

cgccaaaaatttccggggagctgatcgagcgctcaaggagatgtatgagctgttccagaaacaacaacat

caggatgctgttacaacttactgcggaacggaagggatcaccttcaacttcatccctccgcggtcacccc

atttcggtgggatctgggaggccgcagtgaagtcgctgaaacggcatctcaaggctacgataggatccag

tatcttgcggcgagacgacctggaaactatcttagttcaagtggaggcttgtttgaactcgcgaccgtta

actgcactttccaacgatccagaggacctggaaattctgacaccgggtcattttttgattcaacgggcgc

ttacatcggtccctgagccatcttatgctgagattccaggcaaccgcctggaccgctatcaacaattgca

ggagtatgttcggcggatttggaagcgatggaatcgagactacttgtctggtttgcatccgcgtacccgg

tggacttcaaggcgagacaacgttcgggagggcactatggtcctgctgaaggaggacaaccttccccctc

tcaaatggcgcttcggccgagtgctgaagatttatcctggtgatgacggcttggttcgagtggttgatgt

gaaaacgaaggatggaatttacagaagagccatcaccaagatctgtatactgcccggacagaaggaagaa

agagaggtttcgtaataagggttgaaagctacctttcaacggggggcggcta

>bel-26_ag-ltr bel anopheles gambiae

tgttgagtaatgaatttgtacggtataaacgtatgaccttctgtcacttgaatttgttgcttgggaattg

tattgaggggacgaatgaaggaaacaaggaataaagaaagagtctagcgcaagcgttcaatcagtacaga

cgttcctctctctaccagttgagaaattcactaactaaaaca

>bel1-i_ag bel anopheles gambiae str. pest

tggtggctccagagaggactagtagagacctcgcaacgtacaagtcggaggttaacttccgatcttgaaa

cccagagtcggcaggaacatacaagtcggaggttagcttccgatcttgacgaacgtattcctgaccgata

gagaaaaacaaccagcgctccaacggaggaaggttcgtaaaagcggaaaacatcgcgaatgtataatagt

atccaaaaacgtaaagtgcaacaaaccaaacgagtgcaaaaaacgcctgaaacagtgcttaagaagtcca

aggtacagagacagaattcagtaaggcgccgtacgatcaccgatatattgtgattgaataaagacaccgg

tggcaaaaaaaaaaaacgacgttcgctatatcgtttcggtcgtaccacaggtttgtaaacaaaacaaaaa

gggtgtgtgtgtgcagtgaaaaaaaaaaaaaaaaaaaaaaaacgacgttcgctatatcgtttcggtcgta

ccacaggtttgtaaacaaaacaaaaagggtgtgtgtgtgcagtgaaacgcaacctcagaaagggcgttta

aaacggacgacctgagaacatctgatcgaaaatcattacgtagcgtactagtaacaaagtgcagtgtaaa

caattacgcgtgtggcacacattcgcagtgagaacgtaataaaacccacaagacgctaacgaccgcggtt

tacggacgttcccgtaaagcaccggcagtgtactccaattacaacaacacaagtaaatgtgcggatcaag

aaaagatcaacagttggggagtgtaacaatacgggacgtgcatacaaaaaagaaatcttcgagctaaacc

gtatccggctttagcatttccgggaaaccacaaacataaaggtgttgataagagtgcgaactagtggagt

agtacgtgtccagcttattgacgtgtgttgatacggtgatcggtagccaatggtcatcgccccggaagac

ccgattatccaacgaaaaagcgtggcggcaatactacccaagcagcggatctgaaggcgaccagaagaca

ggggtactgacgacaccgcgggggatcacctgaagacgacctcgaggaggacaagcaggggcgttgtaag

agcagcaacggcacgacgacacccatccagatcggcagcgacagcccagttactttatccaccggttacg

caaagtgagtagtatggatatcatatttaaaagcaacccgaaaggcaattgcaagctatgcaagaacccc

gatgagtgggacacacaagttaattgtattgagtgtgacagatggttacatctcaaatgtctgaagctag

aaggtcccgttaaaaaatatgtgtgtccaaaatgctacacaatagctgaggaacgcaagggaaataggga

ggccttaatgcaaacagagaggctactaaaagaaaaaactgaagcggaaaaaagaactagagaagaaaac

gaaaggtgtgagaaggaaatcgaaagactagaagacatattaagaaatgaagaaatacataaccaatccg

acacaactcacctacaagacgacctacagacacttacaacaaacgtaaataaaatggcaaacttgggttt

tgctccacacaagaagacagttttaaaacttccggatttctatggcaattatagaacatggcctcgtttt

aaactactgttcgaagaaaccactcgaacagaaaaattttcgaatttggaaaaccttacacggctccaaa

ttcaccttaagggagatgcattgcgatccgttagcgggttgatgttgaacccaagtaacgtggatgcaat

cttggaaagattggggaggttatatggcaacccagttagcatttttaacgccttactaaaagaccttatg

gtggttaaacgggcatctttggaaaatccatcttcgattattgagttctgtaacgcactgaataacatgg

tggaaaatatgaccatgttgaaccaaacggagtacttgatggaccaaaggctccttacagatctggtcgc

aaaactctctccggaccttaaaaccaggtggctcagagattcacttaacgaggaaggtgacaaaatcaaa

accttgaaagatttcagcaaatggttgaaaccaacagaagacgtggcgatcacacttcttgctatggaag

gtggtcaaagagacagaccggcgaggctgaatactcactattcagccagccatcaaatttcaaataaagg

ctgtctaatttgcagccgtcctcatgaaaccatatcttgttacaagctaaagaatgcctcagtaaacgaa

agatggaaaatgctgaaggagaaaaacgtttgcactaactgctgcaaattctctaaccatgcggccatta

actgtcgctcaaggccgcagtgtacagtggatggttgtggacgacgacacaacaccatattgcatgaaga

aaaattyaactcaatgggcgcggcgtcaaaagcacatttaaattttcatcaaaactcggaacaataccta

tttcaagttctgccaataactgtctataacgaaaacaactccatcgaaacatttgcattgatagacccag

gatcctcaacgagcctcatgacagaaagcctaagacagaaactaaatctgcatggcccaaggaagccgtt

aacactctcgtggacaaatggatgcaaccaggtagaggatacaagcacgtcggtatctctgaaactcaga

ggtccaaacggcaggctgctttatgtcaaggacattaggacagtaaaagaactggacctacccactcaaa

gcatcaatgcaaacgtgttgaagagaaaattttcccacttaaagacggtaaatatttcaagctacaaaaa

tgctaaacccaccattctgttaggacttccacatgcttattacacgcaagctgtggagtccaaatcagga

gcgcccaatgaaccagtggcacacaaaacacgcattggttgggtcgtatttggaaagtgcagagatggtg

atgcaaaagaaaatcaacatcttttcacaatacaggataagaaagaggaggaagaaaagtcaatgaggga

cctgatgaaaaggtttttttcaacagaagaatttggcgtaagggaaaccaaattcaccccaaaatcaaag

gaccatgaaagagccctaagtgtaatgaatgacacactgaaatatacaaataatcagtatgagattggcc

tactttggaaagatcccaatgtatccttaccaagcagctacgcacaggcgctaagaaggctcgaaagtca

agaacggaaaatgaaaggtaatgacgagatgaaaacctggtataaaaatcaaattactgattatgttcag

aaaggttacgctcgtaaactaacaccatttgaattgctgaatagagatccaaagatcaattacattcccc

attttatggtcatcaatccaaataagccaactccaaaaccaagactggttttcgatgcagctgcaaagaa

cgaagggatttcacttaactctactctcttgtccggaccagacgccactacgtcaatttttggagtatta

atccgctttcgcgaataccctatcgcctgttcaggggacatcaaggagatgttccatcagatacggatcc

gcaaagaagatcaagtggctcaacgatttttattcagggataatccacgcaacgaaccccaagtatacgt

tatgaacgtcatgaccttcggtgccacatgctctcctgcttgtgcccagttcgttaaaaatgaaaacgcc

ttaaaatataaagacaaataccccactgcagtggaagcaatagtaaaaaaccactatgttgatgattatc

ttgatagttttcggactatcaatgatgcaatcaagactattaacgaggtttgcctcatacatgatagagc

gcatttctttatgagaaatttcgtttctaactgtcaggaggtaataagaagcatcccagatgatagatcc

tcacaacaagagctgctgcacatctctaataaagatatgaattttgagaaaattttggggcaatactggg

acaaaacaaacgatgtgttaaggtataagcttaagcataccccgtgttccataatttcaaaaagagaaat

gctagcctacttgatgaaaatatatgatccattggggctagcggcaaactatactacgcaagcgaaggtc

atcatccaagaaatttggaaaacagaactggattgggatagcccagtaccagaacgcataatggaacaat

ggcaaagatggaaggaaagaataaaggaactagaacacatacaaatacctagatgctactcggtggctag

caatatcgaagtaactgagttacacactttcgttgacgcttcggagaaagcgttcgcagcagtagtgtac

ttaagaacattaacagaaaaggggattgacgtaaacatagtggcggcaaaaacgagagtggcaccaataa

aaccactctcaattcctaagctggaacttcaagcagcagtactcggagtcagactcgccgagactgttaa

agaggaattaagaattaccactgatagggactattattggtcagattccaaaaccgtcctaggatggatc

aatgccgatccacaaaagtacaaacaatttgtggcggtaagaattggagagattttagatactaccaatg

ctagtcaatggaagtgggtctcctccgaaagtaatcctgcagacgaagcaaccaaggtagttacaagaaa

atctatatggctgaatggcccagtatttcttaaacaaagggaaattgaatatagggaccccaagctaatc

attactcatgaagaaatccgtccaaatcttatgattaaaaccatagagaagagaacattcaactttataa

aaaccgaatggtgttcaaattggctaagactgaagagatcactggcaatcaatttaaaatatatagaatt

tttgaaaagcaaggtcaagcgattagcattttccccgatagtagaaaaggaaaacctggataaagcagaa

aaactcctattgcaaaaggcacaatgggagatatacgaagatgatttagttcagctttcactcaatggac

aagtctctaaaaacagcacaataaagaatctcaatccacaagtaatagaaggactactacgagcaagggg

acgattagcaaatatatgctacctctccgatgacgtgaaacaacccataatattacctaagaggcatcac

gtgacagaattgatcatacagcattatcatgaacgctatatgcataaaaaaatggaagcagttattgcgg

caatccggcaaaggttttgggtaatcgaccttagggccgtggtaagaagcgtgatcagcaaatgccagcg

ttgcaaaaatgaacgcgcacgtcccattgccccgatgatggccccccttccagaaagccgagccgctgtg

ttcaaaaaacccttcacccatacaggtgtagattactttggacccatgacagtgtcaatcggaagaaggg

tagaaaaaagatggggagcgatattcacgtgtatgacaacgcgcgctatacatttagaaatcgctaaaga

cttaagtacaaattccttcataatgtgcctaaaaaatgtgcagcataggcgtggaaagatttgtcacata

tacagtgacaatggtacaaacttcgttggggcaaacaggcaaataacggaactcgtcgaaagatgtgcaa

ccaacggtatcaaatggcacttcaatcccccggccgcgcctcactttggaggtgtatgggagagaatggt

ccgagaggtcaagagcttgctgccaaataatgataatatgccagaagaagtattaagatcggcctttatc

gagatcgaatttattctcaataatagacctcttactcacatccccctcgaaactgaagacgacgaacctc

tcacaccgtttcacttcttgatagggtgttccggagaggccgaacctacgccagccgggatttcagcagc

tgaagctagcagaaacaactggaagaaggcacaagttatcacccaaaactattgggaacgttggttgaag

gagtacctcccaacattagccaaacgagaaaagtggatagaacgctcagacccaatacaacctgatgaca

tagtcgtcttcccagacgaacaacgcgtgggtaggtggttaaagggccgggtagtagaagtttatcccgc

taaagacgggcaagttagatccgcaaagattaaagttgaaaacggcgaatacaaacgccctgttatcaac

ttatcagtactagaagtaaagggcaagaaaattgcagacgtaccttcgtggggagttaaaagaccggtca

acatcgcctacgtcaagaaattagctgaacaattaaaaactcctcctgcaaaaaggaggaagcatctcgt

aaaaccttataatggcccggtatctatgcattataagcctgttagccgcatggaaactaacagacaatct

ttcagttaaaccagttgaagaagctggtatattcttcgaccacgaaggaacgcttctcttgaaaaggggt

gtgtgggaaacaaccttccacacgaaaatacaccccgaaaatgacacagagactttactgacaatggaga

aagaggtgaccacagtattcaaggcactgagtgacatggacactaatcttctaaatttgaaattgacatt

acaacaaaacattcgacacgcacttcaactctcacagactgcagtaaaaagacgaaccaaacgatctagc

ggcatatttggatttttgaaaggtattctatttggagaagacgatattgatgaacagttagccctcttta

gagcttctgaagaccagaaattgaaacatatatcggaagacatgactcataaaatcaagcagggtgacag

acttagaaacaaactaaatatgaaaatagaccacatgaacgaaggtattaagagtcttaataaaagcttc

aatgaaaacaaaaaaaacgtacttataaagcatgtcacagaaacaatcatgctagctgaagacatagtac

aatacattacgacaaggtatctagagttagaaatccagccccttagcatattcgactcgacgaaaatttc

cgagaagatacaatcaaggttacccgatgggtatacaattctagaccacccccgaatttctagcaaagag

ttatttaggggagaaataatagtacatattgaaaacgtcatcgtttcgcaagagagattcgaaatattcc

atatcactgtaataccaaacctaaaaaactttacaactctggacttagatgaaaatgtaatagctataaa

cgatatacactatatataccctacagatattacgcgatacaatagcactcatcacgtgtcctccgatgta

gccgttagaagagatttggattgtatatcctcatctttcagacatataaaaacagaatgcttgtgtggta

taaaaccaattaaaaactcaataaccaaatttgtaaagctctcccagccgaacaaaatcctatactactc

ttctcatcctaacgaaatatacctcaaatgtaacaaaacactgacacatcccgcgtatcaagctggggta

ataacacttagccaagactgtaaaatacagaccaagaacatagaaatccagccaaccatgaaaattgaag

ctgtagaaacaaaaatgtatttcaagccgctggccaaaatactgaatttaagcgcagagcaaaaagaaga

aacaaatatggatcagctctacctcataataattacaagtacaatagcctgcgtcgccactttaatatta

ggaataaccatagcatttattatcaaacaagtacgagctaaaatgtacactttacgccccccgccgttta

aaccgtcaccaaatagtaacccctctacgaggaattacgggggtcagga

>bel1-ltr_ag bel anopheles gambiae str. pest

tgttgatgacagcgccgcataattaggtcatcacggcctgggaactgaccgaccgaacgaaaacaattgt

aagcaaatctaatgtaaacaatatcgaccaaataaaaactgctgatcgcgatcgatcagcagaaaattag

tatataagtcaggaataagcatgaataaatcgactttcaagcacaagaactcaaagactaagttgtttgt

ctgttccaagccaagtcggacggtaaaatcctcctgtcttggcgcttcccaacgagttcagactcggtgc

ggaaaatata

>bel10-i_ag bel anopheles gambiae str. pest

ttggtggctccagagaggagagcctcaccgtagatagtgtcatcgcgtcgttgttagaccggagtggacg

agccgtgtagctaccgtgctccgtgttaccaagtgcgccgtggtcccgagtgtccagccgcaccggaagg

gtcgagcccagagtgtgcttctggaaccaaagcgtccagcccatccgtgacgtcgtcacatcatcatcat

cagaccggattggaagaaagctaagtccgtgcttctggccagagtgtccagccatccgcgtcattgaagt

gcgccgtggtcccgagtgtccagccgcatcaatttatgttatttgtcatacgtccttctgtcattggacc

ggattggaaaaagagctaagtccgtgcttctggccagagtgtccagccatccgcgtcgttaaagtgcgcc

gtggtcccgagtgtccagccgcatcaatttatgctgttttgtcatacgaccgtgtgtcgtagaatcgtcg

tgaaaatggctaccaagaagagcaattattttgttgagaacaaaaacggtgcatgtcgcctctgtaccga

accaaacggtgatagtccgtttgttagatgtgaggaatgcgatcgatattttcacctggcctgcgccaaa

ctatcggcggtaccaaccgcagaagaagaatggttgtgcataaaatgccaagatatcaagatacaatatc

aacaagaagaaaagaaaagtacaccagatcaagccatattagaactcgctatggtattacagaacaatag

tttggaagctagccgccatatcaagaaaaccacattagtaaaccttccagaatttgatgggaaaccacaa

gactggccgcatttcaaaaagacatttgaagacacaacaatagaagcaggttttagcaagctagaaaact

tgaatcgattgcaacgatttgttaagggagaagccgaaaaagcagtacgtgccttactcttggatccgca

gaacgttccggccatcatgggtcgtttagaagaacaatttggaagagcggaccaagtgtacaaagagctg

ttgaaagatgtggtgaaaataaaggtcgaaggacaaatgaaaataatggagctttctgacgctcttgata

accttgttacaaatattaagattttaggaaaaagatcttacttgaatgatcctcgactcatcgatgaatt

gttggcaaaactttcgatcgataaacaactgaattgggcacagcacaaagccagtctagaggcagcaaat

tctgagataacgcttgaccagttcaacgattggatgcgccaaatatcaaaggcattgcgaagtctgccaa

aaagaacagatcgaccacagaacaaagtaaatgttcacaaatatcacaatcacaaaccaccacgaaattc

ccagcaaatgaacattgctcaaaacttgcacacagtttgtcgcttgtgcaaaggacaacatacgttgcca

gagtgtgaaatctttttacagaaaaacattagtgaccgaattgcattcaccacacaacaccatctgtgtt

tcacatgtctcctctccagcgagcatgtccaaagaaactgcccattgtccaaagtatgtagtgtgcaggg

atgtattcggcggcatcatcctctgttacatgaagtgccccagccaatatattatcactataacgacgta

cgtgtctactaccagatcgttccgataaccgtacgcaatgggtcagtggctatgaaaacgtttgctttct

tggacgctggatcatcactaacactcatggaggaagatttggcaaacaagttaggtttacaaggccggca

agatccattaacaatgacgtggacccaaaatctaacggtagaacaagacaccagtcgtcgagtgcaactt

acgattgtaaatgatcaaggaaaagaattcctattgaaagaggtgcgaaccgttaaaaatctacaactac

cacaacaaacaatagacacaaatagattgcttgctctttacccacacctgaaaggcattaaaataaaatc

gtttacaaacgcttaccctaccatcctcataggcttaagtcacagccaccttatcatgccattagaccgc

agaatgggccgtccagaagaaccgatggccatcaaaacaaaactcggatggataatattcggaaatgagt

acacacaaccggcaacaagatcggatcatttcatggtacacaaaaacgaggaaataatgaatagaatgat

ccaacaatacttcagtacggaagattttggagtgaaagtgacaaaaccccttgtgcccaaagagatagag

caagcgaataaaattctagaaaagacgctagagaaaaaggatggatattatcaagtgggactgttgtgga

aaccagaagtggcacacttcccaaatagctatccaaacgcactgaaacgcctagtaagtttggaaaagca

gttgaataagaatagagagctgcaagtctgggcaaagaacacatttgcagattacatacagaagggttat

ctgagaaagttgacgccaagccaggttgctattgcaacaccaaaaactttctatctaccgcatttcgttg

tagtcaaccgaaacaaaccgataccgaaaccacgattggtgtttgacgcggctgctgaagtaaatcacat

ttcgctgaactcccaattactatctggccctgacgaaatggcttcacttttcggtgtcttgctacgtttt

cgagaaggcaacatctgcgtcacaggggacatccaagagatgttccaccgagtaagaatacgggaagaag

atcaggactctcagcgtattctgtggcgagattgtgaaaaccggtgtcccgatgtgtacgtcatgcaagt

catgacatttggcgcgacttgttcgcctgcatgcgcacaagtagtaaagaataccaacgcagctgcccac

gccgaaacacaccctctggcagtagacccaatagttcgacaacattatgttgacgactatctcgatagtt

ttttcacaatggaagatgcaattgaaacagtaagacaagtaattgaggtacacaaagctgctgattttca

cataagaaattttacctcaaaccggggggagttactgaaaacaattcctcaagatcgtgtacaacaaaaa

acaacttcagtacaaatcgaagaaaagggccaagactacgagaagatacttggtgttcattggaacccaa

cgatcgattattttgggtttcaagtcaaaatgaacaaagtaacaaaagacaggccaacaatgagagaagt

tcttagttttgtcatgagtgtttacgatccgctgggcttaataagccacgtaacaatagctggcagaatt

ttaatgagagaacttcatgttataacgaaggattgggactccaccattccagatgaattacaagagaaat

gggaagaatgtctacgcgtagtaaagagtgccgaaaaaatacgaattccgaggcaattggtaataacctt

gaatgatccgctagaattgcatactttcgtagacgcttctgagaacgcatttgctgcttgcgtttacgcc

cgcactgtaactaaggaaggaacagtttttattaatcttgtggccggaaaggcacgcgtggcgccaatta

atccattgtccattcctaggctggagttacaggctgcggttctgggtgtacgtctaaccgaaagtataag

aaaagaacttcgcctattcgtaaaacacataacatactggagtgattcggaagtagtattgagttggctc

aaaaatcgtcgtaaatacgcacaatttgttgcacatcgtgtgggcgaaattttagaatcatctcgtgccg

accaatggagatgggttccatctcgagaaaatccagcagacattgcaacaaaaccatatccaaatgattg

gatttgggtaaatggaccttcgttcttaagaaacaacgaatcagattggccacacaaagagatcgtagaa

acgaatgaagaatgccgcgtagtagctgtacaccaacacgcagaaacagctataccagtcgagaattatt

cttcgtggcctagattgttaaaacatctaacaattctgaaaaagtttgccgattttattagaaatcgttc

agcatttacacgttccattcagcctaatgacgtccaattagtccgaaatggaatgtatcgcagtgctcaa

tgggaaggatttcccgaagaaatggcgactctcaccaaagggcaaccagtaccaaagcaaagccctttaa

acaagctatcaccatttttagactccgatggcatcatgcgttcccgtggaagattggaaaacatcagcac

gcttccccaaagtacacgcacgccaatcatactgccacagaaaccaagattggtaaaattgttagtgaga

aatttccatgaacgttatttgcatcaggccgacaacgtagttatcggcgcaattcgacaagaatattgga

tagtgaatttacgagcagtgctaaagaacataaagaagtgttgccaaaagtgcattcttaaaaccgctgc

accagtagccccctttatggcacctctcccagaattcagagctcacccatacacaccaccgttttataat

acaggggtcgactattttggacctatcgaagtacaagtaaaaaggtcgttggaaaaacgatggggtgcta

tcttctcgtgcatgaataccagagcagttcatatagaactagccgagaagctagacacagacagttttat

ggtatgcctgaagaatttccaaaatcgccgaggaaaaatatgcaacatgtatagcgataacggaaaaaac

tttgttggtgcggagcgcgaattaagagaattagtaacagaaatcgacaagcgcatgggacatgaatcgg

cgctcaagtacgaaataaaatggcacttcaatcccccttccgccccccattttggtggagtatgggagcg

gcaaatacaaaacatcaaaaaaggattgcggcacatgttttccgaatggagtcaccgacacccgactcca

gagacacttcgtgccactttaattgaaatcgaggcaatgcttaattcccggcccttaacacacataccgc

tggaaaatgaagaagacgaaatactcacaccatttcattttcttattggacgaaatggcagccatatacc

accagtagtgaacgaaacctcggcagcaaacaggcaacagtacaaactcacacagcattactcaaagata

ttctgggaccgttggaaaaaagaatatcttccaacactaatacgccgaaataagtggactaaccacgtag

aacccataaaggtgggagacatagtcgtgctgttcgatgacaacgcacctccaggaaagtggatcaaagg

aagaattgtgaaagcgaatatggccccggatgggcaagtacgatcagtggaagtaaaagtgggagaaaac

atattaaaacggccagctgtccgagttgccgtattaaatgtagagcaaaaggaacatttgcttctccaca

caacatctcagcaacaccagggcacaaatcgaataagaccgcttcagcctaacaacgacaacaatgaaac

gaagcctcctcgtaagaaaacaaagatcgccccatgtcattgggcaaaacagttattggaaaataaccca

ccaagcacttcgaccaactaagcaagaagcgtaatccccgcctgtgaattacgtgggggagaa

>bel10-ltr_ag bel anopheles gambiae str. pest

tgttgcgtgcgaagtgcatgcagaataaaaaataaaaagtggaaagacacaacatcggtgaatttccacg

aaacgccagccgtaccccccacgatgaacgtcgctaccagcacgcgcagccccggtaccgtctacacaaa

ggattgctcgatggattgatcgatgcggaatttctggaatcgccagtgcaaacagatgtcgccgggagtg

acaatggaaaccgggagcgcgatgggagttggggacaaacgggaatgggagtgggaggaaaggcgtagaa

ttttgggttagtatcgtgcagaaaaattgtgaaaattttggtatataaaggcggctcaagctggagccaa

atcagattcgaacgataagccaaagtgttaagagcttcatttcagaacatccgaaataatccgaatctca

aggaacgatccccctttctgttgcatagactcccgaggcagcgaggagcactagctgaaaggcttggaga

attccccgttgtagccgtaggagccggaaccttcgcccggcggagcccctcggccgcaaca

>bel11-i_ag bel anopheles gambiae str. pest

ttttggtgcgtagtgaccaggattttgataaatccttcggtttttgtgaaaaccggcgtacagaagcgtt

tgttcataaagcagtgctgcggcactcgatctgtattagtgtttgtgtgtgtgagccttttgcaaaatgt

cgaagcaggacaagttgcggtacaaggagctcaagcggcaccaatacattgactccatcaatcgtgtgaa

agagttcttaaaaacgttcacaagtgaacaacaaaatcaagtgtcgacgcgactcgatcgtttggaaaag

atttgggaatcttttgaaacagtgcaagaagacatcgaagatttggaaatctccgaggaaggcgttgcaa

ctaatgcgcgtattagagcagaaatggaggaaacgtatttgtacgcaaaggcgcaattgcgtagtatgtt

gcccattccggcagccgctgaagttgtgtctgttgctgcaaatgctgcttcatcttcttcttcaagagtg

aagctcccattgatcgcactgccagagttcgcaggaaatttcgatgcgtggttaacgttccacgacacat

acgtctcactcatacactcatcgacggacataacggcaatcgaaaaattccactaccttcgagcttcact

caaagaagaagctgcgaatttaatacaatccatttcggttacgagtgagaattatgatttggcatggagt

acgatcgtcaaacgttattccaacccaatcattttgcgtaagaagcatattcgatcgctcatatcgcttc

ctaagatgaaagaaacgggagcagtggcgctcaaccgtttggttgacgattttcgacggcatgtcaaaat

cctagaacaattgaaagaacccgtgaaatcgttcagttcaattctcatcgagttgatggcggataagctg

gatgatgaaacactccgtgtgtgggaagaagcccatgccgatgaagatcctacatttacggacatgatgg

cgtttttggaaaaacgtataagagtgttggaaacactggcaatagagaagtgtggtgcagttcccaaaaa

accaataaagacaaaagtatcgttgcatgcagctacaactcataccaacaacgtaccagtgtgtgtgatg

tgcaaaaagaacgggcacagtatagcgtcgtgcaatgtgttcaaaggcactaatacacaagaacgcatga

gagtggtgagtgagaaaaggctgtgcagaaattgcttgaaagcaggacatttggcccatgcgtgtgcgtc

caaatacaattgccagcagtgttctcagcgtcaccacacactacttcatgctcacgaagaaaacagcagt

gtattagtgggtgagacttctagctcttcaacaatggcgttggcatcgtcgaagaaatccgccgttaacg

ctatactctctacagtggtattggttgttgtcgatgcatacggcaaagaacacttagcgcgagcattgct

ggacaacggatcgcagccgaacgcgatcagtgaacatctttgtcagcttttacgactaccacgaaagccc

gctagcgtttcaattgctggtgtcgacagcactaccaccaatgcaaagcacatagtatgtacagaagtgc

gatctcggatttaccactaccgacaagcaatgaatttccttgtgttgaagaaagtaacgcagaacattcc

ttcaacgtcgttttctactgctgccgtcggcgttccttcgaactacgttctggccgatccagatttcggg

accgcgcggcgcgtggatatgatcatcggtgcagcatatttctattcgttgctgcgtggtggacaagtgc

atttgccaaaccagcgaaacgttctcatcgacacggtgtttggctggctcgtagcaggagatacaccgac

ctttcatgaatcgcaatcgcaaacaacaattagttgccacatgatggaggcaaccgacaaactacaagaa

cagctggagcgattttggaaggtcgaagagcttgctataacatcattgtctcctgttgaacaacagtgcg

agcagtacttcaagcagacgacgaatcgagatcacaccggcagatacgtcgttcgcatgccgaaacacca

cgactacgctcagatgcttggcgattcgaaggctgcagcccagaagcgctttcggttgttggagcagagg

ctggctaaagacaagcatctgaagcagcagtacgatgacttcatgcgagaatacgtgacgctgggtcaca

tgtttcctgtgccggttgaagaggacagcatggctgcggttcactacttgccgcatcatccggtggtgaa

agagtccagcacgacgaccaaggtgcgtgtggttttcgacggctcggcgaagacaaccacggggcattct

ctaaacgatgtcttgcatgtaggaccagtcgtgcaagatgagctgctgtctctcgtcgtgcgattccgca

agtataaggtggcggtgatcgccgacatcgagaaaatgtatcgccaggtgagtatgcatcccgatgaccg

acgtttacaacgtattttttggcgctttcaggaaacagaagttgtgcaaacttttgagttggcaacggtg

acgtatggtctggctccatcgtcattcctagcaacacgtacgctacttcaactagctgaggatgaaggcg

ctccttaccctttggcaactgaagccgtaaagaagaacttgtacgtggacgatctgatctccggcgcaga

aagcattgagcaagcaattcaacttcgtgacgaactgaccagtctcatgagtaagggaggtttcaggttc

cgaaaatggtgctcaaacgagttgagtgtgcttgatgggttgacacctgatctgcttggaacaacagcat

cccatgaattcgaagcaaccgcaaatgtcaagacgcttggcatatgttgggaaccaccaaacgatgtatt

ccgcttcacgattgctatccctgatgtacgaccctacacgaaacgtacagtgctatctacgattgcccaa

ctgtacgatccgcttggcttgctatcgcctatcatcgtgcaagcaaaaatcctcttacaggaactttggg

caaacaaactcggttgggatgacgaattgccgcggcaattgtgtgacaaatgggaagagttttgcgaaca

gctccccatgctagctcgtttcaagatcccgagatttgctttgacacccaactataactatgtagagctg

cattgttttgcagacgcatcagaagcagcttatggtgcgtgtgcctacctgagatcgcaaagcatcgacg

gcacaacccaagtaacgctgctagcttctaaatcgagagtggctcctctcaaaccacttaccatccctag

actggaactatgcgcagccttgctagctgccagattacagcagaaactgatatcagccattgacatggca

gtaaacgaaacacatatgtggtccgattcaaccatcacgctgcaatggcttgcagcaccacctagaacgt

ggaaaactttcatcgcaaaccgagtaggagagatacaagctgctaccaatggatgcatttggcatcatgt

gccagggatcgagaaccctgccgacatgctatccagaggtgtttctgcggaattgcttttggaaagcaac

atgtggatgcatggaccagattggctgatgaacgatagctcgtgctggcccagcaaatcgtatggacaac

agcacttcactgatgatgagctggaaagaaagggtaacgttgtgttaactgcccaagtagtcgagcccga

cccattgctcctacgatactcctcattcagaacgttggttcatgtaactgcatattgcatgcgattttgc

cacattgcgcgtggtaaagaacaacgcgaaacgagcaatctctctgtggatgagattcaaaatgctaaaa

tcgttttagtaaagatggtacagcgacaagtatttcccgatgaactacgacaactgcgtaagaaacaaaa

gcttgctggtggatccccactcaagctactccatccattcattgacaaggatggtgtcatacgtgttggt

ggcagacttggacatgccgatttgccattctgtgtgaagcatccgatcgtcattcctgggtatcatccat

ttacccaattgctgttgaggcagcaacatgagaaggtgatgcatggtggcatcacatcaacactttcagc

cattcgcgaggagttttggccattgaatggcaggagagcggttcgatctaccatccgagcatgttatcgc

tgcaaccgagccaatcctgttccaattcagcaaccgatgggacagctaccgctttctcgagtcactgcaa

acgaagcatttgtctgtacaggtgtggattactgtgggccgataatgctgaagcctgttcatcgcaaagc

agctcctcaaaaggcgtacctatgcatttttgtatgcatgagcaccaaagcagtccatttggagcttgtg

ggtgacctaagtacatcagggttcctgaaggctttagaccgtttcatcttccgacgaaacaagccgaacc

atatctattcggataatggtacaaatttcgtcggcgcaaagaacgcacttcaccaagtctaccagatgct

gcatgacgaagctcaaaaccgtcaaatcaataactatctagcagaagaaggaattgaatggcaccttatt

ccacctcgtgcaccaaacttcggtgggctttgggaagccgccgtgaaggtggccaagaagcttttggtca

ggcagttaggtgtctcgctactatcttatgaggatctggcaacagtgctgatcaaaatcgaaggctgcat

gaattctcgtccgttgacgccgctttcgaatgaccctaacgatttgtcagctttaacaccgagtcatttt

ctcatcaagggaatgatgcgtccacctccagaaactgacatacgggatgtcccgaccaatcgactcgacc

agtatcagcggttgcagaagtacgctcaacatttctggcagcgctggcgtacagagtaccttcatgagct

tgctcagcaacagcgacgtaatccaccagaacaacaagtctctatcggagacatcgtcattatcaaggat

gaacagctcccacccgctcgttggcccttggctcggatcgtggaagtacaccctgggcaggatgggattg

tgcgtgttgttaccttaaaaactgcctctggggtattgaagagaccttcgtctaagatatgtttgttaga

atgttcacgagaattttgaaaacttagttgttcaagggggccggta

>bel11-ltr_ag bel anopheles gambiae str. pest

tgtttggtaccgcatacggtaggatgtactggtgaggagaagtgtagaggaacgagaattttcggcgtaa

gtgtgtgcgtgtttttgcacacgctgcttgagctgggtgcgcggcgctcagcgatcagtcgcgaatcgat

actgaataaagacacacgtgaactgtacgaacaatataaattcctccgtgtaaggcaaatattgatcaaa

taaaaatatcacaacgtagggcaaacttgctaacgatcacgggacagaaca

>bel12-i_ag bel anopheles gambiae str. pest

tttggtccttcgaaccggatccgaattacggatattctttgctagttttttgtggtttatgctatttggt

taccgagcgccaagtgggagtgcaaagaaatattggatttcttagcagaacggtgtgttctccactccat

tcgtgttgttgctactgctgcaggagccggacactcatcgttggtttgtgtgtgtgagagagagaggacg

tgaagccacgaaaggagctgcgtatctgtgggctgtcggtaaacagcaaaagacgaactgtgcgacgacg

aaaggtgcgaagttgtaatacggtgaatagctgaatttagtgcgtgcgtgaactttccattaataacata

tttgtggataattagtgtcagcgcagaacattttcgaacgcgtgagtgtcgttcaaccgtagtagtggag

tgggtgtgcgtgagtgactatcctagcgccatacttggcacccaaagctgcgcgcgtattagttcaggtg

tcgacaacttccaggggtacggaactcattgctgtgcgttttattaaaaattgtgttaagaagtgcctaa

agaacaattattgggagcaaaatgccagcagcagataaacgagtgaaaatgttcaatttaaagagggtag

aaattatgaacactttgcaagatttcgaagagtttacgaaatcctttgatgcaaccatcgatgcatatca

gatacctagtcggttggaacagttagaagagttggttagtgagttcacggaattacgtaaagcattcaac

gaaacggtagatgattcggaagcgttcgatatcatgcaaaaagatcggcgtgaatttaacaaacggtctc

acgaagtaagggcatttttattaaaaaatagttcccattctggggcgtcgagtgggttgaacactacaca

ggttaacacaactattagtgcaggaactcaaaatcatctgcgccttcctaaggttgaccttccaagcttt

gatggtgaaataacaaaatggcttacgttcaaagacagattttcgtctatggtgcatgactcgacagaaa

tgcctgaagtgttaaaattgcaatatttattatcggcgcttaagggtgatgctgcgcatcaatttgaaca

catgcaaataacggccgataattattatgtgacatgggaagctttgttaaaacgttatgataattctaag

gtgttaaaaagggaatatttcaaggcattttattctctagaaaaaatgaaaaccgactcgacggaagaat

tggcacgtatcgtgaacgaagcaaatagattagtcagagggttagaacgtttgaacgagcctgtcgacaa

gtgggacactccgttaacaagtttattgttttacaaattggacagtaaaactttagtggcgtgggagcag

tactcggtggatttcaaaacagatgaattcacaaatttagtggaatttttggaacagcgagtgaacattt

taaagagctctgcgcaaaatatttgcaatcaatattcggctaattcgatcatggtgaccggcaggcaggc

gagaagagatggtaggaatgtggcattaccagtacagcaaacgaacaatacatttaaagggtatctcaag

tgtccactgtgcaacgaacagcatccgttgcatgtgtgtgagagattcgaaagagcgtcagtgataaatc

gagaggagatagtaagaaaacatggcttatgttttaattgcttgcgaaagggacactcagcacgtgagtg

tagatcgacgtatgtgtgccagcagtgtaaaagaaagcaccattcgaaactgtgtaagataggaagatta

tctgaagtggaagtggttccgtcaacgtcaagattaactgctacggctcaagcaaattgttcgaagaaaa

cagttatattgtctaccgcgcaaattataattctagatgttaacgatcagccatacaaagtgagagcatt

actcgataacggctctcaattaaatttcatcacggagagagtggcacaagaactcagattgaagagagcc

cgcgtgagtgaacagatagctggtgtgggtggagctattatgagagttgcaggatcagttgtgggtacca

ttcgatcactcaccactgagtacacaacatgcttagaatttttaattttgccaaaaattgctaccgattt

accatccgaaacaatggacgtacgaggttggaagttaccaaaagatgttcgattagcggaccctacattc

catgaaaggggctcaatagatatgttgataggggcagacacctttgttgaaatgataaaggcaaaaaaga

taaagcttgatcatgagttaccaacactacttgaaacggaattaggttggattgtgagtggtgcatataa

gcataataatttaaatcaatcaatggcatgcacaattgttagtcaagggggagaaaacgacatagcttct

ttgatgaacacattttttaatatcgaagaagttcaagatcagaatttgtggaacgttgaggaacgagaat

gcgaagatcattttcaagcaacaacaaggcgtgatgagaatggaagatacgtggtgcgattaccactcaa

ggcggagagggaattgggagagtccaaggaagtagccttacggcggctgattggacttgagagaagattt

gagagggaaccgaaggtgaaggaagcatatgaagcatttatgcaggaatatatcactttggggcacatga

gtgtcagagaaaatgaaaatagtagtgacggttactatatgccgcaccacgctgttttcaagcaagatag

caccacgacaaagtgtcgtgtagtttttgatggatcgtgcaaaacgtcaaatggtcgatctctcaatgat

atattaaaagtaggtccaacaatacagcaagacactacggatattttattaagatggcgacgtagagcca

tagcagtggtcggtgatgttgaaaaaatgtaccgacaagtgtgggttcatgaggaggatcgaaagttcca

acgaatactttggagatcacattcaagcgaaaaaataaaaacatatgagcttaatacaataacgtacgga

acggcatcagcgccatttcttgctatacgaaccctaaatcaggtgctagaagacaataaggaaaaatacc

cactagcagcatcgcgtataaatgacttttacgtggatgattttatttctggtgcggattcagagaatga

agcaaaacaattgtgcgaagaaaccaaggcagcgttagcaatgggtgggtttcctttacgcaaatgggct

tctaattgtccccatatattaccatctgaaaccgaaattgataatatacaaagggtaattgaattgaagt

caagagagggtgcagtatcaacattaggacttgtgtggaatccgatcttagacactctaggtgtaaaaat

tagtgaaccagaaacttgtgagatatatacaaaaagatcgattataagaacaatcgcaaaaatctatgat

ccattggggattgtggatacagttaaagcaaaagcaaaacaattcatgcaaagagtatggtcattaaaaa

aagaaaatggtgactcatacgggtgggatgaagaaattccacagcaaatgagacaagagtgggaagtgtt

tgagaggcagttaacacatttacaagaagtacaagtaccgagatgcgtaacgatagtaggagcacgtaat

attcaaatacacggattttgtgatgcttctgaagagggttatggagcttgcgtatatgtgagaagcacga

atggagaggaaatagtttcgcgattatttgtatcgaaatcaaaggtcaccccattagctacaaaacacac

aatagctagattagaactatgcgcagctcatttattaggaaagctattggtgaaactcaaaagggccaca

gaagatccatacgaaacattttgttggacagactctagcacagtaatttattggttgaaatcgtctccaa

gtcgttggaaaacattcgtggcgaatagagtatcacaaatacaaaatgcaacaaaagaatttgaatggag

gcatgtgcctgggattcataatccagcagatgcggtttcgagaggtagaaatcccgcagaggttgttgag

gataagctttggtggcatggaccagattggctagtcaaagacccagaacattggcctaaaaatatagagt

caggaaacacttgtgagacagcgaaagaagaaaaacaaacgaaaactacattaacatgtatggtgaaaga

ggaaagttttataaacaaactatgcgagagagtaggttcattcacaaaactaaaaaggattgtcgcatat

tgtcatcgtttcttcgatcgtaagcgaatccatcgcaaatcttattttgagttgagggaactaaaacgag

ctgaaaagacaatcattcgattggttcaaaatgaagtctatgcaactgaatacgagtgtatcaaacaagg

gcaacaagtagtgcgaaaatcaccattgagagtgattagaccaatactggacaaagataatgtcatgaga

gtaggaggtcggttgtcaaacgccgacataaaagacgaacaaaaacatcctgttattattccaggaaagc

acaggattgcagagttgattgccgacaagtaccataagatacttcgtcatgctggggctcaactgatgat

aaacactatgcagttaaggttttggatagtgggagcgcgcaatgtagcgaaacgtacagttttcaactgt

gtgaaatgtactcgttgtagaccaaaactgattcagcagccaatggctgatcttccagagcagagggtga

gacaagctagaccgttctcaattagcggtgtggactacgcaggaccgataatggtaaagggcacacaccg

acgggcggtgcccacaaaaggctatatttcaatatttgtttgtttcgtaacaaaagcagttcatatcgaa

cttgtatcaaatctaacctcttctgcatttttagctgcactgcgtcgattcgttgcgaggagagggcatg

ttacggaattgcattcggataacggcacaaacttccgaggtgcgaacaataagttgcgcgaactgtataa

attactaaattctgatacacaccaagacgaggttgtaggatggtgcgccgaacgagacatgaagtggaag

tttacacccccagctgcaccacattttggaggtctgtgggaggccgcggtgaaatctatgaaatttcatt

taaagcgcgtgttaggtacagggcatttaacgtttgaagatttatcaaccttattagccgaaatagaagc

atgtctaaattctcgaccaattacggcaatatcagaagatccaaatgatatggaagcacttaccccaggg

cattttttggtagggaatcacttacaaacggtagcggacgtagacatcgcagatgtgccaacaaacagat

taaaccattggagactgatacaaaaacacatgcaacacatttggaatcgttggcatcgcgaatatttaag

tacattgcagaagcgagcaaagtggaacaaaaatgcgatatcgattgagccaggaagattagtaattcta

caagaagacaatgttgcagtatctaaatggccgatggcaagagtagtggatttacatccaggaaaagatg

gtgttacacgagtagtaacgttgaaatgcgcaaatggcaaggaaattcgtaggccaattcatagaatagc

tcctttacctatagaatcgtaaattgaaatcaataattggaattatgtggaattaagatgaggaatttta

agaaatcaaataggaatatgaaaatgaattcaatcattactgaatattaaaaaacattcgtttttggtga

ccgggaa

>bel12-ltr_ag bel anopheles gambiae str. pest

tgttggcgcctaaatggctgcaaatttatagattgtaagattataggaaaagggtacataggattacgtt

agtattagtgttagcaggactgtcagggttatgacgaaaatataaaaggattagtagcaatcagacagtc

gactcggcaacatacctttggaaaccactaaaaaatatcacagtagtttatgagctatcgttca

>bel13-i_ag bel anopheles gambiae str. pest

ttttggtccttcaaatccggatatcgtttgaaaatcgtgatctatggtgatctttcgtgaacattcgtac

gcctttgtgaacagtcgagcattatcgtatgcgttgttcataggtgttcctttcgttgtatgcgccttgt

tcgtgtggagttttatcttgttgttattatctatggtgataatttgaagacagttcctgttagaggatac

tttgttgaatatcatacaattgagtgtgatattgtgcaaattcagcttgctatcgggtatgctgtattag

acgcggacaatttttaggaaaagatacattggtgtgcgtgtacgtgtcgtcaggctgtacctgtttggaa

gtgttggtgagatcgcttcgcagccattttgaagagcagcatcttaaaaagtattgtcggcgcaaatagc

tcaacaaaatgtcggtcaggagaagtgatgtgcacaaaacgccagcggaatccgaggaatggaaaattgc

acccattgacatggaagaagctggaccgtcatcggaaatccctgcgggtttggatgaagcaaaggaagtg

catcggcctattgataaaaaccaatgtatacgcaagcattttcttaacaaattacagcgaatcgaagaag

cattgagtggtacatcgttgggtgacacgacgtttctgaagggatgtgcaaatcggcttacctcactggc

gtcggagtatgaaaaatggcatcaaaccgtcttggaaacggccgatatggagaattttgaggacggtgag

gaggaatatgcacgattcgaaaaacgtcacttcaacttattgctacggattgaacgtggtatgtcgatca

ctacaaatgtttcgcaatctcgtgtaaagcttcccgaattacggctgccaacgtttgatggctctctcga

agcttggctgccttttcgcgattcttttagtagcctgattgatgctaatgcaagcctgtcagatgtggac

aaattgcggtatttgaagggagcattgacaaaggaagcaaacaagctaattgcggacattgagattactt

cggctaattacatagtggcatgggagcttcttaaggctcgttatgaaaacaaaaagctagcggtgaagcg

acatattgatgctttgtttttgataccggtaatgaagaaggattcgtacgagtcgctcattcacatttta

gatagtttcgagagaagcgtcaacataacgaagcagcttggtgtggcaacagagggttggagtgtgcttc

tggcgcacatgctacattctcggcttgattcggctactcaaatgcattgggaggctcaccatcgcagtac

tgacgttccagaatactatgaacttctaacgttcttgaagagtcatgcgttagtgttgcaggcaatgctg

tctccaggccaaaagaaagaacaatatacatcgtcgtggaagcagcgatcgaaaagtgaggtgcacgtgg

taaattcttctatggagatatgctcgttttgtaaaaaaggttcacattcgccctttaagtgtgacatgtt

tagtggctggacagtacaagagcgctatgacaaggtcaaagaaaagaagctttgtatcaactgtttgttg

cctgggcatattatgaagaactgtacatctagtgtatgccgagtttgcaacaaaaaacatcacactatgt

tgcacaaaccagtacaaaccagtaattcagctgaagcatctcctaatgaccgggagatagtgacgcaacc

ggatccacctgcggatcagaacgtggtcacatactgtggcaatgctttgctgacaaattcgatagaaaca

ccatctactatcctattacccacagctttggtgaaaatagagttaccagacggatcgttgcattgggcac

gagctcttttagatggaggatcgcagattaatcttgtaactgagcgtttgtgtcagcgattgcaggtcat

taaaaagagagagaaccatccaattggcggagttggacaaagcaagcatgtatcatcgcactcgacacag

cttaccataaaatctcattgcactagcttcaaggcaaattggaaatttcatgtaatgcgttacattactt

ggaatttacctgcagagaaagtgaacaaaacacgttactgcattcccaacacttgtactctagcagatcc

caaattctatgaaccatcatccatcgatttattgatcggcagagagagctatgatgagctgatgctagaa

ggtattcttaaactggtacccgagaaagtaatgctacagaacaccgagttaggctggatcgtttctggca

gggttgagctcgaacgtcgtcctacatcctctatagtaaatctggtatgtactaatcaagacctagaaaa

tcaactaactaaattttgggaaatcgaatcttgtaatacaaatagcaccatgtctattgaagaaacatca

tgtgagaaggttttctctgaaacaaccacgcgtgatgatcaaggaaggtttattgtaacgcttcctacga

aaaaggatatcgttccacaactcggaaactcgtttgaaatcgccaaacgtagattgaactcgctaaatcg

tcgtcttgcatcaaacaaagaccttaaggctgcttatatagcctttttggaggaatatgttcaattggga

catatggaagaaatcacggaacaacatacgaacattgacacacccatttattacttaccgcatcactgta

ttttacgccctgacagtttaactacaaagcttagagtcgtgttcgatgcgtcttgtgctaccgactccgg

cctctcgttgaatgatgcgttaatggtgggtccagttgttcaggatgatttggtcgcaattatgattcgt

tttcgtctgcccaaatttgcaatcgtagcggacatcgaaaagatgtaccgacaagtatggattaaaaaag

aggatcgctcgttacaaagaatcctttggcagaattgtcccgaaaataagcttcgaatatatgagctaaa

aacaattacgtatggcactgcatctgctccttacttagcaaccaaatgtttgcaaatgctttccgttcat

ggaacttctactcatccggaagcctctagagtactggcaaatgaattttatatggacgacttgcttaccg

gagtagaaactcaaacagaaggagaagaattatgccatcaactaactgacttgttgtcgagtgctggttt

tactttgagaaaatgggcgtctaactcttcccaaattctgcaaagtattcccgtagaccaacgcgatact

tctggattgtgcagccttgacataaatagttccattaagacattgggtcttaaatggatcccagcgaccg

atgagttgggattctgcgtaccgatttggacagaagatgaacaaataacaaagcggatagccttatcaga

tgcatcgcgtctatacgatccattgggactcataggtccaacaataatgatagccaaatgctttatgcaa

aatctttgggcactacaaaaggcatgggatgagccgctagaaaaagaattgcataaacaatggaatcagt

ttcgccaacagctctcaattgtgaaagacatgcgtatcccacgtagagtggtaggaagtacccatcgcat

cgaaatccatgggtttagtgatgcgtctatgaaggcttatggagcctgtttatatatgaaatcggtctct

gaggatggaaaagtttctgttaaccttttgtgctctaaatccagagttgctccactcgcaaatagcaaac

gacagaagaacgtcactttacctcggctggaattatctgcagctttattgttatgccatctctggcaaaa

ggttaaagatagtcttaaacacgagtattcgtgtttctactgggtggattcgaccatcgtgcttcactgg

ataaacagtagcccgtcacgctggaagccatttgttgctaatcgggtgtctgaaatccagcatctgacgg

aacctagacattggaatcatgttcctggggatcaaaatccagctgatatcatctctagaggaatgatgcc

gagtcaattgcaagaatcatgcctatggtggcatggaccagagtggttaagccaaccatccaatacttgg

aagctacatcatccaatactcgattgcccaccctctgagtttgaagaacggaagactgttctgatcatta

acaaacagtctaatattcatcatccaatatttagcttaaaatcgacattctccggactcgttagactgat

ggcatatatgcaacggtttagctacaactgtaaacctgttaatagaaacaatcgtcgtcaaggctacctt

caaacatttgaactacatgcagcaagagagaatttggtacgcattgcacagaacgaatcttttgctgatg

atattcggtctctcgaaactgctggagaagtcaaaacatcgtcatctttaagatcattgacaccaatgct

tgtaaacggtgtactacgcattgggggacgtcttcgaaatgctcctgttgcttacgatcggaaacaccca

atgatactgccctacaaacatccattgacacgtcttgtcatggatttctatcatcttaaaaccttacatg

ctggacaacaactgttgattgcttctgttcgagagaaatactggcctttacgcgttcgaaaccttgctcg

gcaagtagtacacgagtgtatccagtgcttccgttgtaaaccatcgacaatggaacagataatgggagat

ttacccgcagaacgagttactccaacttttccgttcctgaacactggtgtcgatttctgtggaccgctgt

tttatcgttcggcgtccaggaaatctgctccggtgaagtgctacgttgcagtatttgtgtgcctggccac

gaaggctatccatctagaattggtagctgatttgtcgtcggatgcgttcatatcaacactgaaacgattt

gtcgctcgtcggggaaaaccatctcttcttcagtgtgataacgctaaaaattttcgtggagcggaacgaa

aattgaaagtgtttcatcaacaactgcagcaacaacaatttcaacaatcaatttcgtcgtattgcggtcc

agaaggtatagagtttcgttttatccctcctaggtctccccactttggtggaatctgggaggccgccgtc

aagtcttttaagcatcattttagagctactattggaacttcgatcctgcgtcgagacgacttagaaacga

tcatcgcccaggtggaaagctgcttgaattcgcgccctttgaccccaatcagcacggaacccgaggattt

ggaggtgcttactccagggcatttcctgatccatcgtcctctggttgctgttcctgaaccttcatacgag

gaagtgccatctaatcgcctggatagatatcaacagaatcaggaattcgtgagacgcatttggaaccgat

ggagtacagactacctgtctggcttgcagccccgcacgaagtggacgaaacaacgggacaacattcatat

cggaactctcgtgttgatgaaggaagacaacttgccaccgttgaaatggagttatggacgagtaactcaa

atctaccgaggagacgacggcaacatccgcgtggtcacggtgaagacaaaggacggcgaatacaaacgag

caattacgaagatctgcgttttgcccatccattccaacacggaataagtggtggaaatttcaatttccac

gggggccggcta

>bel13-ltr_ag bel anopheles gambiae str. pest

tgttaagaatgcttggtatcatggccgctgacagccaccgagtgacgcaacctgcgctctgacagattac

cgcgtttttgaactgtcaaaccacaagccgaagagttgtgtgagtgtgtgttcgctaggagatcgagaac

agagcacatgcagaaagttgatcgcaaataaaacatcgtaaaaaacccaccgaacagtgccgcattatag

tttgaatcatttcgcttatttcaattaaca

>bel14-i_ag bel anopheles gambiae str. pest

ctcttttgcctacaaaaaagggttcttcagtgcgttaagtgtaatagtgaagaacaatgggaccgaagaa

agcacgtggttgcaaggcttgcggtaatcaggtcgacgacactttgtacgtgcagtgcgatgaatgtgat

gcgtggtggcatttctcgtgtgccggtataacggcatccgtagaagccgtggagaaatgtgcgtggttgt

gcgaggagtgtgccaggaagacgctgagagagcaatcatcgccacgcgagggcaataaggagcccaagga

aggaacctcgaaacacgtggatggggatctcgttcgtaacctcagtttggaagcagcgacggatggcggg

gcgcgcccggttacaaacccacagaggcggccgcttttatcgctcgatgaggccaacgacgagatagcgc

caggaacatcgacccacgtggcagggggacccgtacataacctcaaccaggatgctgcgacggaaggcgg

ggtgcgcccggtcatgacaccaaagaggcggccgctttcatcgctcgatgaggccgacagaggtaaaacg

tctgtctcatcgaacatcgtgcacagaggatcgtgtcctaacctcaacctggatgcggcaagtgatgacg

tggcacgtcaactcgccgtgctgaagcggcgacaggaggtggagaaacggcgcatggagcttgaactgca

gctgaagttcgtgcaggaggaagaggcacttctcgggtttggggaaaataagtctttttcaatttcacca

caacttaactcttttcagactgaaaagagaacagtgaaacgcagcgaagaagaaaaagaggaaccagacc

taactccacgacaagaggctgcgcggcacatggtttctaaagagctcccagttttctccggtgatcccgc

tgagtggccaatttttatatcgcactacgagtatactaccaggcgatgtggatactcgaattgggagaat

atgctgcgcctgcaaaagtgcctgaaaggacctgccctcgaagctgttcggagtcgattggtgttaccgg

acgtagttccgcaggttatcgagaagctacgttccaaatatgggcggccggtgcacttaattaaaacatt

catcgagaaggtgcggaagattccggcaccccaaactgacaagctggacagtttagtcgagtatggggaa

gcagtgcagtgtatggtggaccatatggttgcggctggtgaacgtgcgcatatcaccaacccgctcttgc

tgcaagaggtggtcggcaagttaccaacggatcaacagttacgttggtcgcatcacatccgcggaatgac

ctcggtagatctgtccacattcagcgactacatggaggatttggctgaagacgctgcgaggctgacgaca

attgactctccttcagtgcgcgggaccagcaagggaaggcctacgaagggctacgtccacgcgcacgtgg

atccagatggagcgacaacgtccagcgcggctgagaggcagtgtgtatcctgtaacgtcgcggggcatgt

attgtcgacatgcactaattttcgaggactgccggtaaaggatcgatggaggcgagcgcgtgagctatcg

gtgtgctttagctgcctggagaagcacaattggcgatcgtgcaaaaatcgctctcgttgtggaatcaacg

attgtgcattccgacatcacgcgcttctacacgacccggatgcaatagagtcgccttctactgcagaccg

agaacggcggcacttcccgagaaccagtggaagtcagacgcaccaggtaataaataattatcatcagtcg

aatccgatgtcggcgctttttagaatcgttccagtaacagcgtatggacccggagttatgataaaaacct

tcgcgttcctggacgaaggttcgtcaatgacgctgatggacgaagacctggcaaagcagttaggggtgaa

gggagatagacgacctctatgtatcaagtggacaggtgatacgactagggtcgagccggcgtcgatgatg

atcgatttacagatcggacctgtgacgtcgacaaaaaggttcaccctgaaagctgtgcggactgtcacca

gccttagcctcccacagcaaactttcacgatggatgacaagagatgggaccatcttaagcagctgccatt

accggagtaccgtgatgctcggcctcagttgttgatcgggctggacaaccttcgattggcggtgccgctg

aagacgcgtgaaggccttgcaggggaaccggttgccgtaaagactcggcttggatggtgcgtgtacggaa

agacggctggaagccaaatcggaagggtgctgcatatgtgcgagtgtggagcatcggacgaaaactccac

catccagggggccttacgcaagttttatgagttggagcaactcgggactgtctccagtgacgtgcctgat

ccagatgaacgaagggcactgacgatcctggaaacaacgacggtgcggattggtaatcggtttgaaagcg

gtctgttgtggaagacagacaacgtggagcttccttcgagcttgggtatggcgcgtcgcaggctggaatg

cttggaaagaagaatggaacgtgaccctaagctgaaaaccgtggtgcaccatcacatagccgatatgatg

gaaaagggttatatccacaaggcgacgtctgctgagcttgcagagtgtaattcgaagcgaatttggtacc

tgccgttgggagtggttaccaatccgaagaagccagggaaggtgcgcatcatctgggacgccgctgctaa

ggtacaaggtacgtccctaaatgacatgttgctgaaggggccggacgagttaatttctttgccaggggtg

ttgttccggtttcgaatgtacgggatagcggtgtgcgctgatgtcaaggaaatgttcctgcagatacgca

tgcgcgacgaagacaagcatgcgcagcggttcctgtggcgggaagatcctgctgacgatatcgcaacgta

tttcgtggacgtcgttacctttgggtcagcctgctccccagccaccgcacaatacgtgaaaaaccggaac

gccaaggaacatgccgaaaaataccctcgtgccgtacgtggcatcttgaccagcacgtatgtcgacgact

atttggatagtttcggaacattcgaagaagccagtcgagtatccagagaagtcaggggaatcttctcgaa

cggcgggttcgtactccggaactgggtttccaacaatccggttgttttggaacggctgggcggcgaaagc

tccagtcccggtatgaagagtttgacatctacggcggatgatggagaacgggtgctcggattgcggtgga

acccgagctcggaccaattgtccttttacacgcaggcgtgtgtgggaatggcggagatatttgagacgga

gtgtacccctaccaagcgagaagtgctcaaatgcgtgatgtcactttttgatccgcttggactgttggca

aactttaccatccatggaaggatcttgattcaagacctttggcgagctggtaccggttgggatgaggcca

tcagtcccagtcaaatgcgagattggcgtagatgggtggatgtttttcctctgatagcccagcttaggat

tccgaggtgctacttcccggaggcacgagagaaagtgtacgagaatgcggagctacacttgtttgtggat

gccagccagctagcgtacgcttgcgtgctgtatttacgggtcgtcgattctgaaggagaaccgcattgta

ccatgctatgcggaaaggcaaaggttgctcctctgaagcctttgacgataccaaagatggagttacaagc

ctgcttgttaggtgcacggcttctgaagtccacggaacagcatcacccgatttctgttaaaaaacgggtg

ctctggacggacagcacggtggcgctatcatggatacatgccgaccctaggaattacaggccatttgtcg

cgaatagagtggcggagattcaggagaacaccaacgtgaatgagtggcgatgggtgcccactcaggacaa

tccagcagacgaagctaccaaatggaaagggcgtgcgaacttcaactgggatggcatttggttccagggt

ccatcatttctgctgcaggatgaagagtcttggccgacgagaagactcgtttcaactactccggaggaag

agatacggcgggtcaaccttcaccgtgagaagttgaatcctggacttctccctctaaaagctgaacgctt

cagccgcctggaaagaatgatcaggacgttggcgtggattgtcaggtacgtggacaatttgatgagaaag

gtgggaggagcccctctacaccttgggatcctctctcaagacgaattggagagagcggagacgatcgcgt

ggaagcaagcgcaaggggaatattttcaggatgaagtacgagtcctgagtgtcggtgagggaacaggaag

gagtaccgtgcctaaggaaagtcctatctatggtctcttaccctacgcggatgagcgtggtgttttgcgc

atgcggggacggattggagcagctccggaactgccatatgctgccaggtacccaatcgtattgccacgtg

acgcatggataacccacctgctggtggacaaatttcatcgccggtttcgacacgccaataacgaaaccgt

ggtgaacgagctgaggcagtatttccaaatcccaaagatgagacggttggtttcaaaagtggttcggcaa

tgcgtgttctgccatattcgacgaacattgccacagatccccccgatggctccattaccgaaacagcggc

tcactgcattcgtgaggccgttcacatttgtgggactggactactttggaccgctgttggtgaggagagg

aagagcacaggagaaacgatgggtggcgcttttcacatgcctaaccataagagcaattcatttagaagtt

gtgagtagtctttccacagattcctgtattttggcagtgagacgctttgtggccaggagaggcgctcccg

ttgaggtgttcagcgacaacgggacgaatttcgtgggagccagccagcagctaaggaaggaaatcgacga

gcgcaacgatgccttagctgcgacctttaccaacgcgaacacccgatggacgttcaacccccctggcgca

ccccatatgggaggggtatgggaacgcatggtgcgatcggtgaaggctgcgatgagtacgatgacggaac

tacagcgtacacctgatgacgagacgctgcttacggtgatagtggaagcggagggaatgatcaacacacg

cccactgacgtacatcccgctggaatcggcggatcaggagtctcttactcctaaccacttcttgctgggc

agttcatcgggagtgaagcagagaccggtggcaccgactagccttcagacggggttacggagcaactgga

aaatggtgcaacatatcctggacgggttttggagacggtggataaaagagtatcttccggtgttggcacg

gcaaagcaaatggtttgagactgtgagagagattgaggttggagacattgttctgatagtcgacggtggc

gctaggaatcagtggaagagagggatagtagaacgagtggtttcgggagccgacgggcggatacgacaag

cttgggtgcgaacaaacacagggaccctcagaaggccggcggctaaacttgccttattagagataagaaa

gggtgacaaatagcgtattggtcacgggctggggga

>bel14-ltr_ag bel anopheles gambiae str. pest

tgttacgaaaagaacttaggcgagccctgcaattatctaccaaaccgggcgaaatttgcagcgagttaga

aatgatcgggcgaacccgatcgtggccggagcgcgatgaacaccacacgtgtgtgtgggtgagtgctcaa

gaggccgaaaaggaagaggttcgggttcgggatatcgcagggcattcggcaagcgtacgagcagggaacg

ggttggcagagagcgataggcgaaccttttttgtagttcttgatttttcattgcttttttctttgctcta

actggatagatttgcttaagtacatacagttcaactttagttaatctgttgggtgaaaataaagacatta

ttgtagagctgaactcacaaacgcctactacgcgcctccttgtgcttaca

>bel15-i_ag bel anopheles gambiae str. pest

tttggtgccgtgaccaggattggtgtattgtgaagtgaaattgttgctcgttttttgtgattttgctgcg

tgtaaaaatctgttgactttacgttttttcgtcaccgaccatagacaacgaacagtttctttcgaaattc

gttgtccagtgcttgttgcgtgttgtgacttgattgttgaacattgtgtaattgtttgattggtgtgata

attgatcatacgcacggacagaacatttgtgaatttgtgtttcgtgttcagtgtcctgtgtttttagcgc

catcgttccatctgccattacgagactgatatggctaacgctgtaaatttgctgtccagacggcgaaccc

tggaagaaaaaattcagcgagttatagcatttgctgataattttgtgcctgagcgggatgaatttaggct

tggcttgttcatctccgacaccgaacgtgttgcagcagagttcgatacagtgcagcagttgatcgaggat

ggagcagcacccgaagcgcgcgaaatggagagccattttcgcgctacgactgaggacgccttaatggccg

cgagggccagcctacaagcgttgtcgcggccatcgcataatgttattcccgcctcatcgaccattgctac

atctggagtgaggctgccaaccatttctcttccagaatttgacggcaatgagatgcaatgggcgacattt

cgggacacttttgaagcactaatccactgcaacgaagaggtgctaactatccaaaagttccattatcttc

gagctgcgctcaaaggtgaagctgcaaagttgctggaatcgattccgttgtgtgcatctaactacaacat

tgcctggaaatcgttggtggacagatacgccaacgagtatctacaaaagaagcgtcatctacaggcaatg

ttcaacatcggcaaggtgaccaaggaatcgaacgcatcgttgcacaggctggttgacgattttgatcgtc

acgttaagatgctgcatcagcttggcgaaccaacagcgcaatggagcaccgtgctagaatatgtgttgtg

caccaagcttcccgatgagacgctacggacgtgggaagattatgcttccaccctcagcagcccgaactac

agcatgctaattgagttcctgcaaagaaaaatgagaacattagaatcgatttctatgaaccatccggcaa

cgagagaagctactcatcctagttttgtacggcgagccccacagcacctttcttcctgctcaaccatggc

gagcagttcaaaagggtgcccgcattgccagcatgatcatgccttaagcagttgctataagttttgccgt

cttcctctgtctgagcgttttcagatagctattgagaagaaagtttgccataattgcttaagaaaaggtc

atttggcaaggaactgcgcttcatcgtcccggtgcaaacactgtggtgagagacatcactctcttttgca

tcgttcgtctgcagtcggtacggaaccgaaactcgtgtatgcggaaggacaagaatctacaggaaggaat

gatcgttaccaagcacagtcgctcaacgttactaagcatcctattcgatcggaggaagtgtttttgctca

ctgttcgtttgagcatagttgatgctgatggtaaagagcattcggtacgcgctttgctagactgtgcttc

tcaacccaacctcatgacagagaaacttgtcaaattgctgcagctacaacggtgtccttctaacgttaaa

atatcgggagctggaaagatatctcgtgacgttcggggatcagtgtttgctgagatacgctccaagaggc

aaccattcagctgtggtgttcagttcctggtaatggacaagctgacatccaatttgccttctgagactgt

aagtgtcggtcactggtgtatcccaaaaggcctcgagctagctgatcccgaattcaacacatcgcagccg

gttgatttggtgataggtgtcaagcactactattcattcttccccagtgcagccagagttcatttgggtg

atgagttgccgctattgattgatagtgtgtttggttggattgttgctggttcggctacgttacaatgccc

ggaaccacaggtaacaagttcaaacgctatctgtatgatgtcgctggaagagagcatcgaacgattctgg

aagacagattcattagtgatgaaggatggctactcgcctgaggaacgaagatgcgagcagatattccgtg

atacaacggcgagaaatgagactgggcgttatatcgtacgcttaccccgtcatcccgatttcggcatcag

actgggtgcttccaaggtaagcgcagtacgaagatatgatctgttggagaggaggttcgctaaaaattcc

aagttgaaggaagagtaccatgcgtttatgaaggagtatcttgagctcgggcacatgagtttagttcggg

atggagatgcagtacctgctgagtcgtattatttgccacatcatcctgtgttcaaggagtctagcaccac

aacgaaaatcagggtcgtgttcgacggttcttctaaaaccaccagcgggtactcgttgaatgatgctttg

tgcgtgggaccagtggtgcaggacgacttgctagatcagcttttgcggttccgcacgtataaggtggcat

tagttggcgatatagcaaaaatgtaccgccaaatacttcttcatcctgacgatcgaccgttggtgcgaat

cttgtttcgcttcgagccgcagcagccggtgcagacctaccagctgaatactgtaacgtatggtctcgca

ccttcctcctttctcgctacgcgcgctcttattcagctggctgatgatgagggtaatgcatacccacgag

cgggccccgctctacgaaagaatttttacgtcgacgacttcatcggtggagcccaatcagttgaggaagc

cacctgcctacgaactgaattggctgagctactacaaaagggcggatttgagctgcggaaatggacgtcg

aactgtgttgatgtgctgcatgggctggatgaggcacaggttggaaccacaaccaaaatgagcttcgatt

ctcacgaagccgtgaagacacttggcatcagttgggttccacaaggtgattggctggtgtttgaaggtgt

gtgccagccagacgacgatgtgatcaccaagcgatctgtgttatctgccatcgcaaaaatgtacgatcct

ttggggatgatagcgccgataatcatccgtgctaagatgattatgcaggagatatgggtgtcgtcacgtg

attgggatgaatcgttgccagaagacatcgtatgcaagtggaagcaatttcagaaggagatacgatctct

atcacaatatcgaatggacagattcatactgcttcctgaggcgcgaaacatcgagctgcacacttttgct

gatgcatcttctgcagcctatggtgcttgcacatatgtgcggtgtgaagacgctgggcgagttcgagtca

tgcttttagcgtcgaagagtaaagttgctccgttgaaaaatctgactattgctcggttggagctgtgcgc

ctgcgttctagccgcgcacttgcatcaccgcataaaaggtgccattgatgtggccgtaaatgcatcatat

ttttggaccgactcagctatctgcatgcattggatcaaggcgccaccgagcacatggaaaacgtttgtgg

caaaccgtgtagcggaaatccagcatttcactagtggtgccaagtggaggcacgtagctggagttgaaaa

ccctgctgatttggtatcgcgtgggatggaggtgtccgagttcaacaacagtcgagcgtggaggcatgga

ccagcttggcttgagcattcgcaggatgcttggcccatgcccaatccacaaagcattcctgaagtggcag

agcaagagaggaaggagttaatttcggcagttaccgtatgccacaacgaattgtttctgtattggtcatc

ttatactcgcctggtgaacgttgtcagctattgcatgcgattcattgccatgctccctcatctaaggcaa

ttaagaggaagaagagttttacgatcctccgaggggaatgcgcaactgaaggacgtttcatctcgtaaag

ttctcagcgctcgggagcgagcagcagcagttaatgccttaacacgccttgcccaacgtgagtcttttgc

tgaggagctacgcgatctgcaaggaggaaagagggtcaaaaaacaatcagagctgaaaagactcaccccg

tttgtggacgaaaagggtatcatccgggttggtggacgactgaatttgtcacagctgccgtatcagtcga

aacatcccgcactgctgccgaagggtcatccattggctcgattgattgctgagcatgatcacaagatgct

tcttcatggaggaggacggttgctgctgtcagtcatacgagagaagttttggcctttgaacggaagaatg

ttggtcaaaagcgtagttcggagctgcataaaatgtattcggtaccagcctactcttgcagagcagcata

ctggccagctgcccgctgcccgaattataccaagccggccctttgctgttaccggggtcgattatgcagg

tcccctgtacctaaaacctgcgcataggcgcgcagcatcgttgaaagcgtacctgtgtgtcttcgtgtgc

tttgcaacaaaggcggtgcatttggagctagtgggagacctgtcaactgacggttttctcgcggctctac

ggaggtttacggcgaggagaggtgttccggatcatctccattcggacaatgggaaaaacttcgagggagc

gaggaatgagctacgagagctttttgcgactttgacgagtgaagctgcgcagagtaccatcgcttcatcg

tgcgcggaccagggaatctcttggcacatgattccaccgagagctcctcactttggcggcctttgggaag

ccgcggtgaaaacggccaaacgtcatctgttccgtcacctcggaagtactcggctctccttcgagggtta

ctacaccgtactgcaccagatcgaggcagctatgaactctcgtcctctcttaccgcttacggatgatccc

aatgatttagccgcactaacaccttctcattttttgataggctcctcattaacggcacttccggaccctg

atatgacgatggcacacactaatgcacatgagcacttggcgaagctgcagctattggtgcagaagttttg

gaaacactggcaaaaggagtacttgcaggagttgcaaaaggacccgcgcgttgccaggcaggcagatcaa

atccaacccggtcgaatggttatcctgatggacgagttgctgcctagtacccgttggccgttagcgcgtg

tgatagaggtgcaccccggtccggacgggctggtgcgagtagttaccttgcgtacgataaaaggtttaat

taagcgtccgatagccaaaatatgtcctttgccagtagaagagagaggaaataacactttgccagcagct

acgtaaccgaaatgtccgtatttgtcgatttaatgtttgtgcaccgcgcttcgtcgttgtataaagaaaa

ccacgcggtttatgtcaggtctgagttagtgttagtgttagtgttgatggtacatcaaggcgaggagta

>bel15-ltr_ag bel anopheles gambiae str. pest

tgtttacgttttttgttattgttgcgttttgacagctagggctacgttcatccagcatattgtgtgcgta

ttttcactgggtgaagcgttagcgttcgtttgaattgtctatgtattagtgttaggtccgttaggctaag

aacaaaattgtcaaccattgtcttaaaaggattcgtaatatactgacgcgtacgccaaacggtcaccttt

aaaccctaagcatttttcactgcactttaacataaaatatcacaacgtagggccgatcacgaaca

>bel16-i_ag bel anopheles gambiae str. pest

ttggtgccgtgaccaggatggtctaatatttgcggtttggtttgactggttaaagtgaagaactttgtgt

gatttcttttggcaaaaatcgcgtaacgcgtgtgaaatctgaaagttccgtatcgagcgttggtggattt

ctgaataagaaaaaatcgcgaaagcgtgagaaatcattgtgctagtgggtgttctttgcgtgtgtatgca

ttttgtgtcgcgtattagaaatgtcggttagtgaagattttgccggtttttctgacgcgtcgggttcgaa

caatacaatgcaatacacacaagcagaagatctggatatgattatcctaaagcgcgagcgcgaccgagtc

gtgcaatcgttgacaagaatcgacacgtttttggcgcagtacaaagagagtgatttcccagaattgacgc

ctcgtctcgatctattgaacgagcgttggagggagtttcaggctttggctcgaaatattggcgcaaaaga

ttacagtgaagataacgatgtgctttatggtgagattgaagacaaagtcatgctattgaaaggaaaatta

ttgggaaagctgcgggctggagcggagataccgagtgtgaaacaggaacgcgtgtgcgatgattataata

gtgttcgtttgcctcagttgacgcttcctcaattttccggaaaatatgacgaatggctcccgtatcacga

catgtttgttgttactgttcatgagaacgagaaattgtcgcaggttgaaaagatgctttatttgaagggt

tctctgaaaggtgaagcgctgaaggtggtggatactttgcaagcctgcaattcgaattatgacgtagctt

gggatgctttgaaaaagcgatattctaacgaatatattttgaaaaagcgtcatgttaacgcgatgctaca

atggccacggatgaaagtcatgaacacggtgggtattcatgggcttatcgattgtttcgaaaggaactta

caaattctaaagcagttaggtgaagtgaccgaacagtggggatgtttgattattcagataatcatttcaa

agttggacgaaagcacccaacaaaagtgggaaaggcacgttgaagaaagtgagcaaaaaacggtgacgga

tttgttaaactttttgcgcacacagacgcgcataatggacgcgtttgcagtggataggccaatggcggcg

ggcaaatctacaagtgaacgtcgtgttgcgtctaatgtggctgcagaagcaaagtgcgcaaaatgtgatg

gatcgcacatggtggaaaattgtgactcgtttcggagtttaacgttgccgcgtcgtcgtgaagtggttga

agctaagaagctttgccttaattgtttgaggcaagggcattttcaagccaagtgttggtcacgtgcgcga

tgcaatatttgcaatcgtaaacatcattcccttctacacggtgaaaatgtaagtgaaacgatcggtccgt

cggttcgtgaagaacttccatctaccagtggcacgcaaaatgtggtggtgaacgcgtcatcgaacagaga

gtgcacttctgtgttattatcaacggcgatagtgagtgtgcgtgcgtgtggtaacaaatggttgtcagca

agagcattgatcgatagtgggtctcaggtgaacctgatgacaaagggcttggctgcgcggcttaagttac

cgcagtacgaaagtaaaacggcattatcgggagttggacattcgaaagtggacataacgacgtcggtaac

gactgtcatacgttccaaaagttgtaactatcaagaacgtatgcagtttctagtgttgccgagaatttct

agctacaggccggtaactggaaatcaaattagcaggcagaatctcccgatgaattttgtgctcgcggatc

ctaatttcgatagtgatgctgaagtggatttattgttgggctccgaattttacgcgacttttttgaaacc

ggacaaccgtggtaaaattaggctcgagctaccagcgctcccaacatttattagcactgtttttggatgg

gttgcaactgggaaagttccgctggcttctgaaggtagcaattatgttacttgtggcacgtgtactaggt

tggacgatttaattgagcgtttttggattattgaagaaatacgtgagccgcttcaacatagtcaggaaga

aagggattgcgaagcacattttgtgcaaactcatcagcgggatagtgaagggagatatatagtgaagctg

ccatttaagtgtgacttacagaaccaattgggaccgtccagtgcgatagcgagaaaacggttcttgcagt

tagaacgacgtttcaatcgggacccatggttgaaacaaaagtatacggcggttatcaacgactacatcga

caaagggattttagtcaaggtggctgcgaaccctgattctgaggaagcgcatggtcattatttcttaccg

catcatccggtaatcaaggcgtccagtaccagtacaaaggtacgacctgtgtttgatggatcggcctcta

ccgacggtggtaagtctcttaatgatttgttaatgactggtcctgtgattcaggagaacttgttggcgtt

gttgctgaaatttcggatgaggagcgtggcattagtggcagacataaaacagatgtatctgcaagtcaag

gtgcatcccgacgacactcgttttcaacgtgtattatggcgaggctcatctgttgagtccatcgaagtct

acgagttgcagcgggtcacatttggacttgccccgtcttcctttctggccattcgagtactgcagcaatt

ggcaattgatgaaggggaaaactttcccttggcgagacaggcgttgttagaggacttctatgtcgatgac

tacattggtggtgcctctagcgaagaagaagcagttaggttgcaagctgagctgacgctattgttgagaa

agggcggatttcatctaactaaatggaattctaacaaaccagatgttttatctagcgtttcagcggaaga

cagagcaacatccaacgttaaaatgtttgaagttccagaggagccaataaaaactctaggtatcgcgtgg

ctaccagaatcggaccaactgtacatagactcgaacattcagatgaacaacgagagctggtcccgtagaa

aggtttactctttggtagcacgtatatacgaccctttggggctggtggctcctgtgacatcttgggccaa

gataaatatgcaatcgttgtggttggcaactgatgactgggatgaagaaataccggctgtcatgcaagaa

cgatggtatgcttttcaatcacaactcgggttgctgaaggaggttaagttttcgcgccatgctgttgtgc

ataatcctgttgctgttcaactacattgcttttcggatgcatctgaagcggcttatggggcatgcgtgta

tgttagaacgattggtagcagcggggaagtggtagttgagttgcttgctgcaaagtctcgtcctgcgccg

ctgaaaagagtcagtttggcccggttagaactttgtggagcattactggcagcaaggttgcagaaagtgg

tacgccaagcgttgagaattccagacgtggaaacctttatgtggactgatgcgacaatcgtgttgcattg

gattcgagcaccatcccattcttgggctacgtacgtagcgaatagggtatccgaaattcaggaattgacg

catggctacaaatggatgcacgtgaagggcgttgacaatcctgccgatattgtatcgcgtggagctatgc

cgaacgagctgttagcatcgaagctgtggttccatggtcccgggtggttacaactatcagaggaagaatg

gaagaagaatgccagcggtgtgttggcaattcccgaagaggagttattggaacgaaggaagagctcattg

gtggccgcagtaagtagcgagagcgatgattggtgtgataggttttccaactatgacaaattactgcgga

tcactgcgtattgtatgagatttattcgttgttgccaacgaaagctggatcctaaacacaaaggtgtttt

gttggtgagcgagctagcagaggcgaaaattcgactggtgaaaagagaacaacggatatactttgcggct

gagatcaaggagttgtctgctggacaaacggtacgtcccaaatcatcactgaagacattaggagcttttt

tggacggtgatggtttgctccgagttggtggccgcttgcatcgcgctaaagccatgcaagtttgtagcag

atttccgttggcgctacccaagaagtcacgatttactaggctaatggcagaatattatcatcgattggca

cttcatggtgggccaactgcaacattgagcgcactcaggagagaattttggccaattcaaggacgatctt

tggtcaatagtgtttgcagaggctgtctggtatgcttcaggatgaatcccgcgttagttcaacaaccacc

aggacagctacccgtgtcgcgtgctatgccagctcgaccattttcgatcgtaggggttgatttctgtgga

cccatttacttgaagccggtgcatcgccgagcagcagctgaaaaagcatatatttcaatctttgtgtgtt

tttcagtaaaggctgttcacatcgagcttgtggagtctctatcaactcatgcatttctagcggcgtttcg

tcggtttgtggcaagacggggtttgcccagcgaggtctattccgacaacggtctcaacttccaaggagcg

agtaaggtgatcgatgacttctacacgttgatgaacagcgattcggcggtggaggatatatcgaggtatg

ctgttggcgctggcgttaagtggcacttcatcccaccccatgcaccgaactttggcggcctttgggaggc

agcggtaaaagcggcaaaacgcgtcctactgaaggttgttggtgatcggcagctggcgtttggggagatg

tcgacggtactggcacaagtggaagctcaactcaacagcagaccgcttacaccgttgtcggaggatccgg

aagaacttgatgtattgacgccggggcattttctaatcggggctccgatgaacgctctaccggagcctga

cgtgggtgatgtaccaatcaatagattgaagcggtatgaggaattgcgtagagtggtacagaatcattgg

gcgcgttggcgtagggaatattttagcgaactacataacgaacatcaacgcggcaaggcagtagtagagc

taaaggtaggacaaatggtcctgttgaaagaggatgggaagactcctcaccattggccaatgggacggat

tgctgaggtatttcctggcccagatggcgtagtgagagtcgttagtatcaggactaggaacggcttgtat

aagaggccagcgaataggattagtcttcttccgtttgagagagtgaattagatatcataaagtcaggcat

tttgtgaagtaatggaaagaggtaaatttggtaaatttaggtggccgcta

>bel16-ltr_ag bel anopheles gambiae str. pest

tgttggaatgtaagggttatgaaacggtcattttgaattgtttgcggttgttttgtcagttgggaattaa

aagttaaatgtattttctggcagcactgccgatcgacaatttgtgattaagtatgtgtgcgaataaagcg

gcactagcgcatgaaactcgatacgagccggacgtgttctttactttgtctcctttggcgatcgaagacg

acacaacaaaacacaacgtagggcgtagaggcgtcaagggggaaaggaaccaacaaaccatgttccagaa

cgcaaca

>bel17-i_ag bel anopheles gambiae str. pest

ttaaaaagcctttaaaacggttccaaaggtgaaatgggcccaaaaaggaacgatcactgccaggcttgcg

gtgatcagcgggatgatccggattttgttgcatgtgacaagtgcaatttatggtggcatttttcgtgcgc

cggattgacggaacccgcggaagctgtggaacagcggaaatggttgtgcgtggcttgccaggctaaggaa

cggtccggcttgattcagaggacgccggtcaagcacacagaacaaatggaagcggcaaatgctaacctca

atctggaggaggttacgcaaaacgtggctgagaaacataacctcaaactggttgaggttatccaacaagc

ggcagaaacacccatgccttcttcaagtgggacaagctacaacgcggtgagcaaacctaacctcactcct

gctaaggtatccgacagtgtggctaatcggctagccattatgaagcggcggcaggaggcggagaggaaac

ggatggagctcgagttgcagctgaagtttgtgaaggaggaggaggacttgttgtccgaggagttgggtgt

gatggcgatttcgggagcatcgactgtgacaccgtcccggccggatggagaatcgatgcgaggctcgcag

caggatgagcgtggtcccgccccgcgactggaaagttttcatcgaaacgtgccaaccgaattacccgagt

tttcaggggacccggcggaatggcccgtatttattgcgcactacgattacacaacggagaaatgtggttt

ttccaactgggaaaacatgatacggctgcagaaggcgctcaaaggacctgcgctagaagttgtgcgaagc

cgtttagtgctaccggaggtggtgccacaagtgattgcgacgctgcgatcgcgctatggtcggccggaac

atctcatttcagcgctgattgggaaaatgcgtcggatgcctgcaccttgcagggagaagcccgatactgt

tgtggcgttcggcgaggcagtgcggagcatggtagatcacatgcaggctgctggtctacgggcacacttg

accaacccgttgctgctgcaagagatcgtggaaaggttgccgacgagcgagcagtacagctgggcacggc

acatacgaggcgtgacggaaccggatcttatcgtgtttggcgaattcatgacggaatggatggacgatgc

tgaaacgttaaccaggctggattcaccctcattgaaagcagtagacaggaagaaacctaacaccaagggt

tacgtgcatgcgcacgtggaaaaccagggagcgaccacatcgggaacaagagccatccaggtaggacaat

cttgttttgtgtgtaacaaacggggacaccttgtgagcaaatgcttcgcgtttggagcaatggcggtgaa

ggaccgctggcggaaggcccgtgcactttctttatgttttagctgtctggagcggcacaactggcggacg

tgccaaaacagagcggtttgcagcattggtgggtgcacgcgccgacatcatgcgttgctacacggcgctg

aggagtctcgcgagatcggcagcgagcagaataatatagaaagccgtgaaggaggaatcgatggtggtgt

cattgcggagagtaaccatcaccaatacgtgtcatcatcatcgaaggcgctatttcgaatagtgcctgtc

accgtgtatggaccggctgctacggtgacgacgtttgcgtttttggatgaaggctcgtccatgacgctgg

tggatgacgatttggctgaacaattaggtgttgagggcaaggtggagccactttgcatccgttggacagg

caacactacaagggtcgaggctggatccagacgagtaaacctgaaggtgggacctgttggttctacaaag

cggtttgccatccactcggtacggactgtgccagggctaaacctacctcgacaatccttcgtgcaggacg

aagggagatggcaacatttggagcggctaccgattcggcaatatcgggatgcggaacccaagctgcttat

tgggttggacaatttgcggttggcggtccctctcaggactaaggagggaggcgttggtgatccaatcgca

gtaaagacacgccttgggtggtgtatttacggaaaaccggcgaatctggagtgtgaacggttgttgcata

tttgcgagtgcaacgaccaaggtaacattcacgagacgattcgggaatatttcgacatgcagttgattgg

tgctgcacacggcgtcgaacaggatccagatgagcggcgtgcgaagcagattctggacactaccacggca

cgaatcgggaagcgattcgaatcgggattactgtggaggaaagatgacatcgagctacctcctagcatcg

acatggcgcgtcgtaggttcaattgtttggagaggaggatggagcgagatggacatcttaaggaacaagt

acatcgccagattcgagatttgttgagcaagcagtacgttcacaaggctacgttgcgtgagctggaggag

gctgaccagcgacgcgtatggtacctaccaataggggtggttaccaacccaaataaacctggaaaggttc

ggctgatttgggacgcggcagctaaggcgcatggaacatcgttgaacgacatgctgctgaagggaccaga

cgagctgagctcgttacttggcgtactcttccgatttcgtctgtacgcagtggcggcgtgtgcagacgtg

aaggagatgtttttgcagattatgatacgaaaggaagacaaacacgcgcagcgtttcttgtggcgttacg

aaccaacggacgagttggaaacctacatcgtggatgttgtaatgttcgggtctgcgtgttcacccgcaac

ggcgcagtatgtcaagaaccgaaacgctcgagagcacatggaacaattcccacgagcggtggagggaatt

attgaaagcacttacgtagatgactttctggacagcttcgagacggaagaagaagcatgtcaggtatccc

atgacgtaagggagatttttaggaacggcggatttgagttgaggaattggacctcaaacagtatggaatt

gatgagatgccttggcgaagcaaatggcgacatcaaatgtttatcatccatgggagatgaggcggaacga

gtattgggaatgcgatggaaccctgcatctgacgagcttggattttgcactagggcgtgcacgacggtgt

ctgacctcttgatagcagagaggattccaacgaaaagagaggtattgcgatgtgtgatgtctctttatga

tcctcttgggctgcttgcgatgttcgtgatccacggcaagatcctgatacaagatctttggcgaactggc

acgcagtgggacgaagagattaacgacatgcagttgagacattggcgtagatggattgatctgcttccgg

caatagcagacctacgcattccgcgcagttactttgcggcagcatcgaagaagatgtacgagaatggcga

atggcatttgtttgtcgatgcaagtcagcacgcttatgcatgcgtcttatatctgaggatatttgatgat

gctggagaacctcagtgtacactcatcggtgggaaagctaaggtggcgccactgaagccacttactattc

cgaagcttgagcttcaggcttgcgtgttgggagcgagatttttacgctacacgcaggagcatcatccgat

taatgtgagacgacgagtgctttggtcggacagcacagttgcgttgtcgtggataaggtcggatccaaga

aactacaaacccttcgtggctcatagggtggtcgaaatactggagagcacatcggttgacgagtggagat

gggtgcctactgaccacaacccggcagacgaggccacgaagtggaagggaaagccgaatttcgactttgg

tggcaactggtttcaagggccagagttcttactccatggggaggatgattggccatcacagaggcacaac

agcgacaacccgtcagaagaaatacgacaggtaaatcttcacgtggaagactctaacaccggactattac

ctatccggtatgaacgttttagccgattggagaggctacaaaggatgattggttggattgtcaggtatgt

gggcaatctgagacgaaagtatcgtggcgaacctattttaggaggtgctctacgacaagaagaactctat

gaagcggataagattctgtggaggcagacacaactcgagtactatccggaagaagttcgcattctgagtt

tggacgataacgatggaaaacctggaggaagaacggtatcaaagcaaagccacatttaccatctattgcc

gtttgtggacgatgaaggcgtattgcgaatgcgaggaaggataggtgcagcggctgatgttccttattct

gctaagtatcccgttatactgccgaggggttcccgactggctgagttgatagtagagcggtatcatcgat

tgtatcgtcatgcgaacaacgaaacagtgacgaatgagttacggcaacaatttcagatcccgaagctgag

agcgttggtaacgaagacggtgaagaactgtgtcttctgtaagatcaggcggtcactaccacaagtgccg

ccaatggcaccgttaccaaaggagaggctcacgccctttgttaggccattcagctacgtcgggctggatt

actttggaccagtgttggtcaagagaggaagatcgaacgagaagcgttggatcgctttattcacgtgttt

gactgtgcgcgcgattcacttggaggtggtgcacagtctatcgacggaatcgtgcgtgttggcggttaga

cgatttgtggctagaagaggtgcaccggtagaaatcttcagcgacaacgggaccaatttcttgggtgcta

gcaggcagctgcggagggagatcgaggagcgcaatgaaactctggcggcgattttcacgaatgcgcacac

ccgttggaccttcaacccacctggcgctccacacatgggcggcgtgtgggagcgtatggtacgctctgta

aaagcggcgattagcacggtgatggaggcaaagcacgcacccgacgacgagacgtttgagacagtgatct

tagacgcagaggcgatgatcaactctagaccgttgacttatgttcccttggacccggagaaccaagaggc

aatcacaccgaatcatttcctgttggggagttcttcaggtgtaaagcagcagccagtgttacctacgaac

tatagggatagcttgaagggaaattggaagttagcgcagcatatgctcgacggaatatggaggcggtgga

ttaaggaatatttaccggtgatctcgcggcagagtaagtggtttgaaaatgtgcgggaaattaggaaagg

agatttggtactggtagtggacggaacaatcaggaaccagtggaagaaaggaatagttgagcgaattatg

gcaggacctgatggtcatataaggcaagcgtgggtacgcaccaatacaggagcagttaggaggccagtag

ctaagctagcactgctcgacatagcaacttagggtgaccaaatatggttggtcacgggcgggggaa

>bel17-ltr_ag bel anopheles gambiae str. pest

tgttaggaaaaggcagtgctattgatgagtccaacccggacgtataaacgagatgaagtgaaatgacagc

tagggaaagtgacagatgaaatgacagggaaacggttgcacgcgcttcaaaccagtagcgagtgaagcga

gcagcaggtgacaccgttttaaagttttattgctattttatttggatttatttgtatttgtcttcaggag

aataaatagttaaactaaaactttcgccttgtggctgtacgctctcgcactcaaactgtgtttaca

>bel18-i_ag bel anopheles gambiae str. pest

ttttaaaaaaattccacgtttggaaattttacgcgtttacgcgagtgcgtgagtgaaccgtgtcgcaaga

cagttgaggccgcgaaacacctaggaacgcgaccaacagacgatacgtcggacggcragagaatagttga

ggtgtacgttacacattacaccaacatacgcaatttctcactatggaacacgagcacgaatygaaagcac

ctccagcggtagcacaaacgaacgagtctcagcaacctgaaacgggagcactggatcataggccactygc

accggccgcggattcacttcacttccgatcgttcgcgccgactggacaagattggacggttaactcgtct

ttggagatcgttgaccgcgcaaggtcatcgaccccgaacgcaccattaggtgcaggattgttgctgaggg

raccgcagcaacggttgacttcggaggttcgcgaagcccctgcatcgcggtacacaccgcttgcaaactc

tactgtggcagacctggggmctttacatcgctcatcctcggaaaccgtgccacggttagcgcacctaccg

accactgcggacgtrcaacaaccagcgccatcgcagggaggcgtttctgcaggaaccgtgccgcgggcgg

cggacggtgcatctggagcggccatcgaggacatggaactacsaccgcaagcgcaggacggcgcttctgc

gggaaccgtactgcggacggcggtcggcgcatctggggcagccatttcgaacgttcatcagccgacgcca

tcgcaaaccggcacacccgggaggacgatggcggacaagcttgcggagtcgagcgaagctgttgccgcct

tggaacttcggatgcgaaatttgactcgacgaaagctggagcttgagagacgagagctggagtttgaaga

gcagctgatcttggctgaatcgaagaggatcggcgatggcttcatcagcatgattgacgacgaaggtgag

tcaaacaaaatggttgattggtcgagacgtggggatagtgaggggcatagttcacaycatccgaatgtgt

caccttcttcatcgcgtactgtgcctccaggaaacgcacaacagggaaatgattcagtggtcagtcagca

gcgtcagcctagaagtggagaatatttaaatattgctgatggggcggttgatacttacccgttccgtaac

agcatcggaaccacgttgatgaatgtcaaccagagtcaaattctggctcgaaaggcaagctgcaaggagt

taccgtatttttccggcaaacccgaggagtggcccatttttatcgccacatatgaaacctcgacagctgc

ttgtgggtactccgatgaagagaacactttaagactgcagcgggcgcttaaaggcaaagctcttgaagcc

gttcaaccatgtttgcttcatgcctctaaccttactagtgtgatagagacgctgcgcatgttatacggtc

gcccggagataattgtgcattcgttaatccaccgcatacatcaaatgcctgcaccacgaatcgagcgctt

ggaaactgtaatcgattttgggatggcggtccgaaatatgtgtgccactataaccgcttcggggctagaa

gaatataagtgcaacgtggcattaatgcacgagttggtagaaaagttgcctcatgcgcttcggctagact

gggcacgacaccgtatgcaatcgagctccgcgacactctcggagtttggcaagtggatagaaacccaagt

taaggcagctagtctgataacattgccatccctagaattcaggccagagagaaaactcgaccataagagt

agaacgcatcacatgaacatacataacgctacggggttggttagtgaaggaagtcaaatttgtctactgt

gtgaggccacttgtgttgacctktcgcagtgtgagcagttcaacagactaaatgtgaatgatcgatgggc

aactatacgcagactgaatgcttgtcggaagtgtctgaaaatacacacctacggctgcaaagggaaaaag

gcttgcgggaaaaatggttgcgagtayctgcaccacgaactattacacaatcccgagcgccattcaaaac

caaacgagtacgcggttgccacagcatcactgaacgcgcattctgaggttaccggtgatgtatttttaaa

atacatccctgtaatagtttacgggcgagacaaggccatcacgacctttgccttttttgacagtggatct

accggtaccttcatcgagcatagcctaattgaggarctcggtctggaaggacagccccatcctctgtgcc

tgaagtggacaggtaactcggaacgcgacgaaacgggttcaattcgcgtgtcacttttggagatttcagg

agtcgggcaaaacagcagtgtacatattatyccaaaggtgcacacggtgcaaagcctttctctaccagcc

cagactctggcagtgacacaactgactaaacagtacgcgcatctacagggtttrccaattcatgcgtacg

aaaacgctaggcctcgattgctcattggtatcgacaaccatcatttggttcgaccgactcgttacgcgga

aggtgggaaatatgagcccgttgcggcaaagacgtcattgggatggatcgtatatggcccacgtaayaag

aactccaacagtgttaacatacaagcaatacataccatccacatatgcaactgcggcgcagatgcagagg

ccaatctggacgcagcggtaaaaaacttttttacgctcgaatcgctcggtatcgtcaaaccgttggaaac

acttcgttcaaaggatgatgagcgagcgttggggattctcaacgcggaatcgaaattcaacggaaagcat

tatgaatccggattgctgtggcgtttcgacgatgttaagttgccatgctctcgtgacatggctatgagaa

gatacaaatgcctgcaaaaaagaatgtcgaaagattctgtattagctaaggcagtcattgagaaaatgag

ggactatgaaaggcagggttatatacgacgattatcgcctgaagaactgtcgatgaaaggttcccgagat

tggtttctgcccatatttccggttttcaatgcgaacaaaccaggcaaggttcgagtagttttcgacgcag

cggcgaaggttcagggtgttagcctcaacacatatttactaaatggaccagatctgttggcagggcttct

atccgtattatataaattccgtgagcatcgtgtggccatagttggggatattaaggaaatgtttttccaa

gtccgtatgaaaccagatgatcaacgatcccagatgattttatggaacgagaacgactttacaggtagcg

aaccggatgtctatgcagttgctgttatgactttcggtgctgcgtgttcgccgagtaccgcacagttcat

taaaaatttgaacgcagaccgttttgctgacaaatattcacgggctgtaaaatgcataaaggaagaacat

tatgtagacgatcttttggccagcgctgaaactgacgaagaaatcatcacgttagctgaacaagtccggc

atatccatgctgaaggtggtttcgaaatccgaaactggctgtctaactcacatcgtgtgacatctcacct

acaacgcgaggcatcacccgaagacaagataaacatgtgctccgatgatcgcaccgaaaaggttttgggt

atgtggtgggatacgttaaccgacacatttactttcaaactatcccccaaacacgatatacagctgctat

ctggaggacggatgccaacaaaacgcgacgttctgcgaacagtgatggcgatttacgatccaatgggaat

catcgcgaattttctcatgtacgtaaaaattcttatgcaggaaatttggcgtgctggccttggatgggat

gatgtgttgtccggtcgactagccgaaaaatggagcgtttgggttgcggtattgccgactatttcacaag

tacgtgttcctaggtgttatcgtcagttcacttcggtaaatgcaaaaatacagcttcatgtattctgcga

tgccagcgagaacgggatggctgcagtggcgtttttccgtttcaacgatgggggtattatcgaatgctct

ctggtgggtgccaaaactcgcgtagctccgattaagtttgtatccatcccgcgcctagaactacaggccg

cagtcatcggagcccgctttgctgcagcgattatcgcccaacaccgaatagccatcgaacgcattttcta

ctggaccgattcacgtgacgtgatatgctggatgcggtctgatcatcgccgatttagtcagttcgttgct

ttccgagtaggcgaactcctggagacgacttctgtgcatgagtggcgctggctatcaacaaagttaaacg

tagctgatgatggaacgaagtggcagaaggtaccaactgcagatcccgatagtcgctggttccgcggacc

ggacttcttgtggaagcccgagtgtgagtggccagttcccgaacaatatcccaccgacacgaaggaagag

ttacggctacacatgatgcatcataacacaaacacgaagccatggattcagctcgagcgtttttcatcct

ggaatcgcttgttaaggtcagtagtgtacgtcttacgatttgtctcctttatcgtttctaagagaacggc

tagaacaacaggaccgctaacacaaggcgagcttgaaaaagcagaatatgccattcttcgcttggcacag

agggatgccttttcacaggagatacgacgattaaccgacgcacgtgacgcaagcgaacaacgttgcacat

ggaaatccgtgttgccgaagacgagcatacttgcaaagctgtcccccgaggtcgatgacaacggagtact

gagaatgcgcggacgcctgaccaattgtccttgggttagcgaatctaccaagcggcccgtgatcctgcca

cgacaacaccaggtgacagcactaattttagcagattttcatcgtaggttccagcatataaaccatcacg

cggcaataaatgcgatacgaagcaagctatacataccgagactgcaagccgaattcaatcgtatccgtag

gacatgtcagcactgcaaaaaccgtgatgccaagccagagcctccagagatggggaaccttccctccgag

cgtcttgctgcttatcagaagccgttctcattcacgggcgtcgactattttggcccggtaaccgtagcag

ttggtcgacgagtcgaaaaacgttggggcgttctcttcacctgcctcaccaccaggggcatccatctaga

ggctgcgcactccctgaccacatcatcatgcatcttagctatacgtcgtttcatcgctaggcgtgggcaa

cctttggaattcatcagcgacaacggtacgaacttcgtcggtgcatcgcgagagcttgctgaggcctggg

aagcgatagacaaacagcgtttagcggaggagtttacaacaccgcgtctcgcgtggaaatttatcccacc

tggtggaccacactttggaggctgctgggaacgactagtgcgatccgtcaagaaggcgatgagcgaaata

cggatgtctcggctaccaaccgacgaagtgttaacgacggcattaacggagatcgaagcgatgcttaatt

ctcgccctcttacacaggtaccacttgacagcgaatccgagcttcctttaaccccgaaccactttctgct

agggacagctaacggagaggcaccgaaagcagtattcagtgacgacatcgctaccctaaaaaccacatgg

aaggtatcagaggttatggccaatctcttctggaagaagtgggtagcatactacttaccgaccttaacgc

gcagagttaagtggcatcatcaggttcgtccgattaaggagggcgacattgtagtgatcgtggatccaaa

ccttccccgaaacacttggcccaagggacgggtagtggcggtcatccagtcgaaggacgggcaagtccgg

cgcgcaaccgtagctacaagcaccggaatctacgaacggccggccacgaagatagccgtgttggatgtac

aacaggaaaataatacttacccgccggagaccaacagtcracagcactaagaataacccacgaaatggta

agcgaaaacacatggtcacgacacaacaacaataacgaactgaagagtcaagagcttccgggcaattgac

tgggcgggagaa

>bel18-ltr_ag bel anopheles gambiae

tgttattgtttaacacaggaaaacatcgaatgtcagcgcaagtgtcaagcagtgacatttgacccacgtt

actgctcgactgtgggactgctcgactaacgatgaggatcgcaagcccgcgaatttgcaatggtggaaaa

gccataaaagggaaatttgggaaagcactttctctttttctcgtcatcaccgcggaaccagaagcagtgg

aatttttttaaccactttgttataagtaaatttacagtaccaccgttacgtactgcgttaatagaattca

ataaagtgaagttaagagaacctaactaacgtcgcgagtgaatcatttcgggaaaaaaaatatcaccgga

cgttgctaacgcaaca

>bel19-i_ag bel anopheles gambiae str. pest

tttggtggctccagagaggataataattcattagtatttttatttaccccgtaagagggacccttcattg

ggacaacctggccaaacaatattagtatatgtgttgtattgtccaaacccccttccccgccccctcgatt

ccccaccccctcaaccgagtcgggccagccaggagccccgctgccagggaaggactcaaccgagtcgggc

cagccaggagccccgcagccagggaaggactcaaccgagtcgggccagccaggagccccgcgccagggaa

ggactcaaccgagtcgggccagccagtagccccgctgccagggaaggactcaaccgagtcgggccagcca

ggagccccgctgccagggaaggactcaaccgagtcggaccagccaggagccccgcgccagggaaggactc

aaccgagtcgggccagccaggagccccgcgccagggaaggactcaaccgagtcgggcctgccaggagccc

cgctgccagggaaggactctaccgagtcgggccagccaggagccccgcgccagggaaggactcaaccgag

tcgggcctgccaggagccccgcgccagggaaggactcaaccgagtcgggccagccaggagccccgcgcca

gggaaggactcaaccgagtcgggcctgccaggagccccgctgccagggaaggactcaaccgagtcgggcc

agccaggagccccgctgccagggaaggactcaaccgagtcggaccagccaggagccccgcgccagggaag

gactcaaccgagtcgggcctgccaggagccccgctgccagggaaggactcaaccgagtcgggccagccag

gagccccgcgccagggaaggactcaaccgagtcgggccagccaggagccccgcgccagggaaggactcaa

ccgagtcgggccagccagtagccccgctgccagggaaggactcaaccgagtcgggccagccaggagcccc

gctgccagggaaggactcaaccgagtcgggccagccaggagccccgcgccagggaaggactcaaccgagt

cgggcctgccaggagccccgctgccagggaaggactcaaccgagtcgggcctgccaggagccccgctgcc

agggaaggactcaaccgagtcggaccagccaggagccccgcgccagggatagactcaaccgagtcgggcc

tgccaggagccccgctgccagggaaggactcaaccgagtcgggccagccaggagccccgctgccagggaa

ggactcaaccgagtcgggccagccaggagccccgcgccagggaaggactcaaccgagtcgggccagccag

gagccccgcgccagggaaggactcaaccgagtcgggccagccaggagccccgctgccagggaaggactca

accgagtcggaccagccaggcgccccgcgccagggaagagctcgtgggagctaggccgctgccagggagg

tagccgcccaccgccccgcgccagggaagagctcgtgggagctaggccgctgccaggaaggtagccgccc

accgccccgcaccagggaagagctcgtgggagctaggccgctgccagggaggtagccgcccaccgcccct

cgccagggaagagctcgtgggagttaggccgctgccagggaggtagccgcccaccgccccgcgccaggga

agagctcgtgggagctaggccgctgccaggaaggtagccgcccaccgccccgcaccagggaagagctcgt

gggagctaggccgctgccagggaggtagccgcccaccgcctcgcgccagggaagagctcttgggagctag

gccgctgccagggaggtagccgcccaccgccccgcgccagggaagagctcgtgggagctaggccgctgcc

agggaggtagccgcccaccgccccgcgccagggaagagctcgtgggagctaggccgctgccaggaaggta

gccgcccaccgccccgcacgaggaaagagctcgtgggagctaggtcgctgccagggaggtagccgcccac

cgccccgcaccagggaagagctcgtgggagctaggccgctgccaggaaggtagccgcccaccgccccgca

ccaacagcaaagctgagcgccgtagaaggtcggtaagcgatttactatttttactataattaagagtgaa

gtgaagtgaaacacaaaaaaaatggcgacatcaaaggcgagggcaatcaaggaagcggaagtgaatctcg

tagaggtgatagttggtctaaaacaggaattagcggaaaccaaagaaaagtgggaaaaggaatcaaagga

attaaaaaaagtaattgaaagtagggcaaaagaagatgaagcgtcaatcctcttaaaggagcaacgccag

gctttcgattcgatgatgatcgaacaacgacaaatttttgatgaatggaagaaacagactgaaaatccgc

aagtaacgaccactccaattaactctaccagtaatttagaaagcatagtacattcattagcggatgcact

gaaaacacgcactaacactgctatcctcgatcttcccgaatttgatggggactataaaatgtggcctagg

ttcaaagctattttcgataaaactaaccaagaaggcaagttaagtactacggagcaattagcccgtctat

caaagagccttaagggaaatgccgctcagagtgtaagccgtctgatgattgacccggctaatgtatcaaa

aataatagaccggttggaagaagaatatgggaatgcaaaaatagtttacaaagcacttttagcagacctc

atgcaaaatgaaaatccatcccttagtaaaccaaagacatttttaagcttcatcaggtcattagacgacc

ttgttacaaacatgacagtattaaaaaaggaagaatatcttacggatccaaggctattagacgatttggt

cgataagcttccagaagacctaaagcgtgagtggttaataaccttgattcgagagaaagaggaaggagga

aatagtcgcattaaaacactaaacgattttttgaagtggcttaaacctacagagaagcttgccatcctat

caaatgtaaatgaaggccgggaggaggctatatgtgataaaaacctaagttcaccaatagaaccgaaccc

aaatagaaataccagaacctgttgccatgtttgtcagggagaccatcgcattgtcgattgaagaagattt

cggggtatgcgattaaatgaaaggtgggacgccgttagaagatttgggttatgtacaaactgttgtaaca

acagaaatcataatgctaataattgtcgattaccaccccagtgtcgtgaagccaattgtcatatgaaaca

tcatccattattacattacactaacaagagtcaaaataatttaaacacattgaaccatcacagtaccacc

gaaggaatattttaccaaataataccggtctctgtcattcacgaagacaaagaaattgatacatttgcct

ttatagatacaggttcttccgcaagtcttttgttaacagatattaaagaaagtttgggtgttcaggggat

aagaaagccattggctctgacatggaccaatggcgaaatgcaggaggaagctaccagcgaattagtcaat

ttgcaaataaggggtatgagtggcctaatacaaccattgaaaggattgaggacaattagggaaatgaatt

taccttcacaaactttaaacgcgaactcgttgaaaacccgatacaaacattttgatggaattgatcttcc

aaactacatagatggagcaccaaccattttgttaggcttaccacacgctcaccttatttgtgggtcggaa

aatcgcgttggtggtccagatgaacctattgcgatcaaaacctcattaggctggtcagttctaggtaagg

gatcgaaaagggaaaacaaaggtagcttgtttgtgctaaatgaaaaatgtgataaagaagaaaaaggaat

ggaggaaattatgaaggaattttttacaaccgaaggttttggtgttcaacctagcccaaatataataacc

ccaaaacatgaagaaagagcattgaacttgatgaaaaatactctaagaccactagaaacgggctatgaaa

taggacttttatggaaagaggatgatataaacctcccagaaagctatacccaagcgcttagacggttgca

gggttttaaagaagacttgaaaaagatgtaaatcttaaaaactggtactacaacgaaatttcgttatact

gccaaaagggatacgctaaggaagttaacacggaaatacaaatacacaacagaaatttaaactatatacc

acatttcgcagtagtgaacttaaacaaattaaacccaaagccaaggttggtctttgacgccgcggctcgg

aacatggggatctctctaaattctcagttgctgacaggacctgatgcagtccctccactcattggtatcc

tgttaagatttagagaaggcagcattggagtctctggcgatattcaagaaatgtttcgccgcattagaat

tcgagcagatgacagatgtgcacagcgctttctttatagggaatccccaaattattcaccaaagatcatg

gaaatgagagtaatgatctttggcgcttcttgttcccctgcgtgtgcacaatacgttaaaaactacaacg

ctggattatttcggcagcggtaccctgaggcagttaaagctattgaaaataatcattatgtggacgacta

tctagatagtttcacgtcaatagaaacagcaacccaaagagtaaatgaagtgatacttattcataatcaa

gcagatttttttattcgaaatttcatatccaactctagtcaattaaccaaccaaattccttgtgaacgta

tttcaacccaacctgttttgaagataagcgaaggcacctcaaattttgataaaattctagggcagtactg

ggaaaaagaaaaggatgtattcaagtttattttaaatgatctgaccattcctggccattcagtcagcaaa

cgagaaatgcttgcaaaaacaatgaaaatatatgatccaatgggactccttaccaattatactatagagc

ccaaaatactaatacaagaagtatggaagttgaagatggattgggatcaaaatattcctcctgatctaaa

tagaaaatggcaagactggttgcacaggttgaaggaattagaaacatgggaacttcagagatgttattca

caagcaggagagccaattacaagagagcttcacacatttgtcgatgcgtctgagcaagcatttgctgctg

tcacatatattcgcacagtccataaggaaggagtagacatgcgattggttgcaggaaaatcaagggtagc

gccacttaaaattttaacaatacctcgacttgagttgcaggcagcggtaatgggggcgagactagccgat

actatccgatcggagttaagactcaacattgataatatgcatttttggtccgactcaagaaccgtgctaa

gctggatatgttcggaacctaggaggtacaagcagtttgtagcctttagagttagcgaaatcctctgcaa

aactcacgcgagtgaatggcattggattgcgtccaaagacaatcctgcagatgaagcaacaaaaagaata

aggggggattccatatggatatctggtcctttatttttacgacaaaggaatttcacaaccaccaattacg

attctactactctagtagatatcagaccgatgtttttaatacactctactgaccttatagattttacagt

cataaattcacattggactagaagttgggttaaattgaaacgagcattagccattgggttattatacatg

gagtggttgaaatcgaaggctcagaaatataaaatatcgttggaaataactaaaaaacaattagataggg

cagagaaattgctcataaaaaggctcaaatggaaacgtttaatgaagacataatgttattaaagattgga

aaggaaatatcaatgaaaagccccataagaaatttaaatcctgtgctagatacagatggtctgttgcgaa

tgaaaggaaggctaactgggttatcatttctcgaagaaaacgcaaaaaatctcataatattaccaaagaa

acatgaaatcacccatgtaattgtacggcactaccatgaacgattataccacaagaagttcgaaacagca

ctggccgccttgaggcaacgtttctgggtaatagatagcagagcagtacttaaaagagtaagagcgacct

gtcagagttgcaaaaataacttagctaaacctcaacttccccaaatggccgttttacccgcttgcagggc

agccgtattttgcaaaccttttacgcatacgggcgtcgactatttcggtcctctaaccgtgactataggg

aggcgatctgaaaaaagatggggtgcgctgttcacgtgtctctcaacacgagcagtacatctagaattgg

ccacagaccttagttctgcttcgtttataacatgcctgagaaaaatgcaacaccgaagagggaaaatcac

gcacatgtatagcgataatggaacaaatttcataggtgctgaacgcgaaatgaaaaggttgcgagaaagg

tgtgcaaacgatggcatagaatggcattttaaccccccagctgcacctcacttcggtggggcatgggaga

gaatggtaggcgaagtaaaaagtttactccctctcaagcaagagtcttatcgtgaagaagcgttgcgcgc

gattctggtggaaattgaattcattattaacagcagaccattaacacatataccattagagcatgaagat

gatgaaccgttgacacctaatcatttcctattgggttcatcgggagaagctgtccctacacttcgagaag

ctacatttgctgaagctaccagaagcagctggaaaagggttcaattagtagcgcaacattactggacacg

gtggattagagaatatttgccacaattaaacaaacgcgagaaatggttgaaaaaggtgtctccaattgaa

gttggcgatatagtggttttcccaaatgaacagattaacggtaaatggcaaaagggtcgagtatcaaagg

tctataaagcaaatgatggacaagttaggtcggtcaccatcacatccgggttatcaacagtcaatcgtcc

ggtttcaaaggtcgcaaaattagatgtacttccaaaatccattatagctgaggcgcagcaaaaagtgcaa

tagtccattccaaaaaaaaaaacaaacaaaaaaaaaacaagtaataattataattataatactaagatta

aaaaaaaaaaacaattagtagcaacaatagtaatcagaacatagctttgttcttgcagccacattcgcgt

taaatattaggtgcatcttcgtgtggaagtacatgcaataataataataggatataagtgaattaattat

tggcgagtttatttatgtttttaatcttgtgatgttcaatttaacttattattttatcatgcttatttat

tcaacttttaatgtgtatcgttaagtaattattctataatttaagtatttggtcaatttaatcatagagc

gtcaatttaatgtctcaatcttccagtaaatcagagtaaatgaactgagcctgagtgttcagcagtcgtt

agtctgagaatattcgatagccatttatcaataacggcaaccgccgtcggctagtttattgtaacacctc

ctagaggaattacgggagggggaa

>bel19-ltr_ag bel anopheles gambiae

tgtcgccgacagcggtaattaggctgcgccaaaagggtatgcaatctgcatcacaaccaacaacaacaac

aaatgtcaatagcgtagagcgtaggacgtaggaccaagggccaaaggcttaccaaccaacaacaacaaca

aatgtcaatagcgtagggcgtagggccaaagggccaagggcttagcgatcgctagagcagcatgtgccag

caggatgcatacctttcttggaaaatttgtaaaaacaaggttttaaatccccaggggtaaaaataaatga

attatactaactggagaaaaca

>bel2-i_ag bel anopheles gambiae str. pest

tttggtggctccagagaggaagaagatttcgcggaaaattacgattccgaaactatcggttcttgaaaga

cggaagaagatttaacagaaaatttctttttcgaagctatcggttcctgtgagaattttctctacgaagc

attcgatctagaagtctgttgtgtcggagacataatttttctgcttgtcgaaagggatctgtgtattctt

tgctgcacagatatactaactttcaacaatggcgagcagaagattatattttgttgaaaatgagcaaggt

gcttgtcgcttgtgcaccaagccagatgacattgatgatatggtacgctgcgatgaatgcgaccgttggt

ttcacgcttcatgcgtaaaggtgatacgattgcccgatgaagatgaagaatttgtttgcgtgaaatgcar

aaacgatagggcggagtatatggraatttcgcaatcaacaaatcaggatacaaccctaaaggcactaatt

gaagcgttgaaaatgagtggtttgacctcatcaactcatatcaagcgcatgaccctcaatagtttgccaa

attttaatggtacttcaaaagattggccgaagttcaaacgwgcctttgaagaaaccaccgaggaaggaag

tttcagcaatgtagaaaacttaaatcgtttacaacatgctttgaaaggagaagcagaaagatgtgttcgt

cgattgtttctcgaaccagacaacgttccaatcattatgaagaaattagaagaacagttcgggagaccag

aacaagtgtatcaagatttacttggagaagtattgaaggttagagtcgagaaccagatgaagataccgga

tctgtcagatgctttggaagacatgatcatcaacctcaaagccattaaaaaagagggataccttcaagac

catmgwttagtggatgaattaatttttaaattttcgaccgataaacaattgaaatggatcgaatttaagt

ctagccttgagaaaragaacaaaataccaacacttgaacattttagtgaatggctttatccgatagcaga

aaatatcagaaaattgccgaaaaggaatgaaagatttcgacaacctttaaactttcatcgtcctcaattt

tcgccaaatcaacaacgtccagcggaaataaacaacaatcgtccgatgaatcgtccaccacaacctcata

attctcacccgatgaacacacaaacaagaccattcaatacacgtgtgcggaacgttcaacgatttttcca

gccatgtccatgttgtcagggttctcacgcactatatcgatgtgaacgttttaagaatattccggtacat

gaaaggctagaaatagtggtaaacagccagcagtgtcaagcatgtttgacatcgaataatcatagccaga

acaattgcaatgcggctagagaatgcggtattcaaggatgcagagaaacccatcatcagttactgcacac

tggagacacagttcggatgaattaccatcaaacaattcaaaatgtctactaccagattgttccagtggtg

ttgcgaaacaataatcacacgttagaaacgtacgcatttttagatgccgggtcatctttaacactcatcg

aagagaatacggcaaataaattgcacttaaacggtgtaaccgatccattaaccctaacatggacacaaaa

cctatcggtgcaggaaaactgtagcagaagagtgagctgcatgatcaaaggagtgaacgagaaaaaagaa

catttacttaatggtatacggactgtgaaaaatctacaactgccaagtcaaacgytatctggcagcatat

tagcagcacgttatccgcatcttaaaggcatcaagctatccgactaccagaaagctcgtccaactgtgct

gattggattaaaccacagtcatcttttaatgccgcttggtcgaaagatgggacgaccggaagaaccaatg

gcgattaaaaccaaacttggatggttgatttttggcatcgacaaaatatgtctatcagaaacaaatcact

taatgattcacaagagcgaggatttgatgactgaaatgatgcgacgatatttttccaccgaagagtttgg

cgttaaaccggtgaaaacagtaaaatctcaagcattagagagagcggagaccatcatcgagaaaacactg

aagaaaacggatggtagatacgaagtaggcttgctatggagagatgatgacgtcatattgccgaacagct

atagcaacgcgctccgtcgcctagcaacgcaagagaaacagctagcaaaggaccctggtttgaaaaattg

gctgtgcaaaacatttgaagagtaccagcagaaaggatacatacgcaagcttacgaaagaagaacttaaa

cggcattcacaaaaaatattttacattcctcactttgtggtagtgaacaaaaataagccgataccaaagc

cgaggttagtatttgacgcagcagcaaaagtcaacggcgtttcactaaattcactattattaactggtcc

agacgagatggcatctttatttggagtgctycttcgattccgtgaaggacccatctgtgtgtgtggtgac

atcaaggagatgtttcaccaagtgaaaattcggaaggaagatcaagatgcgcagcgaattttatggcgtg

acggcgatagtaccagaacaccagatacttacgtcatgcaggttatgacgttcggagcaacttgctcacc

ggcatgtgcgcaggtagtgaaaaatcgcaatgcggaagcgaatagtgaaatgtacccgttagcacttgaa

ccaattcgaaaccagcattacgtggatgattacctcgatagtttcttttccatggaaaaggcgatcaaaa

cggtgactgaggtgattcaggttcacgaaaatggtggattccatataagaaacttcatctcgaataagcg

agagttgatggaagctattcctcaagaacgacatcaggtgaaggctatcgtcgatataaaggaaaaagat

tcgtgtgtcgagaagatcctgggtgtccagtggaacacacaattagattgcttcgggtacaaggtagatg

ttggacggataaatctggaaaagaagccaaccaaaagagaagctttgagcttcgtaatgagtgcatatga

tccacttggcctcattagtcacataaccatccagggacgtattctaatgcaatctatcaacgccgcaaca

aatgattgggatacgcagattcctgatagtttgcatggaaaatggatcgaatggctcaaaatgatcacca

gcgttaaagatttagccatcccaagaccaattgttgcttcaattatgaatccagttgaaatccacacatt

tgtagacgcttcacaggaagcatttgctgcagccgtatatgcaagaagcaatttcaatggttgctttgtc

gtacgactagttgcagcaaagtctagagtggctccaacgaaggctttgtcaataccaaaactcgagctac

aagcagcagttttaggagccagattgactgctagtgtaattaaagagcttcgattaaagattagtcgtac

aatattctggagtgattcgaaaactgttcttgcgtggattaacagcgaacatagaaagtatgaagttttc

gtatcgcatcgtgtaagtgaaatattggacacaaccagtgccaaccaatggagatgggtatccacaaagg

ataatccagccgatattgccacgaaaatcaccagcaattcgtggacatggtacaatggtccacagttttt

acaagagcatgaacgcgactggcctggagtacatgaagtagctgtggaacagcacctcgttaccgtgcac

aaagaaacaattgctccagaatactatttttcatcgtgggacaaattgctgaagcaccaaacagttatga

aaaaatacgtcgatttcttgaagaaccggagcaatttttctcgaaccatatcgtacagggacatggaaac

ggctaaactctcattactgcgaaaagctcagtgggaaggttttcctgaagagatggaagctctcgaaaaa

agaaaggagatttcgaacaaaagcagcattcgaacactagtaccgtttctagatgaaaaaggaatattaa

gatcaagaggccgtctagaaaatgcatcatgcttgccatacagcgcgcgtttgccaattatcttacccca

aaggtcacgtgttgtgaaacttctcgttcgagactaccacgagaaatatatgcaccaggcagataatgtt

gtaataggagttttaagacaaaattactggattatcaatctacgaacagtcttaaaaaatgtgaaaagtt

gctgccaaagatgtattttgaacaccgctgctcctaaagcacctctcatggcaccgcttccaacatatag

aacgcatccatacaatcctccatttctacatacaggagtagattactttggtcctctagatgtgaccgtt

aagcgctctactgagaaacggtggggagcgatatttacctgtatgagcacaagagctgtacacctggaat

tagcagagaaactggacaccgatagtttcatggtgtgtttgaataacttccttcatcgccgcggaaagat

aacacatctgtacagtgacaacggtacaaattttgttggcgcagaaaaagaattaaagaaaattgtcgaa

gatatcgatctaagaatgggacgtgaggctgcactaaaatataaaatagaatggaagttcaacccgcctg

cagcaccgcacttcggaggttcctgggaacgtctgattcaaaacataaagaaagcattgcgacatatggt

tactgaatggaagacgcggcacccaacaccagaaacattaagagcgactttaattcagatcgaatccatt

ttaaattcgcgacctttaacacatttgccgctaacgtcagaagaagaagaggttcttacaccattccatt

tcttaattggtagaggtgtagattccttaccagcacctactgaaacatcgcaagtggatcggcaacaatt

tagattggcacagcacaatgccaaaacattctgggatcgatggaaaaaggaatacctgcctactttgatc

aaacgaaataagtggacgtgcaaggtagaacccatcaaggttaatgatatagtgatcatcacgaatgata

acgctccacctggacaatggcttaaaggaagartagtagagactgcaacagcaccagatggacaagttag

atcagtgtcggtcaaaacagtccaaggtgtaataaaacgaccagcagtgaaggttgcagtcatagacgtg

aagcagaaggagcatttgctgtttgtgaagaagcctccgcattcaccaacaaaaagaaaagtgctatctg

aagaaaatttgcagaaagtcccgccaaagaaacgtatgatagctccatgcaattgggctccgaaacttgt

acaacagttgaaaaaagaagatgatgtatgaatcgcggccaatatagcataatccattgggatgaattat

ggtggggagaa

>bel2-ltr_ag bel anopheles gambiae str. pest

tgttacgcacgcgattctaaatttcttttttttaccaatctaatcgtagtaaccattgtttatccttcca

agtcaaaataagatgtttctggaaacacgaattatcccgtccgaacggatattcgggtaaacttgggagg

ataattttagtataaaagtaggccatattttgaataaagcaggattcaaacgcagaagctcttggagtaa

agaggtttaattctaaatatccgaaataggacagagcccctggatttccgagccaaacacccccttccgt

ctgcataggcttcccaaccttggatcagaagcaactagcgggaaggactggttagtaggcgaagtatcaa

atactctgtccactctgctgctgtttgtttctcctccgttacaaccctgcaactcttgctgagggccaaa

ccttcgcgtggcctgccggagaagctcggttcgtaaca

>bel3-i_ag bel anopheles gambiae str. pest

ttctaaaaattcagctaaaattctatttaaaaactcagtcttataattcttctaaattcctctcacacga

gctgtgcaaattcggctcgttctctacttgctctattataccacgcggtaagcgctgagaacaaaggact

gttcggtaaggtacacgcacatacaaatacacagccaacgcatccgaatcgtgtagtgcaaaaatgcaag

aggaaagtgcagtgatcgcggactttacgtgtgcggtgtgcgataaggcggattcggtagattcgttatt

gcagtgcgatttttgcgataaatggtaccattacgagtgtgcgcacgtggataaaacggtagagacgagg

gcatggtggtgtgcggagtgcgaagtgaagaccaaactagcgagcggcaagaaagacgacgagatagcgc

gcttgaagagagagatggaggctcttaaagccacgacggagaaagcgttagctttgatacgggagaagga

tgcggaagtagctcggctcagtaagagcagcgagcgaacctcgttttcgcccggggaatcgcttccttgc

tcaacggctaaacggatttcggtcaacgaagaaggtgacctgagccaaagccaaatagcagcccgtcaag

cagtgcgctatgaactcccttcattcaatgggaatcccgaggaatggccgatatttctgtctacctttcg

aagatcgtctcgtacgtttgggtttaccgaagacgaaaatatccttcgattgcaaggagcgttacgggga

aaagcactacgaacggtgcaaggccgtctccggcacgctgacaatctggaggaaatattgagcgcgcttg

aaaaatcatacgggcgaccggatgtgttagtgaatacgttgctcgaacaaattcgcgaatcaccgccgat

taagtccgagcggctcgatagttttatcgaatacggcgatttagttgctgaaatatgttcaactataaag

gcaagcggaacttctgacagattgtacgatgcagcgctgcttcaagagctggtggaccgtatgccggcat

atctgcgctggagctggggcatgcatagtcaggagctgaaaagtgtgacgatgagcgagttcggcgcctg

gattcagaaggcgaccgatggagcaatggcggtaactcctccgcagctgaagaagaagacgacaacgcga

caagtgcatgcgcaacacgtggagacgcatcagccccagcccaggaggcatcgagagtgcgcgttgtgta

acagtgatacgtgcggtacgatcgcggagtgtcgggtgtttaaccggttgagcgttgctgacaggtggga

caaggtgcgcaccttgaagttgtgcaaacgatgtttgggcaagcactacggcccgtgctcgaagcgtgac

gactgtggtgtccaaggatgcgtcgctaagcatcaccggaagttgcatcgtgtcaccagcgaggaacgcg

tggagataaatcatcatggaacgcgctccgatggtacattgctgcgttacgtgccggtaaagctgcacgg

tgagagtggtccaatttgcacgcatgcgctgttagacgaggggtcgacggtgacgttgatggagcaggag

ctcgccgggcaacttggggttagcggcgttcttgacccgttgtgtttgcagtacagtgcgggagagcgac

gcgatgaacgtgattcggaaagggtagcggtgcaagtctccagtgctgaagaaaatgcatccgcattttc

gatggccgacgtgcgtacagtcagccggttatcgctccctatccaatcggtcgatgtgaacgagttgaag

cggaaatataagcatttggaggcgattccagctgcctcgtatgaggctgtttctcctcgtttactaatcg

gtatcgaccattacagattgaccagacctttgaaaactatagaaggacagccaggacaacctacagctac

gaagacgcgtttgggatggctcatttttggcaaatgcacggataacgctaacgacacatccattgtgcag

ccggagtctagctaccacgtatgcgattgccaaggagagacctcacgggcagatcgtatgatggcagcgt

acttcgaagtggaaggttatggtcctgcgaaggagcctttgttgtcgaaggaggatcaacgtgcgatgtc

gattctgcaaaacaacacaaaacacgtcgacgggcggtacaccacgggcttactctggcggagcgacaac

gttttcatgccggaaaaccgtcaaatggctctatctaggatggagtgtctggagcgtaagatgagccgag

atacgagccttgcggagaaaattaacgcgatactagaggactacttggaaaagggctatgccagaccgat

aagagcggatgagctgaaaaccttctaccctaggaaatggtatcttccagtgttcccagtgacaaatcct

cacaagcctaataaagttaggttagtatgggatgcggcagctgaagttagaggtatctcgctgaacaaga

agctgctgactggccctgacctgttgacgccgctgcaagccgtgctattccgtttccgtgaatatcgagt

cgcggtggcagctgacatccgggaaatgtaccaccaggtacgcatttgcgatgatgacgtccacagtcaa

cggttcctatggagatggggaaacacaaatgcagagccgcaggagtttgtcatgctgaggatgaccttcg

gtgcagcatgttctccaagtacggcgcaattcgtaaagaacgagaatgcagagaaatatcgttccctgta

cccgcgtgcagttcgctgtatccatgaagaacattacgtggatgacatgcttacaagtgtggaaacggaa

ccagaagcgattgagctggcgtatcaggtgagcttaatacacaataatgctggattttctctccacaatt

ggctctcaaatagcatcagagtcgtgacagcggtaaaaggcactgagtcaaccctcaaggaaatggattt

cgaaccgtgtctaaagccagagaaggtgctgggaatgtggtgggacaccacaacagatagtttcggcttt

aaactatcccgtgtaagacacctggagctggcacgcaaggacaaaccaccgtccaagaggcagatgctgc

ggactttgatgtcgatctacgacccgttaggtttgatcgcaggcgtgcttttttatttgaaagtacttct

tcaggaagtctggcgcctacaccttggctgggatgacgaagttccggaagagatccagcataaatgggac

gcctggatggaacgactgccagaattggaaagtttcatcataccacgttgctaccgacagctggcgtcgc

tcaccgaatcatctctacagctacacgtgttcgttgatgcgggtgcagatggttacgcagcggtcgcgta

cttccgttttgagtgccatggacgtatcgaggtgtcgttagttggatccaaagctaaagtggcgcctcta

aagtatctttccgtgccccgcttagagctgcaggctgctgtgatgggttgcagaatagcctcgtctataa

ctagtgctcatcgagaaactattagtggaagctacttctggacagactcgactgatgttatagactggat

aaacgcagaccaccgtaagtactcgattttcgtcgcacatagggttgctgaggtgctggacacgacgaac

gtcgacgattggcgatggcttccaactaaacttaatgtggcggacgaagcgaccaaatggaccaacctgc

agcatcatctcgcctccgaacgatggtttagtgggcccgagttcctgcaactacccgaagcagaatggaa

catacctcgtcgagtaccatcggaaacgtccgaggaggtgcggaaaaaggataggctgaagctggtcggc

atccacatagcgcgtccgattttcatcgactacgagagattttcccgatggacgcggctggtcaggacga

tggcttacgtgtgtcggtatgtaaacattattaccaaaactaaatctccatcgacaggtccgcttaaccg

cgacgaaatccagcgggctgaaacggtaattctgcgagacgttcagaggaacgcttttaccgatgaatat

gccatactctggaaggcgagggaaaactcaacaacaccgtcgtggaaaagtccgattcccagaagtagtt

tgttgttcaaacgaagcccctatatggacgaggacggcttgctgagattgagcggaaggatcgatcgttg

ccgttacgtcgaccccgggaggaagcgacctattctgctaccgagacgacatcgtgtttccgagttaatc

gttgacgatgtccaccgtcgttataagcacggcagccaagaaactgtagtgaacgaagtgagacaacgct

ttgatataccggctctccgatctgtgtgtagacacgtacgcctgcaatgccgaacatgcacattgttata

cgcaaagccagcgtccccggaaatgtgcgaattacctgctgctcgtcttgctgcattctccaggcccttt

tcatatacgggcattgattatttcggtccgatggtaatcgtgaacggccgaaagacggaaaaacggtggg

gtgttttgttcacgtgcttgacggttcgagccgtccacatcgagcttgtgcaatccctgtctacgagtga

ctgcttgatggcagttcggagcttcatggcacgccgcggaacaccaatcgaaatagtctccgatcgtgga

acgaatttcgtgggcgccgatcgtgaattaaaggaagctgcggaacgtgttgattcggcgattttaaatg

aattcggatcgcccgatccggtgtggaagtttaacccccccgcagccccacactttggaggatcatggga

aaggatgatccaatcggtcaagagaatgctgtcgcgcaccctcacggagaggcatcctacggaagcggtc

ctgtcggcagcattgatcgaagtcgagaacatgctcaattctcggccacttacacacgttccagtcgacg

gtgaggatgaggaaccgttaactcccaaccattttttgttaggttcttccgcaggaatgaagcccttggt

gaagcctgatgattcgccagcaggcttgaaacaaaactggagagctgtacaggctaaaatgaacgagctc

tggaagaagtggatcaaaacctacctccccaccctggttagaaggacgaaatggttcgagtcgtgcaagc

ctatcgagaccggagatgttgtcttaatcgtggacgaaaacagcccgcgaaactgttggccgagaggaag

ggtggaacgcgtagtcccatccaaagacggagtcattcgtcgggtcgtcatcaaaacagcgaaaggaacg

atgctggagaggccagtggtgaagctggtatcgctgaacgtcgcgccgagggttatcgtttgacgacgtc

gaaacgcctggtggac

>bel3-ltr_ag bel anopheles gambiae

tgtcacttatggtgacgacgcgatcgttcgaggatcgacaaggagcgtccgtattttagctagcggccgc

tagaggtctcggactggacgataaacgttcggactgaacgaaagggatcgaccgagcgtttgacggatag

ccggtgaccgcatggactgcgaaatagggctttctttttgatcccggacctgcgtggaagcggacgaatt

tttagttttttcttcactctctcggctactaaaattttgtgctgttaaaggcaaaataaaataatcctga

attacttaaaaaagccgtttgtttggtaaattatttatctgcaggccgggcgttgtgctaacgcgcaaca

>bel4-i_ag bel anopheles gambiae str. pest

tttggtccttcgaaccggattagtgtgcgagtgatccacagttcgcgtttagggaagtttgtgaaaagta

aagtttgtgttagaaagtgaaactatgcccgttgaccattctcccgtcaaagaagtggttccagcggcca

acaacatggaggaaagccaaagttccaccgtcagagaatttggctccgttccagacgtcaccaaattgga

ccgtcaacgcactcacctggagtggcagcttgaccgtctagaacagatgttcgtcaaacacagcaccgac

gttcactcgctgaaaaccatagcagaccgagtgaaggtgcttgctttggactacaaacaatggtacgact

cgatcttggacgttgtgagcgacgaccaggctcaggaggcaatcgagcgatatggcgtgttcgacgacaa

agtattcgagttaagcaaaaacatcgaataccagttggcatcgcagatcccattgcctacaacaccaaac

ccatttttcggcagtgagcagaaacccacgacagtcccggtgcgtgcaagacttcccgaaatcgtgctac

cacatttcgacggaaatattcgcgactggcctgccttccgtgacgcgtttcaatcactgatacattcgtc

agaacagctgaccgaatgtgacaagctgcattaccttgctgcgtcgctgacgaaggacgcacgcgcggtg

atcgacgcattggaaattacatccaaaaactacgacgtagcctggaagctgttgtccgagcgctacgaga

acaagtacctcatcgtgaaaaccaccgttgaggcgttattcaacatatcgccactgaagagagaatgtgc

agattctctaagccgactcgtcgacgacttcgagcgtaacctacgaatgctcgaaaaagaaggcgagaaa

ccagatgcatggagcacgctgctggtgttcaggctaagctcgctgctggatccaacaaccctgcgtcatt

gggagcttcaccggaagtctacgaccatcccaacgtacaaagacttagtgcaattcgtccgcaaccattg

ccatgtactgaagtccttctccaagccagccagcggagccagaaccggagacaccatgcgcagcaaccct

cgagtgcaaaccatccatgctgcgaccagtgctgtgcatagtgttgcgtacaacgaaaagtgcaaattgt

gcggtgtttcgaaacattcagtgttccggtgtgaattaatgaacaatatgagtgttgcagatagaaagca

actagtgcagtcgaaaggattgtgcttcaactgcctttctccagctcatcgtttacgacagtgcacatca

agcggatgcaagatctgccaacaacggcatcatacgttgctgcacgaagcagcgccagcaactgaggcgt

cggatgctccatccacgagttctacctgtcctcctcagtcccttacccactgttccatccagatcgaaaa

cagtgttgtgttgctgcaaaccgtgctggtccaggtagaggacaatcacggacgatgtcatctagcacgt

gccttgctggattccggagcacaactcaacatcgttaccgagcgccttactcaacggctgggtgtggcga

aacggcgagagaatcatcgcatcggaggaatcggagaagtttccgtgacgtcacaacattcggctgtgtt

aaagatccattctttagacagcgaatacacagcgtccggaaagttccacgtactcagcaagctcactcgt

gagctcccatcaagccgtatcaacacaagcagctggcagatacctcgtcaagttcagctggccgatcctt

cgtttcacagtcctggcccgatcgacctcatcatcggagcagagctgtactacgacgtcgttaaggaagg

actcatcaagctatcccacgaaagagtgacactccaaaacactgcttttggttgggtgatagcaggcaga

gttaatgttcatgcaccaccaccaccttcatccatcgttggacacgtctgcagtacgagcatcgaagagc

agctgagcaaattctgggagctggagtcatgccgagctaccagcacattatccgtcgaggagagcaactg

tgagaaacagttcgcaaccaccaccaccagagacaccgatggtcgattcatcgtgcagctaccgaaacga

gaggagaagctggctcttcttggggattcgaaagggatagccacacgccggttccttgccttggaacgtc

ggctatcctcgaatgcctcattgaagacagcttacacacaattcatcgaggagtacgcagaacttcagca

catgacagaagtagccgagagtgatgctacaacttcatccccctcatactatcttccacatcactgcatc

gtgcgaccagatagtaccaccactaaactccgagtggtgttcgacgcttcgtgtgcatcagacaccggaa

catctttaaacgatgcactgatgatcggacccactatccaagacgacctgatgtccattctattgcggtt

tagaatgtcgaaatttgccctggtagcagacatcgagaagatgtaccggcaaatcaacatagctgccatc

gatcgtccgctgcaacgaattctgtggcgaaattctccaaccgagccgatacgaacgttccagctcaaca

ccgtcacctacggcacatcatgtgcaccgtatttggccaccaaaaccctgcaagtgctatctcaggtcgg

agctagcacccatcccgaagcggcaaccatcctcggacgagacttctacatggacgacatgctgactggc

gtaaacagcattcccgagggtcaacgagtatgtcagcaactcatcgatctcctggcttctggtggattct

gcttgcggaaatgggccaccaacaacaggcagatcttcgaacatctgccccagcatctgcaagatgaaag

gacgattctcaacttggatgcgaagtcaccgatcatcaagacactcggactgaagtggaacgtttccacc

gacgcttttgtattcaacatcccgcgctggaacgcagacaacatcatcaccaaacgaaacgcgctttcgg

atgtcgcgaaactgttcgacccaatcggactggttggaccagttatcatccaagccaagctgttccttca

agagctgtggagatgtcagatcgcctgggacgagccgttaacaccagcactacaaaaccgatggcttttg

tttcgcgagaagttggccatgctgcaaacgatccacattccacgctggctcttaaccgatcaacgagcaa

cgaatctacaaatgcattgcttttgtgatgcatccgagaaggcgtacggagcggcgatttacttgcgttc

gaccaacaccgatggacgtgtgacaaccaacctcatcactgcaaaatccaaggtggcaccactagcagac

tcccgtaaacaaaagcgtgtttgcttaccccggctggagctttctgcagcactgctactggcacactcgt

acgagaaggtgtcagacgccttgaagcttcaggtcgagaccatcttttggtctgactcaaccatcgtcct

gcattggttgtctgcaactccgtcacgctggaaaaccttcatcgctaaccgggtgtccgagatccaacac

atcactcacggcaaggagtggagacacgtaccaggaacggacaatcctgcggacatcatctcccgaggaa

tggatgcagatcagctggaaacttcaaccctttggtggcacggaccagactggctggcgcaaccatcaga

ggaatggccgaacactcatcaacctcgacaggaagaattcaccacggacgaattggaggagcgaccaatc

tgcatggctgtacaatccgtggctccgaacgaactttttagcctccgttcaacgttcaccggactgcaac

gtctggttgcgtggctaagaagattccgacacaatacgaatcctgctaatcatcaacaacgcagattgga

tcatcatctcagcttggaggaactagccgaatctacactgtgtctagttcgcctagctcaagctgaatca

ttcccagaagacatcaaacatctatcgaaaggcgattcggtcggcaacaactcacctttaaaactactag

caccgtttctacaagatggcctgctacgagtgggcggaagattgcgacatgcaccaatcccgttcgaccg

aaagcatccgtacatcttacccgccaaccatccgctgacgaatcagattgcaactctgtatcatcggacc

tatcaacatgcaaatccacaactgctaatagcgagcatgcgagaacgattctggccactgcgagcaaaaa

acctggccagaagaatcgttcactcctgctacaaatgctaccgttgccgccccacacctgcacaacaact

catgggcgaccttccagcagagagagttacaccaacgtcaactttcttacacaccggagtcgacttgtgt

ggaccgatacactatcgacacacatctcggaaggcgcaactcatcaagggctacgtagcgattttcgtct

gtatggcagtgaaggctgtacacatcgaattggtcgcagacctgtctaccaacgccttccttgcggcact

tcgacgattcatcggacgacgcgggaaaccggctatcatcgaatgcgacaacgctaggaatttcttgggc

gcctcccgagaaattgcctccttgtccaagcaattcaaccaccagtggcaaacatcagtgattaagtcct

gcatcgacgatggcatccagttcaagttcatcccacctcgctcacccaactttggaggtctttgggaggc

ggcagtgaagtctttcaaaacacacttcaagccaaccgttgggaacgccatcctcacgagcgacgaactc

aacacgctactgatccagattgaaggatgcctcaactctagaccacttacaccactctccaatgatccat

ctgatctggaagtgctgactccaggtcacttcctcattcatcgccccatcgtatccctggccgaaccatc

gctggaaaagctgccgttcaaccgtctcgatcgctggcaaaaggtgcaggagtttgtccgtcggctatgg

aaacgttggtcaacagactacttgtccggactacagcagagaaccaagtggaccaagcagaaggacaacg

tgaagctggataccatggtgctgctgaaggaggacggtctacctccatcgaaatggtgtcttggccgcgt

cacgcagatcatcaagggagctgacgacaacatccgagtggtcatcgtcaagacgaaagatggagacttc

aagcgttccatctctaaaatctgcgttcttcccaccgacgagccatccagttcatcctagttgaattaga

taattcaacgcgggggagta

>bel4-ltr_ag bel anopheles gambiae

tgttacggcaagcgtaatggtaacgcgtcacccttagtacgcctagcgtaaggtagagggcgctttcaag

aaatttacctacgtgtaaattttacttaatcataattctattacgcagtgcaaattgctgtagatcggta

tcgtgcagctcgaattcccgcaagtgcagctcgaattaccgcaagtgcagctcgaattcccgaattctgc

cgtatataagcgagtgtttttcctgaataaatttagtcaagttccagagttcaaagcaacaacatcgtgt

cttcatcatcttcaaacttcctcttcatcattaaca

>bel5-i_ag bel anopheles gambiae str. pest

ttttggtccttcgaatcgcggatcgacgattgatcatcatttagttcttcgagcaacgaattgtggtcat

tcgaagagtgtaaacaaacgaaatggcagaggcacgtaaggtaaagtcgctgcgtatccagcaaacggcc

acgcaactctctatacaggcaattcatacattttccaagtcttataacaaagaaacgcaaagagcagagg

caattattcgcaaacaaaacttacaaaaacaatatgcaaaattcattgacctgcaagatgaactgttgcc

cctagatgaaggcaatgaagcggagaatcttgcgcttcgacaaaccgtggaatcagcgtactatgatgct

gaagctaatttagctagtgctgttgaagcaaatgataaaaaaccatgtgtaccagcatctaacatcaagc

taccggacgtgaagcttcccgtcttcgatggcaagccacaaaattggtctagctttcattcgatctttgt

cgcgatgatcgatagtgcggagctgtattcaggcgtacaaaagttgtattacctgcgtacatcgctatcc

ggcccagcactccagctaatacaaagcgttccaatcagcgaagaaaactattccgtggcatggaatctgt

tgctcaatcactacaaccacccaaagagattgaagcagttgcatgtggaagcattatttgaagatgctgc

gctgaaaaaggaatgtgcaaatgaactacgcaaactgatagaaaatttcgaagccaatgtaaatgcatta

acccaattaggcgaaccaactgctcaatgggatacgcttctaatacaaatgcttagccgtcagctcgatc

cgtcaacactacgaagctggaaagaacattcggcagaaaagaaaatcgattcgtatagtgatttggttgc

gtttctgtaccgtcgagtaggagtgttagaagtgttgccatcaacgtcatcaggtaaaccacccaagcaa

cgtgtatttgcaacaaccacaatgccgaacaacaacaccaagggttgtgcttgttgcaacagagaccatc

ctgtgtacacgtgcgatgagtttaaaaaactatccttaaatgcaaagcaaaaggtcataacacagcacaa

attatgttataattgtcttcgtcctggtcatcatctacgtgactgcaaatctgccagcacctgtaagagt

tgtcacaagcgccatcatacacaattgtgttctttaccacaaccctcgcctactgtaccgtcatcagaag

aagatcaacgagatcctccgaccacgttagcatcaacatcggtcgtcgagtcgatcacatgtgcttcagc

aggtcaacataagacagtcctcctggccactgccactgtcataatcgttgacgatgaaggccacaaacac

aacgcacgagtgctgctagattcaggaagtgagagttgttttatcactgaaaatctagcccagcaaatga

aatcaacaagggagagaagcaatctatgcatctctggaatcagttccaccaacacaaccgcaaagcagag

catccgagcgacacttcgctcgcgggttgggcgatactttgccaacctgcagttcttcatactaccaaga

gtcacggggaatcttccatcgtcatcgatcgacaccacgggatggaacctgcctgacaacatctttcttg

cggaccctcacttcgattgcatcggccgaattgatgtcttgatcggcgcggaggtctttttcgacattat

gagaccagctggacgaatacttctcgggaaggatcaaccagtccttgtcaactcggagctcggatggatc

gtatcggggccagccgtaagtacactcacacctacttcacaagcttcttctattacagtcaaccatgcat

ctacaacggttcaagatgttcataaacttatggaacggttctgggmaatagaggaaggtgaaatagtcaa

cgcacaatctaatgaacatgcagcgtgcgaagaacactttcgtcgcaccgtttcacgaaattcttccgga

cgctacgtcgtgcgtcttccactgaaagaacatcttctcacaaaactagttgacaatcgcaaggcagcag

ttcgtagatttcattttttgcaatcccggctcagttctaacaacgaatttagaaacagctacagctcatt

tatcgatgaatacgctgaactggggcacatgaagcgcatttcggaggaagaatataacaacacaaaccaa

catcattattaccttccacaccatgcggtgacgcgtcaagaatcactaacaaccaagttgcgcgttgtct

ttgacgcctccagcaagacatctagcggcatatcgttgaacgatgttttgatggtagggccaacaattca

ggacgatatccgatctatcatcatgagagcacgtaaacattcgatcatgatagtcgctgatattaaaatg

atgtaccgtcaagtgctcgtagatgmtcgtgatacatcgttacaactcatcgtatggaagccaacaccgt

ccgaatcactgcaaacatacaaactgtgtaccgtcacatacggtacagctagtgcaccctatttagcaac

acgagtattgtcacaacttgccgatgacgaaggcagtagctatcctattgcagccaaggtattgaaaaaa

gatttttacgtcgatgatttacttaccggcacttccaccgcagccgaagcttccgaagtaattacacaac

taactgcgcttgtttccaaaggtggttttactctacgtaaatgggccacgaatgatgaacaagttcgcca

aaccatctcaaaagacaaactttcagaagacgagtcattttgtttcgatcgcgaccagattatcaaaact

cttggtttgcattggcatccattgaatgactccatgacgtatcgcatcaaaccatttgaagaaaaactga

tcacaaaacgctcaacgttatcgggaattgcacgattattcgatccaatcggccttatcggaccagtagt

cacgaaggcaaaaatattcatgcaatctctctggacacttaaggccagcgatggctcgatatggaactgg

gatactgagcttccagagcagctccagaaacaatggctatcatttaaaaatgagctcaacttacttaaca

caatacaaatacaacgatgcgttctactaaatgaagctactagcgtccaattacacatttttgccgacgc

atcccaaacagcatacggcgcttgtgcctacttgcgctcaactaacaaggcaggccaaatcaaaacatca

ctagtagcatctcgatcgcgggtcgcgcctcttaaatcacaaagcattcccagactagaactatgcagcg

ccctcgtagcaagtgagctatacaaatctatccagcaagctatgcagctagacgccgatatctatttttg

gcttgacagtacagtcgctctttactggattcaagcatcaccgtcgaaatggaacacctttgtcggcaat

cgtgtgtccaaaatacaacaagccactagcagttgtacatggagccacataaacggccaagaaaatcctg

cagatcatatttcacggggattaactgcaagcgaactcgtcaactgtgacctttggtggaatggcccgcc

atggctccaactagatcaagagcaatggcccaaaccccaactgtcatctcaactaccatcagaggtttca

atagaaggtcggtcgatcactactacagctgctgccaccaaaagtcccccatgcgatgcaatactgttgg

taaacgagttgctatccaagttttctgactaccacaaattgctacgagtaattgcatattgctttcgaat

gagaactaaacgtgattcttcgcaagaaactgtcgttattagcacagacgaattatggaatgctgaaatc

agaatcttgcaagtggttcaaagagatatctttgaaaaagaatggactcagctccgtcaaaacagtcctg

tttccaacaaatccagactaaaatggttccatccgtttctttgcgatgataatcttatccgcattggtgg

acgtttatcaaaatctaatcaaccatttgaaagcaaacatcaaatattgttgtcagcaggacatcctctg

gcagaaatgctgattagacacttgcataaaaaacatcatcatgccgcaccgcaactattgatcaccatcc

ttcgtcaaaagtattgggtcataggggccagatccctagctaaacgcatttgccatgaatgcgtaccttg

ttgtcgtgctcgtcctcggctgctagaacaatttatggcagaactaccaacttcacgcataacaccaagc

cgaccattctcaatagtgggagtggattattggggtcccatcggtctatcacccattcatcgccgtgcat

catctggtaaagcatttgtagctgtctttatctgtttcgctacaaaggctgttcatctcgagctcgtcgc

aaaccttaccacagccaaattcatccaagcatttcgtcgtttcgttgctcgtcgtgggttatgcagtgac

attcacagcgacaacggacggaacttcctaggcgcctccagagaactgcgagcattggtgacgagcaaac

aacatcgaattgaaatcatccaagaatgcacgtcacaaggaatgcgctggcatttcaatccaccgaaagc

gtcacactttggtgggctatgggagtcagcaattcattctgctcagaagcattttttcagggtccttggc

aaaacaactctgcctcaagatgacatggaaacgttgctttgtcaaattgaatgctgcctcaactcacgtc

cactggttcctctcagtgacgatccgtctgacttagagccactaacaccaggccattttctggtcggaag

taatctaaaggcagtccctgataacaaattagaggatataccatccaatcgtcttaaacattaccaactg

gtacaaaagcttctacagcagatttggacaagatggagcgcagaatatttggcaactcttcagcctagga

gcaaatggctcaaaccaccagtaaaaatagacgttggccaacttgttttggtcaaggacgagtcaactac

cccattgcattggccgctaggacgcatcatcaaaacacacccaggcgatgatggagtagcacgagttgtg

acattgaagacagcttctggcgaatacactcggccaattgcgaagttgtgtcttcttccagtaacttcaa

tggttcagaactaacgtctgaaggggccagta

>bel5-ltr_ag bel anopheles gambiae str. pest

tgttcgcgcaacgcgaatgttctcgcaacgcgaatgatgagaactcaacccgtttgtaaacacaacaaca

catgtgtcatttgcgcacagacggagaaacgggaagcgggtgtcaaaaaaccacatggcgatacagtagc

agcgaataaagaactctacatttttctacagcaaaaaaaatccagtgttatcacttgagttatcccacca

>bel6-i_ag bel anopheles gambiae str. pest

ttggtccttcgaacttcggatattgaccctttgtgctttggatatcggtaaaaatcgcgtcatcatgact

cgaacgcctcggacgtctcgcactccaaccgtaaagccggcgccacaacccggttgggtggatgtaattg

ttgctgtaaggccgcatgtgcacatgcgtgaccttgcgtatggcgaattgattcgcataagggacaacat

tgtgcaggcgaaggaagagggaaaggcgctgaccgcggtacagtgcaaagtgttcgggaagaaggcagat

agtgctttagaagaacacaacaaacattatggtgagatcataaaacatgacgaaagtgaagtgcatgacg

ccaagtttaaggagacagtgaaattgcatgaggaggtgatgatggaaatagaaagtgcatctgaagccct

tgcggcacagtccatttcaccaaaggcattgatacctgctcctagcgtttcgtcagtgattgtgaatgct

ccgcttcctcggccaataccttcgttcagtggcaagtatgaggagtgggcgcggtttaaaacaatattca

aggatattgttgacaaaagtagtgaagattcgcgtatcaagttgtatcatcttgaaaaggccttgagtgg

tgaggctgccaaggtaatagatgagaaaacaataaacgacggtaattatgaacgtgcgtggcaactgtta

tccgaacgttatgataacaaacggcgtatggtagacttacatattagtggacttttgaatttgaaaaaag

tgaatgaggaaagttatgtaggtttgcgcggtttggttgaatcggttgaaagtcacgtggaaaatctaaa

gtatttaggtgaaaactttaccggtctgtcgtgtgcgatggtgatacatttgatagcgaatgcgttagat

attgaaacaaagaaactatgggaagcgagtgtccctagaaatgaacttccgtgttatgaaaaaaccttgt

gcttcctaaaagaaagagtgtcggttctagaaaggtgccaaggaaatgttgcagcggggcaaaggggacg

ttttgtttcgaaagggcccagctcaagtggtgttacgcctatgagatcgaatgcagctacaaccgtgtgc

agtgtagttgtgtgtgaattgtgtaagggtgcccatgaaacgtttaagtgctctgagctgatgcgactgc

atggaaaggatcgggaaagttttattagatcgaagcgtctttgcttaatttgcctaaaaccgggacattg

gcgaaatcgttgcaaatcgcatttgaattgtaacaggtgccacggaccacacaacacggtattgcattgg

gacaacggtccagaaccggtttcaagggaacccgaacaagtgagcgcgagccagcatgtgaacaataccg

atgcggcgggaagtattgttctcttgcaaaccgtcttgctttacgcggaattaccaaacaaagaggtgtt

gttgtgtcgtgcgatgttggacagtggatcgcaaatcagttgtgtgactgaagcacttgcaacgaaatta

ggcgtaaatttggtaaacgtgaacgtaccggttaaggggattggcaacattgaatcttcggtgaagaaaa

agtgtacttttacagtgaaatctcggtgtagtgattttgctatggacgtgacatgttttgtgtatcgtga

aataacaggtagaataccctctgtgtatttcgatacgtcgaagtggaatttgccggataaatcgatgttg

gccgatccatatttcaacaatccaagttgtgtagacattttattgggaatggattgtctttcggaaataa

tggaatctggttcagtgaaattggcaaaaacacttcccatgatgacggacactcatttcggatgggtgat

aggtggacgtgttgcggaaatacacaaagagcgtgaggtgtacacaaatgttgtgacaaaagaaaatttg

gaaacaatagtgcaaaggttttgggaagttgaagatgtgacaagtgagcgttccgtgaaggtggaagacg

agatatgtgaagaacattttgtgaagacacattatcgtgaatccagtggcaggtatgttgtgcagttgcc

tttaagggaatcgataagccaattgtgttgttcgcgaagtgtagcgctgcgtaggttttatttgttggaa

acaaaactgttgaaaaatccatcgttacgtgaacaatatcaagcgtttatgagtgaatacgaggagttag

ggcattgtagagtggttgatgaaagtgaggacgatggctccgtgaaaaggtggtatctgccgcatcatgc

tgtattgaacccggccaagaacactaccaagtgccgtgtggtgttcgatgcttcggcgaaggtaaacggc

ttgtctttgaacgacgttatgatgaccggccctaaggtacaacacgacttactttccatcaagctgcgtt

ttagaatgcctcgatatgtggtaagtgccgatatatctaaaatgtttcgacagataaaggtggatccctg

cgacagcccgctacaacgagtattctggagagcttcgccgaatgaacaactgcgtgtgcttgagcttacc

acggtgacctacggaacggcagcagcaccttttctagcaacgcgaacgttgttgcagctagcgagagatg

aacgagaaggttttcccttagccagtcgcattattgaagagaatttttatgtcgacgatggattgtttgg

ggcaaatgatatcgaaactgtccatgctgcacaagtgcaactcattgaggtgtttagaaaggcgggtatg

acacttcataaatggtcagcgaatgatgaaaggcttttggaatcgataccatttgaagatcgggatgctc

taacaaaaatcggcgattgtgaagctaacgaaatcatcaaaacgttaggtttgatgtggaatccgatgaa

tgatgaatttatatttttgacaaaggtaccgtctaagggcagaacacctaccaaacgagaggttctgtct

gccattgcaaaaatatttgacccattggggcttatttctccagtggttgtgttggcaaagattttgatgc

aaaagctatggttagcaaagttggactgggatgatcaaatttctgagccgttgatagaagaatgggataa

ttttttggaagcattgccaacagaaaatcaagttcgaattcctcgacatgtagtgagtacaaatgcagtt

tcattagagatccatggatttgcggatgcttctttgaaggcatatggagcctgcgtctatataagatcaa

tagagaggaatggtgaagcgcaattgagattagtgatcagcaaatctcgggttgcccctttatcgaacgt

gacaattcctagaatggaactgcttgcagcttcattgctttgtcgtctagtgaaaaaagtactggaagca

ttaaaggattttaatttcgaaacgattaacttgtggtcggatagccaaatagttctggcatggttgaaaa

agccgatagagtgcctgaatgtttttgtacgaaatcgtgtagccgagatcaacgagaaccgagcattcat

ttggcgttatgttcggacacatcaaaaccctgcggatgttatatcaaggggtcaatcggcgtcgctactg

gtttcaaatgagatgtggtggaatggtccggagtttttacgtacttgtgagataccaaatgtttgcatcg

atgaactaaatgatgatgaaattccggaattgcgcaacgaggttatctgcaatgttatcgtaccgctgaa

agttctcccaattttggagaaatatgaatcctttagaaagacacaaagaatacttgcttacatcgtgcgg

ttcaaacaaaacacaaagcgtcgcaagggcgaacgcattaatgatccaaaccctactattcccgaattgc

gagaatcgatgcgttggatcattagagcaattcagcatcaggatctaccggaagtagtttcggccgtaaa

aaatagcaaaccgttgcaacgttatcaagaccttaacctttttttggatggtgagttactgcgggtagga

ggtcgtataagacatgcaaatctggcttttggaaacaagcatcaacttcttcttccaaatcgaaatgtga

ttacacatcgtttaattgcaacaattcatcgtgaaaatttgcacgtcgggccatctggagtgattgcgat

tcttcgtcagcaattttgggttgtcaatgcgcgatcaacggttcgaatggttctgcataaatgcattacc

tgtttccgaagcaagccaactactttggaacaacagatgggtgatcttcctagctaccgtgtaacagctg

ctcctacctttcaacgagttgggcttgactttgctgggccaattatgctgaaatccggtatacgtcgtgt

tgcggcaataaagggatacatatgtgtattcgtgtgtatggtgactaaggcgatccatttggaagcagtg

gaggacttatcaactggtgcctttctctcagctttgagacgatttgtatcaagaagaggaatacctgaag

aaatcttcagtgataatgcgacaaattttgtcggagcaaaaaatgaactacgagagctgtatgagatgtt

taggaaggaggcgacaggccaaggcattttccagttttgccaagaaaaggagatcgtatggaaaatgata

ccccctggtgcccctcactttggaggaatctgggaggcaggggtcaagagtgttaagagtgtactgaaga

agatttataaatctgcatctctaacaataactgattttagcacgcttctatgccaaattgaagccatctt

aaactcaaggccattgtatgctcactcaaatgatcctaacgatttggaatgccttactccagctcatttt

acaattgatcgtcccttgattggagttgcagaaccatcatatttagatatgcctgaaggtcgcttgaata

aatggcagagaatccaacaactgcggcagcaattctggaatagatggcaaagggagtatttgtgcgaact

gcaaactagatacaagtggaccaaaataagggacaacgtaaaggagggagcattggttctaataaaggag

gataatacgcccccgcaattgtggaagctgggacgcattgcaagggtgttcccgggcgaggacggattga

tccgggtagtggatgtgaagacaaggagtggtgagtttaaacgtcctgttcataaattagctcttcttcc

agttatagatccgtagcatcaacctggccgggagga

>bel6-ltr_ag bel anopheles gambiae str. pest

tgttccatttcgatggaacgagcgagatgcgcggtctaaggaacgggcgagaatggcggtctgaggaagg

agagagaggtgcggggtgaggagcgaacggaaagctgtccgagtggcatctgttttggcgacaattgtct

ggaacggcggcacccgcaattgttgttcgatctcatcgacaccggccagaattccgcgaaacccaccgta

gaatgcaagcgggcggcttgtgtggtcggacggaccagagaagcgaggcaagcgagttgcggctgttcgc

cggaccacgttaagggggaacagagagggaatgaagagataaggagagagtgagagaagagagggaatta

tgaaatttatttgtgaccaagtggagtgggtcagtctgatgaaaaccgcgaatgagttaagtcgcaacca

ataaagttgtgcgtacaggattaccgtgttcagtacattgcgttaggttatccgctgagatatcaca

>bel7-i_ag bel anopheles gambiae str. pest

catttgtatggtagagagatttgcccgtggtatgaaagcttccgcaaattctctagccttctacattctc

accgaagagaaattgcttcgctcctagacgaacctatcctcgctccagaggaagcactcacactaggagg

aagacagcatcgcttccgataggattgcttccgctctccacctacgcttttgcactcacgcatacggctc

cactaacacgtgtgcagctccagcgagctgcgtatgcgatagcgtgagagagcactacgagagagctcca

agggagagccctttgcgcaccgttgaaccagtgcgacatcgcacacccacaccactgataaaaatctgtg

cactatacacgcacacgaactcatcggcacattagctacgtcatgactacgaaaagaaaccttaccccgt

tcgtcgataacgacaatggtagctgccgcttatgcagccggattgatgatcccaatatggtccaatgcga

cgaatgcgatcgctggttccatatggcatgcgccaaactgagccggctgcctaaagcggacgaaccattc

ttgtgtattaaatgcacaaaagactatgctaagattaaggctccagcgtcgactggctcagaccaaaaca

cggcaattgcagccctaatcgaagcgcttaaaggtagcagcttagatacaaacacatatctcaaacgctt

gacctttgatcagctacctgatttcgatggcaaggccaaagaatggttgaaattcaagcgtgcgtatgag

gaaacgactaagcaggctaaatatacaaatgttgaaaacatgacccggcttcaacacgcccttaagggag

aggcgtataaatgcgtgcaccgtctatttctcgagccagataacgtgccggaaataattacgaagctcga

agagcaattcggcagagcagaactcgtgtacgacgagctgcttaaagacgtgcaaaacatacgtgttgaa

aatcagcacaaaatacctgatttgtccgacgcgattcaggacatgatcactaacataaaggcaataaata

tgcctatgtatttacaagaccatcgcctcgtaaatgagttggcttgtaaactgcctactgataggcatct

aaaatggatcgaatataaatccaccaatatcaaaccgggtgttcttccatcgctggaagattttggcaaa

tggttgctgcctcaagcaaaggtacttaaagaattgccaaaacggccagaaaggccaaaacactcgatga

acgttcaccagtcttcagcgcacaacacaaaaagaccgttcaatcacaaccactttggtaacgcacaaaa

caattcaccggccactaacacatacgcaagaaatgcaatagattacaccgcacagaattgcacagccgca

cgagaacgcggcccacatccatgcgaagaaacgcatcaccgtttgcacttcacagccgatgcggaggaga

attgtcatcaagaacaaaaaatactttaccaaattgttccggtcgtgttacgcaacgaagataaaacgct

gaaaacatttgcattgttagattcagggtcatcatttacgttgatcgaggaagaaacagctaatcagctt

cagttagaagggccgactgagccgattacgatgacgtggacgcagaatttgtccatacgagagaaagaaa

gtaggagagtaagttgcctagtgaaaggagagaaggagaaaaaagaacgggtcttggaaaaaatgagaac

cgtgaagaatctccagctcccgaggcaatcaatcaattgcaacgttctcagagagaaatgtcctcatctg

agaggaattgacatcagcgattacgagagcgcaaggccaactatactaattggtctcgatcatagccatc

tactaataccactagggcgcaaaatgggccgaagcgacgagccaatggcaatcaagaccaagctgggttg

gactgtcttcgggatgaacgtgaaaggacacgaagcgcctcataatcagccgatgatccactacgatgca

gatagtacgatgatatcaaaagagatcgatcgtggaaaggaaataatccagaaaccgttcgtggaaacgg

taccgaatcgagcttggtccgaggaccagcgtatcgcggaaccagatgaagaagatcgcggtatatcgat

ccccgtacttcaccatcatgaagctgtggttccagattcttctatggcacccttgccattgtgtggaaca

catccccacaacccctcaccactacacaccggtgtagatcatcttgagccccctgacatcaccacgcaac

gatttgtcgagagggaagtgcgacaaccctgcccaggcgaatcggcagcggttctgatggagatgaggat

caacccacaaccatatgtacatccaccgcttaccaacgaggaagacgagatcctgtcgagacgaggaatt

ggctccctgactacaaagactcttcgacacagggggaggccccaccgacggagggctttgctgcggcctg

cgattgggctagcgagcaacgcaggcagccagtgaaataatcgctcacttttttattttcgtaattcacg

agaatgaattacgaggggaagga

>bel7-ltr_ag bel anopheles gambiae str. pest

tgttacgttcgcgattattaaattcgtaacggacgtgtcaaattgtaaattcgcgtggtgcgcgatcacc

ttaatctagaaaccgggtacgctacgtgtaaaagcggaataaaaatgcgtcacaaagactaacatgcggc

actcacgcgtacgtacacttatgtgctaattatcctcggaaaggatacactaacaccctaaaaattgtat

gggcttccgggggtataaaagggaccgaacttttaataaacaaaccattcggattttgcaactctaagaa

acctcgtcttttttaattttgcatattcggatcaagaaagaaatctctcatccctcttcacccccttctg

cggcataggctcctcaaattacttgagagagcaactagcgggaaggtttattattggtgtcgaacggttg

agaagatcgctgttacccgagcgggtttgatttacaaggccgcatccttcgcgtggcccacagatccgtt

cgccgtcgtaaca

>bel8-i_ag bel anopheles gambiae str. pest

ttggtgccgtgaccaggattgtgccgatakcctgaatattcgcaattttttcgataaagtgctaaacacc

tttgcctttcactgcccgaccgttacactgcactttacactacactcgcgtacacgataatcgcagtttt

agtaagatgccagctcaaccgcttattttgtcgtcgagacgaacgattttgattgccattttggcccgtt

acgaagagtttcttcaaaactaccagcccgatagggattctatcgaggtggaaactcgtatggccaaatt

tgaccaaatatgcaaggatttggagaaccttcaacagcagctggaggacagtgcaaccactgctgaagag

atgacacacaatgccgcccttagggaggattttgaacggcgcttgattcgcgttcaatcggcattgaaag

cgaaatatagagaaattccgcgcagcgaagtgtcgcagcaaggagccggtaggaacccgctacaaggcat

aaaactgcccaccatcgctctgccggagtttgacggcgattatatgcaatggctgacctttagggatacc

tttgagtgtttgattcacgataatgttgatttgccgccaatacaaaaattccattatctacgcgcagccc

taaaaggtgaagcagcgcaagtgatcgaagccattacaataagcgcctccagctatgaattagcttggaa

aacactcgctgagcgatattcgaatgaatatctgctgaaaaaacgccacttgcaagcgatgttcggcatc

acaccagcgaagagggaaagtgcgacaaccctgcaccagttagtggacgagttcgagcgtcacaaaaaaa

ccctaaatcacttgggagagaaaactgacggctggagcagcatattagagcatttactttgcacaaaatt

gccctctaacacattgcgcgattgggaagaatttgcttcgaccaatgacaatccgagctacgattcgttg

attgcctttttgcaccgccgtatgcgcgtactcgagacgctattagtaaacaaacccgaaccatcaccta

tagaaacaccgataccaccaagacgcaccatttttccgcgcactgctagttttgctaccactgaccgcga

tgttaataaatgcccgttgtgcaatatgccgcacaccataacaaaatgccagcgatttaatgccatgaat

cctgcccagcggtaccgtaaggtacttgatgcccgcttgtgtttgaattgtctgcgagacaatcaccgtg

cccgcgattgctcgtcacagtacaagtgtcgtcattgcaacttggcgcatcacacaatgattcacactga

aagcactcccagcacatcttccactacatttccaatgctagctgcgcaagatgaaccttcacacacaaca

cacgccactgatgatcacacggctagcatacaacgcagctacgcagctgcaataaaacaatcaccttcac

aaatattattacaaactgcacttctaaatgtaaccgatgcacacggcatcctgcatcctgtgcgtgcact

cttagacagcgcatcacagcccaatttgatgagcaatcgccttgctcagaggttggctttgaaaggtagc

acggttaacataaccctcaaaggagcaggactatccaccagaacggtgaggaggtcggttcgagctcaaa

ttgcttcacgtgttgaacactttgacttggatgtcgattttctgatagtagacaaggtgatcgctgatct

gccggcgcatgatgtttccactcgcggctggaacattccttcggaatttgttttggctgacccgcagttc

gataaatcagccccgattgatctcatccttggtgcccgtcattacgcttccttctttacgaacgtaaaat

cgcacgagcttgctccgaaccttccaactatgctgaacagcgtgtttgggtgggtcatgattggtcccac

ctctcctcagaatcctgcatctccgaccgattgcaccgccgcgtccacaatcgtctgcatggcatccctg

gaggagtctctcgaacgcttttggaagctggaagrgttaagcgtcaatgattcgtactcacctgatgagc

ggcgatgcgaaacrttgtataaagaaaccactcagcgcgacgagtcgggtcgatatattgtacgattgcc

caaacagaccgacttcacggaaaagcttggcctgtctaaaactaccgctttgagacgcttcgagctgctg

gagaggaggctagaacgcaacccacagctcaaggaagactatcatgccttcatgaaggagtatttggagc

tggggcacatgtcgctcatgaacaaagatagtggggatgaacgggcgtactacctaccgcaccatcccgt

atttaaagcctccagtaccaccacgaaagtaagggtcgtgttcgacggatctgcaaaaacaagcaccggt

tattccttgaatgacattctatgtgttggtccaatcgtgcaggacgagctgcttgatattgtgttgcgat

tccgcacctaccaaatagcacttgtgggagatatagctaaaatgtaccgacaaataytgctgcattctga

tgatcgtcgattggtgcgcatattctttcgattttcgccgcaagctccgatccaagtatatgagctcaac

accgttacatacggactagcaccttcctcgtttctggctacacgcacacttatccaactagcagatgatg

aagggactgagtatgcgcttgcacctgcagccctgaaacgaaacttttacgtggacgacttcattggtgg

tgccaataacgttcgcgaagctgttcagctgcgtaaggagttatcagcgctacttgccaaaggtgggttt

gagttgcgcaagtggacatcaaacaatctgagcgtgctctccggcttaagcaccgagtatatcggcacac

actcatcgctgcattttatacccaacgagacggtcaaagcactcggcatctcgtggaagcctgaatcgga

tgagctgtgttttgaatccaacactgaggctgatgaagccacgtcgaccaagcgatctattttgtcgagc

attgccaaaatgtacgatccgctcggattgatagcaccggtgatcgtgcgtgctaagatgctgatgcagg

agctatggctactcaaatccggctgggatgaacctgttcctaatcacatctgtaaaaaatggaaggcgat

tcagagcgactggaaaacgttatccgagtacaggactaaccgttacgctctcttaccagatgcaacagta

gaatttcacacatttaccgatgcttckgaggccgcctacggagcatgtgtctacgctcgttgtgaaaacg

cggcgggagaagtccgcatcagcctattagcttcgaagtctcgagtggcaccactgaagcgcgtcacgtt

gccgaggcttgaactaagcgcagctgtcctgggcgcccatctgcatcatcgcgtcaaggaggcaatgcag

atcgtgtgcgccgaatcgtttttctggtccgactcaacagtgacgctaaaatggattgcgtcacctccca

actcctggaagacgttcgtggcaaatcgagtagctgaggtgcaacactactctcatccaaggcaatggag

gcacgttcctggcacatccaatcctgctgacttggtttcccgaggcatgtcggcagcacacttcacgcag

aatcagctttggaataacggtccagattggcttgtgcaaccttcgtcccattggcccagctcagatccag

aaccaagcgatgaggcggacctagaaacacgccaggtgagtgccgctttagtttgtacacaaactcatcc

atggtttggcatttcttcatccttcaccagaatggtacgcatcattgcatactgcatacggtttgtgcgc

aacaccaagcagaaggcgcgatcacagcgaccgataccgcacaccaatgcatccaagacgatcacgccca

agtacgtggatgctgcaaaaactgtgctttgcagactagcccagcaagatgcattttccgcggaaatcaa

gcagctaaaaaagggagaagcattgatgaaacaatcacctttacgaaaacttaccccattcctggataca

gaagaagtaatacgggtgggaggacgattgaacttgtcgcaactaccgtatcagtccaagcatccagctg

ttctaccgaagaaccacaaattcacccgtctacttgcggaagattatcatgaagagatgaaacatgctag

tggaaggctattgctatcccgcattagagaactgtattggccactggacggacgtcgcttggtaaaaagc

attgcaagaaactgcttccgctgtattcggcaagatcccgcactcgcccggcagccggttggccagcttc

caccatcccgcatcacaccgagccgacctttttctgtaaccggagtggattacgccggtccattctactt

gaagccagcgcaccggaaggcagcagctactaagagctatctgtgcgttttcgtgtgtttcgctacgaaa

gctgtgcacttggaactcgtaggagacctcacaacggcgggattcttagcagcgctacgccgattcacat

cacgacgcggattgccagcccacatccattctgataatgggaaaaacttcgaaggcgcagaacgtgaact

gaaggagctttttgagctgttcaacgacgaacaacaccgcaacaccgtggctactagatgcgctgaccgg

ggaatcacttggcatttcaacccaccaaaggctccacacttcggcggattatgggaagcagcagtaaaga

cggcgaagcgacacctctatcgtcacctgggcaatacgcggctgtcgtacgaaggctactgcactgtgct

ccaccaaatcgaagcagcgatgaattcccgtccgctgttgcctttgtccgacgatcccaacgagctagct

gcactcacaccggcacacttccttattggcacatcgatgttcgccgtgcctgaaccggactacacccagc

tgaaatcctgcacgctagatgatcttcagaagtggcagcttttggttcagcgtttttggaagcattgggc

cactgagtatctacaagaaatgcagaaatgttatgcaagtggtggcagcaacaacagcaacatacttccc

ggcaggttagtgatcctcatggacgaatcgttacccaccactcgttggcctctcgcgcgtatcgttaaaa

tccatcccggtgaagacaagatagtacgcgtcgttacgcttaagacagctaagggaataattacgcgacc

gatcacgaaaatatgcgttttaccgctcagaactgatagcgaaaaccacgtgtagtctaggaagcaactt

tttgttgacattcgtcaaggtggggagga

>bel8-ltr_ag bel anopheles gambiae str. pest

tgttcgagcagctacgcgaatcgtgcagatttgaactgcggatcggatcacatccgcgcccgttgatgta

cacatctctatcgatcataaattaataaatagagggcgaaaaggctttttccctctcttcatcaccagca

atttcttacaacggcttcggtaattattagcagcaaaaaccaaagaaagaaaaggaaactttatccgaaa

actattcgcgacgaaaca

>bel9-i_ag bel anopheles gambiae str. pest

tttttggtccttcgaaccggatcgtgatatacggagaagaaataagtttcattctgcatcaagagtggaa

ttcggtattcgtcgtcaagtgtgcaagtcattccgtgacaattcccgccatcgcatatcgccccgcatca

agggcaacggtcggttacacgccatcattttgaatttcgcgccatcgctttgttcgttattttcgtgtga

tatcgcagtattttctttgtgcaatggataagaaaattaaagcagtgcaactgaaaaagaggatcgccct

ggagaacataaaatcgctggaacggttccaggcgaaatattcgagtgatgatgccaagcagattccggag

gtgttagaagatctggcgaaacataaggaagagtttttcaccgcagtttcgaaactggaagagcttgaag

ataaagacgaagcggtcgaagccagcataatggaacggatcgacattgaagaacgctgtcgcaagctaaa

atcatttctacgggaaagacagccaaaggaagaaggttcgctcaacgatacaacgggcttggcttcctca

acgcttgcattcggtcgaccccacgcgccaaatttacgtttgcccaaaatcgaacttccaacatttgacg

gagatcacacaaaatggctttctttccgagatcgcttcatcgcaatgatcgacgcttcagccgagcttcc

atctatcgcgaagctacaatacttactgtcatcgttgaagggggacgcggcggtacccttcgagcataca

cctttaacggcggacaactattcggttacctgggcggcgcttcttaaacggtacgacaattctcgtcttt

tgattcgcgaatactatcgcaaattgcactaccttccgggagtgcaattggtgtgcgttgacaagctcac

gcacctggtggatgaattcacccgcttcgtcaacgggttgaaaaagctgaacgaaccggttgactcgtgg

gacacacccctctcaaacatgctgctgatgaagttggatcgagagacattgttggcttgggagaaacatt

ccgtgcacttcacgacggacaaatataaggatgtgatcgacttcgtgcaagatcgtatccaaatcttgaa

atcgaccaacaacttcgtgaaggatcaagcagctagtggtatcaaggtggccggtctcattcgtcaacca

gggcaacggagattcatcgcgaatgcagctacatctcgctcggctcctgctgcatcgactgcgcacaccc

aacagccaaagtgtccattggagtgttccgaagaccacacactgcgcaactgtccagtgttcatcgccaa

ggaggtccaacagcgacgggacgtcgtcgcatcgaagcggctgtgctggaactgtttgagcagcaatcat

caggttagagcgtgcaagtcggattattcgtgtcgcacgtgtcgtgagcgtcatcacacacttctacatc

attcaccaccctatgctccacccgcaacggtaacattgtcagctcagtcgaatgaagacaatgtgtttct

ggcgacggcaaacatccagatcaaggatgactacgggaacacccatgaagcaagggcgttgttggattcg

ggatccatgtcgaatttcatcgctgaggagttcgcacggaaactgctgacgagtcgcaaaagggtcaacg

tcgctgtatcgggcatcggcaatgcagtacagcagatcaagggttccatcgtcgctaccgttcagtccaa

gacacaacccttcgcaacggagatgactttcttggttctggacacgccatccgcaaacatccctacatca

ccaacggacgtctcttcatggaaaatgccggacgtggcattggcggacagcacctttaacagtccggggc

aaatcgacatcgtcatcggaggcgatacgttctgggagctccacaccggtcgcaagcgctctatcggtag

aggcaaaccgtggctggtcgaaacccactttggttgggttgtcaccggcaacactcatcattcgtcagtc

ggtccgcggctgtgccatctatctgcatacgacaccccactggaggagaccatgcagcggttctgggaga

gtgaaaccatagccgaggatcctgtgctatcggttgaggagaatgcttgcgagaagcatttcgcagcaac

aactgttcgcaactcaagtggaaggtatgtcgttagtttgccatttaactccaaccctaatatcgtttta

ggagagtcgaaggaaatagccgatcgcagactgcgttgtatcgaacggcggttgaacaccaatgctaaaa

tgaaagaagagtatgtgaaatttatgaaagaatatgagcatttggggcatatgaagcggcttaccagtcc

tgcaaacgattcggtagagcattactacctcccacatcacgctgtcattaaggaatcaagcacaaccacg

aaggtgcgtgtcgtgttcgatgcatcctgtaagacttcgagtggttactcattgaacgacaaactcttag

tgggaccagtcgttcaagaagatcttttatcgattatccttcggtttcgttctcgtgccattgctctcac

tgcagacgtagagaagatgtatcggcaaattttacatagccctcatgaccgtaactatctgcgcatccgg

tacagagaacatcctgcagatcctatatcgacatttgagctacagacggttacgtacggcacagcctctg

ctccatttttggcaaccaggaccctaaaacagattgctcttgaccacaaggaagagtatcctttggcaat

gaacgcggtcatgaacgatttttacgtagatgatttgctaacgggtaccgatgatttgtccgaagcaatc

gttatacaaaggcaaatctcagacatgctaaattcagctggtttcacgctgaagaaatgggcatcgaacc

gctccgaagcattgaagaacgttccttcagaagatgtggcggtacaactctcgcacgagtggaagagctc

gaaacaagtatccacactaggcatcgtttgggaaccggcaactgatacactacggtttcgtattgagata

ccacctacaacacccagcatgacgaaaaggttaattttgtcatatatcgccaagatatttgatcccctcg

ggctactgggcccaacgatcatcatcgcaaagatgttcatgcagcaactatgggctctcaagattcatgg

aaaggcatatgactgggacagcgagctaccatcgcacttacagcatgaatggtcgaaatttcactctaca

ttatcttcactacgcaatttgacagtcccacggtacatatcgcaatgcacggcaacaagtctgcaaattc

atatctttgctgacgcatcacaactagcatatggtgcttgttgctacattcgggctgaaagcatggaagg

agtcaccgtgcagctgctaacagccaagtcaaaggtcgttgcgttatccaattcacattccatagctcga

ttggaattatgtgcagcacgactagccacacttctttacgagaaagtccagcaatcactgaaaatttctg

ctaccaccatctgttggaccgattccatgactgtccttcactggctgaattcagcaccaaatcgatggaa

gcccttcgttgcaaacagggttgcaaaaattcagcacacggctggaatacaatgctggaagcatgttcca

ggctcggacaatccccaagcagacgacatttcgcgaggtttaacgccggaaaagttgctagtgtgtgagc

gctggtggcacgggccacattggttagcacgcaactcggaagaatggccacagaacacaccatcaccaag

cgaagatgagagcgcagaagaagaaaaactatcgtcacgggttgcaagcacagcattaatctgcgaattt

cgaaacagtttgttctcacgattttcgatctaccacaaactgcaaagagttgttgcacattgtttgcgct

ttatacaaaacgcaaagcgccgcgtaggaaacaaggtccatgctaaggatatcccaccgctcactgtaga

cgaactcaaggcggcagaactcaagttgtgttatctttcgcaacaagacaccttttccgaggagatacaa

cacctgcagaagggcaaagagattccgaagaactccaaactgaaatggatttcccctttcatagatacgc

aaggtattctgcgcattggtggccggctcagtaacgcacatctgtcggaatcagaaaaacacccggtaat

attatcatcgaaacatccactgtccgcactactagctgtttcgatacacttgagtaagctgcatgctgca

ccacaactgcttttaacaacactacgccaaagcttttggataattggcggtcgcaatttatgcaagtctg

tgtaccacagttgccacgcatgttttaaggccaaacccacacttattaagcaaagtatcgccgatttgcc

aacatcacgagtcacaccaacaagaccattctcagtatgcggagtagactattgcggaccaatctatata

aaacaaaccatacgcaacagaagtccgattaaagcatacatcgccatatttgtatgtttttcaacaagag

cggtacatatcgaactggttggcgatttaacatcaacagcatttatcaatgcacttcgtcgtttgattgc

acgtcgtggtcaaatcagtgaactgcattccgacaatgcaaccacctttaagggagcggcacatgagctg

aatcgcgtctacaagatgctaaagagcgacgaacacgatcgagctgctatatttgattggtgcgcgatga

atcatatgaagtggaagtttatcccaccaagagcaccacattttggaggtttatgggaggcggcggtgaa

ggcagctaaaaagcatatagtcagaacaataggaacaacaagcatcacacaggagagcatgcttacccta

cttgcccaggtagagcaatgtttgaattcgcgaccaattacacctctatccgatgagccgtcggacttgg

aaccattgacaccgggacacttcctcgtcggtggcaatctgcaagcggtaccaatcatcgattacaccga

gacaccgagcaactatttgagggaataccagttggtacaaaaacatctgcaaaccatttgggctcgatgg

tatccggagtacctgcagcagttacaagctcgagccaaatattgcaacgggaaatcagcggttctgaaag

aaaatacactggtgattattaaggaagacaatgtacatcctacctcgtggccgatggggcgcatcgttgc

agtacaccctggaaaggacgatgttgttcgcgtcgttacactgcgcactgcttcagggaagcaaatcgtc

cgcgcagctaatcgtctggcggttttgcctaatccggacgtaattagcaacttagagcagaaggaaacca

ctggcactgagtaacgcgcagctgcaagccacgcgacattgtttacatttcgcactcatggcagaacaag

atacacgcaacacacattcacacatctacacacacgagcagtatgataggagataagcgtcaggttcaag

agtcgaactgtttttgaattcttttttgaactcatttttggatttatattttgcatgaaatttaaagaag

ttcttctttggtggccgggaa

>bel9-ltr_ag bel anopheles gambiae str. pest

tgttggaatttggataattattttcgaacttaaatttgaattgtacataaaacaaagcgttaggaaacta

agattaggttataaactactcgacgttagaaactgctcgatctacacgaattatgaactgcatgaaatat

tatatacaaaaaaggacaaacgaatatacagttgcaaaccgaccagcgataaaggtgtacactttcctat

tcaaatttcctccaaatatcacagccagccccttttcgtgaatatatttcaca

>copia-6_ag-i copia anopheles gambiae str. pest

aggttatgggcccagaacccagaagcgtacagtgattgaagcagaatcttttcgccgttattccgaagaa

gttttaagatgaacccgaactccaccggatcctcaagcgctgcaggtagtagcatttcaacatcaagtct

tcctggcatagaacgactcatcgggagagaaaattgggaaacatggaagtttgccgtgcaaacgttcctg

gaacttgaagatctctggtgtgcagtaaagccgaagaaaaacgacgatggaagctacgaatccgtcgata

cagcaaaggatcgaaaggcacgagcgaaaatcatcttacttcttgaaccagtgaactacgttcacgtgaa

ggaagcgacaacagcgaaagaagtttggtccaaactagaaaaggctttcgaagactctggcctcacaaga

cgagtcggattgttgcataaattaatcaagacagatctagaatcatgcgattctatgtcggattatgtta

atcgtattgtatcaacggcgcatcaactgaatggaattggtttcccgatttcagaggagtgggtcggaaa

tctattactggctggattaacagaacagtatcgcccaatgatcatggcccttgaaaactccggtattgtc

atcactggggacatcattaaaacgaaacttctacaagaggttcctcccacatcagttgaacctgcgtttg

cggcaaggacgaagcacgttaatgctggtaagcaaactaaaaaatcgaatacagctaagggaccgaaatg

tcgaaaatgttcgaaatttggccatatagcgaaggattgctacagcacgaagggaaacgattcgttctgt

gtagtgctttctacgtgtggatcgaaggaatacggaaaatggtatttcgattccggagcaagtgtccaca

tgacgaacaatagcgattttttgatgcatgcgaaaacatctagtggaactgtggtagcagccaacgggga

gaacatgcaaatcaccgcgaagggatcctgcgttttgaagccttcgtgccaaaaaggtgaaattcccgtt

gatgacgtgcagctaatcccgaatctatccgtgaaccttttatcggtaaatcaaattgtgaaaaaaggct

actccgttacgttcaccaatgaaggatgcgaagtggttaaccgaaacggcgatatcattgctactggtag

ccatgacaacgatctgttcaagcttgacgaacgtaaagaaggtgagaaaaccgcgttgacagtttcttca

acagggagcttggaactatggcatcaaaggatgggccatcttaacatcaacggtgtccgaagccttgcaa

atggaatagtgactggcgtcaatattgttggagataccatggccgattgcaaagaatgtccaatgggcaa

acatagccgtcatccttttagcaagataggatcgcgggcggctgaaatactcgaattggttcattctgac

atttgcgggccgatggaagtcaaatctctaggaggaagccgatattatattgtatttgtggatgacaaat

cacgccggatgtggacatatttcttgaaatccaagtcggaagctgaggtaaacaaaattttccaggattt

tcacaagatggtagaacggcaatctggacgaaaattgaaggtactcagaacagacaatggaaaagaatat

gtcaacacagggttcacaaactacttaaagaagcacggcattgttcatcaaacatccaacgcatacactc

cggaacagaatggcatggccgaacgagcgaataggtcgattgtggagcgtgcaaggtgcatgttacacat

ggcgaaactttctaaaagtttttgggcggaagctgtggctgctgctgtgtaccttctgaatcgttctcca

accaaaggccataatgttactccgcaggaagcgtggtctggtaagaaacctaacttgtcccatattcgga

tctttggtactagagcgatgaaatttattccgaagcaatatcgcaagaagtgggacgctaaatcagaaga

gtgcattctgaccggtttcgatgagtttaccaaagggtatagattgtacaacatcagatcgaagaaagta

acagtcagtcgtgaagtaaatttcattaatgaaggtgttgctgttcctatccaagaaaaatcaagcagaa

ggcatatgattctccttgaacatgaagagacagtttctctttcaccgaacgctacatcacaaatggaggc

agttttgagtgaaaatgaggacgaggacagcgacagcgaatacttcacggacgcgaacaatgaaacttct

gaagataccgatggaactactaacgaatttgacgaaacagtggtcgataatgatgcagctgtgaacagcg

aacctctcgttataccaactcaatcgcaaatcctgaggcgaagcagtcggacgcgtaaagtcccagagag

gtataatgattctataatcccacatggctctggtcttttcagcaatgttaccagttcaaagatcatgagc

agcaattcaagcgaggatccaatcacacaccaggatgcaatgtcgcgtagcgattcagaacgttggaaag

tagcgatgcaagaagaatatcaagcgttgattgacaacagcacatggagattgacaactcttccagaagg

taggaaagcaatcaaatgtaaatgggtgtttaaaacaaaacacgatgcggctggaaaagtcaaccgctac

aaggcgcgcttggtgataaagggatattctcagcgaaagggggtagattataacgaaacatattcacccg

tagttcgtcatagttccctgagatatttatttgcactagcggccagaaataatctcttggtggatcagat

ggatgcgataactgcttttctacaaggagatttggaagaggagatatacatggagcaaccaccgtgtttt

gagcagcctggcaagcaaaacatggtatgtcgattgaacaaagcgttgtacggactaaaacaatcaagtc

gtgtctggaatacgaagctagatgcagcactgaaacaactgggtttggaacaatcgaagtatgatccatg

tttatatttctataatggcaatggaaatatgctgtttgtagccatttatgtggacgatttaatgattttt

agtaataatgaagaaatgaagaatcagctgaagacgaaattgagcagcatgttccggatgaaggatttgg

gaccagctaaacattgtttagggattcgtgtgaattatttaaatgacggaattgcacttgaccaggaagc

ctacatagaaactatcctatcccggttcaaaatgcaagactgcaaagctgttgctactcctatgaactct

tccataaagctaactaaggaaatgtcgccacagacagaagaagaaaaggaagagatgtcagcggtgcctt

ttcaagaggctgtaggttgcctgatgtacctagctcaatgcaccagaccagatatcctgtttgcagttaa

tcagctgagccgatacaacaataatcctggatcgcgtcactggcaagctgtaaaacatcttatgcgatat

ctaagaggaacggcatcgatgaaactcaaatattacagaaaaggtaacgaacaaataactggatattcag

atgctgattgggccgctgatacagaagataggaaatccaccagtggatatattttcttgatgcaaggagg

agcggtgtcatggtgttgcaaacgacaaccaactgttgcattatcaacctgcgaggcggaatacatggca

ttgtcagcagcggtacaagaagcatcgtggtggaaaggattgttagaacaatttggtaagaagcaatcga

ttcagatattttgtgataatcagagcactatctgtattgcaaaaaatggaggatatacaccacgaacgaa

gcatatcgatataagacatcattacatcagggatgctttggatcgaaatgttgtgaatctccattacatt

aacactgaagaacaagttgcagatggtcttacaaaagcattacaacgaatcaaacaagaacgtaatcgac

gatctatgggaattacacaacaatcggcttaaggaggag

>copia-6_ag-ltr copia anopheles gambiae str. pest

tattggaattatagtaagccgtatttgggttatgagtaatttaatcatagtaacctatgaatttgtgtag

ttataagttttggtatgaaatacaattcattctgtttctaaccgtacaccaagctggttgacttcactac

ttccaata

>copia-7_ag-i copia anopheles gambiae str. pest

ggttatgggctcctctgtaaaggaaggtgctggcattccccagctgaatggaacgaactacggaaaatgg

cgttttcgagttcgtttgttcttggaagcttcggaagtttgggaggcgctcgaagaagacgtgcctgagg

cggtcggagaaccgcggaacaagtttttgcgaatggaccggaaggcaaaatccttgctggttggatttgt

tggagacgattgtctagccatagtcgaagaaaagggaacagccaaggaaatgtggaaagcccttgaagac

acttttgcgaagaagtcgggggcaagccagacgatcttgcgcaaacgactggccacgttacgcatgaagg

aagggtgttctatgcgaagtcattttgccgaattcgacgagctagtgcgacagttgaaaaatgcaggtgc

gaaaatgcaagagaacgatttggtatcgcaacttttttttaacgttgccggatagctacgatcctcttgt

gacagcccttgaaaatatccaagataaagacctctcattagaaatggtgaaacatcggttactaggagaa

gagtctaagcgagtcgacagggtggactactatgtcgaagaaaattcaaccgcttttatcggtggaagta

atcagatgaagaaattcaaaggaagatgttaccggtgtggaaaattaggccacatgcaaaaggattgccg

atccaagatggaaaacagaaatgctaactctgttgtggcaggcaaaactgtgagtttcatggtgaaacct

cagtgtgaagtggaagaaaaagagcaagcaatacattcatttgtaatcgattccggatgcagtgaccact

tcatcaacaacatcaaataccttcaaaacattcgaaaattgaaagaaccgtttatcgttgatgttgccaa

agacggtgtaactttagtcggagaatacgaaggtaccgtgcgtggaaaaacgaaagaaggcgttatttta

gaaatgaagaatgttatttatttacctgagttgagaagtaacttagtttcagtaaagaaaatgacgcatg

ctggaatcgatgtgcttttcactcgtgaagatggctttgaaaaagccctaatgaagctagagaaggatgt

aattggcgttgctcatatgaaacaaaatctttatgagctagagttgcagttagaaacgagacgatctgca

aatatgtgtatgacagcggtgagtacactaaagcctcgtaatgaatattttcgatttcaagctacaggca

tggtgatgcaaaactgtatggtagacggtcgcgatcgaaaatcaacacatttttgcgacgcatgccgaga

accatataaaaaaaatcaagaattggcctttatctcattggataacgacacttgtggttctgaaaatcga

gatgtaaaattcccttttcatgaacagcgggacaatgaccgttttcagcaagagggggaaatggtcggaa

aagataatgctgccaacggtttgcaagtgcatcaaaagttagtcgatattgaagagaataatgaggaata

ttttgaaatattaagcgataaaggaatacgtgaaaaagaaaatggcgaaagggaagaaccgataacttca

aaactcagcgcaaacgtaagtcattataattgtgaaattaggaagaaagctatcaaaggtgaatcgttaa

catcaaggcctgattttaaagtagcatttcttcatggaaaactgcctgaagctcctcctggtttgttgag

gcaattcttgggcttgttacaggatgttggattgaaagtaatgacattgattcgaacccttccgaagcta

cataccaataaggcattagaagctggttgattgaaagtcttgaggaagaaacttgggatgaacgatcgag

aggggg

>copia-7_ag-ltr copia anopheles gambiae str. pest

tgtaaacgaagtagccgcgtgtaatcgaagtagtctttaatagatttgggaacgatcgtttcattacaag

ttagtcgcaaataaaccattgacgagaacggtcgcattaaagagaaacttgttaaaataaaaaacttcca

aagtatttcaaagaacagagttccttta

>copia-8_ag-i copia anopheles gambiae

ttaggttatgggctctccacgtctctaactagtgtgtcgcggatataaaagggattcattgaagataatt

ttattgttcgtggtcgtgttcattccggtcgatattatcgaagtactctggaaaaaccaacatggaggaa

gaacgaatgcaacgcgtgtatctttttgatggaacgaatttttcgaattggagttttcgtatggaattat

acctggaagaattggggttactccattgtatcgaaaagactctggaagaagaagatttttttcaagtgaa

tccgacgacagttattgaagcgctaaatgaagaaaaacggctaaaacggaagcaacaagatgcgaagtgt

aaatcggttttaattcataaaatagccgataatcaactggaatatgttcgaggaaaaacatctccaagag

caatttggttgacgttaaaggaaaatttcgagaagaaaggagtttccggagtgttttacttgctgaaaca

gctatctacgatgaagttcgatgaaaagcgaacattgcaagaacatattttatcatttgaaaagctcgtc

agggaacttgaatcggctaacatcaagttggataagtcggtattagtattctttttgctacaatcgatgc

cgaaatcatttgaacatctgattacggtattggaaacgttgccggttgaacagtgctcgatggaatttgt

caaagcacgtttgctgaatgaagccgttaaacgacaattcaacgtggaaacagtcgaaaccagcacagcc

ttttctggaagagttggaaaacctggaaatactggaagatttgttttcaaatgtcatgcttgcggaaaac

ctggtcataagcgtataaattgtccggaaaacaaaggaaatgaaaggcacgtaaaagcggaaaatcagaa

gaagaaaagtacggcacattatgcggaaaaccaagacgacacggcgtttataactggtgatgtgaattta

gatgaaaatttccaatggatactcgatagtggtgcatcggaacatatggtaaacaacaaaaagtatctgc

agaatattcgaaaactgaattcaccgattgttataaatgtggccaagtcgggagtatcactaaccagtga

tgtaattggagatttgaagatatttgtgaaaattcaagacgagcagttgccatatacagtacacgatgta

ttgtatgtacctggactttttgcgaatttgttctccgtgaaacgagtcgttgaacgaggaatggaagtaa

aattcagcgaaaatggtgcaaaaatcatgcgtggatctaaggttttgtgcactgcaagtcgaaaaggtcg

attatatgaattggatattttaacaccaaatatcgaatctgccatggttactgatcctcaagatttgctg

acaatatggcatcgtcggtatggtcatattggaaatacgggtctagtgaagctcatccaagctgaaatgg

ttgaaggaatcgacaactgtggaaatgtgaaaccgcacgcaggagtctgtgaatcgtgtatgatgggaaa

acaaacaaggctaccatttgaaacagttcctcgacctcgatcatcgcgaccattggaattgatccattcc

gatgtttgtggaccgttcacaccagcttcctgggatggtaagaagatgttcgttacctttattgatgatt

acacgcattttacagcagtgtacgtgctgaaatcaaaggcggaagttttcgacgcttttgttaagtacac

agcgatggctacagctcactttgatcgtcgaatatcccgagtaagatgcgataatggccgtgaatatatc

aactcaaattttacacatttttgtgaggaaaagggcattgtaatggaaccaaccgttccatatacaccgc

agcaaaacggagttgctgaacgcatgaacaggacgattatggaacgtgcacgagctattttggacgattc

gaaattcaaacgatcgatgtggaatgaagcggttttgacggcagtacatctgatcaatcgaagtccgtcc

tcatctttaagtatgagcaaaactccatacgaattgtggttcgggcacaagccaaatgtgtctcgtttca

gaatattcggaagcaaggtattttgtcacataccgaaagagaagcgaacgaagcttgatgtcaagagtca

agtaggatttcttgttggctacggaatcaatggctatcgtgtgtgggatcctgtacatcggaagattatc

gttgctcgtgatgttgtaatcgaagaattaatgtcgaatcgtcgtttggaagaatccacttttgaccaag

aaaggatgctaccagaacaggttagtgaaacaaatcgtaatattgaatttactgttcgacatctggaaga

ttttggcgattatttgaacacatcgactgagggcattaaaaacgtgaaaaatacaaatattgaaactacg

aatagatcggctgacattgttggaaatatggaagaaacatgtcatgaaaactcgaatgatacgcctgatg

ttattgacaacacagaacataacgaagtagcagttcgtcgtagtgaaaggcttcgaaaaccacctgtgcg

ctttagtgattatgaagctaatgtcgcatttgcactgaacgcggaaaattacgtggaagatttacccgat

acgattgatgcacttcggaagcgtgacgattggcctgaatggaaacaagcgatcaatgaggagatgcaag

cgctcgagaaaaatgaaacatgggatctagtagaacttcctgttggcgcgagagcagttccttgtaaatg

ggtattcaaaatcaaatactcggagaatggtactgtgaacagatacaaggcgcggctcgttgctaaagga

tgttctcaacgtcaaggatatgactatcaagaaacgtatgctccagtagtccgaataacaacagtgagaa

ccctactagcagtagcagtgcagaagaagttctatcttcatcagatggatgtgcggaccgcctttctgaa

cggaaatctttcagaaacagtgtacatgctacaaccaccaggatttgagagggggaagaaggtatgtaaa

ctgaacaaatccttgtatggtttgaaacaggcgccgcggagctggaatgaaatgttccacaattatatgt

tgacactggaatttgtacggtcagcatatgacagttgtttgtacactcggaaatctgcgaaagttgagat

gtatctaatcctatacgtcgatgatatagttttggcatcaaactctttggaggaaattcagctggtaaag

gaacaattaaaaaggaaattcgaaatggacgatatgcaagaaataagcaacttccttggaatgaaaattg

gttacgacatggctcgaagtacgttaaaaattaatcaatcaaagtacgtgaaggacttgttaaaaatatt

tggtatggaagattgcaaaccatcgttagtacccctagaaaccaatttgaagttaacacgcaaccataac

atggagggaacaacacagcatccgtaccgtgaacttgtaggatgtttaacctatttgatgattacgtctc

gtccagacattagtattgcagttaattatcttagtcgctttcagagtggtgcaacagatgaacactggac

acatctgaaacgtgtactaaggtatttacaaggcacaaaggattatttcctagaatatcgacataatcaa

gaggagccaattgtaggatttgctgatgcagattggggtagcgacatggaaagtcgtcattccacaagtg

gctacatatttcaaatttttggaaacactgttctatggaccacaagaaagcaaggaaccatagcattgtc

atcaacagaagcggaatatgttgcgttaagccaatcatcctgcgaagcaatgtggttagaaaatctgctt

accgaatttggcgtgcatttgaatagtccgttaattatatacgaagacaaccagtcgtgtatgtatattg

ccgaagagcctcgcgaacaaaaacggatgaaacatttagacatcagatacaacttcatacgagaatgtat

ccagaacggaagaatccaactgaagtatattccaagcgaaaaccaacttgcggatatgtttacgaaaggg

ctttcttcagcagtttttacgaaacatcgatttgcgataggtctaagaggggg

>copia-8_ag-ltr copia anopheles gambiae

tgttggaaaaacaaagtagaccatagcatatacgacgggtataaaagaagcagctagcttcagttatgtg

gtagtttagtataataaactgtagagacacattctaaactca

>copia1-i_ag copia anopheles gambiae str. pest

ggttatgggcccagaagtactgtcgaacaagtgttaagtttgtgttatattgaagtaataatctatcgaa

aattttaatcttgaagaaatactgaaaacttatcgaaaattttaatcttgaagaaacactgaaaacttaa

atctagaaacatgtctgaatcacacgtcaccattgaaaaattaaacgatcaaaattacgcaatatggaaa

ttcaagatggaacttttgttagcaagggaaaaggtgctgactgtcgtgaaagattcgaaaccagcaagtc

ccgacgctgcatggattgcgaatgatgaacgtgctagggcactgatcggtctgtcgttggacgacagcca

actcatccatgtcatgcaaacgagttcatcgaaagatatgtgggatgccctaaaaggctatcatgagcgt

tcatctttgtccagcaaaatacacgtcatgcgaaaaatgtttgccacaaaaatgactgaaggtggagaca

tttctaaccatctcaaagaactatgttctctgcgacttcgtttaattgcgctgggagaagaaatgaaaga

tccatcctttgtcgcgttaatgttgtccagtttgccaaaatcctttgatggtttgatcgtggctttggaa

agtaggcctgatgaagatcttacggtggattatgtaaaaggcaaattgttggatgaaggaagacgtcgag

cagatggtgcagatgaagataaagcgttactatctggaggaaagaayawcacgaaattttggaaggacag

gaaactaacaaccaacaaggaaaaacagtgccattattgcaagaagaatgggcacataagaaaagactgt

agaaaatgggctgcagacaaaagaagtaaactagatggtgaaagcgtcaacgttgctaatgaagacaatc

gagaggaagtatgtttgttcattggagaargaaacgaaactggaccatggtgtttcgattctggtgcaac

ttctcatatgacgaacgatacgtctattttgaaattaatagataaatcgaagcaatcctcgatttcatta

gcgaacggagattccatcaagtcagctggtgtcggaarctgcaaattgttttccatggatggaaacggaa

aacgcaagaaagtttccttggacaakgtgtgtcatgtaccatctttgacgacaaacctattatctgtaag

taaaattaccgataatggattcgaartgyttttcgataggtwtggatgtcgtgtcctgaaaggaaaacaa

gtattgctgattggtgaacgtaaaggtggtctgtattatctaaaacagactgaacaagccatgttggtag

ataaaaaccatgaagcttcctgtatacacctatggcatcgtcgatttggccatcgtgacatagaagccat

aatgaagattgcgcggaacaatttgggaagcggcttgaacatcaaccgatgtcatgtgaaatccatttgt

ggatcatgctgtgaagggaagatgagccgtgatcctttcccaaattcttcatcttcaaggacatctggtg

ttggcgaactgatacatacggacttgggaggaccgtttgaagtatcaacagcccgaggaagccgatattt

tatgactatggttgatgattttagtcggtatacaattatctacctgktgcaaaacaagtgtgagacagaa

aaccggatcagagaatattgcrccatgatgaaaacacagtttggacactatccgaaagtcatcagatcgg

atggtggtggtgaatacaggagcaattctttaaaggaattttttgtagatcacggsatcgtgcatcaaca

aactgccccatattctccacaacaaaacggcgtggctgaacgtaagaaccggtacctcgttgaaatgatg

agatgcatgttggcagaatcgaacatggacaaggtgttctggggtgaagcgatcaccactgccaattatt

tacaaaatcgcttgccatcctccttactggaatcgacaccttacgaaatgtggcacggaaagaatccttc

gtatgaacatcttcgagtatttggttcagaagctttcgtacacattcctaaagaaaaacggcgtaagttg

gataaaaaggctgaaaagttggtattcgtcggatacgcggacaatcagaaggcctatcgattcgtaaacc

tggagacgaaaacaattaccattagtcgtgacgcaaaatttttagaacaatgcgagattgagaaaattgg

aacaaaaccgaaaccaacgacatcaggaggagtagtggtactgccacttggatcaactccttcattatgt

cgcgcagaagaaactaccacgagagaaaacatcgttcaaatggaggcttctgctgaatcctcctgcatta

gggaatcgaacatgaacgatacygtagatgawcttgatgttacaccatacaacagtgcatctgatggcga

actatcagatgaaccaggggctattgaaatgcatcaaagtgtacgtaggtccaygcgaacaacaaaaggc

atcgcacctgttcgattcagggaggaaagttatatggcgggatcttctgaacaaaacgaagaacccagaa

atttgaaagaagttttygtctgtgcagcgcgcgaaaaatggatatcggcaatggaaaatgaactgaaatc

acacgaagaaaacggaacatgggatgcattggtagagctacctgctggcagaaaggttgttggttgccgc

tggatttttaagttgaaaagaaatgcagctggacaagtaatcaaacacaaagctcgtctagtggcgcaag

gctattcccagcaatttggcgaagactatgatcaagtatttgctccggtcacaagccatacaacatttcg

tttgatgctcgctatagcttctaaaacacagatgaaattacggcatttagatattaaaacggcctattta

tatggtgatctagatcaggagctttttatgcgacaaccacctggatacgagayaaaaggcaaagagcatt

tggtttgtcgattgaaaaagagtatttatggtctgaaacaatcagctcgatgttggaaccagaaactgca

cggtgttctgctagagattggcttccaacaaagtgctgctgatcagtgtctgtacattaaaactgaagat

ggaaaaagagtctacattttagtgtacgtggatgatatgatagtcggttgtgtggacgagactctcattg

attctgtgtatcacgctttaaccgaacatttcgaaatgacggacctgggaccagttagttactttctggg

aatggaggttaaatrtgaaaaaggtaactacagcgttagcctcgaaggttacattgaaaaattgattcgt

aagttcggattgagcgaagcaaaaactgcgaaaacaccgatggatgaaggatttttgaagcagcaagact

caagctctattttgaaagactctactcaatatagaagtctagttggtgctcttctatacatatcggtgtg

tacgcgaccagatattgctgtaagtacgggaatacttggtcgtaatgttagtaatcctactgaatcatgc

tgggttgcggctaagcgtgttgtaagatatttaaaagcaactaaacattttaagctcactttcaacaaag

ctggtagcgatttgattggttattctgatgctgactgggcaggtgatactataacaagaaaatcgacttc

cggatatgtgtttttctatgctagtggagctgtgtcatgggccagtcgcaaacaaaccagcattgcatta

tcatcgatggaatcagaatatatttccttaagtgaagctactcaagaacaaatgtggcttactcgattga

tgaaagacttaggagaacatattgaaaaccccgttaaaatctttgaggataaccagagttgcatttgttt

cgtcaactctgatagaaccaatcgtcgatcgaaacacattgaaacaaaagaacactttgtcaaacaacag

tgtgaatctagaaaaatgatgcttgaatattgtcccacggaagagatggttgcggacattctaacgaaac

cactaggagcaacaaaacaaagaaaatttacggagatgttagggcttcatggcacacgttgaggaggag

>copia1-ltr_ag copia anopheles gambiae str. pest

tgttgagaaagcaacatgtgtgcaatgaacacatcatagtagtctttagaaaatgtttaatattgtcagt

ctagtttaaaatacattacatacaagttcattgtttagtttctgctcctcttttattccactgtgttata

actacgctcaaca

>copia2-i_ag copia anopheles gambiae str. pest

ggttatgggcccagctctgtgtggccagttcaattgaaaagtgcgcgacgcggttcggaaagacagttat

tttttcggtgtgaaaaaattaggacatttccggaaggtacgcagtgcggttaaggaatcgtgtgtttttt

cgtggtgaaagaaaaaacccaaccgggaaggtttttgcacgggcaaaaatggatttttcgaaagtgggcg

tcatccggcggaacaaccgaaactatcggtcgtgggctttcaaagtgcagatgttgatgatgcgggaggg

tacgtggacgtacgttgacccgggtgtcgcgccgacaccggtaactccggagtggacggagggtgattcg

aaggcgcgggcgaccattgctttgttggttgaggataaccaacacaatctcatcatgacaaagaacacag

cgaaagagacatgggatgcgctcaaggcacaccaccacaaagccactcttaccgggaaagtttcgttgct

gaaagagatttgcaacgcaaactatcgtgaaggtgagaatatggaagattttttatacggcatggaggat

cattattctcggctggagaattcgggtgaaaaactctcggcgaacatgcaggtggccatgattttgcgga

gccttccaaaagcatttgacgcacttaccacagctttggaaagtcgttcagataaagagctaacgatgga

tcttgagcgggcaaagctgatcgacgaaagtgagaagctgtacggcggaaaggtgcaggaggagcgagtg

ctgaaggcgaaaagtgaagcaaaaccaggcgcgtgtttcttttgtggtcaacctggccataagaaacgag

aatgcaaagagttcctgaatcggaagagcagcggggaaggtgaaaagaagaaaaagattaagccgaataa

agaacaacaagtgaaaacagtgcgcgaaaacgacgcaagttcgttcacgttcatggttcgtcagcctgaa

attcgcggtaacgatcggtcgtggctaatcgactcgggtgcaagttcgcacatgtgtagtgacaaaagcg

cgttcacggtaatggaacaaagcttgcgttcaaatgttaccgtcgcggatggcagcgaaaatcgcgttga

aggcgttggcgattgcctgatcaagtgtgcggttgaatacggtgaaataattgaaatcacgctacggggt

gtgttgtatgttcctacgctggaaggaaacatgatttcaatcggtaaactcgcggaaaaaggtgtgcgtg

cggtttttgacaacaccgggtgcaagctcgtttacggaaatacggtcgtcgcggtcgcggataaagtgag

cgatatgtattggttgcgaattgcacaggatcgagtgatgaaatcagtggtaaaggagcacacgaaaaac

tgccaacacacttggcatcgtcgtcttgggcacagggatccagctgtcatcggtgaaatgaagcggcgcg

atttggtgtcggggctagaagtggtcgactgcggtatccgctggacctgcgaatgctgcatcgaatgcaa

aatggcacgctcgccatttccaccagttgcggaaaaaacctcgacagaagtgctggatataatccatagt

gatgtgtgcggcccaatggaggaaacgaccttagggggatgccgttactatatgaccctaatagacgatc

atagtcggtatactttcgtctattttctcaaaaagaaatcggaggccgaggataagtttcgcgagcatgt

aaaattggttcaaaaccaatttggccggaaaccgcgaatcattcgctccgatcagggaggagaatactcc

aataaggcgcttcggaagttctgtgcggacgaagggataaagatggagtttactgcagcatattcacccc

agcaaaatggagttgcggagcggaagaaccgatcgctaacggagatgggtcggtgtatgcttcgggatgc

aggtatgcataagcgattttgggcggaagcaatcaacaccacttgctacttgcaaaatcgattgccgtct

gctgcagtagagcgtacgccattcgagatctggttcggcagaaaaccagatttgaccaacctgcgactgt

ttggatgtgttgggtacgtactgattccgtcggtgaaacgaaaaaagttagacgtcaaggcggagcgtat

gacttttgtcggctattccggcgagcataaggcgtatcggatgctaaacactcaaacgggagaaattcaa

attagtcgggatgtccgttttcttgagattgatgacggatccaaggagcagacatacggtgatcccaaaa

tagaggataatccgactgaaagcgttgaaatcgagtggtctctcgatgaaacgaaacgggaagctaaaac

taacgtggccaatgatacaatctccgaatctgaattttacggttgggattgttcagacgatggctggcca

cgaggtttttggaacgacaatgataacaattggcttcgcggactgtgggacgatgacgacgctgaagctg

gagctgcgatgccggaggcggtgctggatgctgtaccggaggcgatgccagcagctgtgccagaggtgtc

gaatactcccgttcgtcgtttacagagggtgacagctggcgttccaccggcaagatatgacgaagaagta

tatctggtgaaggaaagtgtagcagaaccaaaaacgtataaggaagctgtgtccggtcctcagagtgctg

aatggaaaatagcgatggcagaagaaatgcagtcccatcaggaaaatggaacgtgggagctagcggagct

gccgccacaccggaaggctatcgggtcgaaatggatcttcaagtgtaaggcagatgaagacggtcatttc

gttcggtataaagcacggctggtggcgcagggtttctgccagaaattcgggacggattacgacctggtgt

ttgtccccgtcgtaaagcagattactttccggacgatgctggttctggcgagtaaaaggaagatgttaac

gaagcacgttgacataaagacggcgtatctacatggtcttctcaagaaggagatttttatgcgccagcca

cagggattcgaaagcgataacccgaacgaagtatgcaggctgcatcgcagcatttacgggctcaagcagg

cagctcgtgtctggaatacgaagatcgacgacgtactgaaaactatgggtttcatccaatcaacggcgga

cccatgtttgtacatacgcgaaaaagcgggtaagtccatctttgttctcatttacgttgacgatgtgatc

gtcatatgtaacacggaggaagaattttctgaggtggtccacgtcttgacactgaatttcacgatcagcg

tcatgggtaacctaagattttttctcggcatacgaattcggcgtaacgatgggcgttactgtatggacca

acgagcttatttggaacgagttctggagcgtttcggcatgctggatgctaaaccgtccaaattcccgatg

gatcccggcttcttaaaacgaaaggaggagaatggcaggaagttggattcgccaaaagcgtatcaaagtc

tcataggagctctgttgtacgctgcagagatcagcagacccgatattgcaatcgccacagccattctggg

caggagagtgcaagatccatcagaagcagattggaacgaggccaaacggatactacgttacctcaagggt

acactggatagtgtattgtaccttggaagcggcggacaaaagctggagtgttttgtggacgccgattggg

caggcgacgagagcgaccgcaaatccaactcggggttcgtgtttaagttcggcggcgggctcatcggatg

gggctgtcataagcagaagtgtgtggcactatctagtaccgaggccgaatatgtttcccttgccgagtgt

ctacaggaggtaaagtggatactgaaactgatggcggatgttggcgagcaactggatggtccagttctgg

tcaacgaagacaatcaaagctgcattgcgctgactaaaggagaccgagccgaacgcaaagcaaagcacat

cgatacgaaatttaatttcgtggaggatatggttcgggacggcatcgtgaaactgcagtactgcccaacc

gaacacatgcaagctgatttgcttaccaaaccgttgcaagcagtgaaacttcgacaacttagggaagcga

tcggaataaaaccattcagtgttgaggaggag

>copia2-ltr_ag copia anopheles gambiae str. pest

tgttgagcttctcggattgatgcgacaagtgcctatcaggcaacaccgaatgattgccaatcgggccaca

ctttgcacaccacacacacgattgaactctgaataaagatcattcctgcattaagcgtacaagcgaacac

acgtcttttcatttggtaaaacagtccactcggtatttccactttgcgtttctcttccagttcaaca

>copia3-i_ag copia anopheles gambiae str. pest

ggttatgggcccaggattagtggcgattaaaacgttttaaccaaaagaaagatttcgcaaaatggaacga

ttgggaattgcaaaactcaacggaggtaattacagtgtctggaagacaaaggtcgaattcctcctcatcc

gagaagaattgtggcagtatgtgatcagcgatggaccgggtaacacgacggccagtcccactaccgaagc

catcggagcagtatggaagagtggtgatcagaaggcgcgagcgacaatcggccttttactggaagataac

caactcaatctaattaaggactgtaagacggctaaagcgacatgggaaaagctgcgaggacactacgaaa

aggccacactaacttcgaaggtgtcgattttaaaaaatatatgtgagaagcgtttttccgacggtgagga

tatcgagcagcatatcttcgagatggaagaattgttcgataggctaacattgaccggtgaggagctaagc

aagagcctgcaagtggcaatggtgctccgaagtctcccgcaatctttctcggttctgaccacagcgttgg

aaagcaggtctgacgacgagcttacgctggatttggtgaaaaccaaggtggtagacgaagtagccaaaag

gggaaacagaggttgctgtgattctgtactaaagacaattgttaagaagaatcaaatgttatgtcatttc

tgtcaacaaccagggcataaaagaaaggattgcctgattctaatggagaagcggtccagagatttgaaac

agtcggaaggccagcatggtatgaaacggagccagttatgtgtgcaaacaactgatgaagaaactctgga

gcaggaatattcgttcacgatgcgtggatttacagccaattcgtggatcgttgattcaggagcaacatcg

catatgtgtatcgatcggtcatgtttcgtggagctggacgagcgttatcagcaggacgtaattttggccg

atggcacaacggcgagggttgaaggaatcggttcgtgtcgaataaccacgctgtctccggagggtaaaac

atcaagagtgactcttaacgatgttttgttcgtcccgaagttagagacaaacttagtgtctgtgaaaaaa

ctgacggcgaaaggtgctgtgattctttttgacatgagcggttgtcgaatcgtaaaggatcagaaggtta

ttgcactagcaacaatttcaaatggattatattcactgaagaccagaatgcagaatgtcttgacccgaaa

gaatgcccgatatcgatcaattgcaaagtgtttattgatgccaatggatcttgaatatcggaatgtacag

cagaaggaggagggactgatgcttaggaaaggcgagcttagaaaacgcaagttacgaaaaggcgagtcat

gttcttttgtacattgcaaagatcggactatgtcatcagggtctcgaatctgggttggatgtcatggaaa

agatatgtttggaaggtgtgagaagtccatacctagaaataatgaatttcagagcgtgtgttgtagatca

tagcaacctcgatagttagaatctgcgcctacaacttggtttagagccagccgagattgaggaggag

>copia3-ltr_ag copia anopheles gambiae str. pest

tgttgtgatcatagcaacctctctagtttaaacgaagtgtagtaggctttattgaaatagagaataaatc

agtctgcattttcctttcgtacgaacaagaagttccaaca

>copia4-i_ag copia anopheles gambiae str. pest

ggttatgggcccagacccaatccaaaattaagtaattaatattctatcgaagataaaagtgccacacttc

acatcacccgaacaaagagtgaaattttgtgtacgagagaaatgagcacggaggaagcgaattccgcagg

accgagcagagagagcagcgcaggagcagcagcactaagcggccacgcggtcgggagcaacaacctcgca

atgaacgtaggtatcgaaaagttgaaaggacgggagaactacgtttcgtgggccttcgcgatgaagatga

tgttgtgtagagaacggtgctgggatattgtaacggctagagacgataaggcggtagacaaagacatgga

catgcgggcattgtccactatcgcgcttagcttagaaaagcacaattacagccttgtgatggacgctaat

acagcaaaggaagcttgggaaaagcttaaagctgcgttcactgatgatggtgtgtttagacgtatctctt

tattgcaagagcttgtttcgttcaaattaaataatttttcttcgaccgaagcatatgttgatgcaataat

gtctacgtgccacaagcttagagaaataggcttcgaagtgagtgatatttgggtttcgtcaatcctgctg

atgggattacctaaatattacgcgcccatggttatgggattggaagcatcgggaatggccatgaaggcag

atgcgataaagttaaaaattttgcaagaagtgaaaaccacgtgccacaaagatgatgaagcgctctttag

tagaggtaatcctaacctgcgtaaagggggaggagcaatgaagagaagcacaaaagaggttacatgctac

aattgccaaaagctagggcattttgcgattaattgtcctgaaaagcagaagcacaagaaaaataataaaa

cacgtgcaatgagttctgtgttggcaatgggtgatgtgagtgaatgtgaatggtactttgattcaggagc

aagttcgcacatggcaaaatcaggtgtagatttctcggaaagacaacacatatgtcacgaggttagtacg

gcaaacaatgctagcatgaaagctattacgaagggcacagtttccgtaaattgccaagaaggtgcggtaa

atttattaaatgtgttagaagtaccagacttagctacaaatttactatcagttagtaaaatatgtaaaaa

tggcttcaaagtggtatttacagaacgtaaatgcgaagtgtttgatgaaaatggagaagtgttcgcatcg

ggtattgctgagaatggattataccgattgaatgaaaatagagtgagaacatttttatcctatgaaatat

ggcacaggcgactaggacatttgaatttccaaagtattcaaagattaaaaggcatggccgatggcattca

aactaaacaaactaacacgtacaattgtgtagcgtgcattgaaggcaaacatgcaagagagtcgtttcct

acaagccaagaaagatgcaaagaaaaattagaactgattcattcagatctatgtggaccatttgaagttg

aatcaattggtgggtcaaaatacttcatgactttcatagatgatgcaacacgtaaagtatttgtgtatat

gctcaagtctaaagatgaagcaaagacagtatacgagaagtttaaatcgatggtacaaaggcaaagtggt

cgacaaataaaattatttagaagtgacaacggtcgtgaatatgtaaatgccagtatgaaagcgagtatgg

aacgtgatggaatatgtcatcaaacgacatgcacgtacactccagaacaaaatggagtagcggagcggat

gaaccgcactattgttgaaaaggttcggagcatgctaaacgatgcgcagttaccaaagcgattttgggcg

gaagcagttaacactgctgtatatttaattaatcggagtcctacgagagcgttaaatgacattactccag

aggaggcatggtcaggcaaaagaccacatttgggacatctcaaaatatttggctctacagttatggtaca

caagcctaagcagaaacgagtaaaactcgatccaaaatccgaacggtgcattttccttggttatgcacac

aacacaaaaggatttagagtttttaacgttgccaccaacgaaataattatcagtcgtgatattattgtcg

ttgatgaaggtcaatgtgaaggttttggcaaggaacaaacaactcctgttgagtttctggaactgctttt

tgctgaaggaaaggatgagtcaaatagtacacgtaataatccgattaatatttcaccaacagaagaagca

tcggatggtcagacagaagaaaccccaacaaggtttgatcagagcaatgaaactccaaggcgcagtcaac

gacaacacaaacttccaagcaagtacaaagattatgtcattaatcgcaaatttgttccttcatcaacatt

agctaacgaagcagaaaacgtatcgagtgactcggactatacgacaccagagagcgagtctgatgaagcg

ttagttgttttctcgcagcgagaagatcccagaaactatgctgaagctatgaagtctgaagatgctaaac

agtggatggatgctatccaagaagagcttcagtctattgaggctaacaatacatggtcactggtagacct

accaccgggacggaaggcgataggcagcaagtgggtcttcaagactaagagagatgtggatggaaatttg

ttgcgttacaaagctcgtgttgttgcgcaagggtttagtcagcagtttggaactgactatgatgaggtat

ttgcaccagttgttaagcagacgactttccgtgtgctaatggggattgcggcaaaaaggggaatggcggt

aaagcagtacgatattaagaccgcgtttctgtacggcgatttagaagaagaaatctttatgaaagttcca

caaggtgtgaaagtggaagataacaaggtttgtagattgaagaaagggctatacggcttaaaacaatcag

caagatcgtggaatcagagacttgatcaagaactaaagcgccagggatatacgaattgcttagcagacag

ttgcttgtacaggaaaagatgcggaaaggaatggtgctacgtcttagtatatgtagacgatttgatagtt

gcgggagataatcttgacatgattgaatcattgcttgctgaattgaaaaagtcgtttgaggttaacattt

tgggcgacataagattctttcttggaatagaagtagagaaaaataagcaaggagattattttgttaatca

gcgcaactacatcaaagatgtaattatctctagtggattaacagatgcaaagccttctagtattcctctt

gatccagggtacataaagatcgaagcagaagaaatcgaactttctgataataaggagtatcagcagttaa

taggcaaattgctttatattgcgattaacacgagaccagatatatcggcagccgtatcaatacttagcca

aaagataagtaaacccacacaacgcgattggtgtgagttaaagagagttgtgagatatttaaagggaacc

attaactatcgtctacgattgagcgaaaaaggatgcgataatggtataattggctattgtgactcagatt

gggccgagaacagaatagatagaaaatccaacagtggatatgtttttaaagtaaacggcggtacggttag

ttggacttgtagaaagcaatcatgcgtaacgttatcaacagccgaagcagaatttgtcgcaatatcagaa

gggatacaggaagcgctgtggttgaaattacttcttgaggaattaaacgatgtacaggaagttattattc

acgaagacaaccagagctgtttgaaaatcttatcaggcgaaaagttgagtaatagaactaagcatattgc

aacgcgctatcattttactaaggatctaattaagaaaggacagatcagctgcgtctattgttcaacagaa

gaaatgatcgcggatctattaactaaaccattagccaggattagaatacagaagttagtaagcttgatag

ggttaagtgtttcactgtgagatatacaacgacagcgtaagggaacttgcgttgaggaggag

>copia4-ltr_ag copia anopheles gambiae str. pest

tgttgttatgaaccaacgcaagatgattctcatgagaactggatttagtaatgaaaacacagaacgaaat

tttatagcaaatagagagaattatattgtcaaaattaggaataaagaaaaccctcttccgttactgcatc

caaccaaacaagacgtgttttctctcagctccgaacataaccctagtagtttacaaca

>copia5-i_ag copia anopheles gambiae str. pest

ggttatgggcccagactcgaaaattgattaaagatccagaatatcgaagacttgaatctagaaagtttag

tgaaaatggcgcttcctagctcaagcaattcctcgtcaagtttcgtcggttcgacaagtattccctccat

tgaacgtctgctcggtcgggaaaattggacatcctggaagtttgctgctaaaacgtttctacaactcgaa

ggactttgggaagtagtaaaacctgtgaaaaaagaggatggaactttcgaaacggtggacgaaaaaaagg

atttgcaagccagattgaagctcatccttttactcgacccaacaatttatgtccatattgaagacgcgga

atcggctcgatctgcttgggacaaattggaaatggcgtttgaggataaagggctttctaggcaaatcggc

ttgcttcacaagctgattaagtctgatttggatacatgtggctcaatgaattcatacgtgaatcaggtaa

tatccacggcaaaccaactaaatgccattggtttcaaattgcccgacttgtgggtaggaatgattctttt

ggctggtttaccggaagagtatcgaccgatgattttggctatggagaattctggtgttgcaatcacaggc

gactatgtgaagacaaaacttcttcaagagaggccgttgctacgtaacgaaaatgttcaagcgttagcca

ctaacaaacgtcgtgaagttttcaagccaaaacagaagccttcttcgtcgaaaggtccgcaatgtaggaa

atgtggccggtatggtcatattgcgaaattctgcaaggatgatcgaaaaggaggaacaacgttgtgtacg

gtgctatcgacatttggaaacagtgaagctaacgagtggattttggattcggcagcgtatgcgcacatga

ctagcaacaaggatttattgagcaacctgcaaaacgcaacaggtaaagtagttgctgctaacggaggaac

tctggacattgtcgctcgtggaactgctataatacaaccgaaatgcatggaagagattgtaacgatcagc

gatgtaaagctgattccaggtttaacatcgaatttactttcggttagcaggatggtagaaaagggatata

ctgttcaattcaacaccaaaggttgcaaagtatataaccctagcggcaagttggtgcttactggtattca

caacaataatcagttcaaggtggaacaggtagcgaacaacatgcaggaggctctgtcgtgtaacacagca

gaaagtttcgaattgtggcataaacggatggggcatctgggtgctgtgaatctcaagaaacttgctggtg

gtttggcaactggcatcacattgaaaaatatggacggtgcggattgcagagtttgcccgttaggcaaaca

ttcgaagttaccgtttccgaagaaaggttctcgagctgaaaatgtcttggatttggttcattcggacatt

aatggaccgatggaaacgcactcgcttggtggacatcgatactacatcacgttcatcgatgacaagacga

ggcgtatattcgtttatttccttaagacgaaatctgaagtagaagttttcgaagccttcaagagattcca

cgcgatggcggagcgtcaaagcggaaggaagcttaaaacactacgtacagataacggtaaggaatatatg

aacaaatccctaacatcgttcttgcagaaagagggcatccgccatgaaacttcgaacggatacacaccac

agcaaaatggactggcggaacgtgccaacagaactattgtggaaatggcgcggtgtttactgttcgaagg

aaacatgaccaaaggtttttgggcggaggcggtttcaacagcagtttatcttatcaatcgttctcctaca

cgtggacacaatttaactcctgaagaagcgtggaatggaagaaaacctgatctttcacatctgcgtgtgt

ttggaacgaaagcgatggtaatgattccgaaggagaagcgacgtaaatgggatccgaaatctcatgagtg

cgttctgactggctttgatgaagaaacgaaaggatatcgtctgtacgaccataaaaagaagcaaacgatc

attagccgcgaagtaatttttttagatgaaggttgttcatcgaacgtgacagttacagcaatgcaagagc

caagaagaacattcgttagactggacatcgaggaaacaacttcaatccaacctgtgcaaatcccggttcc

caattttgcacccgatgctggatcagacagcacgttggaagaaaatgatgcagaaacggatggtgaactc

gatgaaagtacgacagatgacgaaaacaacactagtacagaaacgatggtacaatctgaagacgattcga

gtgattttcttgggttttccggaagcgatgtcgatggctttgtagcagttgcgtctggtatgtacagtgg

aacatatgctgatcctgtttcacaccaagaagcactcgctagggatgatagcaaagaatgggggactgcc

atgcaggaggagtataacgctctgatggagaacaaaacctggacgctaacttcgctcccgaaaggaagac

aagccataaaatgcaaatgggtgtatcggactaaatgcgattcatctggaaatttaactcgatacaaggc

tcgtttggtcgtcaaaggattctcacagcgaaagggggaagattatgatgaaacgtacgctcccgtggta

cggtattgttcgctgcgatacctttttgccctggctatcaagcatgatctgatgatagatcaaatggatg

cagtaacagcatttctgcaaggagatcttgatgaagatatttacatggagcaaccaccttgcttcgttga

tggacagcggaaaacgttggtatgtaaacttaacaaagctatttatggtttgaagcaggcgagccgagtt

tggaacaacaaactggatgcagcattacaacggttcggcctgataccaactcagtacgatccttgcgtgt

atgtaggtagcgaaggaggtaagatcattatcgttgctatatacgttgacgacatgatgatttttagcaa

cgatgtggcatggaaaaagcagctgaagaaacatctttgtagttgtttccgtatgaaggatttaggagca

gcacagcactgcctaggtataaggattcaacggacaaaagaaaccatcaagttggatcaagaaatctaca

tagaatctattttaaagaggttcaatatggataaatgtaaaccagtggcagttccaatgaacaacagtga

gaagctgaccaaggaagaaagtccgaagagtaacaatgaaactgctgcgatgaaagatgtgccgtatcaa

gaagccgttgggtgtttgatgtatttggcacaaagtactcgaccagacattctgtacgcggtgaacatgc

tgagtcgattcaacaaaaatccaggacaaaaacactggaacggagtgaagcatgttatgcgctatcttcg

agggacttctaattttaaattggtttacaaaaaaaatgtagattcgaaaattatcggttactgtgatgct

gattggggatctgatccagatgaacgaaaatccaccactggcaacatctttatggcgcaaggaggagcaa

tttcatggatgtgcaagaagcaaccgacggtagccttatctacgtgtgaggctgaatacatgtctgtatc

ggcggcggtacaggaagcctcatggtggcgcggactttctgcgaaactggcgaatgcggatgaagtgatt

gaaattcgttgtgacaatcaaagctgcattgcgatcgcgaagaatggtgggtatcatccacgaacgaaac

acattgatattcgtcaccatttcatcaaagatgctctcagccgtgggattgtcactctagaatatgttag

cacggaggaccagattgcagatggacttactaaaccattgcaacggactaaattcgagattagtcgcgag

ttaatgggtatctctgaggcttgaggaggag

>copia5-ltr_ag copia anopheles gambiae str. pest

tgttgaagagcaagccttgagtaaaatagtaggctatgattattaatgtaagattctagaaatatagtct

tgttccaaccagcaacctttacacagctactctctaca

>gypsy-18_ag-i gypsy anopheles gambiae

agctgtagacaggataaacgaggcggcgtgattaaagtgcgtgaagcgtaaagcattccaggagtaaagt

attgtactacaagtgttcggtagaatgagtgataacgaaagcgaagtttttgcagcggccacagagggac

cgttcgtgacggagcggaagaagccaggccgcaatgagacagccgagttgagagcaatggtggccgaatt

acaactaaaactcgagcaagcggtgcaacttaacagtggcggaggatggatagaagtgataggccgtgcc

gaggacgaaacgaattgtggggacgagtgccagatatcagggagctgcgcgaggtcattcacccgtttga

ccctagggatacgacctgccctgatgcagcagcatggctgaaaaacttcgacgagacgagtgaagtgtac

gggtggaaagaggtagtgaaactacactgcgcacggctgagccttagcggatgcgctaaactgtggtggg

aagcaaaccagagcaaaatcaaaacgtggtccaatttcgaaggtgcgctactggccggattcccatcatc

taagaacgcagcattttaccacaactggctggtgacaagaaaatggcgcaaggaagagacacccaccgag

tacgtgtacgccatgttagctatgggatgcaaaggcgggtttaacgaggagactacgacgagttacattg

tgaacgggttaggcgagatgtggcggggtgcaagggtagcagcgagtcgcgtaacaacgatcgaaggcct

actcaaggaaatcgcctgggtggaaaacattaccgcagtggctgagcatagcagacacgtcgaccgagta

ggggagaggaggtgctttacctgtggatcggsggagcatgcggcgcgagcgtgcaacaagggtcgagaac

caggcatcggtgaacggagtggcgccgccgggagcagccgcggggcgcctagatcgaattggcgttgttt

cacctgtgacggtatgggacacatctcgacggattgtccccgacgtagaaccgtagcacggggagaaatg

cgacgaacggtggagccggcgagcgggggacgcggaatactaaatgtgaatacgttagatgatgaaggag

agggcagatgtcaagcagaggtaggcatggggaagattaaaatcagggcacttgtagactcgggagctaa

cgtatctaccattcgtaccaaaattgcagggctcgctggaacgccacggtttgtgaaaggaacccttcat

ggatttgggggagcgaaagtggaggtgaacmcgaagagagaaacmcgaatacgactagatgacatccaga

aaatgccttacgcacaagaaaacgtggtccacgaaatcgtatagagctgctagagaacgacataatagaa

ccctcggtatcggcatataacagtccaatcgtgctagttaagaaaaaagatggcaagtggcgtatggcat

tgactatagggcgctaaaccagaagacggtaaaagatcgatacccaccaccggaaatagatagatgctta

aatacgttagaaggcgcaaaagtattcatcagcttagacctgtattcgggttattatcaggttcctgtag

cagaatctagcagggaaaggacggcgttctctacgcctgatggacatttccacttcaaaagaatgccgtt

tggactgtaaatagtggagcagtgttccaacggctatagataagatggtcaagggcttgaagcaaagaat

gtagtagcgtatgtggacgacatattaataggagctaagaccgagcaagaggcaatcaaggtactggaaa

aattattagaggcagttaaagaacatggatttacaattaacctgaaaaagagtaaatttttaatgtcaaa

agtggagtttctaggagtagaggtatcaaaagacggggttaaaccaggagaagctaaatgcctagcggtt

aaggaatttccaacaccatgtgatagcaaggaggtccaacgattcctaggcctggcgggctacttcagga

gatttgtagagaaatttagtatcattgccagaccactacacagtttaacaaaaaaaggtgtggatttcca

atgggggcggcaagaagaggaagcgtttcaagcgttaaaggcgaagcttacagaaagacccttattagcg

ttatatgacaggaacgcagacattgagttacatacagacgcatcgaaggaaggtttagctgggatactgt

tgagcagtagtggggtaggatggcgacccatcagttttttcagtcggaaaacgacagagttagagagcag

ttaccacagctacgatttggaaatactagcagtggtagccagtatagaacgattccgacaatatctgcta

ggccgtccatttgtggtaaagacggattgtagcgcggttaaagatacctacaccaaacgcgagatgaacc

ctaggattgcaagatggttcttaaaaatgcaagaatatgattttgttatcgagcatagaggtggtaacaa

aatggctcacgtagatgcacttagcagaggagcagtagagcaagggagagaagagaccacagtcgcagaa

aaaatattagctatcgacatagataccgaagacttcttagcaaccatgcagcaacaggatgataaactag

cagaaatattgaaaacattacaaaaacctgcgacaaccaatgctgaaaagcagattcacgacaactataa

tatagaagggcatagattaatgcggaaggtagcaggagcgaataagtgggtcgtaccagttagggtgcgg

tggaggatagtgagagcatttcatgacgaaatgggtcattttggaaccgatcgggtgttggatagtttac

gggaaaagttttggtttcctaagatgagaaagtacgtacagggatacatagaagcgtgtccaaattgcgc

atacaataaaaaaaagactggacggccagaaggttacatgcacccgatacctaaggaaccgataccattc

catacggtacacatagaccatttagggccatttgtaaaatcagcaagagggaatcagtacatattagcca

tggtgtgcggattctcaaagtttgcaatactcaaagcagtaagctcaaccaaaacagcaccggtaatacg

attcttagaagaggtgtcagcaatattcggaacaccaagtagaatgattacggaccggggcacagcattt

acatccaaaacgtttgaggagtattgtgaagcaaataatagccaacacattcgggtagcagtaggatccc

cacgagcaaacgggcaggtagaacggtacaacagaactatactgacggctctccgatgcatggtagaaga

agaaaatcgaagatgggatgaaaaattatcgcaaatacaatgggcggtaaataatagcagaagcgccact

acaaaacagagcccaagtagcttggtgctatcgtaccaccccagggatatgcatcgtaatatcatagctt

tagttctccacgacgaggacgataatagaatacaaaacatagaagagagaaaacttaaggccgtggagga

gatagacagagaacaacaaaggcaaaaaataggtttcgacaaacggcgaggggcgcccagcaggtacaat

gtaggtgatatggtgttggtagagagggatatattggccacgggcgagagccgtaaactagagacacgat

acaaaggaccctattgggtagtggcagtgctagctaacgacagatatgttatcgaggacataccaggagc

acaaaggacacaacgaccattgcagaccacgtatgcagtggacagactgaaaaaatggggaagagttcaa

gatttagaattcgagggaagtataggacaggaaaaacggcagaaagaagcatacgcggaagagaaagatg

acgctgaggacagcgatgaatgtgtggtgtccgaac

>gypsy-18_ag-ltr gypsy anopheles gambiae

tgtaaactcgccttaagtgcgaaacgataagcgcaaacgataagatgcaggcagataaggatgagagcga

tcattatcaaaagagagagagataggcattagatgagcgacagtgagtgaggacggacgtgkgcaagcga

ataaagtaccgttaca

>gypsy-30_ag-i gypsy anopheles gambiae

cgtaatattggcgacgaggataaaaaaaggtcacagcagagaatccgcaagtaaaaagatgagtcaaata

ggaaacatagagccctacgtaatgggagaaagtttcaaagagtatattcatcggctggagatgtacttca

ccgtcaatgatattgcggtagaaaaaaaagtaccagtgttgatcacaatggctggtgcgtcgttgtattc

ggtagcaataaagttgtgtgcaccggatgatccacgagataaaacgtatgcagatttagtgaaggtgttg

gagaagcatttcaagcccaacgtgaacgttgtgtccgagcgttatctactcagaaagtgtaagcagacag

cggaacagtcgattgctgattacatcattgagctgaaggcagcggcacagacgtgtgatttcggcagttt

cttatcagacgctctaagagatcaattcgtggctggcgtgtacgatctggagcttcgcaaacggctgctg

aagaaggaagatttaaaattcgtgcgagcatgtgaaatcgctcgatcttgggaagcagcgcaagaacaga

acgaggtaatgaccgacagagagagaaacgtgtcggcggccatgcggaaagcagcaaagcagcaaccggc

gaaccggcagcgagggcaacaggtgatgtgtcggcggtgcggcaaaggtcatgacccgttaaactgtcct

gcgcgaacctggaagtgttatgtgtgtgggaagcaagggcacgtagcgaaagtgtgtcgtggtcgatcgt

atcagagaggcgtacgaaaagtagaaggaactaatacaagtgtaccgcaaatgatcacgttagtgtgcga

tggaaaaaagttagtttttgaaatagattgtggtgcgtgtgcgagtgtgatctcgcaaaagacatatacg

gatgtatttagcgaagtagaggtgaagccgaccaccatgtcttttatatcggcgtcgggtcaatcgttaa

aacccatcggtaaaattgaagtgaagattaggatgccgaaggcggaaaaagaagaaaagttagagttggt

gattatacctacggaaagggaagtaagtcctttattaggacgagatgggttggatttattatttccaaac

tggagaaaagtgtttgagataaaagttatagaaacggatctaattatggaaatagcaaagaaatttccga

aagttataaaatcaggagagaacgaaacaattgaaggtttcaaggcggatttagtattaaaggcaggttg

tggtcctgtatttcataaagcgtacccagtgccatactcaatgcagacgaaagtagaagaagctttagac

aagcttgtagcagaaggtgttttacggccaacacgattttcaaaatgggcaagtccaattgtaatagtga

agaagcaagacggaagtatacgtttgtgtttggacggtaaagtggtattgaataggcaattatcagtgga

acattacccgttaccgaaagtggaagatatttttgctaaaatagcagcatggaaggtattttgcaagata

gatttgtcgggtgcgtacttgcaagtgttattatcggaaacgtctcgtgaaatatgtacggtaaatacgc

ataagggattatttcaatatactcgtatgccttttggcatacattcagcaccggcaatatttcaatcgtt

gatagatcaagtattgcaagatacagaaggaatcgcgtatatcgatgatattttggtaggaggtaccgat

atgcagcaatgtaaagagcgtttatacaaaattttgtggaaattgaatacgcataatgtaaaaatcaatg

tggccaaatcagtcttgttcaaaagcagtattgagtatttaggatatatattatcagacaagggtattca

tcctaacgagtctaaagtgagagcgattgttaacgcacccgttccaaagaatgtaacgcaactgcaagct

tatttagggttggtgaactattatcatcgcttcatacccaatttagcaaatttattacatcccttgtata

accttttgaggaaacaaactaagttcgaatggacggagcagtgtcaaatagcgttcgataagtcgaaaaa

tcagttagtagatcatcaagtgttaataccttatgatgagagcaaaccgttggtattgcaagtggatgcc

agtccttatggtgtaggagcaattttatcccatataatcgatggtgaggaaaaaccaatagggtttgcat

catccacgttaactgcagcgcaaatgaattatgcacaagtgcatagggaggcattagcggtaatattcgg

cataaaaaaatttcacaaatatgtgtatggaagggcatttaaactaattacggataacagtggcgtcaag

gaaatattcaatccgtcgaaaggaacttctgcaattacagcagcaaggttgcaacgttggtcnttatttt

tagcgaattataattattcgattgaacataaggcagggaagttaatgtcaaatgtagatgccatgtccag

attaccgttgcctgaagaaaatgaactggaaaatgtgaatttgggagtaaatgtgctaaaagaaaattca

caaattgaaatcgaggtgataaaaaaacaccaattacaagatggtgaaatcaggaatgtttataatttgg

tgaaaaatggatggaaaaataatttagagccagggttgaaagtttataacaaagtgaaaaatcgtttagg

aatagaaaacggaatattatttttcgatgatagaatagtgattccaaaaangttaagaaacgatatactc

aacaaatttcacgcaaatcacgatggtatcgtaaagatgaaaattagtgcgaggaagtttgtttggtggt

tgggaatggataagcatatttcggattttgtaacagaatgttcgatttgtcaagcttgtcaaccagttag

caaagagatagtagaaacaaaatggccttcatgtggaattccgtttgaanggattcatatagatttattt

tattttgaatctagaacattattaataatagtagatgcgtattcgaagtttatagatgtgcgtttgttaa

atatggccaaagctaacgacatcatagagcagatagaatctttttttggttattttgggattgctcagga

agtggttactgataacggtccgccgttcaattctgaatcatttgtgaattttttgagagagtatggtgta

aaagtaactaaatcaccaccgtatcaccctcaatcaaacgggttagcagaaagaggagtgaggacagtaa

aagaagtattaaaaaagtttttgttagattctaaacataaatttttgcctttgagccgcaaaataagcaa

atttctgttgcactataggaatacgcccactacggttactaaagaaacgccgtcacagagaatttttgct

tacattccagtaactgttttaaataaattaaatgcaaagcaagtagaaaaaaataaggataacgaagata

attgtaaagcggtccgtattcataacataagaaaggaaaataagtatatcgattttaaaagcggagaaaa

ggtagtgtatagaaaccattttaagganttagtgcgatggattccagccactattataaagaaagttagt

tatactacttacactgctaatataaatggaaatatcagattagtacacaaaaaccaattaaggaagctga

aacgcaagttgaattatagttatattactgaaaagaattggtcccaaaatgatacattgttgaatacaga

gaataattataaacgcaaacgtagcgaatcacaatctccacccattaggagatcagatagattgaaaggt

caaccgcgactnaaatattgtaaatagttaatcgtaagtttgcgtttagtggggagac

>gypsy-30_ag-ltr gypsy anopheles gambiae

tgtagtatatatacatatgcgaaattatagcatgtaactaggtgttagtgagatcggtgttagggctcag

gttagtgtaagattatatggtcagttcaataaaagcactcatggttgaacca

>gypsy1-i_ag gypsy anopheles gambiae str. pest

ttggtgaccccgacgtgatctccggattgttattagttctcctaatttgaaaatttgtgaacgaacatgt

ctaccgaaaacgtttctaacgaagaaacttcccctgctactgcagccgtttcggttaaacttccggaatt

ctggaagaacgatccatcgttgtggttttcgcaagctgaaattcagtttttgttggccggcgttcataag

gatgaaacaaaattttatcatattgtcgccaaactcgaacaatccgtgctttgtcatatcgccgattatg

taaaacagcctcctgcgacaggaaaatatgaagccgttaagcagcgcctcatatctcgattcgagctcac

ggaacaagccaaaatggatcagctacttggatcgtacgattttggagaccttcgtcctacgcatctttta

acgaagatgcaggaacttgctgccggattgaatgtgaatgattcgttgttaaaaagactattcctgcaaa

aacttccagctaatatacgtgcaatacttagcatccacgatggaagtctttcgaagctagcggagatggc

agataaaatgatagaaatggctcctcaaacatcagttatccatgcttctgtgcaaaaagaaacgacggaa

aatttagcagaagaagttgctgccatgaaagtagagctacgccaaatgaaagcacggcaacctgagcgcg

gtcgattgcgttccacttctcaaaatcgttccaatgaaaacatctgttggtatcatcggaagtatggaaa

tcgagctacgcgatgtcgaagcccttgccagtatcatcagtcaaaaaactagatttccgcccatccgaaa

tcggcgaggtgggcggattaagaatcagtcgccgtctgcaaatcttcgacaaatcttctggtattcggtt

cttaatcgacacgggatcggatgtatcgataatacctgcatccaagatagagaagactcgagaaccatcg

ccgtttttactccatgcagcaaacggaacgaaaatacgaacgtatgggagcaagtttgtttcagtggatc

tcggactacgccggaagttttcgtggaattttttgcaagccgacgttacttctgcaattattggtgccga

tttcctcgcgcattttggtcttcttgtagatcttggaaacagaaaacttattgatggtggtacgaaatta

cacactgtttgcggattatcgaaatcttcggtttacggtgtaacaactatagcaaaagatcatccttttc

gagacttgctggtcgaatttcgagaaatcaccgccccgccgacaatgcgcactgaggtacgacataatgt

aacccatcatattcaaaccactggacctccagttgcttccaaacctcgtagaatgccgccagacaaactt

caagccgctaaaaaagaatttgagaccatgatggagctcgggatttgtcgcccttccaaaagcagttggg

caagtccacttcattgcgtaccgaaaaagaatggtcaatggcgctttgtcggagattacaggagtttaaa

ccggataactgtgccagatcgttatcctgtgccacatattcacgatttgctaaacaatttcttaggtaag

aattgttttaccactctagatttggtacgagcatatcattttgttccggtcgaggaaagcgacgttccaa

agactgctgtaatcactccgttcgggctctttgaatttaccaagatgcaatttggattatgcaacgcgag

ccagacatttcagcgctttatgcatcatgtcttcggtgatttggattttgtggtggtgtttgtcgatgat

atatgcattgcatcgtctaatgaggaggaacacctatcgcacgtgcgaactgtctttgagcgtctcaaat

caaacggcttggtactgaacttggacaaatgcaagtttgtccaaaaagaagtcaactttttggggtatca

catcaatgcatctggtatcaaacctcaagctaatcgcgttcaagccgttgttgattacagtcgtccgatt

acggtgaaggatcttcgacgatttttggcattgctgaatggttacaaacgcttcatccggaatgctgtct

cattgcaacaaccattgcaagcactcattattgggaatcgaaaaaacgatacaagaaaacttcagtggac

gatcgcagcagacgaagctttcgtgaaatgcaaggaaagtttagcaaacgcagctttattgtcctatcct

gactcgtcaaaacgaatgggactgatgattgatgcttcggatacagcggcaggagctactctacaacaaa

atgtcgctggcgcatggcaaccgctggggttcttctcgcagaaattttccccttcacagaaaaagtattc

tgtcttcggtcgcgagttaacagccatgaaattagcggtgcagtattttcgacatctagtggaagggaga

gaattcactatttatactgatcatcgtccgctcacgtatgctctgaattctaattcaaatcatcttcctc

atgaagaacgatatttgcagtacatttcgagttttacaaaagatattcggcacattagtggcaaagacaa

ttctgctgcagacgcattatccagagtcaacaccatctcagctccttcaacagtggattttgaattatta

tcgaaagcgcagcatgatgatccagaactacagaagctacttgctgatcgaactacatcaatgaacttgc

agttaagatcatctgtttcaactaatcaattgttgtattgtgatgtgtcggataatgtacatgtcagacc

gtatgtaccagagaagttacggttagaagttcttcgcaacatccattgcctttctcatcccggtgttcga

gcaacgagaaaaatggttgcgcgaaggtttgtttggccctcaatgaatcgagacgtcgcccgcttcgtca

gatcttgtattgattgtcaacgatcgaaaatccatcggcatacatctgcggcgctcaacgaattcgagct

tccaaaaagtcgtttccgccatgttcatatcgatttggttggaccacttccgacgtcgaacggaaagcgg

tatttattaacgatgatcgaccggtttagtcgttggccggaagcagttcctttgccagatatactagctg

aaacagtcgctaaagcattttgcgaatgttggatttctcggtttggtgttccagaaacaatcacgaccga

tcaaggacgacaatttgaatccgaattgttcacggaattgacgcggcttcttggggctctccgtattcgt

actacagcgtatcatcccgaagctaacggtcttatcgagcgttttcatcgaacgttaaaaacttcgctca

cttgtgtcgattcgaaacgttggtgcgataaactgccgttggtcctgctcggtttgcgaactgctatcag

ggaagatatcgattgttctgttgccgagatgacatacggacagccactgcgaattcctggcgattttttg

gaaccctcgaagacggaaatatgtcgctcagagtttgccaaactgctttgccggaccatgcaacaaattg

gaccaatcagaaactcgcatcatgacaaacgatcggtatttgttccgaaggacttgcaaagttgcaaaag

cgtttttgttcgaatcgattcagtcaagcggcctcttacacacccatacgagggaccttttcaaataatc

gaaaggcatgaaaagtatatggacttaaatatgaacggtgagaaacgaaggatttcgattgatcgtatta

aaccagcatatatttgtgaaaaggattcgaatgaagataacgaaagaacaaaagttacgccatccggtca

ccgtgttcggttcttggcgtaactgagggggactc

>gypsy1-ltr_ag gypsy anopheles gambiae str. pest

tgtggtggattgcttatagcaatgtcttagttgtaatcaagtagccattggttacagcaaccgtggttac

actgaatatcgaataaatgtagttagtcttagttcactcttagaacggtttacatcgtcttttaatcgct

cccaca

>gypsy10-i_ag gypsy anopheles gambiae str. pest

tggcgaccgtgacagcgtagcaagaacaaagctacaagaacattaaagtggagtaaaagctaaagtgcag

tgctgtgtaaaaagcaagttacgcgcaaagtgcggtgcggaaaaatcgagtgaaaaagttaaagtacaat

actgtaaaaagcaagttatgcgcaaagtgctgtgcaggaaaattgacagtgcccgaaaaaaaaaataaac

cctttaagcatatacattacaagtgaataataagcaagactgaacaagtggataaaaagaactaaaagtg

cagtgagaataagtgtatataaagatcgattaacgatcattaggccatcagaagtcacctgcccaatgta

catccaacactcggacgctgcgataacagtaaagcacccgagcgacggttggatcagcgacgaagacaac

acggattttgacggaccgtcagagccagaggacccagcgatcgtagaggcacggcgtcttacgtggaaag

ccatcgactccgtggagcaaaccacaaacctaaccccagaggaggagttcgaggcgaagcagccctaccg

gccaacacacgaagacatctgcagttgcatccgggacacaaacgcgacgctgaggatcctggtcaaccta

ggcgtggaggtaccgtacgatcttttagaagaacttacagcggcggtcagttgctatattgagggagtag

tcgacacgcaattgggtctagaactcatttcagacgtcgactacgatctcaacctagtagcttgcggtaa

gtcttaccagcacgcctggagaacaacggtgttcgacagactagaagcgcagctaaggcgctaagaagaa

tgtgcttacctcgggaaccgaaacctaaagctgtagtcctcacggaggaggaatgatttcctgtaatcca

agagataaggcaactcaatgatttcctggatcattatgaggattaggaaaataaaataacattatgcatt

gaaaaaaaaataatattcttcttctgaaagcaaatttgaaagaaaatatttttttccactttgaagattt

ttcaccttgaaaaaaaaaaaaaaaaaaaaaaaaatatatatatatatatatatatatatatatatatata

cgtatatatatttaataataatatttttcgtctaaaagtaatttggaaagaaagaaaagatttttacttc

aacggttttttcaccttggaaaaaaaatatatatatatgatagtaatatatatttttttttaactgaata

atatataattcattttacatgactaaactgaagggtattgtaagaagattagaattaatcaacaaaacat

tgcagcaaaatcagggaagagtaaggcagtgtgctctaaccacatacagactacaggtggacgaaatata

ctccgtatttaggaaagaagtagggacaaactacgacaaatacgacgagacagaaattaaattttacaat

aatatcattcaaaatttaatcactaacataatcgaaagaattaacaaagagacaactaacaactcagacg

atttaaacgaaactttgaaaacaagtaagaaactaacattaaagaccacaactcacataatcatatcact

tttagctgcatacaaaagacaaaaacaattcgttcccattgctcacgaagcaataaaaacaaatacaagc

gtagacgcaaataacctacccaacatggatctattaaaaatcatcaatacggcaaccaatgccataccaa

cattcagcggtagagatgacgaagccagggcaatgctagctgcattagaaatgctaaaagaatcagttga

tgaacaacatcatcgcataatagtgcaagctgttgaatcaaaactaaaggggaagggaagaaaaatcata

ggtacaacggttaatagagtcgacgaaataataaacaaaatcaagactcatttgaaaaaaaccgaatcac

cagaagatatagcctcagcaatacatgccacaaagcaaaaaaccacaccgaaggattttggtgaggaaat

tcaagcactgacagaagaactagaacgggcatatctcaatgaaaaaatggatccagaattagcttctgaa

aaaaccaaaaaaatcgctatgacagcttttggaaaaggactaaaaaaggaactacatcaagggctagttt

tgtcaggtacaatccccagcctagaagctgctatcaaagcaattgatatcatggacaaactaagtcaaaa

ctcacaggagagaaagacgaataaatggcagaatggtcaggacaacagatataatagttccaaccaacga

cagcccaataatccaagaaacaatgaacaacgctcaaataacaactggagggcacaacagccacaaaccg

ggagacaagggaatgcccaaaatagacagtctacacagggaacagggtataatactcaaaataggcaatc

cgccagcccaaattttttagggcaacgcgagccacaaagagcagtgcattgcactcaggtggagacccag

aacccacaggtggaccagccctccacaagccaatacgggcaacatacacaataaatgtgcaaaaatcaaa

ttttataaagacaagattagggctagcagattcaatatgcaacctatttgtagattcaggttccgacatt

tctatcatcaaaggcaacaaagtaagacctacacaaacttacaaaccaaaagatatagtggatatcataa

gcgtaggagaaggaacaataaccactcatgggtccacaattacggatgtaatagtggagggaaagaaaat

ccaacaattatttcacatcgtaccagacaacttcaagataccggcagatggtatacttggtagagatttt

tttatggatcaccgatgtataataaattatgatacttggattttctctgtaaaacacgatggagagtttt

tggaaacacccattgaagatactatcaatgacaaaacactcatacctcccagatgtgaagtaattagaaa

actagataagttaaaagaattagatacggatgcggtagtatgcgcagagcaactgcaagaagacgttctt

gtaggaaactgcattgtaaataaaaactacccatttattaaaataatcaatacttctaataaagctaaat

tagtaaacattagccatatcaaaacaatacctttaaatgaatttgaaatcgtaaaaactaataatcataa

gaatgaaaataggttagcaatcataaaggaattaatccgaaaggaaaacatttccgaagatacagataca

tcttttgaacaattactgttaagctacaatgatatttttcacctacctaatgatcatttaactacaaata

atttttataaacaagatataaaattagaagataaaagacccgtgtacataccgaattataaacaaaatca

ttcccaaggaccagaaatcaaaaagcaaattgaaaaaatgcttcaagatgatgtaatagaacactcggtg

tcacattacaattcacccatcttactagtaccgaaaaagtcctcagatgagaaaaaatggagattagtag

tcgattttagacagcttaacaaaaagctgctccctgataaatttccactacctagaatagactccatatt

agatcagctagggcgagcaaaattttttagcacattagatctcatgtcaggatttcatcaaataccactg

gaagaatcatctaaaaagtatacagctttttcaagcacggatggtcactatcaatttaaacgattacctt

ttggattgaacatttctccaaatagttttcaacgaatgatgaccatagccatgacaggcctcacgccaga

atgcgctttcgtatacgttgatgatattgtagttgtaggagcttcggaaaatcaccatctaaaaaattta

gaaaaagttttcgacagactaagacactacaacctcaaactaaacccagagaaaagttgctttttcaaaa

aggaagttacttatcttggacataagataaccgacaagggtattcttccagatgactcgaaatacgatag

cataaagaattacccgataccacaaaacgcagacgacgtgagaagatacgtagcattctgcaattattac

agaaagttcatcccaaattttgctttgaaagcaaaaccgctaaacagtcttttaaagaaaaatacaaaat

ttgaatggacacaggagtgtcaagaagcattcgaatatttaaaaaacacactgattagtccacaggtttt

acaatatcctgatttcagtaaaccatttatactaaccacagatgcttcaactatggcatgtggagctgtt

ttagcacaggaacacggcggcaaagatatgccaatatgcttcgcgagtagaacctttacgaaaggggaag

cgaataaagcaataatcgaaaaagaactagccgcaatacattgggctataatgcatttcaagcattacct

atacggtaaaaagtttaccgtcaaaacggaccatagaccaatagtctatctgttcggtatgaaaaatccg

tcatcaaagctgacgagaatgagattagatttggaagagttcgattttacagtcgaatttgtaaaaggga

aacagaacgttgtagcagacgctctatcgcgaattaaaatcacctcagatgaaataaaatctatcaatgt

gattacgaaaagcatgagcaagcctgttacttccgataatgttttaggaaacacgtcagagtctgatcaa

ctcaaaatgttccatgccttagcatacgacgaagtaaaagacttaccaaaactagaatcatcagtaaaaa

agaatgaagacactatcgagttgataggaaaaatcctaaacaaaagaaagtccaaggagctcttatcagt

aagagacatccatctgaaaacagatataggactgcaggagcctttattagtaaaggatttccaacgaaga

aaggaaaaatctgccatagtgcaatttatcaaaaacatagaaaagaagctcgtaatgaaaagcattaccc

agctagcaatctctgaaacagacgagatattcaaagaggtgcacccaaacgaattaaagcaaatcgctaa

caatcatttgaaaaatattcagatactaatatatactaaaccacaaaagattactaacgaaaagacgata

aacgacatactcgacaaagtgcacaacacgccgacaggaggacacattggacaatataaaatgtacaaga

agattagaagagaatattcatggaacaaaatgaaaaagacaatcaaagaatttttagacaaatgcttaac

atgtaagcttaacaaacatcaaacaaaaactgcagaaccttttgtcaaaacagacacacctaacactccg

tttgaagcagtatcaattgatacagtaggcccatttcaaaaaacaaacaacaataaccgatatgcagtaa

caattcaatgtaatttaaccaaacatgtaacagtcatagcaattcccaacaaagaagcgaatacggtagc

tagagcggtaatagaaaaaattatgttaatatacggcacaaatataaagaaattcagaaccgatatgggt

acagaatacaagaatgaaatatttaaaaacatatcagaaatcctcaaaatagaacacaaattttcaactc

catatcacccacaaactataggagcattagaacgaaatcatagatgcctcaacgaataccttagaatatt

tacaaacgaacacaaggacgattgggatgattggatcaattactattcattcgcatataacacaacacca

aatttagaccatggttatacaccatttgagttagttttcggaagaaatgaaagaataacaccgaatgtga

aagatacatattcacctttatataattatgatgattattctaaagaattcaaatacagattaaaaatagc

tcataataggactagaaaacatatagaacaagtaaaattcaaactattaaaagaacaacaaaacattaat

caagttaatttcgaaataggagatcaaatagccctgacaaatgaaaatagaaccaagttagatccggtat

acaaaggaccatataaagtaaaagagataaatggacctaacatgataattgaaaacacggaaggtgtagt

acagaaaatacataaaaatagagcgattaaattatgacagaataacttcacttcattacgttattcttcc

gaagggtggagg

>gypsy10-ltr_ag gypsy anopheles gambiae str. pest

tgtagcagatttactgctaacagttcaaccaacctatagagccacctaagcagttatacaatcgttacta

accaaaaaccggatgacataacattaagagttgaaccaactcaaaaagccacctcaacataatcgttgct

aaccaaaaccatccaactaaaaacacattcaaattaaccagcaatcaaatcaggtgagacagaaaaccta

ccatgcaataaatgcttagcacacacaaaccgtaggacaggaagcgcgaaccgacaacttataaaaacat

aacgcgacggtaacatgaaacgcgcctcgcaactcagaacggctgcaaaacgattgcgcaacaataacac

caagtgacaaacaaacaaccggacattgactttatattttgatgaacaaacctcaaaacaggaaggttag

caaccacataatgaaagtaggtcaatattagtcgtaaacataaaataagtgtaaaaagacattgtgtcat

cactgtttaagaatgcgttataaataaagcttattcgaaacagtgagacagttcactagtttcagacacg

tagatcactcgtgtttagtgtttctccccattagcagaagccgccgaaggctctgcccaattggtaacca

cgtttggttatcagctgttcagtcggttgaccgatcagcaataccccttctacggaagaagtgcaagttt

gcaccttaatcggttaatactcccgcagcgttttaacgccgcctggaatccgatccatcgcgcaagtaaa

gttccgtagttccacccgtccgttgcataagtgtaacttgaccatctcttcctaacgaagtgaacgaatg

atacaattcattcgcaattaaaccaactacggtacgttctcgcctcgataccccgcctcgttctgcactc

ttcctccacattccgcgcatcggcttaccgcacacggttctggtaagcaaagtgcgcgccgaacctacca

ca

>gypsy11-i_ag gypsy anopheles gambiae str. pest

tggcgaccgtgacttttaaactgtaatcttcggatgtgcaaaaaaaaagtgacgaatgaaacctcaaaca

cggacaaaaagtgcaaagtggaaacgttttataaaaatcgcaagtgcttctgaaatgacttggaaagtga

ttaattaccaacaatagtgaactacgagtgaaaaccaatcattttcaaaatgggaggcaaggctgcaaaa

cctgaaacaaatataaaaggagaccatgatctcacaatagttcaaactcagaatattcatacagaatatc

atctgactcaggatttaaaactaaacattattttagggctgctaatcaccctgtgcattgttaaaatagc

gaaaacttgttacaaacaccttcgtaaccaagcgcaaaaacacgctttaaaagtgcttacgctaccaaag

tagcaacgtaaacattgaatcgagaacagtgaatgaatgatatacgaaaaaggtgatatgctatttgacc

cacaaaaataggtaaaggctgtgggaaagtaccgtaagttcaccaatgcagctatggaccgcgattatga

aaaacaattatgcgcgtatgagaacctacctcaacgtgtaaaaacactgtgaccagcggtatggtttgga

acagccgttcccaacgcggaagacgagaagaataaggcaagaatggacggttgctcactgtcggacagac

aggtgaaagaagagatccctgggccaagggacagcagttcaccgagcaccgggaacaacgtagcaccgtg

agcagcaacaacaacaaatgtatttaaaaaggtaccgtacacatgcaatttttacggacgaagctgcata

aatatgcaaaagctgttagaaaaaatagaaatactagatagaacgtatgatcaggttagacagctaaaca

aatgctataggctctgcgcgttaaccacactaagaaataacactaaggaattatatgacgaaatacaaga

gcttctacgaaagcacgaatcatccattaaagacgaaatattaacaaccttagttaaaaaaagtagacac

ctatattacgaaataaataagtgcattaaaatacatttcgaaagacatccagattcgttaaatacaacat

tatcagaaaaccagttcgacataacgatagaaactaaatctgacaaaatggctgacattatagaactaat

taaaatcaccacttctctcatatcaaagtatgatggtaatgagaaagatttaaaaggtgtggtgtcaaat

ttaaatgtattaaagaaaatagtgaagccggagaatagggaaacaataatagagctagtattaggacgtc

tgacaggtaaagcgcgaattgttgtaggagaagccccaaaatcaatagaagatatagttaacaaactaca

agacagatgcagcataaaggtaacaccagaaatagtagtatccaaaatggataatacgaaacagacagga

acaatagaagatttcggaagcattatagaaaaactaacgcagcaacttgaagaagcatacatagcggaag

aaataacaccagaagtagccaggaaaaaagcaactaagtctggaattagcgcattgagttatggacttaa

ggatggcgaaaccaaaataataatgagatcaagcaaatttgaaaccctgcatgaagcaatagaacaagca

gtaaaattggaactagaagacagaacgaaaaagggaaagaatgaacagacaaaaattttatattcaaacg

ctactaggaacaatagagggtatggtaacaactaccagggaaggaacaattacaatagattcacaaataa

taataataataggtatcagacacaaaacccacccaggttcccacccgcaagatatggacataacaataac

cgaaacaataataactacagaaataacaactttaacaacactagaaatcagcacgcaaatcgacaaaata

attccaacagaaatcagtacgtacaatccaatcagagaaataatagcaatttgcaaaataatcgagcgcc

tattcataacacagtaacagccgaagaacagaataattttttagggcaacctcaagcatcggaaaatacc

caatactaaccataaatcctgatgcagataattttgttaaagtaaaaatagaaattacaaaggaaatcta

tagcacactcatcatagataccggagcaaccgtatccgtacttaaagctagtaaattaaaaccaggttgt

aaaatcaatacatcaaaaaaattaaccttgataagctctagtgatcatgaatcagagactttaggaactg

ctatgacaacaattcactttggcgattattccattatgcacgaatttcatataatagaagatgtagaatc

cattttttccgacggactattaggaaaagactttataaagcacagatgtattgttgattatgttaattgg

atgatatacttctcatctgataacggattgatttcacacccaatagaagacaatgtaaacggaaattata

ttttaccaaaacgaagtgaagtagtacgaaaaataagtataccaaacttgacagaagattcaatcatctt

atcacaagaaatccaaccaggggtattttgcggaaacacaatagtctcaaaacgtaatcagtatatcaaa

ttcattaataccacagataaagatgtttcttttaacataaaatcttatacaccagaagttgaaccattaa

gagagtatgagcaattacagaagaaacttgacacatctaaggaacgaattcagaaaattcataacaaaat

ccatatagaaaatattccacaaatagcaagagaagagttagaaaatcttatcacaaaattctcggatata

ttttgtttagaagatgaaccggtctctactaacaatttttatacccaggaaatttcattaaaagataata

ttccttcttatataccaaattataaacaaatacattcacaaacagaggaaatgcaatcacaggtagaaaa

gatgttgaaaaataacattatagaacattctgtttcatcatataattcaccgatactattagtaccgaag

aagtcaggtgaaggaaaaaagaaatggcgtttagtagtggattttcggcaattgaacaagaaaattttac

cagacaaattccctttacctcgcatagacacgatactagatcagctaggaagagccaaatatttcagcac

attggatttgatgtcagggtttcatcaaatcaaacttgataaaaattcgagaaaatacacagctttttcg

acacctacaggccactatcagtttacaagaatgccatttggactcaacattagcccaaacagctttcaaa

gaatgatggctatcgctatggctggtttaacaccagagctagcatttgtatatatagatgatattatagt

tactggatgcagtgcacggcatcatatcagtaatttaggtaaagtttttgataggctaagaaaatataac

cttaaactaaatgcagaaaaatgttgtttcttcaaaacagaagtaacgtacttaggtcataaaataacag

ataaaggaatttatccggacgacgcgaagtttgacacaattaaaaacttcccgattcctactaatgctga

tgaagcaagacgttttgtcgcattttgtaattattaccgtaaatttgtacagaattttgctaagatagct

aaacctattaataatttgattaagaaagacgttaagtttgcatggacttcagaatgtcaagcagcttttg

atacattgaaacaaagcttactctcacccacaattttacaatatccagattttaaaaaacaattcattat

tacgacagatgcatcggatatggcatgtggtgcagtgttatcacaaataacagatggaaacgatttgcca

gtcgcatttgcgagtaaaagttttacaccaggggaaaagaataagccaattatcgagaaagagcttacag

ctatacattgggcaattaattattttaaaccttatgtatatggacaaaaatttatagttagaacagatca

tagaccattagcatacttatttggtatgaaaaatcctacttctaagcttactagaatgagactagattta

gaagaatttgactttgaaatagaatatttagcaggtaaagctaatgttgcggcagacgcactatcaagaa

taatccttaactcagatgacctaaaagcatcaataccaaaatccaaaacgattttaatggttaatacaag

agccatggttaagaaaaataacgagaaaactgatataaacaaagacgaaccaatcgcaacaacagggact

gatcaccccgcgatgtggaaaacagatagacctttagaagtgagaaaggtactaaaaataggtacgcaga

gaattaagaacaacgttgaattcataatatacaaccattcatacagtaaagcactaggaaaatttctttt

gagaaatgatgtaaatggaagtcaagcattagagtttgctcttctagaaatgtgcaaaatcgcgaaacaa

tatggaagaaataagctagcatggtcagaagaagatcatttattcgaagaatattcccaacaaactatta

aggaaatagccaacagagccattaccaagtttgaaataatcctgtttactccaactagatggataacaac

agaaaaagataggctgagaataatttcagattatcatatgaccccttcgggaggacatataggccagtac

agactgtaccagaaaataagggaaaaatataaatggaaaaatatgaaagatgatatcaagaaatacgtac

gaaattgtaaagcatgcatagttaataagacgactagacatactaaagaaaaaacagttgtaactacaac

accgacaaaaccatttaacataatttcaatcgacacagtaggacctctaacaaaaactaacaaaaacaac

aggtatgcaataaccatacaatgtgacttaacgaaatatatcgtagtaatacctatccataacaaagaag

caaacactatagccaaggcattggtagaaaacttcatccttacattcggaacatttatagaattaaaatc

agatcaaggactagaatataacaatgaaatattacacaaaatctcagaaattttaaaaatcaaacaaact

tttagcacagcttaccacccacagacaataggatcattagagagaaatcatagatgtctaaacgaatacc

taagaagctatacaaacgaacatcatgatgactgggacgattggacaaaattttacgaatttgtttacaa

tacaacagaacactcagacacaaactacacaccatacgaactagtatttggtagaaaagcgaatttacca

caagatatatttaaaacaaaaattgaaccagtttataatattgaccaatattatttcgaaatgaaatata

aactccaaaaatcaaacgaaattgccagagaaaatttgataaaagcaaaaattaaaagacagcaaacctt

aaataaagatacagtaccacttattataaacttaggagatcaggtatatttggaaaatgaaaataggaaa

aaattagatccagtctacattggacctttcacagtagtaagtgaccaagggcctaattgcgtaatacaaa

ataatacaacaaagaaaatctctacagtacacaaaaatagattaattaagtacacaggagaataacttca

atcattgtaattcattacgttattctattaaaggggggagg

>gypsy11-ltr_ag gypsy anopheles gambiae str. pest

tgtagcatgcacatgcacatgctatactgttttcaatcagttcacacacatttcttattacacttaataa

acacaaaattgtcaacacaacacaacacacaaaaagaccaaattccaaccactcaagataccaacacaac

ccaaaccacataaataaccttacgtcaggaattgtaagtcagcagaaaaacccttatccaaaaacactta

aaacacacaagcgacacacaaccccggaggtgcgaaaaaatcttacccagcacaaaacgcttcaagtggg

taaagcgtaaaaacacagcattgcataatagcaaccaacagttaataaataatgaaataggagacaccgt

acctcgtgtgtacaaacagaaccgtaagcgtaggaaacaaaacaccgcgtaagcgtcagaaccaagaact

gaccttacataataagcacaaccatatgtaaactcaaaacttgacattcagcataattgacacaaaatag

aaagtaaagtaaaaccaaatctaactaaaaggaaatgaatcgtataaataaagatgtaagatgagaccga

gtgctgctcagttacacacagtacgcacagtgacacggttcagtggagccgttcaagtcttatcgtaatt

cgcacaaacaacacaaatatgttaaattaattcattgtgtccactcgttacctggagccctgatggctcc

cagtgatcatccgaaactaatgctttgtttttgacgaattgacctcgcgagtgatgtttaatccttctgc

accgctgctctaacaattgcaacctgatacagaatattaca

>gypsy12-i_ag gypsy anopheles gambiae str. pest

tggcgaccgtgaattttaagctgcaatcttcggatgtgcaaaaaaaaaagtaatgaatgaaaccccaaac

acggacaaaaagtgcaaagtggaaacgttttataaaaatcgcaagtgcttctgaaatgacttggaaagtg

attaattaccaacaatagtaaactgcgagtgaaaaccaatcattttcaaaatgggaggcaaggctgcaaa

acctgaaacaaatatcaaaggagaccatgatcttacaatagtccaaactcagaatattcatacagaatat

catctgactcaggatttaaaactaaacattattttagggctgctaatcaccctgtgcattgttaaaatag

cgaaaacttgttacaaacaccttcgtaaccaagcgcaaaaacacgctttaaaagtgcttacgctaccaaa

gtagcaacgtaaacattgaatcgagaacagtgaatgaatgatatacgaaaaaggtgatatgctatttgac

ccacaaaaataggtaaaggctgtggaaaagtaccgtaagttcaccaatgcagctattgaccgcgattatg

aaaaaaaaacaattatgcgcgtatgagaacctaccgcaacgtgtaaaaacactgtgaccagcggtatggt

ttggaacagccgtgcccaacgcggaagacgagaagaataaggcaagaatggacataccccgtcccaaccc

ggacgagacatagagactgatgatgagatcgagacatcgggcatgactccagctacgagaactccaaggt

actgcataacccttaatttataaattaatatgatgaagaatgaaacaaaaaatagaactactagtagaaa

gattaaaacaactgcatgataatctaaaacacatagacagatgctacagacagtgtgcaataagcacata

tgaggaaagcgctaaggaaacttttaaaaagctacaagaaaagttagttaaatatgagaatgaaataagt

gaagaagaactaacgtacacatctaaaattgctagaacactatatagcgacataactaaattcatagcaa

ttcacaaacaaaagtttacagtttcaattaataacgttagtttaggaacccaaacaatggcgacgtttaa

cctaaaagaagcttcggctgtagtaccgacatataatggatccgcagaccaattacaatccttccttagg

gcaatacattttgcaaagagaatgttcccggatgatcaagagggactattggtagacttcatttacacta

gattatctggtaaggcagaaaccggaataaaacccaaccttaccacagtacagcaagtagcagaggatat

aaaatcacgctgtgaagaaaaggtagaaccaagcaaagtaatagctaacatgaaatcattaaaaactaag

gatacaaagacactttgcaaagaaatagaagagcttacagaaaaattgaaggttctctaccttcaaaaac

aaattccaaccgaagtagcaaatgaattcgcaattcgggaaggaataaattcattaatagataaagtgtc

aaatcgagaagctaaaacagctctcatcattggaaaattcacaactataaacgaagctactaaagtagta

gatgaatgcgaatctcgacaagacaaagcacaagtacttgcttaccagggttcatacaacaatagtagat

tcaataattatagacaacagaacaggaattatcagtataataaccgtcctaactcaaaccaatcatacag

acgcaatgacaattacagaaacaacaacaatttcaataaccgcaattataacagtgatggctacaacagc

aatcaaagaaataataaccgcagtggcaataatagagacaaccaaaatcaaagatacaataataatggct

atcaaaacaggccaaatagaagtcctagggctataacgaatcgagaaacagggcaacaatccagaaacgt

atatcatactacagctcaggtacaggaggatgagaataattttttagaccaaaaggaatcaactcagact

ctagaccaatatactcgctagatttgaatggagttgattacataaaaataaaattgagttttgctaatac

cgaaacatctatattattagtcgacacaggagcatcagtatcattattcaaatcaagcaaattaaagaaa

aatcacagtccaataagatcaaattcgatttcattgacaggcatttctaacacaccaatatattctaagg

gtattacaacttgtactattttttttaacaatttagaattggaacatgactttgtattagttccagatga

atttaacataggagcagacggtatattaggtagagatttttacaaactttacagatgttctattaactat

gaattactattgcttacattcacatgccaaggagaggaaattcaacataatattgaggaagacgacggaa

aaggatttattttacctatcagaagtgaagtagtacgaagaatataccttccaaatataactgaggacac

catagtattcgctcaagaaatccaaccaggagtattttgtggtagtacaatcatttctaaagataatcaa

gtagttaaattcatcaacacaaagcaacgaaatatctacataactcatgcagaattcaaacctattacag

aaccattatcgaattatgaagctaaacaagtaaataacaaagcaggagaagtaaacaatgatagactgca

aaaacttttacaaaaaattaaaatagataaaattcccacctcggaaatttacaatctaagaaaaattgtt

acagaatacaacgatattttttgcgtagaagatgatccaattactacaaacaatttttaccctcagaaaa

tcgaattaaaagacaatatcccaacttacataccaaactataaacaaatttattcacaaacagacgaaat

acaaaatcaagtagacaaaatgcttaagaatgatataattgaacattcagtttcaccatataattcacca

atcttacttgttccaaagaaatcaacagatggtaataaaaaatggagacttgttgtagatttcagacagt

taaacaaaaaggttataccagataaatttccattgccaagaattgactcaatactagatcaactcggcag

ggcaaaatattttagcacccttgacctcatgtcaggatttcaccaaatacctctagaaaatgattcaaga

aaatttacagctttttcaaccggatcagggcattaccaatttaaacgtatgccgttcggtttaaacatta

gccccaacagttttcaacgtatgatggctatcgctatggcaggattaactcctgagctagcatttgtata

tatagacgatataatcgttactggctgcagtgcacggcagcacatcagtaacatagttaaggtttttgat

agattaagacattacaatttaaaattaaatccagaaaaatgttcattttttaaaacagaagttacctatt

taggtcataaaataacagataagggaatatatccagacgattctaagttcgaaacaattagaaattttcc

aatacctaaaaatgcagacgaagtacgaagatttgtcgcattttgtaattattatcgtaagtttgtacat

aatttcgctaatactgctaaacctttaaataatctaattaagaaaaaagtaaaatttatttggacagacg

aatgtcaaaacgcatttaatagcttaaaacaaagtcttttatcacctacagttttaaaatatccagattt

taagaaagagtttatattaacaacagacgcttctgatgtagcatgtggagcagttctttcacaaataaca

gatggagaagaccacccaatagcatatgcaagcaaaagcttcacaccaggagaaaaaaataagcctatta

ttgaaaaagaattgacagcaattcattgggcaattaattatttcaaaccatatctatacggtagaaaatt

tactgtaaaaacagatcatagaccattagtatatttgtttggtatgaagaatccaacatcaaaactaact

agaatgagattagatttagaagagtttgattttaaaatagaatttttagcaggaaaaactaatgtagtag

cagatgcgctatccagaatcgtaacggattctgacgaacttaaagcatctatccctaaaaataagacgga

tttagctaatcctattttattagtgaatactagagcaatgacaaggaaaaatataactgcagataaaaaa

gaaaaagagaaagacaaggaagaaacgaaagtgaaatatgatcaaactaatatgtatgaaacagataggc

catctgaaacaactaaaatgttgaaaatgaaatcaaatattattgaaaatgaagaatttattgagctcgt

gatatacaatcacaattattacaaagcgctgggaaaatttaaaatacccataacttctgcgaagcaaagt

caaacactagagtttgtactgcatgaatcatgcctaatcgctagaaagtatcacaagaatttagcaattg

catcgaatgacaagttattcgaattttattcaatgtcgaccataaaagaaattattaacaaaatgataac

agacgttcatgtcatcgtgtatacacaacctaaatggatagaagagaaagaagaacaatttcaaataatg

tccaatttccacacaacaccagtaggaggtcatctaggacaattcaaactatatagtaaaataaaagata

aatacaaatggaaaaatatgaaagcggatatcatcaagtatgtcaaaagttgtaaagcatgcgcaactaa

caagatcctaaaacatactaaagaggaaactgttgtgacgacaacaccctctaaaccttttaacatcata

acaattgataccgtaggaccgctaccaaaaacagcaaacaataatcgatacgcagttaccatacaatgcg

aattatcgaaatatatcgtaataatcccgattcaaaacaaagaagcaaacactatagcaaaagctttagt

agaaaatttcattcttacatttggaaactttttagaaatgagatcagatcaaggacttgaatacaataat

gaaattttaacccaaatatcaaaaatattagaaatcaaacaaacattcgcagcagcatatcatccccaaa

caataggagctttagaacgaaaccacagaagcttaaacgaatacttacgaagttataccaatgaacacca

tgacgattgggatcaatggactaaattctatgaatttgtttacaacacttcagtacacagcataactaac

ctcacacccttcgaattagtatttggaagacaagcaaatttaccacaagaactatacaaaacaaaagtag

acccagtgtacaatatagaacaatactacaatgaaatgaaatttaaattacaaaaagcacatgcaatagc

caaaaacaaactaatcgcatcaaaaatacaacgtcaagccaaacttaatgaaaatctaaacaaattaaac

atacgcataggagattacgtttacctaacaaacgaaaatagaaaaaaattagatccagtctacataggac

catttacaatcgtagaaatcacggatacaaattgcgttataaaacataatcagacaggaaaaatcacaac

ggtacataaaaaccgattaaaacagttttagtgaataatgcactcattcgtaaaaaactcaatcgtacat

tattcaaaaagggtggagg

>gypsy12-ltr_ag gypsy anopheles gambiae str. pest

tgtagcatgcacatgcacatgctatactgtctataatcaattcacacgcaattctcatcacacttaataa

acacaaattgtcaacaccacacaacacacaaaaaaggataaaataaccactcaagaaattaacacaaccc

aaaccacataaataaccttacgccaggaattgtaagtcagcagaaaaacccttgtccaaaaacacttaaa

aacacacaacccgcacacagccccgtaggtgcgaaaacagcagaaaaacattacccagcacaaaaacgtt

tcaagtgcgtaaagtgtaaaaaacacagcattgcataatagcaaccaacagttaataaataatgaaatag

gagacaccgtacctcgtgtgtacaaacagaaccgttaatcgtaagcgtaggaaacaaaacaccacgtaag

cgccagaacccagaactgaccttacagaataattgcaaccatatgttaaccgaaaaacctaaccctcagc

ataaccgacacaaagcgaaagtaaacgcaaaatcgaattgaagaaaggtaccgtataaataaagatgcaa

gatgagaccaggacacacaggcagagacagttagaatcacagtcacgcacagtacgtacagttgtaaagt

ggtgctgctaagtcttggtcggtcaagtctcgatttgcaaacaaagtgaaatgtgtatttaattcagttg

tgtttgtccgaaacctccaccatcgtgcttacaaatatacctgaagagtgatgtctatagtatggcatcg

ttgaactatccgtcgcgtccgaaccaacttattaca

>gypsy13-i_ag gypsy anopheles gambiae str. pest

tggcgaccgtgacaagtgtgaaactgtgatgcaaaaaaaaaaaaaaaaaaaaactcgcgtaaatacgaag

ttaattcgcgagtgcgtaatattccttccggtcagaagaatattctagtatctgcgttgatgtttagcgt

gaaagagtgagagagtgaagacgagagtgcagcgcaacgctacaattgtacgataactcgaactatcgtt

attcaaacgaggtgcagtgataaaatatacgggtttcctgaaaaatcacgaaaattgcacaataactcct

gtcgtgtatcgtcaacgaaccaaggtgcagtgaaagagagagtgtatcctggttataaaaaaaaaaaagt

ggccaggaaaagttatatgaatgctagcaaacattcggtgataaaaaacaaaaaaaaaaagcagcgatag

ttaacggtaccgtgcgtaaagtgatctaagtgcagtgacctcgatcggacaagattatcgcacgtacgag

taagccacatttcgtaccatcggcgataagcatcacagtttgaaccggcatgaccttttgcgacaagtga

ccaagtgtcgtttgcacgagagagacataacgttgttgcgagaagaaggaagaacggagcagccgatatc

aaacggcaatacagcgtgcggtgaaggagcgacggcgaagcagtgagctgtgagagagcgagcagcgatt

tgcagtggataggagcgacggtgcagcagtgtgctgtgagagaacgagcagcgatttgcagtggataaag

cgagataacgctatacttatcgatcgggcagcggtacacggagtaaggatcaagcgaaaaaaagggagaa

agtaaggtaggctacacactcaatatagttgcaatttactcatttaaatattttttgtaattgctcaatt

aaattcttttttatgaaacgtacaatattaccattttccattacaatatgcacatactattggggtgtta

gtctaaaaaaaagaaaagaaaaaaaaatattgcaattatctgggtgtcaaaaaaaataataataataaaa

aaaatataaaataaaaaataaataaaacaaaaaaaatataacaataacatctcgatcacatctcattcaa

tgctcttaaaaataaatcaaaatattataaggcttaaaaatattgaaaaaaaattatcgtgtgatagaaa

gtttcgcagatgtttgcttgatcattacagtggttccgctaaattttgttttgataagatcaagtattac

ctggattcaattcaggaaactgaacccgtttcagagctgaaacgaattttgaaggaagcgagtgacatat

acgccaatatccaatcgcgcatacaatttcatcaaacaaacaaaattcaagttaagtttaaaactttagc

aaaactagctatcgtgttcaaccgttggaatcaaaacaaaatggaaactttcgatatcaaaacagccacc

gctttggtgcaaatatacgatggcaatacagatggtttggaaaattttgtggactcttcaaatttactaa

aggaattatgtccaaaaaatccacaaatgttggtaaaatttttaaaaacaagattaataggaaaggcgcg

tttaggactgccgcctaatattgacgattttgactcgttaataaatgatatacggtccagatgtcaagaa

aaacttaatccagataaaataataagtcaattgaaatctattcgccagacggatactaaagctctttgtg

atgaggttgaattactcagcacaaaattaaaaaacgtttatttgcagctgcacattccggaaaaagttgc

aaatgacatgtcattaaagcatgggattaatgctctgattgaaaaagttcataatcaggaaacaaaaatt

gttctaaaagcaggtcaattcgcatctgtttcagatgcatcggagaaagtattagaaaacgaaaggaata

gcaacggttcacaaatattagccttcaacagaaactactatgacaatagaaaattcgttcgtaataaata

cccgccgaacacatacaatagattccagccaaacacaaataatagatggttttccaatagatctcccctt

ggtaacaatcaggcaactagcaacagataccaaaaccaaagatatcccaataactctttcagaaaacaat

ttccaaatacaaatactcgtagagtctacaatacacaagctgcagaagaggaacattttttaggggtacc

acaggcgttagaagaaatcattcaacctacaaacgatggtcaacattagcgataaacttaaatgctaata

actttatcaaaattaaagtagaaatggcaatgggtgaaataagcatattaattatcgacacgggagcaga

cgtgtcattgttcaaagttgataaaataaaaccaacacagcaagtctacgcgcaaaataggataaactta

acgggcataacaaccgaatcggtatcaacattagcaactactagcactaatataacattcggaaatgctt

ctattaaccacacttttcatatcattcctgcagaaatagatatcaaagcggatggtattttgggaagaga

ttttttcactaaacatagatgtgttatagattatgagcactggcttttaaatttcaattgtaacggtgtt

acaatcagccaccctattgaagatagtattaatgatggtttcatattgcctctacgcagtgaggttattc

gaaagataaatttgcccgacctcgttgaagactctattgtccttgcgagcgaaatattaccaggagtatt

ttgtggcaactctgtggtatcaaaacaaaaccaatatgtaaaatttgtaaacaccactgaaagtaatgtg

tatattgaaaaggattcatttaaaccacagattgaccctctcaaaaattatattcagaaaaatttaaaat

ttaaaagctctaaaacaaatgaatctaaaataaaaacattattgagcaaaatcgatagtcagaatgtccc

caaatatataatccctcatttagaaaaaatattatccgagtaccatgatattttctgcaccgataatgat

caaatcccaactaataatttttacgagcaatcgatacaattgaaagataacgtaccatgttacattccaa

attacaagcaaatatattctcaaagtgacgagataaaaaatcaggttgataaaatgcttaaaaatgacat

aaaagagcattcggtttctccatataattcaccaattttattggtaccaaaaaaatctggagataacgaa

aaaaaatggagattggtagtagactacaggcagttgaacaaaaaaataatgcctgataagttcccgctac

ctagaatcgatgatatattagaccaattaggaagagcaaaatattttactaccttggaccttatgtccgg

gtttcaccagatacccttacacaagaactctagaaaatatactgctttctcaacttcttcaggccattat

caatttacaagactcccttttgggctgaatataagccctaatagctttcaacggatgataacgatagcaa

tggcgggacttacttcggagagtgcttttgtttacatagatgatattattataacgggttgcacaatgca

acatcatctagaaaacttaataaaagtgtttaacaggctaagaaattacaacttgaagctaaatccagag

aaatgtatttttttccaaaacggtgtgacctatttaggtcacaacataacatataaaggcatatatcccg

atgagtccaagttcgaaacgataaaaaaatacccggtaccaataaatgtggatgaagttagaaggtttgt

agcgttctgcaattactatcgcaagttcgttaaaaattttgctgaattggtaagaccgctaaataattta

ctaaagaaaggagctacattcacgtggtctaatgaatgtcagcatgctttcgacactcttagaagaagcc

ttgtatcacctactatattacagtatccaaactttgagaaagagtttatcattactactgatgcgtccga

tgtagcatgtggcgctatcttatcgcagataacagatggtaatgacctacctattgcatacgcgagtaaa

agttttactaaaggagagaaatctaaaccgactatagaaaaagagctaacagcaatacattgggcagtaa

attatttcaaaccatatatttttgggaaaaagtttaatgtacgaaccgaccacagaccattggtatatct

attcaacatgaaaaatcctacttcaaaactaacacgcatgaggttagacctggaggaatttgattttgaa

gtggaatttgtgcaaggaaaaacgaacacaggagctgacgcactatcgagaattgtcattgattcagaca

aattgaagcatatgcaaaagaataatgcaaacgaaacaaatgataaatcactactagcagtaaaaacaag

agcaatgacaagactgaacaatttcaagcttcctccagaattaaacgaaggtgaaaaagattccataact

aaacctacactttggcaaacagaaaacccaacagaagtaaacaaacttttgaaaatcctatcaaaaacag

aatatggcaaaatcaaaataacagttttcaataataattacaacaaggagttaggaaatacaacaattca

ttgtaatgaagatggaagtcaggcgctagagtctgctcttctaaaagtacaacaaatagcaaaagaatac

aatcgtgacaaaattacaatttcattagaagattccctattcaggcaatactcttttcacaccataaagg

aaatcgcagataatgccatttccggtttagagatcattgcttttattccaccaaaatggatttcaaaaaa

ggaggaaatagaaaatattttagaaaactatcacatgtcaccttcgggaggacatgtcggacagtatcga

ttatacctcaaaattagagaaaaatataaatggaagaatatgaaggaagaaattaaaaattacgttagag

gctgcaaagtatgcaaagtaaacaaaatcgtaagacatacaaaagaaaaaagtgctgttacgactacacc

tttaaagcctttcgaagtagtctgtatagacacagtgggtcccttgccgaaaaccaataaaaatcataga

tacatcgttacaatgcaatgtgaactctcgaaatacattgttcttattccaacagaaaacaaagaagcga

acacgatagctagggccatggttgagaactgcatattaaaatacgggaggtttcttgagatgaaatcgga

tcaaggccttgaatacaataacgaggtattaaaaaaggtagcggagttgctggggatcaagcagactttt

gccactgcctatcacccagaaaccataggttccttggaaagaaaccacagaacactcaatgaatacttaa

gagcatttacaaatgagcatggtgacaattgggatgattggctaaaattttacgaatttaattataacac

aacacctcatacagatacgaactatagcccatacgaattaatatttggaaaaaaagcaattcttccccaa

gatttatgccatggtaatattgaaccagtatacaattatgatgcatattttaatgaaattaaattcaaat

tgcaaaaatcaaacgaaatagcgcgtaaaaatttaattgagcaaaaagaaagaagacagattagagcaaa

tagtaatgtaaacccccttatcataaaaataggagatgcggtttatctaaaaaacgaaaatagaaaaaaa

ttagatccgatatacttaggaccgtacatagtagtaaagttagataatgttaactgtacaataaagaata

caaatacaaacaaactaagtacagtgcataaaaatagattaattaagggatgaaaagcatacgcttttca

tttctaaaggggggagg

>gypsy13-ltr_ag gypsy anopheles gambiae str. pest

tgtagcgcctgcaagataggcaaaacgcgcacagaataattatagctagctaggtttaaattatacacat

atacacttacacacatacacatagttagattttaattatgcatatatatatttacacacatacatctaca

attaaggttacacgtaggatagaatagaatagcataagataggaaataggaaatagtaaaataggcgata

attaggattagccaatgaggaataggattaaggaattttggcgcgaacgagtgagtataaattaaggata

agttgaggtttaggatcattaacaaaagtccgatctaagaggaaacgctgcccgagttaacatagaagaa

ataaactccaagtaactacacaaattcaagccacattattcttttgggtagttagaagatttcatatcac

taca

>gypsy14-i_ag gypsy anopheles gambiae str. pest

tggcgaccgtgatcgtgaaatccaacgacgacacgaatctgtgataaatccacgcaataccaaatcggat

gtgcacagaataccaacaacaaaacatccatcggtatacaaaaaaaaataagtctaacggtaacgtgtgt

aattcctcgactacgcaaacccgaagctagcaagtgtgcttgagagtacattgaccgacgtttggagcaa

cgaccgaggatcacgtcgaatcaggactagattcccaagcggtcaggtgaaatcgaatgcgtgtcaagag

cgagagaaaaaaaaaataagaaccgtacgggaatagcacaccacggcaaatagtgatcggtaatagtcgg

agagaacagaggtgtgagaaaattaatagcgtaaaagcgggcgtaatgagagtggtggattgctatcatc

atcagcagtcgaaggcatgagaagagtgcgacgaaagtagtgatctgtacggcgagtgcagtgtacagtg

taccacgaggccagtgaacagtacgagaagtgtgcgacaaaagtagtgatctgtacggcgagtgcagtgt

acaatatgagaagtgtaccaccaggccagtgaacagtacgagaagtgtgcgacgacggcggtagacggca

ggagtatactgtgtgacgacagcagtgaacagtacgagaagtgtgcgacgacggcggttgaaggcaggag

tatactgtgtgacgacagcagtgaacggaaagagaagtgtacgacgagagaaacgagcagtaagagaagt

gcatcacattatcagcgagcggtacgagaagtgtgttgtgacgacggcagcggaaggtgagaattaacga

gagcagcggacggcgtttggcgagagcagttgacggcgtatgaagtgttcgagaatatcggacggcaaat

gcagcagcgaaatacgataatgacccctcccccccgtaaggtaggatatgcgtagtgaaatagaattggg

ttacaaagaatatttttgggtttaattatactaaggagctaaggattaagggttaaattataattgtaat

tgctcaattaattttttttctcgcgcaaaaaaaaaaaaaaaaaaaaaaaatatatataaaaaattaaaat

atatatgcatacatatatagaaaaaagtcactattcaatataccccttcacgattatttaccttcgttgc

ctcaaaatagtcgacatgctcgtttaaatgttacctaagatcaaacaatatttaaaaagattacaagata

ttgaagaaaaattaaagccgggtgataaaaagtttcgcagatgtttgattgaacactatggtgtcactgc

aaacaacacgttcgaaaaaataaaaaatttagtatccaaattaaagatagcagaggcgccagtagaatta

gaaacaattactaaatttgcaaatatcagctacaacaacattaattcgtacataaagtttcaattacaaa

ataaagtccaatttaaatttaaaacacttgttaagattagcattgcattacgccgttggaaaaggaaaac

catggaggatttcgatataaaaaccgcagcaacattagtacaattatatgacggaaatgcggacagctta

gaaaattttctgggttcagcaaatttattaaggaagctttttccgaaagacacgaagcagatactagtag

agttcctcaaaacacgattaacaggtaaagctcaactagggttagcaccaaacataactgattttgaaac

ccttatccaggatgtaaaatcacgatgccaagaaaaaataaaccccgacaaggttatagcagaattaaaa

tcgatttgccatacagacacgaagaccctatgtgacgaagtggagatactatgtacaaaattaaaagtga

tgtatctcaaacaaaacataccggagcaagtggctaacgacatggcagtcaagcgaggaatagaaacact

cattgacaaggtcaaactcacggaaacaaggattatattacaagcagggcagtacacctctatatcagat

gcaacagaaaaggcactagaaagcgaaagaatgcacaattcagctcaacaagtgttcgcatttagaaaaa

actttagtgctcccaatcgctcctttgacaatagatattcaaatcagtacactcgtaatacgtacaacag

gtcacaaaatagaggaaattttacttcaaattggcaaacaaataatcggcatcaacataataattttcac

aacaccaataaccgacgatttttcagaaataccaacagaaacaataacaggatttacactacacaagcca

cagaacaagaacattttttagaagaaaacccgcagcataccgattaccaatcttagccataaatctaaat

gccaataaccttatcaaagtaagagtagaaatgtcaacagaagaatacagtcacctaatcatagacaccg

gagcagatgtttctttgtttaaaataaataaaatcaagtccaaccaacatatatctgcgcaaaacaaaat

taatctgacaggtataactactcatacaatagatactttagccactacttttacaacaatacatttcgga

gagacgtctgtcaaacaccaattccacctgataccaaaagaattagatattggggcagatggcattttgg

gcagagatttttttgcaaactatagatgtgtaattgattatgagcattggctactcaattttactcacaa

tggcatggccgtctgccaacccatagaagataaatccaatgatggtttcatattacccacgcgaagtgaa

gtgatacgaaaagtaaaactgccaaacatcgacgaagattctattgtgtttgcagaagaaataaggccag

gattattttgtggcaattcaatcatatcgaaggaaaaccaatacgtaaaatttataaacacaactgagaa

aaacattttcatcaaccataagtccttcagaccccaagtcgatcaagtgaaaaattataatgttacaaaa

ccctccggtaacaaggaaataaccagatttgatagtataataaatagcataaatatgaaaaatgtcccac

aatacatagaaagtgacttaaagaacctcctagcaaaatatacagacctgttctgcgtaaatgaagataa

aatttccattaataatttttacaaacaatcgatacagctaaaagacaacataccgtgtttcgttccgaat

tataaacaaattcattcccaatcagatgaaataaaacaacaagtcgataaaatgttaaaaaatgatatta

tcgagcattcagtatcatcctataattccccaatattgttagttccgaaaaaatcagagacagagaaaaa

atggagacttgtcgtagattaccggcaattaaataaaaaaataatgccagataaatttccattgcccagg

atcgatgaaatattagatcaactcggtagggcaaaatatttcacaacactagatctcatgtcaggtttcc

accaaatacctttggataaagaatcgcggaagtatactgcgttttccaccgctacaggccactatcaatt

caaaaggttaccctttggactaaacgtgagcccaaatagttttcagcgcatgattacaatagcaatggct

gggttaacccctgagagcgcgttcgtttacatcgatgatataatcataacaggttgcactactaagcacc

accttgataatttaaagaaagtttttgatagactacggcattataatcttaaattaaatcctcaaaaatg

taaattcttccaaaccgaagttacttaccttgggcacaaaattacagacaagggaatataccctgacgaa

tccaaatttgaagcaattaaaaattaccccaaaccagttaactcagacgaagttagaagatttgttgctt

tttgcaattactaccgtaaattcattcgtaactttgcggaaatcgcgaaacctttaaacgaattgttaaa

aaagggaaaaatattcaattggtcgtccgaatgtcaacaagcttttgatacccttcgcaacaaccttcta

tcacctatggtcctcaaatatcctgattttacaaaagaattcattatcactactgatgcgtcggacacgg

catgtggggccatactatcgcaaatatgcgaaggaaatgaccatcctgtggcatttgcttctaaaagctt

taccaagggtgagaaaaacaagccaaccatagaaaaggaattgacagctatacattgggctatcaattat

ttcaaaccttatgtctatggtcgaaaatttaaaattcgtaccgaccatagacccctagtttttctgttca

acatgaaaaatccgacctccaaactaacacgaatgagattggaccttgaggagtttgaatttgaagtaga

attcgtagccggtaaaactaacgttggggctgacgcactctcaagaatagtaactacctctgatcgtctg

aaatcattgcaagaatgcactaatgaggccaacaacaatcaagctttagtagtaaagactagggcaatga

ctagagctaatagagaggaaacaaatcctgtagcacaaaacgacacaagcgagaagataggcaaaccagc

attttggaacaccgagaacccatcagaggttaataaattattaaaaattcagtcaaaaataaataaagat

tcaattaaaataacactttttaacaacaactacaacaaggaactagaaagtacaataatcagattaaacc

agaatggaagtcaggcattcgaggctgctcttctaagattatgtacttatgcaaaaaagcttaatagaga

caaattagctatctccatggaagacgaaatttttactcaatactcgcctcaaaccattaaggaaatcgta

aatagaaccattttcggttgcgagattattggatttgtaccacctaagtggataacagcaagaaaagaaa

tagaagaaattctacacaattatcacatgctaccttcgggaggacatgttgggcaatatcgcctctatct

aaaaataagagacaaatacaaatggaaaaacatgaaagaagatataaaaggcttcgttaaaaactgcgaa

atgtgtaagataaacaaaataacacgacacacaatagagaaaccagtagtaacaacaacacccttaaggc

cgttcgaaataatttcaatagatacagtgggccctcttccaaaaacagccaagcacaacaggtacgctgt

tactgtacaatgtgacctcacaaaatatgtaatcttgataccaacaggaaataaagaggccaacacaata

gcaagagctctcatagagaatgtcattttaacatacggtaaatttgcagaaatgcggtcggatcaaggtc

tagaatacaacaacgaagtgttgaaaaaagtagcagaagcactggggatcaaacagacgtttgcgactgc

ttatcacccacaaacaataggcgctttagaacggaaccatagaactttaaacgaatatttgagagcattt

accaacgagcatggcgatgattgggataactgggtcaaattctatgagtttacttacaacaccacagcac

atacagatactggctatacaccatttgaactattatttgggagaaaagcaaatctcccacaagacatagt

gacaggcaaaatagaacctgtgtataatcacgaactttattacaatgaattaaagtacaaactacagaaa

tcaaacgaagtagcgcgaaagaaattaattgagcaaaaagaaaaaagacacatcgctccaaactgcagca

tcaacccgctaataattgaaccaggagataaggtgtacttaaagaacgaaaacaggagaaaactagatcc

tttatatttaggtccatatattgtttctaaaatacaaaacccaaattgtacaatcgtaagcaaatacagc

aaaaaggaaagtactgtacataaaaataggttagtcaaacgatgaaaatctatgctatacgattttcatt

tctaaaggggggagg

>gypsy14-ltr_ag gypsy anopheles gambiae

tgtggcgcatacacataggcgtaatgaatataattatagtaagctatatagcagaaattatagtaagcta

tctttataggaatagttgtgattggtagaagcgtgttagtataggttagaataggatagctaatacgaaa

agataaggatagaataggtagcataggaaattaggcataagacggaattaaggatacataggaagtaaat

gaattaagctaggattagatagggaaattttagtataaatagcgggataggatttaggatagtcagataa

aagttagacttcataaggggaaactctgtccaattatagagaaaattatagagagtataaataaaagtta

cagttccaacaaaacacccctttttcaattgtacactaaaagtgatataccaca

>gypsy15-i_ag gypsy anopheles gambiae str. pest

tggcgaccgtgaccttaatctgcaatcgacgggcagaagtataaacaaattatttgtgatacgtacaacg

gtaacgtacagcagtgtcgtgctaacgcatcaaatagtgtaaaaaaaaagtgcgtttttgaccataaccg

gtatacgacggaaaaaagtgtcaaatgtgcaaatttgtattgaacaacgcagctggcacaagtgacccat

atataaaaaaaaaaacaatcgctatgaatgagcgaccaaaagcaaatacagtgtggtgtacaaaatgaca

actcatcgaaaatataagtgatgaaccgtttgaagggcgaaaaaaaagtgatgtacagcaatacaaaaaa

ataaagtgttgtgacaaccttcctcaacgtgcaaaaatgcaacaacattataaataaacccttaagtgat

cagtttgcaaactagaacaatcttgaagtgattaataacgcaacgaacccaaaccaacgtaagcggaaat

aacgatgtcacagtgctgtgaaaccataagcagaagtgatgttcatctgaaaggtgatagaatgaatggc

gagactacggtatacgagatgtgactagtgtgattgaatggaacaaccgttcccaacgtggaagacgaaa

agatactgcgagctcactgcccaatctggatcggcagacgagaatggacaagtatcccaaccccgacaag

acatcgagcgacaactgatgacaccgtagaacttcacatcaagcaccaattaaacaccgagcaccggtaa

cgagcgttacaccatgagcagcaacaacaacaaatacatgctatgtatttaaacaaggtaccgtaaatat

gcaattttattgatgaggctgcataaatatgcaaaaactattcgagaaaatagaaatattagatagaatt

tatgatcaggtgaaacagctgaacagatgttataggctttgcgcgttaacaacattaagggataatacta

aggaaatatacgacgaaatacaagaactcctacgaaagcacgaatcgtctattaaagatgaaatattaac

aaagctagttaaaaagagtagatacgtatactacgaaataaataagtgcataaaaatacacttcgagaga

catccagattcgttaaatacgacattatcagagaaccaatttgacataacaatagaaacgaaacctgaca

aaatggctgacattatggaattgattaaaatcaccacttctctcatatcaaagtatgatggtaatgagaa

ggatttgaaaggtgtggtgtcaaacttaaatgtattaaagaaaatagtaaagccagaaaataaagaaaca

gtaatagaactggtactaggacgtctaacaggaaaagcacgaattgtagtaggggaaaccccaacttcaa

ttgaagatatagttagcaaattacaagacaggtgcagcataaaggtaacaccagagatcgtagtatcaaa

aatggacaatactaaacagactggaacgatagaagattttggaagcgttattgaaaaattaacgcaacaa

ctagaagaggcatacattgcagaagagatagcgccagaagtagctagaaaaaaggcaactaaatcaggaa

tcagtgcattgagttatggacttaaagatggcgagaccaaaattataatgagatcgagtaaattcgaaac

cttgcatgaagcaatagagcaagcagtaaagttggagctagaagatagaacgaaaaagggaaagaatgat

cagacaaagatcctatattcaaacgctaccaggaacaatagagggtatggaaacaactaccagggaagga

acaatttcaacaaattctcaaataataatagatatcagacacaaatcccacccaggttcccacccgcaag

atatggacagaacaacaacagaaataacaataacttcagatataacaacaataacactaacaacaacaga

aatcagcatgcaaatcgacaaaattattccaacagaaatcagtacgtacaatcaaatagaaataatagca

attggcaaaataatcgagcgcctattcataacacagtaacagccgaagaacagaataattttttaggcca

ctctcaagtatcagaaaataccctatactaaccataaacactgatgcagataattttgttaaagttaaaa

tagaaattgcaaaggaaatctatagcacactcatcatagatacaggagcaaccgtatccgtacttaaagc

tagtaaattaaaaccaggttgcaagatcaatacatctaaaaaattaacattgataagctctagtgaccat

gaatcagagactttaggtactgctatgacaacaattcactttggtgattattccattatacacgaatttc

atataatagaagacgtagaatccattttttctgacggactgttaggaaaagactttataaagcacagatg

tattgttgattatgttaattggatgatatacttctcatctgataacggattgatttcacatccaatagaa

gacaatgtaaatggaaattatattttaccaaaacgaagtgaagtagtacgaaaaataaccataccaaact

tgacagaggattcaatcatcttatcacaggaaatccaaccaggagtattttgcggaaacacaatagtttc

aaaacgtaatcagtatatcaaattcattaataccacagataaagatgtttcttttgaaataaaatcctat

acaccagaaatcgaacctttaagagattatgagcagctacagagaaaaccaaacacagctagggaacgaa

ttgagaaaattcataacaaaattcgcatagaaaatattccacaaatagcaagagaagaattagaaaattt

gatcacaaaattctcggatatattttgtttagaagatgaaccggtctctactaacaatttttatacccag

gaaatttcattgaaagataacattcctttttatataccaaattataaacaaatacattcacaaagtgagg

aaatgcaatcgcaggtagaaaagatgttaaaaagtaacattattgaacattctgtttcgtcatataattc

accgatactactattagtaccaaagaaatcagttgaaggcaagaagaaatggcgtttagttgtggatttt

cggcagttaaacaagaaaattttaccagacaaattccctttaccccgcatagacacgatactagatcagt

taggaagagccaaatatttcagcacattggatttgatgtcagggtttcatcaaatcaagcttgataaaaa

ttccagaaaatatacagctttttccacgcctacaggccactatcagtttacaagaatgccatttggactc

aacattagcccaaacagttttcaaagaatgatggctatcgctatggctggtttaacaccagagctagcat

ttgtatatatagatgatattatagttactggatgcagtgcacggcatcatatcagtaatttaggtgaagt

ttttgataggctaagaaagtataaccttaaactaaatgcagagaaatgttgtttctttacaacagaagta

acgtatttaggtcataaaataacagataaaggaatctatccggacgacgcgaagtttgatacgattaaaa

acttcccgattcctactaatgctgatgaagcaagacgttttgtcgcattttgtaattattatcgtaaatt

tgtacagaattttgataagatagctaaaccaattaatcatttgattaagaaagacgttaagtttgcatgg

acttcagaatgtcaagcagctttcgatacattgaaacaaagcttactctcacccacaattttacaatatc

cagattttaaaaagcaattcataattacgacagacgcatcggatatggcatgtggtgcagtgttatcaca

aataacagatggaaacgatttaccagtcgcgtttgcgagtaaaagttttacaccaggagagaagaataag

ccaataatcgagaaagagcttacagctatacattgggcaattaattattttaaaccttatgtatatggtc

caaaatttatagttagaacagatcatagaccattagcatacttatttggtatgaaaaatcctacttctaa

acttactagaatgagactagatttagaagaatttgactttgaaatagaatatttagcaggtaaagctaat

gttgcggcagacgcactatcaagaataatccttaactcggatgacctaaaggcatcaataccaaaatcca

aaacgattttaatggttaatacgagagccatggttaagaaaaataacgtgaaaactgatacaaacaaaga

taaaccaatcgcaacaacagggactgatcaccccgcgatgtggaaaacagatagaccttcagaagtgaga

aaggtattgaaaatatgtacgcagagaaataagaacaacgttgaattcataatatacaaccattcatatg

gtaaagcactaggaaaatttcttttgagaaaagatgtaaatggaagtcaagcattagagtttgctcttct

agaaatgtgcaaaatcgcgaaacaatatggaagaaacaagctagcatggtcagaagaagaccacttattc

aaagaatattcccaacaaactattaaggaaatcgccaacagagctattaccaagtttgaaataatcctgt

ttactccaactagatggataacaacagagaaagataggctgagaataatttcagattatcatatgacccc

ttcgggaggacatataggccagtacagactgtaccagaaaataagggaaaaatacaaatggaaaaatatg

aaatttgatatcaagaaatacgtacgaaattgtaaggcatgcatagttaataagacgactagacatacta

aagaagacacagttgtaactacaacaccgacaaaaccttttaatataatttcaatcgacacagtaggacc

tctaacaaaaactaacaaaaacaacaggtatgcaataaccatacaatgtgacttaacgaaatacatcgta

gtaatacctatccataacaaagaagcaaatactatagcaagagcattggtagaaaactttattcttacat

ttggaacatttatagaattaaaatcagatcaagggctagaatataacaatgaaatattacacaaaatctc

agaaatcttaaaaattaaacagacttttagcacagcttaccacccacagacaataggatcattagaaaga

aatcatagatgtctaaacgaatacctaagaagttatacaaacgaacatcatgatgactgggatgattgga

caaaattttacgaatttgtttacaatacaacagaacacactgacacaaactacacaccatacgaactggt

attcggaagaaaagcgaatttaccacaagatatattcaaaacaaaaatagaaccagtttataatattgaa

caatattatttcgaaatgaaatataaactccaaaaatcaaacgaaatagctagagaaaatttgataaaag

caaagaatagaagacagcaaatcttaaataaagatacagtaccactcattataaaaataggagatcaggt

atatttggaaaatgaaaacagaaaaaaattagatccagtctacattggacctttcacagtagtaagggac

caagggcctaattgcgtaatacaaaacaatacaacaaagaaaacctctacagtacacaaaaacagactaa

ttaagtacacaggagaataacttcaatcattgtaattcattacgttattctattaaaggggggagg

>gypsy15-ltr_ag gypsy anopheles gambiae str. pest

tgtagcatatctgctatacagaacatattgtaatacacactatcagccatagacacatagtaacacactt

tggaaagccaaatcacaccattgtaacacattcattataacacactcagtaaatcagatcataccataca

cacccggaagagctcaggccacaccgttcatataaaaacaattgtgacacacttaacagaaccaaccgaa

acgtacaacataagcaggaacagtttatgctttataacccaaagcaggaacggtatatgcattaaaccca

aaacaggaacttagtgacaagaaccatcgttcttgtcaacaaaagacaaacgtaactagcttagtgaaaa

caataacttttcaacatacaacccggaaccaaatgtgagaaacccttaacttcttttgagtataaataaa

accaactccgatcatggcaggtcagattcgttcggactgtcaggataggactatgcccatcctacatcta

tcgagttctcatttaaagagtttagatatgtccccctgcccaaggtaaggcctctaaactccaacgtttc

cagtaagggaaacctcctaaaggtttctatgatcgcctggacgatcaagtgtcgaaagtccgcttcgaaa

cagtatccacctcagttctacttaattcggcaaacgatgacgaaggccaccctgaccaacgcgagttcaa

agttactacatggcaaccgtcccaaaatcgaacatactgtgactatctcaaaacataccacatggcgacc

gtaacagtcatcaaacatattaca

>gypsy16-i_ag gypsy anopheles gambiae str. pest

tggcgaccgtgacacagagccaaagcaaaggaatacgaacaaaacactgaagtaagcataacgaaaggct

gcaggttataaaaatcccgttggacacggtatgtaaacactatggtaaaatttatattttgaaaacaaaa

ataccatagcgcgaaaagcggacgaaggtatgtgtaaaaagtatcgcaacaatcgcgagtgcaaatccgt

cgtaaacactattatgcacaatacatatacactaagctcacaaacactaaagtattcggcatagatgtac

gtttagatcacattacacttataaacacaaaagccgatacgcgtcgtaaagtgagatgcaataatgtgca

gtgacaccaatacaaattagttccgtgatacaacaactgtgaaaaaactcacccgaaactgcatgaatat

cccgaaacatcaccaatacagtgctaaaaatgaaatcaaatgtgaattaaaagcaacaacaattacacat

gcagtgacacataaataccgaaccgaaatactaatcagaatagtgcacaagaaagtgtgaaactaagtgc

gctaagtgaaaccgaactgaacaatcccataatcgcagcagcagcagcacttccacaacgaacaacacga

ggaaagtgttaagaacgatagaacgcgagcaacaatacgcgaggaagcagtcaacgacgccatcgcagta

tacacatcgagcagctgttaaactacacatcgtgcatgccgtaggacacgcaaacgataagtttcgttaa

gtcaggtattgtaaaattaacactatgggatatatatgaatattcagtaaaaaaaatatatatatataaa

aaaaaattaataaaaaaaaagtaaataaaaaaaaataataataataaaaaaaataggaacaattatagtt

tttgcgaaataattttattggacttaaacgttaattaattatgttatttattatgattgtcgttattatt

taatggatcgaatttaatgagtaagttaaaggataaattattccagttaaggttaatacaacataaatta

gagtatttaccaaagaattttagagtttgtactgtaagtaaatataaaattttggctagagaactattaa

catccatacaatcggatttgatagagctcggagaagccataagtgaagagtatttaaacaaaattaacag

tgaaaccaacgaatgcttttcagcgattgagtttcgcataaacaattaccaaatgacaaacgattcccaa

acagaaaaaatggcgcatttcgatattaaaattgcaacatctcttgttcaaacatatgatggatcaaaag

agaatcttgacattttcatagattctgtaaacttacttaaagagattactccaaatgatcaaaaggtaat

gctggtaaaatttatcaaaacacgaataacagggaaagccagaataggattaccacgagatctggacaac

atcgatgaaattctagaaaatataaaagtaagatgtgaagaaaaaacaaatcccgaaatagtcatcaacc

aattaaaaactatccaggcaagacaaaccactaaaatttgtgaagaggtcgaaatcttaactagcaaatt

aaaaggactttatctagaacaaaaaattcccgaagaagtagcaaacaaaatggctgttaaagttggagtt

gacacacttaaagaaaaaatttccaactctgaaacaaaaattctacttaaagtcggacattacccaacaa

ttacagcagctacacaaaaggtattagaaaatgaaatcgaaaacaataattcaaatcaagttttagcctt

taatagaaaccaaaatttttccacaaataatttcaaaagatttcctaacagcaaaacaaataattataat

agatataactcgaacccaaacttccaaaacagaactaagaggaggtattatgaaagtccaaattcaaata

gaacgaacccaaatacatataccccaaatagaaacagagatagacatttctataggaaccctagacaata

taatcaatttggaagacaaaatacacagcccaataacttctcaaatcaacaagctcgtagaatatttact

actgaagctgaagaagaagaggaacagaataattttttaggccaagcccggcaggaatagaacagctgcc

ggtaatgacattgaatttatctgccaataattcaactacaattaaagtagaaaaagctgcaaataagtat

agttcattatttatagacacaggagcagcagtttcattatttaaaataggaaaaattagatctcagcatg

agatagatagcaataacaaagttagacttacaggaattactaataactctttaattacctatggtacaat

tagttcaaatttaatatttaacaacgcaacagttagtcataaatttcacttgattccagatattatagaa

atagaagccgatggaattttaggaagagatttctttacaaaatataaatgtaagatagattatgaatatt

atttgttaaattttgatatcaatggaatctcaatatctcatcctatagaagacaattcactcttaattcc

agcaagaagtgaaattattcataaaatagatttaaagaatttaaaaaaagattcagtagttcatgcacag

gaaataacaacaggaatattttgcgctaatacaatagtttccaagaataatccatgtataaaatttatta

atactactgaaaaagatactattattaacacgaaatttttcaaacctacaatagagccaattaataactt

tgaaatttataaagtagcagaaactaaaacaaatatgcatagagttaaagaaatattaagcaaaatcaat

ttaaaaaattatccagatattattagtcaagaaataagtaaactagtaaataaattttctgatttatttt

gtttaaatgaagacccaattacaactaataatttttataagcaaacaattactcctaaagacaatattcc

tacatatatacccaactataaacaaattcattcgcaaagcaaagaaatagatcagcaagtaaataagatg

ttagaaaataatataatagaaaactccgtttccccttacaattcaccaattctgcttgtccccaagaaaa

caggaaacgataagaaatggagattagtagtcgattttaggcaattgaacaaaaagatattaccagataa

gtttccacttccgagaatagacacaatattagaccaattaggaaatgcaaaatatttcagcactctagat

ctgatgtcaggttttcatcagatccctttggaaaaagaatcgcgaaaatatacagcattttctacaccaa

aaggacattatcaattcacaagactgccttttggactaaacataagtcctaacagttttcagcgtatgat

ggcaatagcaatggctggcctcacacctgaaatagcattcatttatatcgatgatattatagtaatagga

agctcaatgaaaaatcatatagaaaatcttacacaagtatttaatagattacgatactacaatcttaaat

tgaacccagagaagtgcaaattctttaaatcagatgttacatatcttggacacaaaataacggaccaggg

aatataccctgatgaaaccaaattcgaaacaataagaaagtttcctataccaactaacgcagacgaagtt

agaagattcgtagctttctgtaattactacagaaaatttatacaaaactttgcaaaaattgcaaagccac

taaatgatttattaaagaaaggcacactttttgaatggaaaaaggaacaacaaacagcatttgattcatt

aaaggagcatctgctatctccaaccattcttaaatacccagactttagcaaagattttattataactaca

gacgcatcaaactttgcgtgcggagcagttctctcccaaatcacaaatgggcaagattttcctattgcat

tcgcaagtaaaacttttactaaaggcgaaaagaataagagtataatagaaaaagaattagtagctataca

ctgggcagtaaaacattttaaaccatatgtttatggacggaaatttacaatcagaacagaccatagacca

ctggtttatctctttggtatgaaaaatccaacgtcaaaattaaccagaattagactagatctggaagaat

ttgatttcacaatacaattcgtagctggaaaaagtaatgttgcagcagacgcattatcacgaatcgtgta

caattcagatgagttgaaagaattgatcccaaaaactaaagaggaggatattaaaagcattaaaaacaat

tcaattatggtagttaacacaagagcaatggtaaaggcgcaaaagaaagtaaaggatacaccaacagctg

gaagaaataaagaagaagtaactaatcaagtattttggaagacagatagaccgtcagaagttaataagat

tttgaaaattaagtcagaagttaaaagaaatggaatagaaatcaaagtatgtagtcacaactataataag

caacttggaaaaagggttatccctcttaacaattctaaaggccaaaatggaagtcaagaaatagaacttg

ctcttctagatatatgccaaattgctaagaaatttaaccgagacaagttagcaatatcagttgaggacaa

attattcgaatattactctcaattaaccataaaggaaatagcagacacagcaattttcggttacgagata

attttgtttgatccacctaaatggataacaagtgagaaagaaaagttgaaaataatggaagaatatcatg

ccatacccacaggaggtcatatagggcaatacagattatatttaaaaattcgtgaaaaatttaaatggaa

taatatgaagcaagatataataagttacgtgcagaaatgtgaaatatgcaaagtcagcaaagtaaataag

cacacaaaagaaaaatctataatcacatcaactccagcaaaaccatttaccatcatatcaattgacacag

ttggtccattacccaaaacaataaataacaacagatatgcaattacgattcaatgcgacctatcaaaata

tatagtgatcattccaattaaaaacaaagaagcaaacacgattgcgaaagcattggttgaacattttatc

ttgacttatggaagctttttggagcttagatcagaccaaggattggaatataacaatgaggtgttaagta

aaatagcccaaattttaaaaataaaacaaacattttcaacgccatatcatccacaaacaattggagctct

agaacgtaatcatagaagcttaaatgaataccttagagcatttactaatgaacatcacgatgattgggat

gattggataaaattttatgagtttgtttataacacaacaccacatacagaaacaaaatatactccgtttg

aattagttttcggtagaacagcaaatctaccacaagaaatttataaacataaaatcgatccagtttataa

tatagaacaatattacaatgaaatgaaatttaaactacaaaagtccaatgaaattgcacgtaggaacata

attatagaaaaagaaaaaagagaaaaagaattaaatcagcacataaatcccatagatgtaagcataggag

acttggtatacttaaaaaatgaaaatagaaaaaaattagatccattgtacttaggtccatttataattac

gaatattaaagatccaaattgtacaatcaagaataagcatacgcaaaagactacaacagtacataagaac

agattaatcaagaactaaatgaataacgcaattctttcgctcattcactcaatcttacgttattcacaaa

aacgggggagg

>gypsy16-ltr_ag gypsy anopheles gambiae str. pest

tgtagcataggttcgacacccgacactattagacttagcaaattagaattagattagaatagcaaagaca

caattattagtataacatccgctataattatctgcagtaaattcattacatcctagacacaaaccgcact

aaggctagaatgaaacagctaatgatagaatgaaatagcatgataagtggcgtgatcgtattcaccaaca

cactcaaaagaaatagtttttacaagtataaaattttagattaaacattatcagaacataaattactgta

attagatgcacacaaatattagacataaaagtgcaatatgttctaatccgaaccaactagataaataggg

aaaatttaatagagttcatccaattgcgcagtaggataatacacatggaaggacgatgttacctaatgta

cgcaaaaagtcacgtacgcataatgcaggagatagagcaggataataaattgtaatcatgcataacggag

cggataagctaaatatgcccataagcgcaatgtaattatttcgtataaaaggaagctcgcgcgtagctag

aggagagttgttgtatcagtaaaatcacgcagtccagtccgccgcgcaaaatttagttcgcaaatcatta

gaagccaaccacgctattaagtttaagtaaagtggcaaaaataaagtgagtttatataaaagtgcaaata

caatccgtctttagtcaggaacggcactaagtgatgacaagttcactaca

>gypsy17-i_ag gypsy anopheles gambiae str. pest

tggcgaccgtgacagcgtagcaaaaacaaagctacaagaacgttaaagtgcagtgaaaaaaataaaataa

aagtgcagtgctgtggagaaaaagttatacgcaaagtgcggtgcgtggaaaaaaatacagtagaaagaaa

aaggttaaagaaagaacggtgtcagtttttaccacataaccattatcgtgagaactcaatacagtgcccc

aataatctaataagcaataaagtgatctttaatcgatttttaacaaagtgcgagtggtgcagtttgaggc

ataaggtaacaccaaacgcgggaaataaataaaaacgttccaagtgcaatctaaaagtggcacaagttta

aaagttagcaaaaacacatcgttgcgcacaagtatcaagtacccgaaaagcagtgcaggaagtgaaatcg

cagtgtcagtaaactgaaaaaagtagcgagtgtgtatatttattctggcataacacacataagctatggg

tcacgataactcaaaaccggaaacaaacgttaagggcaataacgacctcaccatcgttcagacccaaaac

atacacactgaacaacacgaggcacacgagttcaaacttaacctgattttggctctgctcgcgttgattg

ttattgcaaaagcgctcaaaatagtgtacaaaatagtgaaaaatcaggcgaaagaacaagctgtaaagat

gttgtctttacctaaataaatagtgaagaaaaaaaaaagaaaaaaaacattagtgaagcaataattcgga

tataaaagacaaagtgccgatcaagtgttcatcagacgacaaccacaagccgcaaaccaggaagacgcac

actgcccacctgcaagccgccgtagccgcccgcctacaaggaagtcgtaaccgcctaccaggaagaccaa

gcgcccaccagcaagcgagcgcagccgcttaccaagaagaccaagccgcccaccagcaagcagcctacca

ggaagaccaagcgcccaccagcaagccatcgcagccgccactaactgaggagatgagcacctggcacggg

ctgagaaattttaacgaagacggtgaatacgtggggccatacatcccgaacaagaaggatgtcgaggatc

aacgaatcgagatcctggagttactgaaaaacgccggcaacaaggagatagaaaacaaggtagaacacat

atatactagctataaagaaaataaaataacaactcgggagacatttgacaaagccacagatgtcttaatg

acattattagggctgtaggaggaataaaaacaattaaataacaagaaaaaaaatttttttttcatacata

ttattaacaacaagaaagaaatttttagtttttttttactatcaataaaaaataaaataaaataaagtct

tcttttacaaaaaaaaaacttatacaaatccactcgcatagaaagaaaataacatttacgtttgtccacc

ttggaaaatatttcctctcactttttccgcttttaaattttttttctaaatactattcacaattagaaac

tcaaaaaaaaaatcaatcaataaataaataaaataaaataaagaattttcttcttttttctcaacatata

aaagaaagaaactttcctcgactctcgaggaaaaccagattttattttatataagcttttatataaaaat

ataagtctgctttctctagaaaaaataaattgtacttgattttatgtatgaacttaaattttttttccca

caatcattgaaatccaaatttcgaccatatgactaaactaaaagaaattgttaggagactagaacttatt

cacaaaaccctacagcaaaacaaagggaacattagacagtgcgcattggccacatacagattacaagtag

acgaaatatattccgtgttcaggaaagaaatagagaccaactacgacaaatacagcgactcagaaatcaa

gttctacaacaacattatccaaaatttaatcaccaacatagtagaaaaagttaacaacgagacaattaac

accgacaacaacactagcgacttgaatgagacattaaaatcacacaaaaaactaacgttaaaaaccacag

ctcacgttataatatcaattttatctatatataaaagacaaaaacaaatcgttccgacagcgaacattga

ccacacagctattaaaacaaatacgagcgtagactcaagttacaaaccaaacatggatgctttagagatc

ctaaaaacggccaccagcctcatacctacatttagcggtagatatgacgaagctgaagccatgttagctg

ctttagaaacaatgaaagaagcagtggatgaacagcaccacagactaataatgcgggtggtacaatcaaa

actaaaggggaagggcagaaaaatcatcggaaaaacggtgaccaacatagaagacgctctggcaaaaatc

ggtgcatatgttaagaaaacagagtcgccagaagatattgccaccgcaattcatgcattaaaacaaaaaa

ctacaccaaaggattttggtgaagaaatacaagctctggcagaagagctagaacaagcatatcttggaga

ggacgttgcaccagcactggcaacggcaaaaaccaacaagatagcaatggcagcttttggaaaagggctc

aaaaaggaaatccatcaggcaatagtattgtccggtaccattcccacccttgatgcagctattagagcaa

ttattagcatcgataagacgaatcaaagcacgcaggacaaaaagtcagacaatagacagaatgggcagga

gaatagatacagctcgaatcagcgtcagacaaacactcgtggtatagaccaacgtcaaaacaataacaac

tggagattcccaccccagcaaaacaataacagttggagatcccaaccacagcaaaataataacaattgga

ggtcaccacaaacacaaaacggtagacagggagccgggttcaacacccgaaacaggcaacccgccggccc

aaattttttagggcaacgcgaaccgcaaagggcaatcctttacacgcaaatggagacccagaacccacag

gtggaccagccctccacaagccattacgggcaacatacacaataaatgtgcaaaaatcaaattttattag

gacaagattaggtctagcagattcaatatgcaacctatttgtagattcaggttccgacatttctatcatc

aaaggcaacaaagtaagacctacacaaatttataaaccaaaagatatagtggatatcataagcgtaggag

aaggaacaataaccactcatgggtccacaattacggatgtaatcgtggagggaaagaaaatccaacaatt

atttcacatcgtaccagataacttcaagataccggcagatggtatactcggtagagatttttttatgaac

caccgatgtataataaattacgatacttggattttctctgtaaaacacaatggagagtttttggaagcac

ccattgaagatactatcaatggcaaaacactcatacctcccagatgtgaagtaattagaaaacttgataa

gttaaaagaattagatacagatgcggtagtatgcgcagagcaactgcaagaagacgttcttgtaggtaac

tgcattgtaaataaaaactacccatttattaaaataatcaatacttccaataaagctaaattagtaaaca

ttagccatatcaaaacaatacctttaaatgaatttgaaatagtaaaaactagcaatcataaggatgaaaa

taggttagcaatcataaaggatttaatccgaaaggaaaatatttccgaagatacagataaatcttttgaa

caattactgttaagctacaatgatatttttcatctacctaacgatcatttaactacaaataatttttatg

aacaagatataaaattagaagataaaagacccgtgtacataccaaattacaaacaaaaccattcccaagg

accagaaatcaaaaagcaaattgaaaaaatgcttcaagatgatgtaatagaacactcggtgtcacattac

aattcacccatcttactggttccgaaaaagtcctcagatgagaaaaaatggagattagtagtcgatttta

gacagcttaacaaaaagctgctccccgataaatttccactacctagaatagactccatattagatcagct

agggcgagcaaaattttttagcacattagatctcatgtcaggattccatcaaataccactggaagaatcg

tctaaaaagtatacagctttttcaagcacggatggtcactatcaatttaaacgattaccttttggattga

acatttctccaaatagttttcagcgaatgatgaccatagccatgacaggcctcacgccggaatgcgcttt

tgtatatgtcgatgatattgtagtagtaggagcttcagaaaatcaccatctaaagaatttagaaaaggtt

tttgaaagactaagacactacaatcttaaactaaacccagaaaaaagttgctttttcaaaaaagaagtta

cttatcttggacataagataaccgacaaaggcatccttccagatgattccaaatacgacagcataaaaaa

ttacccgataccacaaaacgcagacgatgcgagaagatacgtagcattctgcaattattacagaaagttc

atcccaaactttgctttgaaagcaaaaccgcttaacagcttattaaagaaaaatacaaaatttgaatgga

cacaagagtgtcaagaagcattcgaatatttaaaaaacacactgattagtccacagatattacaatatcc

tgacttcagcaagcaatttatactaaccacagatgcttcaactatagcatgcggagcagttttagcacag

gaacatgatggtatagatatgccgatatgcttcgcaagtagaaccttcacgaaaggggaagcgaataaag

caatcatcgaaaaggaactagccgcaatacattgggctataatgcatttcaagcattacctatacggtac

aaagtttaccgtcaaaacggaccatagaccactagtctatctgttcggaatgaagaatccgtcatcaaag

ttgacgagaatgagactagatttggaagagttcgattttacagttgaatttgtaaaagggaaacagaacg

ttgtagcagacgctttatcgcgcatcaagatcacctcagatgaaattaaatccatcaatgtgattacgag

aagcatgaacaaacctgttacttccgataatgttttaggaaacacgtcagagtctgatcaactcaaaatg

ttccatgccttagcatacgacgaagtaaaagacttaccaaaactagaaacatcagtaaagagaaatgaaa

acactatcgagttgataggaaaaatcctaaacaaaagaaagtccaaggagctcttatcagtaagagacat

ccatctgaatacagatataggactacaggagcctttattagtaaaggatttccagaaaaggaaggaaaaa

tctgccatagtgcaatttatcaaaaatatagaaaagaagctcgtaatgaaaagcattacccagctagcag

tctctgaaacagacgaaatattcaaggaggtaaatccgaatgaattcaagcaaatcgctaacaatcacct

gaaaaatattcagatactaatatatactaaaccacaaacgataaatgacgaaaagacgataaatgacata

ctcgacaaagtgcacaacacaccgacaggaggacacattggacagtatagaatgtataagaaaatcagaa

aggaatatgtatggaacaaaatgaagaaatcaatcaaagattttctagacaaatgtataacctgtaaact

aaataaacatcttatcaagactgtagaaccttttgttaaaacagatacacccaacattccattcgaagta

gtatcaatcgatacagtaggaccatttcaaaaaacaaataacaataatagatatgcggtaacacttcaat

gtaatttaacgaaacacgttacggttatagcaattcctaacaaagaagcaaatacggtagctagagcagt

aatagaaaaatttatgttaatatatggcacaaatattaaagaattcagaaccgatatgggtacagagtac

aaaaatgaaatatttaaaaatatatcagaaatccttcgaatagaacacaaattttcaacgccatatcatc

cacaaacgataggagctttagaacgtaatcacagatgtctaaacgaataccttagaatttttacaaacga

acacaaagatgattgggacgattggataaattattattcatttgcatataatacaacgcctaatttagac

cacggttacacaccatttgaactagttttcggaagaaacgagaaaatttcgacaaatataacggaaaaat

ctacaccattatataattacgatgattactcaaaagaatttaaatacagattaaaactagctcacgatag

gactcgaaaacatatagaacaagaaaaaatgaaactactgaaagagcaacaaaacataaaccaagttaat

tttcaaataggagatcaaatagcattgacaaatgagaacagaacaaaactagatccggtatataaaggac

cgtataaagtaaaagagattaacggacctaacatgataattgaaaacactgatggtgtcacacagaatat

tcacaaaaatagagcaattaaaatatgacagaataacttcatttcattacgttattcttccgaagggtgg

agg

>gypsy17-ltr_ag gypsy anopheles gambiae str. pest

tgtagcagatttactgctaacagtaggtcacatctaacttagcattgccaaaagccaggtaaaaagacgt

taaccaccagaagcgcaagcgcatagccagaagccaggtaaagaaacgttaaacaccagaagcgtaaacg

ttctcgtgttttaccccagaacataaataaggaaaacgcgccacagataagcaattaaacttccgtaaaa

cccaggcataaacaggaaaacgttcgctacatccacaatgcacggatagttgataaaacacaggcacgct

aggtataatttaagaaacacctgtaaaaacccaaacccggaaaaccaaatgaaagctcacatttgacaaa

catctggttgccaaatgtgtcacaactttcacaccatctttcttataaataaagctcgaattgatggcga

agtcagttcaccttccatagacacgtagatcactcgtgttctggtgcatctcaccaatttgcagattccg

ccgaaggctctgctcatatttgataatcatttttggttatcagctgttcagtcggttgaccgatcagcat

taccctcatcgaaataaagtgcacgtttgcactagttccacccaacttctcccacggtgtccgattccgc

ctcggtcatcaactcgacgcgcaaggtcgatccagtccgcgattccgccgacgtaagcaagtgcttcttt

tcggacttagcgtaagtgtgaacaggttgacgtaaccgatacgcgacgaatgtgtttacattttggtgtc

gcgttccgtacgatccccttcctcgtcccacaccctttcactgtgctccgcgcattgactcaccgaaacg

gtttgagaagcgcggccgaacctaccaca

>gypsy19-i_ag gypsy anopheles gambiae str. pest

attggcgacgagtgacaaattctgagaaccctggaacgctagcgctacgcagttaaagaaaagctgaaac

aatcgggtataagtggagacagtgtcatgatcgaagagaaaccaacaagcagtggtacaaagcagaacat

cgcacagagtgaacaacgcaccacaatggcaaaattcgatatggaaccttttaacaaagggctcatgcaa

tgggcccgctgggtgaaacgcttcgaaggagcaatgtcggtatttgaggttaaatcgaataataaaaaag

ccatgcttctgcattacatgggtgttgattcatacaatttattgtgtgatcatatttctccggaagaacc

agaagacaaaacatacgaacaaatagtgaagtgtttggatgagctgttcgacccgaaacccctcgaaatg

gtggaactatggaagtttcgtcaacgacttcagactgaaggcgaaactgtaacggagttcatcacggctt

tgcaaaaagtggcggctaattgtgatttcgggcaatatttgacaaaggcgctcaggaaccagctagtttt

tggtgtacggaatccaagaatacgcaaccggctgattgaagaaagaaatctaacactggaaaaagctaag

caaattgctttagccatggaagccgccggagatggcgctgaagtgctgaatagcagaagtgctgaagtgc

tgaatagcagaggtgctgaagtgaaagaagtaaacatcgtgagcaacacaacgaagaaaaacgtcgagtg

ttatagatgtggagaatcacattttgcatatgtatgcaagcacaaaagaaccgtctgcaaaaagtgcggg

aaaattggacatttgcagcgcgtctgtcgcacgaacaatagaagcaaaagtgtgaggttaattgatgaac

aacatcaagacagcgaggagaacgaagaggtaaactcaattttgatcaacaatttataccaaaatgcaaa

ccataccgcaaaaatatacataacactaaaagtcaataatactaaaattcaatttgaagtagattcagga

tccccgttttctattatcagtatgaacgacaaacaaagatggtttaaagatatccctattagagaatcag

atataaaattacaaagctattgtggaggatctataaagttatttggtacaattagtgttatagtggaaaa

tgctaaaacaaaattaacactatttgtggtagagtctaaaagaggacctattgtaggaagaacatggatg

cgagatttgaaatttgattggaacgaattattaagtaaggggaactcgtacgtaaatcaaattgtcaccc

attctaatactaatcaagaagtgataaaaaaattaaaagaagaattcgaggtagtctttcgtaagtcatt

aggagagatttcaaatattcaagcatctcttatcctaaaagaaaatgcgttgccaatatttctaaaaaat

cgtactataccatttgcattaaaagaaagtgtcgagaaagaaattaacgatttagtaaatcaaggaattt

taataaaagtcaatcgcagtgaatgggctacaccaatagtacccgtaaaaaaatcaggaaaccgtgtacg

attgtgtggagattataaattaacggtgaacaaaaacttggtaatagacgagtttcctttgcccacaata

gaggagctttttgctaacatggctgggggagaaaaattctcgaaaatagatttagcgcaggcatatttac

agatgacagttaaacctgaacatcaggaatttttaacactgaacactcacatgggactattcagaccaac

acggttaatgtacggggttgcttctgccccagccatattccaaagagaaatcacacagattctccaagga

attccaggcgtttctgtatttttagatgatgttaaaataacagctcctgatgacaagacccatgtggaaa

gactgcgcactgttttaaaacgatttcaagaccataatatgagagtgaatgatagcaaatgtaatttcct

tgcagattgcatagaatactgtggatacaggatcgacaagtacggtattcacaaaatgaaggagaaaatt

actgccattcaactaatgccaaaaccaaggaacaaagatgaggtaagagcttttgtaggccttgtgaatt

attacgcgagatttatcccaaatttaagtgaaaagatctatcccataaataatttacttaaaaacgaaat

tcctttcgaatggcatgaaggttgccaggacgcatttgaatggatcaaaaaggaaatgcaatctgaacgg

atcttggtacactacgatcctagcttaccaatagctctagcagtagatgcttcgccctacggagttggcg

ccgttttaagtcacatctatccagatggcaaagaatatccaattcaatacgcatctcaaacactatcacc

aacccaacaaagatatactcaggtggataaagaagcatatgcgattattttcggagttaaaaagttttac

cgatatctttatggaagaaaatttatcttaataaccgataacaagccagtttctcaaattctatcaccac

agaaaggtttgccaacgctatcagcaactcgcatgcaacattacgctgtatttctagaatcattcaattt

tgagattagatatcgaccatcaaaagaacacggaaatgctgacggaatgtcacgattgccaatccgagac

attcagctggaggatacagaagaacccgatgaaattgaactaaatcaaatagaaaatcttcccgtatcag

tagaagaacttagtaaagagactagcaaagacataaatgttcaattactaatagacggattaaattcagg

gagaacagtatccattaacgatcgttttggaatcgaccagacacaattttctcttcaaaagggatgtctt

atgagaggcgctagggtctacgttccacctcaattacgaaatcgagtcttagacgaacttcatgaaggcc

atttcggcatatcacgcatgaaatctcttgctagatcttattgttggtggaaaaatatggataacgacat

agaaagattatcaaaaaactgtgtttcttgtgcaaaagtaagaaaagatcctcctaaagtaccaactcat

gtatggaagcgtccacaatcagtttttgaaagagtacatgcggattacgctgggccatttatgggtatat

attttcttattctagtagatgcgtacagcaaatggcctgaagtaaaaataacaccggatatgaatacaga

cactactatagacaaaatgcgagaaatttttgccacttttggcttgccatcaattttagtgaccgataga

ggtacacaattcatgtcagaaaaattccaaacattcttaaaatctaacggaataacccataaaacaggag

ctccttaccatcccgcaacaaatggtcaggcagaaagatatgtacaaacaattaaagataaaattaaaac

tatgcaatgccataaatctgaaattccaaaaaagttacaaaacatactgctagcgtacaggaaaacaact

catccaagcacaggggaaagtccctcgcggttaatgttaaacagacagatacggtctcggcttgatgtta

tggtaccaaaaatcgaaaagaaatccaatcccgaagtagtacacaaaacgacaagatcatttgcagtaaa

cgaaagagtagcagcaagagatttcctttcccagaccgaaaagtggaaatttggaacgattacaaagaag

ctaggaaaactgcattacgaaatacgattagacgatggaaggatgtggaaaaggcacataaaccaaatga

gatctggtcctgaagaaatatcaatccaccatagtagtaaccaaaacatagaaccttggatagatgaatc

agtttatctgccagataggatggaagagtttcaactccctcatgattcagaaacggagatggttagatct

gataatatcaatgaagatttagcttgtggagaagaatctcaaactcaagttaggagatcaacaagaactc

gacaaccaccaattcgataccgtgattagggattattactttaatttagaatataaatatgaattgtctt

tcacactttagcaagtaatcactttatattgttaggaggagagagt

>gypsy19-ltr_ag gypsy anopheles gambiae str. pest

tgtcatatacgtctgacagctgtccatcgacagaaactgctagttgggtagcaaacagctgtcagtgaga

ataaacggcactctgtgtctgaactacgaacgaaacacatgtgtcttccttccacgagttataaca

>gypsy2-i_ag gypsy anopheles gambiae str. pest

actggtgaccccgacgtgatcgcgtgcgcgagtgagtgagtggtaacctgacgaacaccgtgtccagccg

agaaaaaacgtgtttccattgttccacggtccggaccgacggcaacgttcccccccatcatcgaggagcg

gccgaccacgaaggaggcaccacgcaagcgcagccagcgaaaaaaaaccccgtgcacaaaccccgaaccc

acgtgagtgcaaatcgacaccgaaggtggccgacagtgaggaacactgttcaggaacatttttacccgac

ggagcgaccgatcctagcggaaaagtttcctctcggtgctgagcgatcgccgaacattttgctgacacac

cccgcgccgtgtcgcacacccgccgagcattttggtacccgtacgtgtttgcgcacccgccgatcataac

ctcacacgtaccgccgagcgcgctccagacccacgcggtttttgtgtgtgcaccgtgtgtgtgtgtgtgt

gtgtgggtgaatgtgcgcaggccgacgccgagcggattgcgtcagaattttgctcgagctacgttcgtca

tttttttcgaccgtgcaccgaagacgtcgtcagcgcacgcagccatcgttctcttctcgccgacaccacc

gaccgaacgccaccgaagatcatcgcccctcgtttctcacaccaccggcgtcatcgacgaacgcagccaa

cgagcgactaatcctaacacgatcgaccgcgtgtgcggatttttcgtcgccgaaggatcgacctagccaa

cctccagctggacttgcttgcgcccccgccactaaggtaagatccacccttttttaactaaccttagtcg

taaggatgttgcacagtccgccggtccgcgacgtatcgactcccgatggcgtaaccccgagtgccgatcc

agccgcgagtggatccaaatcgcctcacgtaccaacaccgcccgttccgaataccccgcgcgtaccaggg

ccgtccgcctgcgacgccatgtttatgccgcccgaatcgcagattgacactttgaatgccatgcagctga

aaccaccggagatggacaccactgacattcaaacctttttcttcgcattggaaaactggttcgatgcgtg

gaatatcaccacgaaccaacatattcgccgttttaacattcttagaacgcgtataccgcttcgtgtcctt

cctgagcttcgccccctgttggagaacattcgacagtacgctacggaccgttacgaggtagcaaagcgtg

caataattgagcactttgaagagtcgcaacgaagccgcttgcatcgtctgcttgccgaaatgaacctcgg

ggaccgaaaaccatcgcagctattagcggagatgcgccgcgccgcaaatggagcaatgacggactctatg

ctggtagatttgtggatcggccgtctcccgccatacgtccagtccgccgttattgccactaacacggata

ccaacgatcgagctaaagtagcagactctgttatggattcgttcgcgttataccaccgaacgggcccgta

ccaaaccatccacgaagtacgcaacgaggacttcgaacgtctttctcggcacgtaacggaattaggtcag

cgcttggacgccgtactgagcaagctcaacgaacgagaacgcgcgcgaccacgctcacgtacccggcaac

gtcaaccgaaccaggatgcggtaacacccagcggacactgctattaccacacgcagtacgggcaagcagc

gcggaactgtcgtgccccctgctccttcaacaatcggcggcagggtagtaactcggccactgcttccgat

tgacgcttaaccagaggccaacctcaacagatacacgtactttcgacccatagctatcgtctcgtaataa

ccgatccaaaaactaacatcaaattcttaatcgataccggtgcagacgtttcagtaatccctcgacaaca

cagttccgtcccgagtaaaccctccaccatgaagctgttcgccgctaattctacaccaatccaggtttac

ggagagtcgctctatactctcgatttgggacttcgccgatctttcctttggaacttcatcatcgcagacg

tggggacagcgattattggagccgattttctccaacatttccatctgctcgtggacttgcgcaaaaaatg

tcttgtcgacgccttaacgaacgtacgttctaccggagtgccgagccaaaacccgtcggaaccaaccgta

aaagtatgtgattccacctcaccgatcgccactctcctaaaggaatttcccgggttaactgcactatcca

ctcctggcaccttactgcagtccgaagtgacgcaccgaatcgaaacgacggggcaaccaacattcgcaag

acctcgccgattaccacccgaaaagtacgcagctgcccgcaaagagttcgaatcactcgtccagctcgga

gtgtgccgcccctcgaatagcagctgggccagcccgctacatatgacaaaaaaggccgacggcacctggc

gcccttgtggtgattaccgcgccctaaatgcaaaaaccgtacccgaccgttatccactaccgtttttaca

ggacttcacgatgcatttgcaagacaagatcatattttccaaggtcgatttgcacaaagcataccaccag

ataccaattcatccggatgatatagcgaagacagccatcacgacaccctttggactttacgagttcacta

ccatgcctttcggattgaggaacgcagcgcaaacattccaacgccttatccatgatgtcctacgaggact

cgagtttgttttcccgtatatcgacgatatgatcgtagcatcaacgtccgaggcagaacaccacgaacac

ttacgccaacttttcgaacgattggagaagcaccaactagccatcaatccagccaagtgcgagttctacc

ggaacgagatttcctttctgggccatctggtcaacgcttctggtattcgtcctctccccgatcgagtcca

agccatcagcgagctgccacagccaacgacgattatggagttgaagaagttcctcgccatgataaactac

taccgacgttttctgccgcacgccctggaaacgcaaggtatacttctcgagatgactccaggtaacaaaa

agaaggacagaacgccattaacctggtcgctagaagcttccgaagcattcgcccaatgcaaagagcaact

gaaacgtgcaacgttattggcacatcccgtgaagaacgccgaactttctctatggaccgacgcttcagat

ttcgcagccggagccgtacttcaccaacgcaccaacgaagacctgcaaccactaggcttcttctcgaaac

gtctcgaaaaggcacagcaaaagtactcgacctatgaccgagaacttaccgccatctatctcgccatacg

acacttccgataccagctagagggtcgggaattctgtatttatacagaccacaagcctctaaccttcgcc

ttccgacaaacgcacgacaatgcctcacctcgacgagcccggcagttagacttcattggccagttttcca

ccgacatccgtcacatcgccggaaaagacaacgttacagccgatctgctctcccgcatagagacagtgca

cgcgacaccgaccatcgattatgagcgattagcagaagaacaagagcgcgaccctgaactttccgacatt

ctcagtgggaaaattcagacggacttgttcctgcagaagacaccaataccgggaagccccaagtcactct

acgccgactgccctggaggtatcatcagaccgtacatcacccgatcgtttcgaacacaacttctccacgc

cgtacatgatctcagtcatcccggagcccgcgccacagctagactaataacagagcgtttcgtgtggctc

aatgcaaggaaggaatcccaggacttcgctcggaactgcttagcctgccagcgcgctaaggtaggaaggc

acgtcaaaagccccttgataccgtaccctgcaacaacagcgaggttcagtcatatcaacgtagacatcat

tggaccatttcccatcagtaacggtaaccgatactgccttacgataatcgaccgatttactcgctggcca

gaagcaataccgatctcggatatcaccgcatctaccgtcgtatcagcactactattccactggatcgccc

gattcggagttccggcgcacgtaacaacggaccaagggagacaattcgaatcctccttgttcaaagagtt

gacgaaagccctaggaacgaaacacatccgtacgacagcctatcacccgcaggcaaatggaataatcgag

aggtggcaccgcactcttaaagcagcaatcacctgcaaagacaccgcaagatggagcgaacacctaccgc

taatactgcttgggctacgaaccacgttcaaaaatgacatcaacgcctcgccagccgaacttgtgtatgg

aacgacgttgaccatcccggcagaattcttcatcgcgaaaccgcaaaatgccctcgccgaccaatccgac

ttcgccaaaacgttagaggagacgatgagcagcattcgaccacagagcaccgcttggcataccaaccgca

caccgttcgtgcattccgatctgaacaagtgtactcacgtgttcatacgcgacgacaccgtccgacctgc

actaactacaccttaccacggtccatataaggttcttacacgcaatcctaagtcttttcagatactccta

cgtggacagccaacgctggtttcgatcgaccgcttaaaaccagcgtatggcgcagaagaggaagccaccc

cggccccgcagtgctcgtgggaagggctaacgacaaacctgctgccgccaacaaccgaccactcggaaac

tctgccgttaccggacgtccaggcaaattcggaccgcagagacgccaccgcagcctccaaaccgacgtcg

cgcgaacaaccagtgcgtaatcagacgacacccgcaccaccatcgcacccgacgacatcgagacaaaccg

accgagccgccgtcgacgccccaccaccctccatcctacgccgcaacgaccagacggtatcgaccggcgt

caccaggtctcagcggaaggtcatcatacctctacgttaccggtgacaccgctctaggaggggagtac

>gypsy2-ltr_ag gypsy anopheles gambiae str. pest

tgtagcgaccagaccgccatctggcgtgagaatcgtgagcgatcgtgacatccagggacaaagacacgga

tctccgtggagcgcactcaccaggcacacgaatgtgtcaaagtgacgtgcgccgcgtgtccagcgctaca

cttcctgctgtcagcgagcacattctctctcttgcgacccaacctcgaaagcgaacagacctcttccttc

gctccgcgctcgaaacttcctcaacgtgaaaccctctcgcacgaacgtgcgcaaccgttcataatatagt

gtaaaataaagttccgtattacctactcacgaaacccaacgcgttcgcgacataaaaaattagggaccac

ttttgtggcgcccctaaa

>gypsy20-i_ag gypsy anopheles gambiae str. pest

atttggcgacgagaagtacgaagctacgtgaatgtgctaggcgcaatacatatacaaacaagttgctgca

tcgtgaatttggctgtctactgcgtaattttacatcacaatggcgcttcctaacccggttgtcgacgatc

cggcagtcgccagtcccgctttgcctgctgtgagtggtgctgcggtaccagttacatttcatttcgagcc

attcaatcctgcttcatcgaaatttgaccgatggttaaatcgactacaaatttcattccggatttaccac

gtgcgtgaagccgataaacgcgattttctgctacactacatgggcggccctacatacgatgtgctgtgca

ataagctgaaaaatgctgagccacatacaaaaacgtacgacgagattgtagctctactgaaggaacatta

cagtcctactcctttggaaatactggagaatttcaagttcgcgagccgtaaacagctagagcaagaaact

ctaagcgattacctgatgcatttggagaagctcgcccaaacatgcaatttcggggactacatggacaagg

ccctccggaaccagttcgtttttggcatccagaaccgtgtgatacagtctcgattgctggaagtgcgcga

cttaaccttgacaaaggcaaaggagatcgcattcggaatggaaatgtctcatcgtggaaccgatgaaatg

cacaactcacgtcaaaaaagtgaggttcagcacatcgagcatggagcaaacaaaactaaaaaaagtttcc

agtcatcgagccaagccagttccagtcaaagttccggtcgcctgtcgaacaagcagaatggtggaaataa

acgatgttatcgttgcggggatcctgaccactacgcagacaaatgcaaacataaagctacgatctgcaaa

tactgcaagaaagcggggcatcttgagaggatgtgtctcaccaagaccaacgagaaggggacggatgacg

cacatcacctggaggagcagccgtgtgttatgaaggatgtgttacacctgaacgcgatccaaggtattgc

tggtaagtttttgttgagtctgtggataaatcaaaaacagctaacgttcgaggtcgacactggttcaccc

gtatccttaatcaacctacaagacaaacgaaaatactttaacaattttgacatttcccctactaacattc

gactcgtgagttactgcgataatgacattggtgtgcttgggaaaataacggtaaaagtagttgcaaatgg

tgaggaatttacattgcctctacatgtcgcagaatctagtagacatccgttgttagggcgtgattggcta

cttgctttgaatttagatttcaatcgtgtattccaaccaggtacacattcagtttcctactgtagtggca

aaaatcagtctaccactaatgcattgaataacttacttacaaaattttcacgtgtctttgatgaacgtgt

tggtaaaattgaaggaatacaagctacacttactgttaggaaaaatacaaaaccggtatacataaaagct

aggccagtggcatttgcagtgcgcagcacggttgataaggaaattgatcatttcgtgaaagaaggcatat

gggaaaaagtggaccactcagagtgggctacacctgttgttgctgttaggaaagccggaggcaaagtgcg

gttgtgcggcgattacaaaattactcttaacccaaacttactggtggatgaacatcctcttccgacggtc

gaagaactttttgctactgttgcgggaggggagacattctcaaaattggacctttcgcaagcttacttac

aactcgaagttcgacccgaggatagggacttacttacattgagcactcatagaggcttgtttcgtcccac

tcgactcatgtatggagttgcttccgcacctgcaatttttcaacgtctgatggaggaaattttgcagggc

atacctggcgttactgtcttcattgatgacattcgcgttactggttctgatacaaaaatgcatttactta

gacttgaggaagtacttaacagattagataaatatggattgcgtgtcaatagagaaaagtgtgacttttt

ctctgatcgaattgagtactgcgggtacatggtggacaagcaaggaatccacaaactccgcgaaaagatt

gatgcaatacaaaacatgcctattcctaaaaataaggagcaagtacggtcttttgttggactcattaact

actatggtagatttttccctaacctcagtactattttgtacccactgaataatttacttaaagatgacgt

tccatttgtgtggagtgctgattgtgataaatcgtttacattggtaaaaagggagatgcaatccgatagg

tttttagtacattatgacccgtcacttccggtaattttagctactgacgcgtccccatacggggttgggg

cagttcttagtcatcagtatcttgatggaactgaacggccattacagtacgcatctcagacccttactcg

aacgcaacaaaaatattctcagatcgataaggaagcctactcgatcatttttggtgttcgcaagtttcat

caatacctttacggtcgcaaatttattctggtaacagacaataaacctatcagccaaatcttttcggaat

ctaaaggacttcctactatgtccgcaatgcgcatgcagcattacgcggcattcctacaggcgttcgatta

taagattcgacatcgccgttcgtcggaacatttcaatgccgatgctatgtctcgcctaccggtttcaact

actgaccctgaatcggaaattgaagaaccggaggtagtcgaggtaaatgcaatacaaacactcccactga

ctgtagatgaattgagtgcagctaccctagcggatgtgaatgttcgcgaattgctacgtgccctaagaac

tggaaactcagttgaagggaaacatagatttggtgtgaatcaggaagaattcaacttacataaagattgc

ttgatgcgtggtagccgagtatacataccacctgcattgcgaagaaaggtgcttgaagaactccattcaa

cacatttcggtataacacgaatcaaatcacttgctcggagttattgttggtgggaaggcatagacagaga

catcgaaaacctggtcaacgattgtgcttcctgtcaggctgcaaaggctaatcctcccaaagtcactttt

cattgttgggaaacacctacggaaccgtttcagcgtgttcatgcggactatgctggcccattcatgggac

tttactacctcatattaattgacgcatactcgaagtggcctatggtctacgttgtgaaaaacatgactac

ggaaacgacaattcgtttgtgccgggagtttttcagtacttatggattaccttctgtctttgtgagtgac

aacggtcctcaatttacctctactgaattttcaagatttcttaaactaaacggaattactcataaactta

gtgctccgtaccatccagccactaatggacaggctgaaagatttatacaaacaatgaaatctaaactaaa

gtcgctacaatgtgatcgaggggaagttcacagtgaaatttgcaatatactgctctcataccgtaaaatg

attcacccagccaccggattttcaccttcaaaattagtgttcggtcgtcagatccgttcaaggctggatc

tcatgataccatcaaacgatccaaactcaaatgaagttcagtctaaaatacgtgcattgactactggatc

aaaagttgcagctcgagaatacgttcacggaaacaagtgggagtttgggacaattaaagaacgtctaggt

aaactccactatttagtgaaacttaatgacgggcggacctggaaaaggcatatcgatcaactacgtagtg

ttggtgcagggctatcggaatctactaaggaagaaatttctttgcgccgtgaagagattggtggtgaaaa

tttctacgacaacactatcgcagtcactccagacattactacaaatacatacaactacgataacacttat

acatccactgacatgtccctccctgctatcccgactgctcctaatcttgaaacgggtatccaactaccga

tttctaacgaaacagctgcaattgatcaacccacagggtcagcggacttgccggtggaccaaaggttgcg

tcgttctctgcggaccatcaagcctccgcaaaggctcaacctataacaacgaattctattttgcgcggaa

gagc

>gypsy20-ltr_ag gypsy anopheles gambiae str. pest

tgttatatctgagctacacttctggcagcactggagctgtcatacgctattccccatacacaccgtggta

tgaacaactaccttccatacacaccgtggtatgaatagctagattctatatacactgcgatcgcttccgc

taccgttctacgacacttgaagaagagaagccgaactacaagcactacaggactacaaatatatttagat

agagataaaacagttcggttgtacaatttacttaattacggttccgccccatctacaaca

>gypsy21-i_ag gypsy anopheles gambiae str. pest

tttggcgacgaggaaaagttcagaagcatctcgaaggtctttttagcgaaatcatcaaataacgaaaatg

tcccaagaggatctgcgaaacgccatcgtccaactcacgaatctcgtcgcgaagcagcagcagcagatcg

aaaatttagcaaatcgaactttttcgacggcggctagcggaagcgagaagacaatcgagtcgttggcaaa

tggaatccaagatttcctgtacgatccggacgcaggagttttctttgatgcatggtacgctagatacgaa

gacgtcttcattcaggatggtcattctctcgacgatgccgcccgtgtgagattgcttctacgcaaattaa

gcacccctctgcacgataagtacgtgaacactattctgccaaagcatcctcgcgatttctctctggatga

gaccgttacaaaactgaagaagttgtttggtcgccagaagtcggtttttcactcccgttaccagtgcttg

cagtacgccaagagtgacgcggatgattttacttcgtatgctgccatggtcaacaaacactgcgaagcgt

ttcaactttccaagctcacgtcggatcaattcaaagctctccgatttgtctgtggactccaatcgccacg

ggatgcggacatccgagctagattgatttcgaaactggaagctgatgaaactgcagttgttgaacaagga

gaagctgcaagtaaggtgactttggaaaacctggtggaagaatgtcatcgtgtggccaaccttaagcatg

actcgcagatggtggaaaacaaggaggcttgctccgtcaacgccatttcgcgcaaccagaatcattcttc

ttcgaagaagaccaacaaagtgcccaagacaccctgctggaaatgtggggaactgcactacgttcgtgaa

tgtccgtttgcatcgcacatgtgtactagatgcaagcagcaaggacataaagaaggctactgttcaagca

gtaagcccgctgcatccaagcctttcaagcaatggaagcccaaagagagcatgaagacgaatggcattta

cactgttcgcaacgtcggaagaaaacgcaaattcgtctcggtcgagctcaacggggtagcagtcaagctt

cagcacgactcggcgtccgacatcaccatcatttcgaacgaaacatgggctagcatcggacgaccaccca

ctcaaccgaccgatgaatctgctgtcacagcgtctggtagtgatttgaatctcctcgcagagtttcaagc

cgacatcactattaacaacgtgaccaagacagggcgcattttcatctctgatagcgccgatctcaacgtt

ttgggaatcgatactatggatctgtttgatctgtggtccgtaccgattaacagcttggtcaacgtcgtac

atcaaaactctgaccaatatgttgatcgcctcaagcaccagtttccggaggtttttcgaagcacgctggg

tagatgcacaaaagcgcaagtgaagttatacttgaagcctgatgcccgtccatgctactgtccgaagcga

ccagtggcgtatgcggctcttcccaaagtagatgcggaactcgaaaggctcgaaaccaacggtataattt

ctccagttcaattctcggactgggcagcaccaatagtcgtcgtacggaagtcggacaatgtttcggtccg

tgtgtgcggtgattattctacggggctgaacaacgcgctggaatgtgaccgtcatcctctacctcatcct

gacgatcttttcgcggagctggctggggcacgctatttcacacaccttgacttatcagacgcctatctac

aagttgaggtcgaggtggaatcgcgcaagctacttaccgtgaacacacatcgcggccttttccagtacaa

ccgacttcctcccggagtcaagtcggcacctggtgctttccaacgcattattgacagcatggtggctggc

atctctggagtgaaaccttaccttgatgatatcttcattgctggccgcaccaaagaggagcacgaccgta

tcctctatgctgttctcgaacgcgtccgtgagtatggtttccatttacgccttgaaaaatgtcgtttcgc

gcttccccagatcggtttccttggattgatcgtcgacaaggacggtgttcggcctgacccgtccaaaaca

gaagccattgccaagatgccacccccgaaggacgtgaagcaacttcgctcctacctcggagctatcaact

attatggtcgattcgttccacagatgaagcacctcagggctcccctggatgacctgctgaaaaaggatgc

tcgctggaactggacaaaagagtgtcagaaatcttttgagcagttcaagaccattttactctccgacctg

ctgcttactcactatgacccatctaaggagatcatcgtcgcggcagacgcatcgaagtatggtctaggcg

ctgtcgtcatgcatcgtttccccaccggtgaggtgaaggcaatcgcacatgcttctcgctcactgacggc

agcggaaatgaactacggccaagtggaaaaggaagcattggcgttgatattcgctgtcacccgtttccat

aagatgctgtacggacggcatttcactctcgaaaccgatcatcaaccgctactcaaagttttcggctcga

aaaaaggaataccagtttacacagccaatcgtctgcaacgatgggctttgacacttctcctgtatgactt

cgagatcaagcacatctcgacgatgaatttcggctacgcagacttcctgtcccggctgatgtcatcacag

cgcagaccagatgaggattacgtcatagctgccgtctatgtcgaatccgaagcaaaggcgattctcgaag

actccatcaacaatctgccagtcacacatcagatgattgtggctgagacacgtaaggatgctgttctgca

gcaagtgattagttacatcaatgaaggatggccagcaagcgtgaagctaatcaccgatcctgatgtgaag

aagttcttcgtcagacgtgagggacttcaagtcgtcgataactgcgtcatgttcggcgatcgaatcgtcg

ttccatcaaaattccggaagcgaatcgtccgccagctacatcgtgggcacccaggaatggagcggatgaa

gtctctggctcgcagctacatttactggccgaatgttgacgacgatgtggcgcaattcgttcgtcagtgc

gatgcatgtgctgaagcagcgaaggctccgacgaaagcaaccctggaatcatggcctcttccggaccggc

cgtggcaacgagtacacgtcgatttcgctggcccaatcgacggacatcactatttcgtgattgtagatgc

ctactctaagtggcccgaaatttttcgcactagatccatcaccacgacaacaactttggacctgcttcgt

gaaacattttcccgttacggcaatccagacacgctagtctcagataacggaacgcagtttacgagcggac

agtttcaacagttttgcagtgagaatggcatcaaccatattcgtactgctccataccacccgcagtcgaa

tggccaagctgaacgtttcgtggattccctcaaacgcggccttaagaagttaggtaagggggaatcacca

acattacagcatctacagacgtttctttcagtgtaccgatcaacacccaaccggaatacacctaagggaa

cgtctccagccgaagcgtttttaaaaagaacgatgcgcactacgttggatctgttgaggaaaccatatcc

tccgactgcagccgttaaccacaaacaaaatgaacaattcaacaaacggcatggagcggtcaagcgatca

tttgtagagaatgacttggtgtatgttgaacaacacgtacacaacaaaaagtcgtgggttcctggtcgag

tcattgaaccaaaaggatccgttaactatgtcgtgtcgcttgacttgcatggaagacagaagctggttag

atcgcatgtcaaccaaatgcgttctcgctacggttccgaaacccctgggcaagaacaacaacaacttcca

tgggaagttttattagaagaagttggtactactgctgcagtgaaaccagctggcaatcatgttgctatta

atttgaactctacggtgccagctgatgttcctgtttttgaaaatccgccaaccgatttcaacccgccaac

aaccaatgaacaaccatctgcatcagcatcggaatccatcgcatccgaatccgtcatagccgaatctatc

gcatctgaatccgtcgcatccgaatccatcggatgcttgctacgtcgttccgtacgaacaccgagaatcc

ctcgatggctctcatcatacgacctttattaaaaagggggaga

>gypsy21-ltr_ag gypsy anopheles gambiae str. pest

tgtagtaggctatgactactatgactaccaaacagtgggattttgcctcagcagaatttcacttcctagg

catagtagcctatgccatgtttagtaataaagcagttcatagttaaccaccaaactagcaagttggtttt

tatttgctctctgtgtgaactgtaaca

>gypsy22-i_ag gypsy anopheles gambiae str. pest

aattggcgacgaggaagtacgaaaaccacgttattatacctaagcaaactaagcttgtgttgttttcatg

gatccggcgtcaaagaagaccgaaattcctgctgcttctgtcaccacacgggctgctgctaaatctgcca

gtaccggaacacctacggccgattcttcttcaaaccttcctactggcgtccttgctacggacaattctgc

tactgctaagcctatttcttcgaacacttctactagaggccctgttacggcccattctgctgcggtaaaa

ccaaaggcatccagttctactacatccagcacatcggtgcccggctcaagtgtgggtgaaggtaccgctc

tgtctgtttcggtccgggggataactactgctgttacgaatatgtctacaatgttctcatttgagccgtt

tgatccaacaaactgcaaaatccagagatggttggaaaggttgcaaattgccttcaaaatccatcgagtt

tccgaagaagacaaacgtgattaccttctccattatatgggtggtgctacttatgacgtgttgtgcaaca

agttaaagaatgcagaaccgcaaactaaaacgttccaggaaattgtttccattcttcaagagcatttcaa

cccaaatcctttagaaattttggaaaatttcaagtttgcaaatcggaagcaagctgaaaacgaaacactg

tctacgtatctaatggaattggagaagctagcacaaacatgcaattttggggattacctcgacaaagcct

tgagaaaccaatttgtattcggactccaaaaccgtgcgatccaatcgcggttgctcgaggtgcgcgactt

aacattggctaaagcgaaggacatagcttttagtatggaaatgtcaaatcgaggcgcagacgaaatacac

ggcgccggtgcagcgtatccagttcagcacatcagcaccacgagcaagaagaagagcaagccaacaacgg

ttcaaaggaaaacagcttgctatcggtgtggaaacgaagagcattttgcggataagtgtcgacaccggaa

tgcaatttgcaactactgcaagaaaatgggacacttggataaagtgtgtcgtacaaaactacaacgagca

ggagtacacacactggagtacgagcctgatcttcctgcctgtgacgatgacgttgtggatgtgctcaacc

tgagagcagtgcaaaatctggcgggtaagtttttgctggaaatggaaatctctgatagaaaacttatttt

cgaggtggacacgggttctccagtatcactaataagtaacagggatagattaaattgttttcctaacaca

ttgatgaagaaaagcaatgtaaaattgaaaagttactgcaacggtgttatcaacgttcttggagagatcg

aagtgagagcaaaaattaaaaatttagaaattcctttgccattgcttgtcacaaaatccgacagaaatcc

tttgcttggacgtaactggatgagaactataaagttagatttgaataaatttatgcataccagtcaagaa

gtttcatattgtgaaaaggaaaatttcactccatgctcaatactagaagctctactaaaaaaatattcgg

aagtatttgaggcaggaataggcaaaattgaagggttacaagcttcattaactcttcgcaaagaaacaaa

acctatttttatcaaagcgcggcctgtcgcatttgctgtccgagatgctgtaaccaatgaaatcaataag

ttagttgaggaaaacgttctagagaaagtggatcattctgaatgggctacacctattgtgccagtaaaaa

aatcagggggtaatgtaagattatgtggtgactataaaatcacggttaatccaaacctattagtagacga

gcacccactaccgacagttgaggaattgttcacaaatattgcaggcggggaaaaattttctaaattagac

ctttcccaagcctatctgcaattggaggtaagccccgattgtcgggatattttaacattagcaacccaca

aaggattgtaccgccctaccagactaatgtacggggtggcatcggcaccagcaatatttcagaggttaat

cgaacagattctacaggacattccaggagtaactgcttttattgatgatattagaattacagggccaaac

gatgaaatccatttaaaaagattagaagaagtactaaaaagattacgaaagtacaatctaaaagtaaaca

aagctaaatgcgagtttttcgcagatcaaatagaatattgtggatacctagttgataaacatggcataca

caaactacatacaaaaattaaggccatacaggacatgccggctccgaaatccgtagatgaattaagatct

tttctaggtttggtaaattattacggacggttcttcccaaatttaagtactgtaaactatcctttaaaca

atcttctaaaagatggaataccatacgtttgggacgagcattgccaaaaggcattcgctcaggtaaaaag

ggaaatgcaaacggagagagtactggtacactatgatcctaaccttcctctcatattagctacagatgcc

tcaccctacggggtaggtgccgtccttagccataaatttgccgatggaaccgaaaggccattacaatacg

catcgcaaactttaaacaaaactcaacagcgatattctcagatcgacaaagaagcctacgcaatcatttt

cggagtgcataaatttcaccaatatttatatggacggaaatttactctggtaacggacaacaaaccctta

tcacagatattttcagaatccaaaggattaccaacgatgtcggcaatgcgaatgcagcactatgcagctt

ttttacaagggtttgattataacattcgtcaccggaaatctgtaaaccactgtaacgccgatgcattgtc

aaggttgcctttaccgtcagaggattctgatagaagaatagaggattccgatttagttgagatcaacgta

atcgaaacattgcctttaacagttcctgaattggcaaaagccacagctgtagacccaaatgttgaagaac

tactgcgtgccctcagaacgggtaaaataattttaccgaaacactgttttggtattgatcaaaatgaatt

cgatttacaaagtgattgtattatgcgggggagtagagtttacattccgcctttattaagagaaaaggta

ttacaggagcttcactcttctcattttgggatctctagaatcaaatctttggcaagaagttattgttggt

ggcccaacatagacaaagaaattgaaaatattgtaaataactgccaaccctgtcaagagacaagagcaaa

cccaccaaaagtaccgatacattgctgggagaaagcagaagggccttttcagagggtgcatgtggattat

gctggtcctttcatgggaagctacttttttattttagtggatgctttctctaaatggcctgaggtccgtg

tcgtcaacaacatgactaccgaaaccacaattaatgcctgcagggaaatattcagtacattcggaattcc

tatggttttggttagcgacaatggcactcaattttcgtcaacggaatttaccaggtttttaaagctaaac

ggggtgattcataaatttagtgcaccttaccatccagcgacaaatggacaggccgagcgttttattcaaa

cactaaaatcaaagctgaaggcagtaaagtgtaatcgtacagaaattccagaagtgttgagcaatattct

attgtcttacagaaaaataattcatcctagcactggtttttcgccctctttgttagtatttggaaggcaa

atacgcagccgtattgacataatgattccaacttcaaactcgaacaatagagaaatcataaaaactaaag

attttgaaataggacaaagagtagctgcaagggagtacattaaaaataacaaatgggaattcggaaaagt

aacagcaagattaggtaaactgcattatgaaatagagttagatgatggacgaacttggagacgtcacatc

gatcaaatgcgcgctgtgggtattagtatgcaaaaacccataaataggaacacttcttggcaaggcagag

agactacttcgcataatttggaatcagaaactaataccatcccgaaaaatagcagtacaccagaactaat

gacagattgtggatctaggtcaatagtacaagcgcaaccatccaaccctccaccaatgagttttccatca

gaggctccatgttcagcggaaataccaccggaagcaaaactgcggcgatcagcaaggagtatcaaaacac

cacaaaggctactactgtaaataactatttcggagggaagagc

>gypsy22-ltr_ag gypsy anopheles gambiae str. pest

tgtcataaccatcgaaatgtcaacatcaccaaatggaccaacctggtagcggttggctgacagcggctgt

caacagggggaacgggaaaaaagtgcgccactggagcagcgtcgggagaagcgtacaaacggaaaagata

cagaagtacaaatatacgagtgaaatacaaca

>gypsy23-i_ag gypsy anopheles gambiae str. pest

atttggcgacgagaaaagggaattatacgttttcagttacttaaacagttccctacgttggtgagaagga

gcatttaccgttattacttctatcggtgctacgaatacggtgaattttgatgctgttggcggtactacgt

ctggcgttgctactgctgccggagcgtcagctggattgattacgggacgagctgctaccatttttttgct

actaccggagatactactgctgctcctactactcttactgttgctgtgactgctactgcttgtatatcga

tgcaaccacaaaccgtggcgagcgctggatcttccacacctactgcttcttcttcttccggtaccaacgc

aaaaatggctaatatgtgtgttttcgagcctttcaaccccgtgtcatcatcgttcgatagatggatggaa

cggctaaaaatttggtttagaatcaaccaaattggagatggcgataaaaaggactaccttctacattaca

tgggcggccctacgtacgacgtattatgcaacaagctgcaaaacgccgatccctacacgaagtcattcga

cgaaatcgttgcactcctgagaaatcacttcaaccctgcaccgctagaaatcctagaaaacttcaagttt

actagccgtaagcaattggagaacgaatcattgagcgaatacttaatggagttagagaagctggctaaaa

gctgcaattttgattcgtacttagacaaagcgctgagaaatcaatttgtctttggcattcggaatcgtgg

aatacagtcacgattgctggaagtacgcgatcttacactatccaaagccaaggacattgcgtttggaatg

gaaatgtcattgcgtgggaccgaggaaatgcatggaaccagcccgagatgtgaagtgcaacaagttacgg

ccaatacaaagaaaatatcatcgacaaacaacggtcaacagcgaaagtgctacagatgcggcgatacaaa

tcacatggctaatagatgccagcacaagcaaacggtttgcagcgcttgtgggaaaagaggacatctacaa

aaagtgtgtttgtctcgccacaacaacaggcgtcaggagaacacacattatttggaggaaaacgacccaa

aggatgttcttcatgtgagcacggtccagaatcatgctggtaagtttttgttgaatctgagggtcaatca

aggtgtactaacattcgaggtcgacactggatcgccggtatcattgataaacataaaggacaaacaaaaa

catctcaaagacatagaaattttgcaaacggacttaagactagtaagctattcggacaatgacataggtg

tgttgggaaaattattggtcacagtagtggtagagggaagaaaactcgtattgccactgtatgtaacgaa

gtccaacaaacaccccttgctgggacgtgattggttacgtgcattaaatttagattttaatcgcattttc

aaatctggcacacacactgtttcatactgcgatagggatgatgaatgcaagtacagtgcattaaatgcct

tacttcagaaatatccatcagtattcagtaaggaaattggaaaagtaaaaggaattcaagcttcactaac

agtacgggaacatacaaaacctgtgtatattaaagcaagacaggtgccgtttgcgcttcgtgatgcagtt

gataaagaaattaatcaatttgtaaatgatggtgtatgggagcgcgtagaccattctgagtgggcgactc

ctgtcgttgtggtaaaaaaagctggtggtaaggtacggttatgcggagactataaaatcaccttaaaccc

aaatttaatggtggatgagcaccctctccctacaattgaggaactttttgtgactgtcgctgggggtaag

acattctctaaaatagatctatcccaagcatacttacaacttgaagtacgaccagaggatagaaaatttc

tcacccttagtacacatagaggattgttccagccatcaaggctcatgtatggtgttgcgtctgccccagc

tattttccagcgtctgatggaggaggtgttacaaggaatagaaggagtgacagtttttatagatgatatc

cgtgttactgggccagatagtgaaacccacttacagaggttggaatcggtattgcaaaggttagacaagt

acaatttgcgagtgaatagggataaatgcgattttttcgccaagcaaatcgagtattgcggctatatggt

tgataaagatggcattcacaaagttcgtaataaaatagacgctattcagaatatgcccattcctaagaac

agggatcaagtacgatcatacgtaggtttgataaactattatggaagattctttccaaatcttagcacga

ctttatacccacttaataacttattaaaagaagatgttccattccaatggacaaaggaatgcgaaagttc

atttaaagccgttaaaaaagaaatgcaatctgatcgatttctcgttcattatgatccttcactaccagtg

actttagcaacggacgcttcgccttacggagtgggagcggtccttagtcatcaatacccagatggtacgg

aacggccaattcaatacgcgtcccaaactctcaatagaacacaacaaaagtattcgcaaatcgataaaga

agcgtactcgatcatttttggtattcgaaaatttcatcaatacctttatggtcgtagatttattttaata

accgataacaaaccgatcagtcaaatattctcggaaactaagggacttcctactatgtcaactatacgta

tgcagcattatgcagcatttcttcaaggttatgattacatcgtgcgacatcgtcgttcatcagaacactg

caatgccgacgccatgtctcggttaccgacatgcacgactgatcccatgaatgaaatagaagaacctgat

tttattgaagtcaatgccatcgaaacattacccctcactgttgatgagttaagttcagctacaattgcag

acgatactgtccgtgaattgcttcgagccttaagaatgggaaagagcattgatgctaaattccgatttgg

catagatcagaatgaatttagtttacagaaggattgcttactccgcggtactcgtgtttatgtacctcct

gctctacgtaaaaatgttttaaaagaacttcactcgacacatttcgggatttgcagaattaagtctctag

ccaggagctattgttggtgggagggcattgataaagacatcgaaaatgtggttaaggattgtcaatcctg

ccaagtgtcgaaagctaatcctcctaaaacatcattccactgttggaatactccaaatgaaccatttcaa

agagttcacgcagattacgcaggaccgtttatgggatattactatctgattttaatcgatgcctattcta

agtggccttcggtttatgtagtaaacaacacaactactgatacaacaatacgagtgtgcagggagttttt

cagtacttttggaatcccatctgtgtttgttagtgacaatgggccccaattcacttcagctgattttaca

aaatttttaaaactaaatggaatagtacataagcttattgctccataccatccggccactaatggacaag

ctgaacggttcgtacaaacaatgaagtccaaattaaaatctttaaattgcgatcgttctcaagtccatag

tgaaatatgcaatatccttttaaactatcgcaaaatgattcatcctgccactggtttctctccttcaatg

atggtgtttggtcgacagattcgttcaagattggatcttatgattccatcggatgaccctgagaggagtg

aaattcagaataaaattcgagagttgacagttggctcaaaagttgctgctcgagaatacctacataacaa

caagtggagcttcggcactataaaagaacggttgggtaaattacattatttagtacaacttggagacggt

cgaatttggaaacggcacattgatcaactgcgtagtgtgggcgataatcttcccatatctaccgaggatt

caattttattgcgcgcagaagagcccaatagccacactgaagacttgactgtgacctctgacttaccctc

gacccagaccccgttaacggacacacagtcttcaacaaccaacgtttctgactctgcacatccgactata

caaactgtccctgtgcgttcaaaggtgatccagtcgtcgacatctactgagtgtgccacctttgagggga

cttccggttcgtcaggcttgacagcggattctggacttcgtcgatcaacaagaaccatcaaacctcctca

acgattggatctgtagcagagcggaagtaggtgctttatttccgggggaagagc

>gypsy23-ltr_ag gypsy anopheles gambiae str. pest

tgttatatacaacattgacagctatggcatgttggcagctctgcgactgctcgaacgagcagcactgctc

gaaacaggaagggagcgaatgaaatgtcatgcatacaaggaactgaataaagaaaacgcgtgtaacattt

gtactgcatcaaaaatacaata

>gypsy24-i_ag gypsy anopheles gambiae str. pest

ttttggtgtcagaagtgggatagtccaggatacgtgtcggatacatcggagccagcggaaaagcgatttg

tcgcccgctaaacaaacgatacatcacacacacatcccaagcagaaaagtgaggttagcatcaccacgtg

cgaacaacattacgacaaggaggtttctttcgctgtacgctgaatacaacagaaaacgatggcaaatgat

aaccgtgagttgctggaggccctttccggaatgcttgtgcaggcacttaaggcatccatcggaccagccg

ttgaacaagtaagtgcagaactacgcaacacaggtgaaaatatcccagcgccgcttccgaaagctccgtc

atttgctatgcccgaataccgtgccaacgagggaacatcggtcgctgattattttaaccgctttgagtgg

gcgcttcagctaagtaaaatcccggaaatacagtacgcggattatgctcgtgtgcatatgggagccgagc

taaacacgtcgctaaaatttttagtcgcaccaaaaaaaccacaagaagtgccatattcggaaatgcggaa

aattttagtagctcattgggaccagaaaaagaataaattcgtagaaagtattaaatttcgaaccatcgtg

caacaacgagacgaatcgattgcacagtacgttctccggttaaagcaaggttcagcaaattgcgaatacg

acaattttttagaccgaatgctcattgagcaaatgttacatggattgacagagcgcgacatctgtgacga

gatagttgcaaagaatccatccacatttcaagacgctctcgatgtagccctcgcgttagaagcaactcgc

aatattgctcgagacattaacacgtcgcaaccagcttctgaagctactaacaagctaggctacgaaaagc

caaatgtaaaaaaaccatacacgcgtcgaaacacgacaaacaagcagcatgcaaactcgccagagaacac

cgcattcaacaatacacacggtaaccagccagtagcttgtaatggctgtggaggtccacacctcagaagc

gagtgtcgtttccgtagcgccaaatgtaacaattgccataagaaaggtcatattgctaaggtctgcaaat

cgggtaagtccaaccatcacatttcacaacaagatatctcttcgccctccggtagtattgatcaagtgca

acggcttaaccgtattcataacataccgtcgagtgagaaaaaaatgatcgatgttaagatcgatggtaaa

tcgctgaagatggagcttgataccggtgcaccttgcgcaatcgtatcagaagcaaccctcaaatcaatta

aaccacatttcaccttgcagacaagcgacagacaattttctagttatactgggcatcgcatcagctgtat

tggtaggatgaatgtcaatgtaactattggagccacaacgcgaaaggagcaactctatgtagtgtccgga

gcacacgattcactcctgggacgcgaatggatctctcactttgcagatcagatcgatttaaatcgcatgt

tctcctcgcgtacatccatccatacagtgtcaaatagcacattatctccaaattgcgaaacgcagctaac

aagattattagacagctatgctgatgttttcagtgagtctccgggtaaactgacaggtcccccggcaaaa

gtacacttgaaagaaaatgcaacaccagtgtttgctagagcacgcgacgttcccctcgcgctgcgagaaa

ggtatgccaaagaaatcgacagtaaaataaattccggtttttacgaaaaggtcgaatattcggagtgggc

atctcctacacacgtggtcgttaagaaaaacggtaagctgaggataacaggtaattacaaacctactgta

aaccctttaatgataatagacgaacatcctattcctcgaattgagagtattttcaaccgaatgaaaggtg

ctactctattttgccatttggatgttaccgacgcatatacgcatcttcccatagacgaacagtttcgtca

tgtcttaacccttaacaccacaactcatgggctcatacgaccaaccagagcagtatacggtgccgccaac

atacccgcaatctggcaacgtcgaatggaagaagttctcttaggccttacaaatgtcgttagcttctatg

acgacattatcgttttcgcgaaagattttgaagagcttttacaagccttaacaagtatcctaagcagaat

caaggaaagtggtctgaaacttaaccgatctaaatgtgtctttgccacaccatcactcgagtgcttaggt

caccgaattgatcgcgaaggtcttcacaagtcgacgaaacacattgaagcgatccgagacgcaccaagac

cgtcttctcccgaacaattacagctatttttgggtaaagccacatactattcagcgttcataccagattt

gtcaacaagagcaaaggtattgcgtgagatattatcagcagatcgttttgagtggacggctgaagccgaa

gaagcctaccgcgatatcaaaaacattttaatttcaccacaagtccttactcagtatgacccaacactac

cattgatattagctactgacgcaagcaagacgggtctcggagcagtgctctcccatcgactcagtaacgg

ggtagaaagacccatagcttatgcaagctgtacaatgtcggcgacggaacaacgctatccggttatcgac

aaagaagctctcgctatcgtttgggcagtcaagaagtttttcaactatttatatgcacggaagttcacgc

tcgtcacggaccacaaaccgttgacgcaaatcctgcatccagagaagtcactgcctacactttgtataag

tcgcatggcaaactacgctgactacttagcgcactttaatttcgatgtagtgtaccgatcgactaacgaa

aataagaatgccgattattgttcacgcattccaagtccctcgacacaatccagtgtcaacagcctttctc

ttcgtagaggaggaaatgaggatcaagacgattttgaagattttgtgcttaaccaaatccagcagctgcc

cattaaagccgatcaaatcgcacgcgaaacgcgaaaagatgagcacttgggtaaaattttgaaagacctc

gaaatgggacgaaacctatcacaaatcggctataaagcaccagaagccaaatacaccatggttgccaatt

gtttgctgtttgaacaccgtgtcgtgattcccgacatctttcgtcctgcaattctgcaagatttgcacgc

agcacatattggtgtggtgagaatgaagtctttggcccgttcatatgtctactggccgggcatagacaaa

gacatcgagcagctagccaaatcatgccacgaatgcgctcaaacggtctcagcacctcctaagttcaatc

aacaccattgggagtatccatctaacccttgggagcgtgtgcatgttgactatgcgggacccgttgctgg

cgcgatgctactgatcatcgtggatgcgtacagcaagtgggttgaggtgaaagtgactcactcaaccact

accgaggcaaccataaaaatcctcgacgagctatttgcatcctatggagcccccctaactgttgtaacag

acaacggaacacaattcactgcagcagagttcaccacatttcttcaacgaagcggtgtcaagttccacaa

acgctccgctccatatcatccggcaaccaatgggcaagcagagagatacgttcagacagttaagcgagct

ttgaaggctatgcattcgtccagcactacacttcaagctaacctgaacgagttcctgctccagtaccgca

aagtcccgcacagtgaaaccggtgaagcaccagcaaagcttttcctagggcgaaacatccgttcacgtct

cgacctggttcgaccacaatccgtccagacaagaacagcagagaagcaacgagtcgcttttgaaccatcg

taccgaacattcttgcccggacaactcgtctactgtctctcgggaagtacgagaatggataagtggatcc

gaggtacagtggtatcccgactaggcgatctacactactccatcaactgcaatggtaaccagatgaaacg

ccacgtggatcagatgcgaccaaccctagacgacaacaggacagaacagccgcgaagtgtacctgtacca

actcaaactccggaggtacaccatcaccgcaggcactactacgggtcaaccgactctccacaaacatcga

gtgtccccgtttcatctcggacagtctccgtgtcatcagactcatctacttcgtccgattcatcgtatga

cacaccgacaggaagcccgatccgagccagtgacgccccgcccttcgtccgccgttctacaagactgcga

aacccgccgttgcgatactcgccgtagttcatttctaagaagggaggag

>gypsy24-ltr_ag gypsy anopheles gambiae str. pest

tgttatatatgaaccctggtatttgacagttgtgtcataacgcctgacaggttgaccctacctttcgttt

gttgttaaatgtcattccttctttacaccttattgaattcgtttggttactttttgctacaactccttaa

acaaacctcatgtgaacttttctgcctaataaagagaattaaatacataaca

>gypsy25-i_ag gypsy anopheles gambiae str. pest

gtggcgacgagtttagttacattgttttatttattgaactgtttttatcgaaccgaacgcagagttaatt

tattttgtttcgttttaacgtattatttattcaaagtgtgtcggccgtatacgcgtcaagtcgttatttt

attttttttattaatcgtttgtgaatcgcgccaaaagaaatcggtacaatttcgcgtaagcgtagaagga

tgctaaacccggacgaaatgatggaggaggaagagcatcgccgcttatcccaaaattggggtggaaatgc

agtttttggtaatgggcagcagcagccatcgcagagcaacgtagcgttaccagctcaaccaccattgcaa

aatgtagtcggggcgccatcgcagcaaggtgcatcgacctcggggcaagacgccgggatgctatcgcaaa

tgctgcaattattgcaacagcaaatgaaccagcaacagcagcttatggcgcaaatgttgaaataccaaca

gcaatccgtcccgcagccttcgcaacctagtttgataccgactaatcctgagcttataattgatgcgttg

gcgagcaacataagcgaatttagatacgaggctgaatcaggagcaacgtttaaggcctggtacgagcgtt

acgaggacttatttttgagggacgcttcccgactcgacgatggggcgaaagtacggctccttgggcgtaa

gttgggcacggcagaacacgctcgtttcaccagttttatactacctcgcgcgcctcgtgaactaacattt

gacgaaacggttgcaaagctaacggccctttttggtagaacggaatccttactcagcaaacgttacaagt

gcatgcagataaccaaagcaccccgggaagatctgctcacgttttcttgccgcgtaaaccgtgcctgtgt

cgactttgagtttgccggaatgaacgaggagcaattcaagtgccttatccttgtttgcggactcaaagaa

gaagtcgatagtgacatgcgcaaccggttattagcccgtatcgaggagaaacatgatgtgacgttggagc

agttatcagcagaatgccagcgtatcaccaatataaaagtggatagtgcattaattgccaacgaatcagg

agaacgggtccttgcagtgaacagcggtggctatagacaaaaacaatttcaatttaatcgtcagcagtac

cagtcctacggtcaaccaacacaggcgcatgcacgggcaaatgataacaccatgtcagtacaaaagccgt

tgaatgcgtgttggttgtgcagtggcccgcattggaagcgtgagtgtccatataggtcccacgaatgtgc

ggattgtggaaggcttgggcatcgcgaagggcactgtgaagtcgtcaatcgattccagcggcgtggatac

aataaaaaaggtactaatgtggcaacgcgcgtcgtgaacataaatgtgtgcaatgtagaagcaaggcgaa

agtatgtacatatcttgatcaacggaagaccaactaagttgcagctagacacggcatcagatattacggt

gatcagcgagggattgtggaaggatatcggacagccatctctgataagggctacggtaaaagctaaggcg

gcctcgcaggagtatcttgaactaatgggtgagtttgaagcgttattaaccattgcttctaggacccaaa

aggcagtagttcgagtcgcatatgctaacctgttgttactaggagcagacgtcgtggagtccttttcact

cggatctatcccgatggaccattattgtagtagcatcgatgcggaaagtgcaactcagatgacatgggag

gaacgttttccaacagttttccgagggatgggtctttgcaccaaatcaagcataaagttgaaagtgaagg

aaggcagtcgacccatatttcgtcccaagcggccggtggcatacgcaatgctgaagactgttgatgagga

gctagatcgattagaaaccttgaacgtgatcacgccagttgattattctgagtgggctgcccccatagtg

gtagtgcgtaaagcgaacgggaaaattaggatctgcggtgactattctactggactgaacgatcttctac

aatcacatgagtatccactccctttgcctgaggatatcttcgctaaattgtctaaatgccgcatattcag

caaaattgacctttctgatgcttttctacaggtccaaatcgataaagagtatcgcccgctgttgacgatc

aacacccaccggggattataccactacaaccgtttgtctccgggaataaaaattgccccagcagcatttc

aacagttgatcgacacgatgctggcaggactgtcaggagtttgtggttatatggacgaccttataatcgg

tggcttgacagatgaagaccacgataagactttgggtcaagtactgaagcgtatagaagaattcggattt

acactacgggctgataaatgcgtttttagaatgtgtcagataaaatacttagggcatgtaattgatggta

gaggaatacgccctgatccggaaaagataagcgccatacaaaacttaccgccacccactgacatcgcagg

agttcgttcttttcttggagctataaattactatgggaagttcataccgatgatgcgggatctaagattt

ccactcgacagccttttaaaagatgagaagcaattcaaatggacaaaggagtgtgaagcggcatttatga

aatttaaggaagtactatcatcagaacttttactaacacattacgatccatccgcagaaataatagtggc

agcagacgcctcatccgttggaatcggagccactcttagccacaagttctccgacggtagcatcaaagtt

gtgcaacacgcttcaagggcgctgacaaaggcggaatccaactatagccaaatagaccgcgaaggtctgg

cccttgtttatgcggttacaaaatttcacaagatgttgtacggccgtcattttcgactccaaactgatca

tcgtcctttactccggatttttggatcgaagaagggcattccgatatacacggctagcagactacagcga

tttgccctcaccatgcagttatatgatttcacaatagaatatatacaatccggaatgtttggaaatgcag

atatcctttcgcgactcataaggaaccacgcaaagcccgaagccgaatatgtgattgccagcctaaacct

agaggaggatttaaggtcagtagctatcaacgcgatttctaattcctctcctctttgttttagagatgtg

gagaaaagtacgcaagcggacccattgctgcggaaagtctatctatatattcaggaaggctggccacggg

atgctacttttggttcagagttggcacgttttcacgtcaggagagaagcgctatcgaccgtcgaagggtg

catcctctttggcgaaagattagtgataccggaagatctgcgtgcgcattgtctagaacagctccatcga

ggccacccaggcgttgaacgtatgaaatctctcgcacgaagctacgtgtactggccaagattagacgacg

aaatagtgcagtacgtggctgcttgtgaggcatgtgctgcagcagcaaagacaccgcctcaagcaaaacc

gacaccgtggccgaaaccttctggtccatggcaaaggttacacgtggactatgcggggccaattttaggc

gactactttcttgtggttgtggatgccttctcaaagtggccagagattgtgaaaacctccacaacaactt

cacgggccacggtagcgatattacgtggattattcgcacgctttggcatgcccctgagtattgtcagtga

caacgggccacaatttacgagtcaggaattcaaaaacttttgcgagtgccatgggatccgacaagtaaca

acggcgccatttcacccgcaatccaacggacaggcggagcgtttcgtcgacacattgaagcgggctctaa

agaaaatacaaacgggaggcacatctatggatgaagcgttggacacattcttgcaggcgtatcgcaccac

gccaaatccagcgcttgagtggaatacaacaccggcggaaatcatcattaagtaccccgtaaggactcac

ttggagctattacgtcggcctcctgttgtcgaagaggaaacagaaatcaccgctgcagggctccagccag

gagatgttgttgcaaccaagaagtactaccaaaatacatggaaatggatctccgctgaggtgctacgaag

gctgggaacggtgatgtatgagttaaccagccgaaacggtcgaatgatgcggagacatatagatcagatc

aggaaacgatcagttaaagaacctcatcagttggtgcaggatagtgagacgcccacacaactgcccatcg

acattctgttgggtgaatggagtttaacggtaccgaacgagcctttgccgctaagcagtacaacggagga

attgcgcacggtcccttcaccccagccattctccccggcacaggttgcaccttcggaaagcaatcatcgt

aagatacgctcacctcgccgttcgtctaggaatagaaagcttccgcgaaggttcgatgcgttcatacttt

aaagggggaga

>gypsy25-ltr_ag gypsy anopheles gambiae str. pest

tgttatggcccaacctgcctaacggctaagggcactcaccgaagacaggcaatggtcttcgggtgatgtg

tgagtgtagcaggtcgggccagagagagagccgagcggcaaagtgcattattatactgtaggctatcgca

cgggcaacaagaggagatagataaaggcatattccatatattcagaaagacgaaagtgtactgtattcga

tagaagggtggcgatatcacaaca

>gypsy26-i_ag gypsy anopheles gambiae str. pest

aagtggcgacgagggacggagtaaaaacccgcagtatggcgaacgacgaggaacctcgaccgactacaag

tgctgcaaatgctgcaaatgctacaaataacaacaatactgctgaggccccccgcaccatcactcatgca

aatttcgcattcgacgcctttgataaggcaaaatgcaaatggagtcgatgggtggaaagaatcgaaacag

cgtttgagatatacgctataaaggatgtaagggtcaagcgaaatcttcttcttcaccacatgggaggtga

agcatacgatgtgctgtgcgacaaaatagctccgaaacgaccacgggaatgcgagtacaaagaggtgatt

gctacgctggaagaatatttccatccagaaccattggaaattagcgaaaatttcaggttcaagtgccgac

gtcaaggtgacaaagatgcccattcagcagaagaaagtgtggacgaatatttagtggcattgcgaaaaat

tgccacaacatgcaatttcgggcagtatttgtcaaccgcattgcgaaaccaattagtatttggtttgaag

agaaacgatatccgtaatcgtttgcttgaaaagcggaaccttacgttagaggaagcgagagacatagctg

ttggcatggagctatcaaaaaaaagcagtgctgaaatagaaaatggtggtccatcacaagatgtacatgc

agttcagaaacgtaatgaaaaacataaccacaaagaagcaaaagcaaacaaaagtttgccaaagaacgta

acctgttatcgatgcggtgatgcaaaacatgtggcaaacaaatgcaagcatatcaatacgatttgcaact

tctgtcggaagaaagggcatttagcaaaagtttgcatgaaaaagcaaagccaaaatgaagtgtgcacggt

aatttcgttaaacaacatcgttccgaaatggtggatagatcttaccgtagataacattacaatgcgtttc

gaagtggatacaggatcaccgctaaccattattggaaaacaatgctacgagaaatatttcaaaaacaaac

ctcttcaaaaatgcaatgtcgaactcgtgagctatacaaacaatgcaatcgaagtgcttggcacaataaa

agtaaaagtggataatgaaatgcttccgctctatgtggtgaatctcatgaaacgaccattacttggtaga

gaatggttaaatgcggtgcccgattggaataatcggttgcaggtaaacgaagtaaatgaaatggcaataa

atacaaacagccttaatactattttgcagaagtacgccgacgttttcgatccaaatttaggcaaaatatc

gggagtacaagcacacctgaccctcaaagaaaacgcacagccaatcttcttgaaagcacgacgtgtgccg

ttcaatttgatcgatgctgtagatcaagagcttgacaagcttgttgcagaggacgtgctagaagaagtac

caaccagcaaatgggcaacgccgattgtaccggtgcggaaggcagaaggtaaagtacgaatttgcggaga

ttacaagcaaactgttaatcaaaagttgcaggtagatcaacacccattaccaactgtagaagagttgttt

gcgtccctagctggtggtaaacggttttcaaaaatcgatttagtacaagcttatctgcaaatggaggtag

cacccgaagatcgtgagatgctgactcttaatactcatcgaggactgtttcgtccaaagcgtttgatgta

tggtattgcatcagccccggcgatttggcaacggcaaatggaaggaatcctgcatggaatttcaggggta

agcgtttttctcgatgatattaaaatcactggtcctaatgatcaaattcatttgcaaaggctagaagaag

tcttaaaacgactaaatgatagaaacatacgccttaacaaagaaaaatgcctgttttttgctaaacaaat

agattattgtggttacaccatagatgaacatggtatacacaaaatgcgtgacaagatcgaagcaattgct

aatatgcgtaggccaagtaacaaagacgaagtacgttcgtttgtgggtctggtcaattactatggaaggt

ttatgcgagatctgagcacaattctctatcctttaaataacttgctaaagaatgacactccatttaaatg

gaacaaagaacaagagaaatcatttcaaaaagtgaaagagcatatccaatctaatgaatgcttagtgcac

tattcaccagaactaccacttttgttggcaacagatgcttctccatacggagttggtgcagtattgagcc

atgtatatcccgacggcacagaacgacctattcagttcgcgtcacaaactctgaataaggtacaacaaaa

gtatatgcatgtagataaagaggcatacgctatcatgtttgcggtgaagaaatttttccaatatttatac

ggacgtcaatttacactggttaccgataatcaagcgattgctaaaattctaggagagcacaaaggtattc

cagtaatgtcagctttacgaatgcaacactacgcaacttacttacaagcatttgattacaaaatacgatt

cagaaaagccgccgataatgctaatgcagataccctttcacgactgccattaagttgccaagataccact

acattctttgaagaaactgatggcattgaaattaaccagattgaaacacttccacttacagtaaaagaac

taagccaagcaacagccgaagatctaacagtaaaaacactcatacaaggtatcacacatgggaagcaagt

accaaatgaaaacagattcggaattgaacaacaagagtttacggtacaacaaggttgcttgcttcgggga

gtaagagtttacgttcctgctaaattgagagcgcgagtactagaggaactgcattcgacacattttggcg

taacaagaacaaaatctctagcaagaggctattgctggtggcccggtatcgacatcgctattgagacaat

gataaagaactgcgctgagtgccaatctacaagaccggaaccatcaaaagttcccttacactgttgggag

aaaccaacggctccgttcgaaagagttcatgtagactttgcgggacctttcatggatacctatttcttta

tcatggtcgacgcctacagtaaatggccggaaatacgaatttgcaaatcgactacagcagaaaaaacggt

acaaatgtgtcgcgagatatttagtagtcatggattaccatcggtattggtcagcgatcacggaagacag

ttcacttccgatgtgtttcagcgatttttgaagatgaacggaattgtccataagatgggagcaccatacc

atccagctactaacgggcaagcggaacgttatgtgcaaacgttcaagcaaaagctgaaggctttgaaatg

ttcaaagtctgagttgcatcttagtctatgcaacatactaatcacttaccgcaaaatgattcatccttcc

acaggtaaaacaccatcacaactgatttacggtcgtcaaataaggtcacgtattgatcttatgctacctt

cgaatgaagtacatcatggaggaaatttgcatacgaagcaattttcggatggcgaccgtgtaagagtgcg

agactacctatcgtcggacaaatggaaatttggacgagtgatggaaaagttaggaaaactgcggtattcg

gtacgtctcgacgatggcagagtatgggaacggcacactaatcaaatgatgggtgtgggggaggacctac

cagattcgaacaacaatctgacagatgcacaacctacaagtagtcaggatgcagtgaacctgagacgttc

gagcagattgcaaggaagatgatttcaatgcaatcatccggatccttgttttcagtcgtcaaggggagag

a

>gypsy26-ltr_ag gypsy anopheles gambiae str. pest

tgttatatacacctcgtacacaccgtgcgtatccaaccacgatgtgtaaaccgctgtgacagttgacgcg

cggcggcagatatggaagtgagggaagaagaaaatacaaagaggaatttggcggcacgctctctcgcgta

cgaacaaccaaacagagtggtcgtgtttcattcaatatatccgaaagatataaca

>gypsy27-i_ag gypsy anopheles gambiae str. pest

actggcgacgagaataccacgatgtcgaaaccacctgcaaaagaaagctcagaacaatggataattgaaa

tgttcaaccagcaaaagctcttgaacagtgtattgtcggtgatgaaaacaaaaaatgatgatggatcgga

gaaagcgatcgatgccattgcggggcaaataaaagaatttcaccacacggaggatacaaacttcgaggct

tggtatagcagatacgaaggtttgtttcttgacgatgcgaagcggttggatgacggagcaaaattgcgtc

ttcttcttcgtaagattggcgttacggagcacgaacgctatataagttccatcatgccgaaacaaccgaa

agacttcgatttcaaaaacaccgtggagaaactgaagaaactgtttggagaccgtgagtccatcgtgtgc

aaacggtttaaatgcttgcagctagtgaaagagcctcacgaagagtacggagcgtatgcgtgccgcgtaa

ataaaaaggttgttgaggcgaagttggcggggatagcagaggaggaaataaaatgtctgttgtttgtctg

tggtctaaaacgggatgcggatgctgatgtacgagttcggctgctctcaaagatggaggacaacagtgaa

atcacgttagatcagctaaccggtgaagctcaacgcctgcttaacctgcgccaagatagcaaccttatct

caaacactgcgcatgaggtaaacgcagtgagaaagaatgagacgaaacaacaaaacatcaaaggaggtgg

caaggaagcgaaagcagtaagttgctggttgtgcggagggcaacactttgcacgagaatgttcttttacg

tcacacaaatgcgaggactgcaacgagataggtcacaaggaaggattttgtgaaacggcgtaccgtaatc

gttctcgctatcgcaataacaaagcgaaggtaagggtggtgacagtgaacaaaatccaggctggccgaaa

gtatgtcactgctgcgctaaacggacgcgagctgaaactgcagttggataccggtgcagatatcaccatt

atatcgcagggaaaatggcgtcagataggaggtcccaaattgagtccagcatccgtttcagcgaggacag

ctagtggaacaccgcttaagctactaggtgagttttgttgcaaaatgacgattaacaaacaacagaaaga

agcgttagttcgtgttgtagaagaggaactgttacttttcggagcagacagtatggatgttttcgggctt

tggaaacagccacttgatgccttttgcaacatcgttgggtgtgttaaagatgccgcagcagctgtgattc

agcaatttccatcgttgttctcgaacgaacttggtcgttgtgataagatgaaaattagtctacagttgaa

ggatgacgtgaaacctgtatttcgcccgaaacgtccagtagcgtatgctatgcagtcgatggttgaagat

gaattgaccaggttagagcgtaatggcataataacacccactgattcttcggagtgggcagctccgattg

tggtagtacggaaggcgaacggaacagtccgaatatgtggcgactattcgacaggtctgaatgacgcgct

tcagccgcatcagtacccgttgcctataccacaagacatatttacagccattggaaaatcagctgttttt

agtcagattgacttagcagaagcttttctccaagtggaggtatgtgaaaggagccgtgagttgttgacga

taaacactcacaaggggctttaccgtttcaatagattgccaccgggagtaaagacagcacctggtgcatt

tcagcaaatcgttgactcaatgttgagcggattagaaggtgtcgcgggttatatggatgacatcattgta

ggcggtgctgatgaggaaagtcatttgaaaaacttacgcgcagttttgattcgtatcgaggagtttggtt

tcaagctacgggcagaaaaatgttcctttttgaagccccaaatccgataccttggacatttgctagaccg

gcagggtatacgaccagacccagcaaagattgaagcgattttgaagatgccagcgccaacgcagctcagc

gaagttcgttcgtatttgggagcgataaattactacggtaaatttgtgtttcaaatgagagatttgcgtt

accccttagaccttcttttgaaaaagggcggagaattccagtggacagctgaatgcgaaaaaagttttcg

ccgctttaaggagattttaagttcagaccttctcctgacccattatgatccttccaaggaaattattgtg

tcagcggatgcttcatcagtaggtcttggagctacgatcagtcatcggtttccagatggtagcataaagg

ttgttcagcatgctgctcgtgctctaacgaaggtggaaatgaactacagccagccggatcgcgagggtct

tgcaatcgtgtttgcagtaacaaagttccaccgcatgttgttcggacgccgcttcatcctccaaaccgac

caccagccgctccttagaatctttggctcacgtaaaggaattcccttgtatactgcaaatagactgcagc

gctgggcattagcactacttctttacgatttcgcaatcgaatatgttgcgacagacaatttcggaaatgc

cgacatactgtccaggttaatggaccgacacgagaaaccggaggaggactacgtcattgctagtatccag

ctagaggatgatattaatcatctagtaaccagtgctactcaagccctaccgttgaactttaaagatttgg

agcgtggtacacaagcagatcctttgttaaggaaggtgttccagttcgtccaaaatggttggccaaaggg

tgaagagaagactgaagacctaaagcaattcttcgcacgacgagaggccttatctacggttggaaaatgc

ctcttattcggagaacgggtagtagttcccgcgaatcttcggaatcgagtgttaaaactgctacacaagg

gtcatcctggagtatgccgcatgaaggcgcttgcaagagggtatgtgtattggccgaaattggacagcga

aatcgagaatgcagtgaagacgtgtcagtcgtgtgcaagtgcggcgaaatctccagagcattgcacacca

gtagattggccaaaaactacaggcccctggcagcgtattcacgttgactacgctggacctatggagggag

agtattattttctggttgtagattcctactcgaaatggccggaaataataccaacgcgtcaaaccacagc

acaagcgacagttcaaatgttacggggattgtgtgcacggtttggtataccagaaactgtcgtaagcgat

aatggaacacagtttacaagttcggagttccgtgacttttgtatggagaatggtattcatcacgtgcgga

ctgcgccataccacccgcagtcaaacggccaagccgaaaggtttgttgacacgtttaagagagcggtcaa

gaagattcgggagggtagaggtgagatgcacgaggctttggatgaatttcttttcacctacaggaacacg

ccgaatagaatcttggatcaaaaatcgccgggggagatgatgttaaatcgcaaggttcgaacgtctctgg

agatgttgcgtccatcatttcgcgatattcgaccagcggatccagtggtggccgacgaagctactaggag

gagtttctccataaacgaccgtgtctacgcaaagcgataccatcgaaacggctgggaatgggtatctgga

gtagtcgacaagcgcgtgggtaacgtaatgtacatggtcaaaatcgatggcaagaccttgcgctatcatg

tgaaccagcttcggatgcgtaatgcagagaccaacgaagggcgaactcagcaaccaatgccactgaatgt

gttattagatgcctgggatatgtctggaacaacggtgtcagcatcctcatctgaaacatcggcagcgact

acaacaaatcttgcggaacgtgagatgacacctgcggatggtacagaagcaagcctacgccgctcgacga

gagtgaggaagcagcctgcgtggttggaggggtatcatcgcagttaaagcgggggaa

>gypsy27-ltr_ag gypsy anopheles gambiae str. pest

tgttggagacgctaacccgtcggcataaacgcgttgcgttgtttgacgtttgctgcttgggtcggaaccg

aagccaggaaagtgcgcgctctttgtatgcgatctccacggtgtacggacgtgtgtgtgaaccagaataa

tttgtaaccctttagaactatctgaattaccgaaatacaagaacttttacttcgtaaatacaaca

>gypsy28-i_ag gypsy anopheles gambiae str. pest

gttttggcgacgaggatggttgatcaagcaagtaacgcgggcaacgtgccatctacgagttcttcaatta

taccgaacgcagctgccgctgctggtacagttccacctccgaattttgctatggaaccatttgacaaacg

caaatcaaaatggatgcggtgggtggatcgactggaaactgccttcgacatctacagagtgaccgaagag

cgggataagaagaactacttgctacattacatgggaaccgaaacttacgacgtcatttgcgacaaggttg

ccccggctgcacctcgtgattgtaccttccagcaaattgtggatacgctgaaagattatttcagcccgca

acccttagaaattgcagaaaactacaagtttaacagtcgccgccagggtgacaaggatgcagttacagcc

gatgagtcggtagatgaatatttggtagcgctacgcaggttggccgcatcttgtaacttcggtgattact

tagagaaggcgttgcgtaaccagctggttttcgggataaaaagaggagacattcgcgatcgacttctaga

aaagcgaacgttgacgttgcaggaggctcgggacattgcagtcagcatggaattgtcccggaaaggtaga

accgagatcgaaggttgctcagcgaaacaagaaatgcatgcggtgtaccagtctaaaggtaaaggttgga

agccaaataagggtgtggacaaacccagcacgagtacggagaaatctaattgctaccgttgcggagacaa

atcacatttggcaaatgcatgtaagtacaaaaacataatttgttcgttttgtaaaacaaagggacatttg

gcgaaagtgtgttttaaaaagaagaaaaatgaagcacattcgacaggcagcgcggcggctcaaacaaatt

acgtggaacagcaaggcaatgacgcaacaatagaaagcacaaacatacgagatgtgtgtacagttggttc

atctacacattgcaagaaactgtggttaaatatgaaggtgaacaaaaagacaattaaattcgaaatagat

acaggttcaccagttacgatcattagtgaagaagatcaaaaaaggctttttcctgacgcacagctacgtg

cgtgcacaaccaaccttgtaagctattgtaacactccgatcgacgtatgtggcatacttgacgtagatgt

acagttagcaagacatacattaaagctgccgctgtatgtagcgaaaactacgaaacatcccttggtaggc

cgtgaatggttgtcagaaataccactagactggaacaaggttgtggcggattcgaaatcggttaacaaga

ttgaatcggattctatgtgcagtgtcgcagattgcaacgcattgcttgagcgatatccaagagtttttga

agcttcaatcggtcgcatctccaatgtgcaggctaacttgcgactaaaggaaaatgcacgaccagttttc

atcaaggcacggaaactgccatttaacatgataaaagtggtagaaaatgaattggacaagttggttcagg

aaggtgtactggtaaaggtagattcaagtgattgggcaacaccaatcgtaccgataaagaaatcacagaa

tcgggtgagaatttgtggcgattataaacagaccgttaatccgaatctggttgtagacagacacccccta

ccgtcagtagatgaactttttgcatcacttgcaggaggaaagaaatttagtaagattgatctggtacaag

catatttacagctggaagtggcaccggcagatcgggaaatactgacattatctacacatcgtggtctgta

tcgacctaatagattgatgtatggcattgcatcagctccggcaatctggcaaagacaaatcgaatcaatt

ttacaaggtattgaaggagtcagtgtctttctagatgacatcaaaatcacaggggaaaccgatgaaatac

acctttttagactcgaagaagtgctacgtcgactcaatgaacgggaaatacgcgtcaacaaagaaaagtg

tgaattcttcgttgatcaaatagagtactgtggctacgtaattgatgcgaagggcatacataaaattcag

aaaaaagtcgacgccatacaagaaatgccgaaaccacgaaacaaggacgaggtacgctcgtttgtcgggt

tagtaaattattatggtagattcttgcagaatctgagtaccatactttatccattgaacaatctactcaa

aaccgacgtgccatttcaatggaataaacaatgtgaagattccttcaagaaggtaaaggaacaaatgcag

tcagataattgcttagtacactactctacggagctaccgttgctactggccactgatgcttctccctacg

gggtcggagcagtactgagtcacatttaccccgatggttccgagcgtcccatacagtttgcttcacaaac

attaaatcgagtacagcagaaatacatgcaagttgacaaggaggcctatgccatcgtctttggtgttagg

aaatttttccaatacctttacgggcgcacattcactttacttacggacaaccaagctatatcgaaaatat

ttggagagcacaaagggttgccggtgatgtctgcattacggatgcaacattacgctacgtacttgcagag

tttcgattacaagatccgatttaggaagtctaccgaccatgcaaacgccgatgccatgtcacgcattccg

ttacatattactgatccggataacgagattgaagaatcggattcgattgagctaagtcaaattgaaacgt

taccgttgacagctgttgaattagagcaagcggttgctaaagatcaacaggttcaaaaactgattcaagg

aatcaaacatggccgggtagtagaagcgaaagatcgttttggagttgatcagcaagaatttgccattcaa

aaaggatgtttgctacggggtatccgagtctatgtgccagaaatcttacgcagaaaagtgttggacgaat

tacattcagcacattttggaattactcgaactaaatcgcttgcaagaggttactgttggtggccgggaat

ggaccaggatatagaacgaatggtagcaaactgtactgaatgccaattgattcgagcggaaccagctaaa

atgaagttacattgttgggagacaccaagtgctccatttcaacgagtacacgttgatttcgccggtccat

ataaggacttatattttttcatcttcgtcgatgcttacagcaaatggccgatggtaaaggtttgcaaatc

aataacggcagaacaaaccgtaaacatgtgtcgcgagttgtttagcacattcggaattccatcagtactg

gtaagcgaccatggcgtacaatttacttctacgctgttccaacaatttcttaaaatgaatggcgtggtgc

ataagatgggtgcaccttatcaccctgctacgaatggccaggcggagcgatatgtgcaaacaattaagaa

caagctgaaagcattaaaatgctccacatcgaagatcaatctggaattgtgcaacattttacttacctac

cgaaaaacagtacacccagcgacgggaaaatcaccatccatgcttgttttcgggaggcaaattcgatctc

ggttagacctattgattcctacgcaagagtcatcaaggaaggcagagataccagtacgtagtttccagat

cggagcaagagtgcgagtacgagatttcctgtcccacgataaatggaaatttggaaaaatttctgcgcga

gttggaaagctgcgatacgaagtgaaactagatgacggtcgtgtctgggaacgtcacatcgatcacattg

cggaggtgggtgctgatctacgaggtacttcggcggtcaaccaggagagcgatttacgggaacaaggcga

tacaacgtactttgaagaggtacttccttccacttcgaatgcgaatacggcggttactaccagcgacagt

gagataaccgtggaaaatacaccagggtcttctgatcgagatgccgatagacctgtagaacggaataacg

atccgggcatggaacgggatctgacaccagcgcctgtccgaccgatcaacttggaagaggagtcggtcct

acgccgttctactcgtgctagaaaacctccccaaagattgaatttgtaaatggaacgatttttttttcaa

ttacgaaggggagaag

>gypsy28-ltr_ag gypsy anopheles gambiae str. pest

tgttgtgtacaacggggatgggaatgtgacacttcacggccaaggtatatgtttaccttacagtgtggga

accttgggaagaaaagggggttccggaagaagaactgtcagagggagaacaaaaagggaagcctacgagc

taggaagcaattacgaggcagaataaataatacagtgaactacgataaataactgttccttaattacaaa

gatacaaca

>gypsy29-i_ag gypsy anopheles gambiae str. pest

tctcagaagtgggatagtgagcccaaaatgtccgtagatgaccagagagtgactaaggatgagatgctgt

gcgcactccagtcggccgaaataaccgtgccatgtacggcaacactgatgcagatccgtgctctctataa

ggaagcgtaccctagctcgcaacaaaatggcgattccaacgacaccatgtgcaacgcagccattgccgaa

agtaaagaagagagcgtcacccagatcatgaaaactgatgtgatcaaagaaaacgacgaagctgcagcag

aaattgtgctcctaaagcaaaaatgtgagatacttgaactgcggaacaaattagcagcgcttgagagcca

aagtcgtgaatactccaatacagctagattgcttcatccggaagaggttaagcagattattccgatgttt

ggagagggtgccagtttcttgcagtggataaaaacggtaaaacacagtgctgaagtttatggttggaccg

accaaatgacattgatgtacgctagcagtcagctaactggcgctgcaaaggagtggtacagtggtttccg

gcattctgtaacatcgtttaaagaatttgccgaaggaatgggaaaagctttcccagacacgcataatgaa

gctgctattcacaagaagctgttgcatacattcaaaaggaatgatgagtcctacgccgcctacatctttc

gtgtacatgcccttggaacaactgggaacgtgagtaacgcagcgattataacatacatcatccgaggact

atcgcgcgatcccatgtatgacaacttagtggcgaaagaatatcgtgatgtgtatgatctgattgatcat

gtaaaccgatgcgagatgcattaccaaatgcgtgaaccaccgtcatcccccactcgtccacaaccttcaa

gccgttttacatcgatgctgaaaaccaacaccaaaccagccaatgggcgagaagaaatggtgcgatgcta

caattgttcgagtttcggccatttttccaaccagtgccgaaaaccacgtcagtctgcccaactatgtttc

ttgtgtggaagccctgatcacaagaaacaagactgcccgaacgttgcccagaggatgcctgccgctatat

ttgcaccgtgcgatcagcagagtttccaacatcagtgtccggcatcacgattgcctcaatccggtggcaa

tgattactcacatctgaacctgtcgatggctgctgcagcagaaaacaattacggagatgataatggaaat

caagtgactggttcaggagctaggatcgatccaattcaggaggtaagtgtcgcattgctgaatgataatg

aattaagcgcaacgcaatcacgtgtgttttgtttgtttgattctggcagccctaaaagtttcatcagtga

acaacttgtgccaaacatcaaacatactcctcaattttcgggattctgtggattaggcaaccagcagctt

acatctttgggccatgtgaacattagaataaaatttcgaaatatcaacgtttcccattcctttatgatac

tgccaaaacaacaaacagcatggcccttgattgcaggaagggacttattaaaaaagatgaacatccattt

acattacctttgttatcgttattctcaagacaatctgatgaacttactaaaagaaaataaatctactctt

gcaacacacgtgaaagcgcgccttaattcattggggatttttaaaactaattcgaaggatgaggcagaca

tttggacgcaatctattttccaatcagaccagaaaagatacgatagtgttgatgaacataatcagaccaa

cgatagtggtacggaggctgagagtattttgaggactttttcagagttatgtgctattgatattagtaag

gaagcgaacacattggatataggaaacgaattgagtgaagaccagatgtgtctgatagcttcatgtgtgg

aaaataactatcttcagcccttaaatgaaactattcttgaatcacaacattcaatgaaaataagtgttac

aaatgatactccaatattctgtaaacctaggcggttgtcttttgccgaacgcaatcaggttcgtgatata

gttaaaaatttattagaaaaacaaattattcgacccagcaattcaccttacgcatctgctattgtgttag

tacggaagaaaaatggtgaggtacgaatgtgtgttgactaccgtccgttaaataagataacaattcgtga

taactatccaatacctttgattgacacttgcctcgaacacctaagcggtaaacggtatttcactctctta

gatctcaaaagcggctttcatcaaattaagatgcacgaagattctattagttacactgcctttgtaacac

ctgacggacagtatgaatatctgaaaatgccatttgggctaaaaaacgctccatcaggatttcaaagatt

cattaactcagtactgcgagaattcatcgacgaagccaaactagtcgtctatcttgacgacataatcata

gcgtcaaagacgtttcaagatcatttagaaactctaggtgctgttttaaaaactcttaggaagaatggat

tggaactgcgtattgacaaatgcaaatttgggtgcagtaacttggattaccttggttactatgtaaactc

aaagggaattcaaccaagcaactaccacattaaggctatccaaaattacccagtgcctaaaacctcaaag

gaagttcagcgttgtcttggtttgttttcctattttagacgattcgtcccgtctttctcgaacattgcaa

aacctttgagcaatctacttaaagaaaaagtgtctttccaatttgatgaagcatgtatgaacgcatttaa

tgaattgaaaaccaaattaattaatgctcctgtgttggcaatttatgatccttctcgagaaacagaacta

cattgcgatgccagcacagttggatttggctcggttttacttcaaaagcaagatgatgggaaattccatc

cagtggcatatttttcgaaaactgcttcttctagtgagtccaatcttcacagctacgagttagaaacttt

gtcagtgatttacgccttgaagcgttttcatgcatatgtgcatggtatccctataaagatagtaaccgat

tgtaattccctagtggaaactttgaagaacaggaactgctccgccaaaattgcaaggtggtccctgttct

tagaaaactatgaatacactatgcagtatcgccctggaacagcaatgggtcacgctgatgcacttagtcg

ctccaaaatggcaggtgctgttgacgagttggatctcgacattcagttgcagatagctcagggccgagat

cctacattagttcatctaagaaccgaactagaaacaaaaccaattccagggtacacactactggatggtg

tgatatatcgtcaatcccctgaaagcaaattgcaattgatcgttccaaaagaactgataaaaaatgtgat

tagaagcactcatgaaagcataggccatttaggtgttgacaagtgctgttcacaaatcgccaaacactat

tggttttcaggtatgaaaaatcaggtacaaaatttcattcagaattgccttaaatgtatacttttctctg

cccccccaagaaaaaacaagcgcaatttgtacaacatccccaaaagttcagtacctttcgatacccttca

catcgaccattttggcccattaccatccataaaatctaaaaagaaatacattttagtggtgatcgattct

tttactaaattcacaaaactttaccccaccactacgaccaacacaagggaggtttgttctgtgttgggac

aatattttaactattatagccgtcccaaacgaatagtgagtgatcgtgcaacttgtttttcatctcagga

gtttaaaacgtttctcagtgatcgtaacataatacatgttcaaaatgccgtttgttcccctcaagccaac

ggtcaggttgagagagttaacagagtaatcaaacccatgttaagcaaaattactgattccgtggatcatg

cagattggtgttccaaattatctgaagtcgaatatgcgctgaacaacacaacacactcgtcaacgtattt

tgctccttcggttttgttattcggtgtcgaacaacgtggtaccattataaacgaattcaaagagtatcta

gataacaaaaacgaaccttctagaaatttagaaactattcgttctgaagcttctgaaaatataaaaagat

cacaggaaatcaacctgattcagtttggcaaaagacacaacccagcggtagaatttgcggaaggggatct

tgttgttattcgaaacgtcgataattcagcaaattctaacaagaaatttattgccaagttcaaaggtcct

tacatagttcacaaacaacttccgaacgaccgttatgtaattcgagatatcgatggtttccaacatactc

aaatcccatatgatggtatacttgagtctgataagcttagacattggattgcgccagatgctgatcttgc

cccagaattatgtgatgcgacactacatgatgagaatgtttaacgaattgaggacaatttatttgtcagg

ataggccgagt

>gypsy29-ltr_ag gypsy anopheles gambiae str. pest

tgtaaaagtttggaaaatgtatatacgtgtaaacgctacttgatgtcacggtacgtgtatattagcatta

ggttatttttgacagctgccattagaacagaataaaaagcgaacgaatcaggacgccaaatcgaagtgac

tacaatttaatcgattttaca

>gypsy3-i_ag gypsy anopheles gambiae str. pest

ttttggcgacgaggattattactgaacccggaacccgaagctcacgagattctgaagctaacgagaatta

gcgtcaaggatgaacaatgaaaaccttgaaaccgttatcaaccaaatggctcaacttcttcaacagatgg

ctgcttttaaaactagaagtcctgacgaaatccttgaatctttatcgaaaaccatcgaggagtttcgttt

cgatgaagaaaacaacatcacttttgaaaaatggtatgtacgtttcaaagacctttttcaaaacgatgcc

agcagtttgaatgacgaggcgaaagtgcgacttttgctgaggaagctggaaacgtccgctcacagccggt

atgtgaattacattcttccgaagcaaccgcatgaaaattcgtttgaagaaaccgtcaatattctcaaaaa

aatcttcggaaagcaatattcggtgttccacaaaaggtaccagtgcttgcagatagtgaaatctgcatta

gaagatatcatcacttacggcggaagagtgaataaggcatgcgaggattctgagttcgagaatttgaaat

tagacgatttcaaatgtctaattttcatttgcggactgcaagctccggaattctcggatatcagagcaag

actgctatctcgaatagaaaacgcgacgccggaggctaaagtgaacattcaaacactaatggcagaattt

caacggcttatcaatctgaaagcggatacaacgatgatcgaaaatcaatcaagatcgaagcattccgtgc

atgcagtttcagagaagaaaccgtatcgtttgccacagccttcccgattcgacaattcaaaagatcagcg

tcaaccgaaatcagattcgaatactgtccctcgtactccttgttggcaatgtggaaaaatgcattttgta

cgggattgcaattttacagatcatctctgcaaagtgtgcaaaaatgtaggccacaaggaaggttactgta

aagccatgagatcaaaatcttcaaacaacaacacatatcaaaagagtgagaagaatcaaaatcaaaatca

aaagaaatctcagggaattttcgttaatcatgtcacgaaaaacactgctaaaaggaaatttatctctatc

ataatcaatggcataactacatcactccaactggacaccgcaagtgacataacagttatttctaaaacaa

catggcaacatttgggtcaaccgaaactttctcaagcatccattgaagcatcgaatgcatcgggagaaca

actcaagcttatcggtgaattcgaatgcgaggttatccttaatgcgattacagagaaatgtgtttgtttt

gttacatcatcaccagtcctaaatgttataggtatagattggatagataggtttaatctatgggcaattc

ccttcgatgtactgtgtaagaaagtttcatcagcatgtccaaaggatcgaatagtacagcttcaatcgaa

atatccaacagttttcgatgattcgcttggacattgcacaaaaacgaaggtgaaactgtttctgaagccc

aatgtaaaaccagttttctgtccaaaacgaccagtaccattcaatactatcgctcttgtggatgcggaac

tatcaagactacaatcactaggcatcatcacaccaatagacttttcggagtgggctgctccgatagtagc

cattcgcaagcctaatggtaaagttcgaatttgtgcggattattcgacggggcttaacgaagctttagag

tcaaatcattatcctctacctactcctgaagaaatttttgcacagttgaacggaagtgttgtattcagca

tagtcgatttgtccgatgcatacctacaagtagaagtagaagacgaatctaaacaccttcttacgatcaa

cacacatagaggtctttttcagttcaaccgtctcgcaccgggagtcaaatcagcacctggagcttttcaa

agactagttgacggtatgattgctgacattcctggcgttagaacatttctagatgatgctataattttcg

gtaaaacttgggaggcacacaagcaatcattagacacatttcttcagcgtttaaaagaatatggcttcca

cgtcaagctggaaaaatgtcatttctatcaaactgaaattgtctatttggggcatgtggtagatcgcaac

ggcatacgtcctgacccagagaagctgaaaaccatcgcatctattccagcaccaacgaacatttccgaat

taagatcgttcttaggagctgtgaatttctacggccgatttgtgagaaacatgcatgaactcagacaccc

gttagatcaattgctaaagaaggatacgaagtggaagtggaattcggattgtcagacatcgttcgaaaaa

ttcaagaaagtactgcaatctgacttactgttgacacattatgatccgaatcttcctatcatcgttgcag

cagatgcgtctagcaccggcgttggcgcagtcatttttcataaatttccgaacggatatttgaaagccat

tcaacatgcttcaagaacattaacgtctgcagaacaaggctacggacaaccggaaaaagaagctctggct

cttacttatggggtaaccaaatttcataagtacttacttggtcggaaatttactcttttaaccgatcata

agcctttactatccatatttggttcaaaaaagggaattcctttgcacacagcaaaccgactccaacggtg

ggcattgatgttattaaactacgattttaccatcgaatatgtttcaaccaccgagtttgggtgcgctgac

atgctatcaaggctaatcgaccgttgcaagcaaccagaggaagattacgttatagcttcgatttcacttg

aagaggatattcaaaacgtcatgcacgaatcattgaaacaagtaccagtatcatttgctgacattcaaaa

agcaacaagattggacgaaacactgcaagctgttttgaaattcatccgggaaggctggccgaacgaagcg

tcgtcaatcaaaaatcaagacattcgttcgtattacacacgaaaagaatcgttaacgcatgtagatggat

gtatcttgtttcacaacagagttgttgttccaaacatttacaggaaaaaggtacttcaacaattccatcg

cggtcatcctggaatggtgcgaatgaaatccatttctcgaagtttcgttttctggccgggaatggatgtc

gatatcgaaaactttgtccggcgttgtacttcatgttgcactgctggcaaagcaccaatcaaagaaacac

ctgaatcttggcctgtgccggaaaagccatggtcacgggtacacgtggactatgccggtcctgtagatgg

cgtgtattttttggttgtagttgatccatacacaaaatggccagaagtatatgcaaccaaaactacaaca

gcaaagacaactatcaagtttttaacgcaatcattcgcaacgtttggtgttccagaaactatagtctctg

ataatggtacccagttcaccagctttgaattccaagcattttgcaaacaattgggtatttgtcacatccg

tactgcaccataccatccgcagtcaaacggtttggcagaacgtttcgtggacacactaaagcgtacattg

cgaaaaattcgagcaggaggagagactttagatgaagctttgcagacttttttgcaagtttatcgaacca

cacctacaccagataagtctccagctgaattgatgtttcagcggcctattcgaacagttcagtcattatt

actacctccagtttcacggtcacgaaatgaaaagccagaagctggtagaaaattcttcgatcctggagaa

gcggtttatgcccaagtccaccgcaataattcctgggagtggaaaccggcatcaatagtcgaacgagtcg

gtagagtgaattacaacgtgtttttggaagacaaccagcgaattattcgctcacatgcaaatcaactgaa

gaagcgtctacaggaggaaacatcgtgtggaaatagaaattgtcacgacactggaaatttgttaactatt

ttctttgacgaatttgaattgggaactccacaggcagttgaaaactcaattgaaatgactgaacaagaac

attactattcagctgatgaagagtccgctgaagaaattgaaggagaaaattggcaaacatcatcgtccac

agttactactcaagccactccaccgatgcctgcatcttctgctcaacccgtgaaagcggaaagacctcga

agaattcgtaggccaccagccaggtttgaaccgtattggtgaattaaggggggaga

>gypsy3-ltr_ag gypsy anopheles gambiae str. pest

tgttgcatagtaacacgcataacgcagtaacattgcaagactcgatcagagtacacattgagtgaataaa

gacgattccattctgaactaaggaataaagcagttgtgtttttctcaagatatattccctgcgacatatc

a

>gypsy32-i_ag gypsy anopheles gambiae str. pest

tctcagaagtgggatacgtcctatcgactacacctgttttgctgtttgcaccctcacgataaaaagtata

gcgaaaagtgtgtgcgtgtgtatgtgtgtgcgaggtgacgccacgatccgccatcgcgatgcctacaaaa

gatgagatgttgtgtgctcttgaaagtaagggcatagaagtccccgttacggcttctattccacaaattc

ggaatatgttttctgaaaacgttctacaactaacatcagcggaagcagtcgcaacaacctcacgaggtga

gatatgccccccgtcgagcaccgccatagccgccaccaccaccaccgccgtagctaccgtcgcggcaaat

tgcgcatccaccattttgaaaggcgacaacgataccgattctgccaccattttgagtgcttccgtagctt

gtgctcccatcgcgcttaacaatgagaattcttcttcgcttcctgctaacgccgacaacgaaaatgaact

caacgaaatgcgacgacgcttggaacttttagagcttcgccaaagggtccaaacccttgaatatcaatcg

ggaaaattttcggcaagcgatttgaagctagatggaattattgagccttttactggagacgatgcatcaa

agtgcatcattgaatggcttgacgagctagatcatcacttttctctgtgccgcgtccaagattctgataa

attcttctatgtgtaccgtttactgaaaggatctgctgctatggtcgctaaagcctctcgtgcttcaaca

ctgcaacaattaaaagacgagctggttgcaaacttttatgtgacacccacgaccgaaggcgtctaccgac

agctgcgtaaccgtcgtctctcgccccatgagacagccctgcgttacgtattagatatgcagcgaatcgc

cagtcgcgcatccgttcctgagcctgaattaattaacatcatcttcgaagggcttggcagtccgtcccac

accgctggaatgcgttttctggtcgaaaaagtggaggatttgaagccacttttgaacaagttcgaggcga

ttcgaccacgatatgttgctaaatcatccgagacgccctttcccagtgatcgcaaacgggttacaactaa

tcggggaacaacaccaacaactgtccgttgtttcaattgctctcagtttggtcatcatcagagtgcttgt

acacgccaacgccgcccaccaggagcttgtttccgttgcttccagctgggacacaactataaaagctgtc

cgaatgctgaaaccagcgctgctgcttatccggatgcggccaatcgcgttgatatcgataatgcagaaac

actgccattaaatgaattgcacgaggtgagtgcttcttttcttaagtcagggaaaataatcatgacagtc

aaagaatattccttatttgatacaggtagcccagtaagctttattaatgagaagatcgtacctttatgcc

tgttatctgatccagttccttccaactacaaaggtttaggaaattcgcatctttatactcgtggccaaat

acactgccgaattcaattcagggagacgattctccatcacatgtttgttatactgccacatacatctatg

gcatggccaataatcatagggaaggatctacttccagcacttaatgttcatcttatgtatttcaaagatc

acatcccgcttaaacgaacttgcattatccctacaaaaactatagaggaacaaaaaaggaacgttagcaa

atctggtatattagataatgcctttagtgaaatctgtgctattgatacatttgaatctgaatttaaactt

gatactggcccttctctatccctcgaagagacttcaaccattttgtctatttttgaaaattcttatataa

ataatacttctaaaaataatgaactatcaaatcactgtatgaaaattaatctaactaatcaaactcctat

tttcacaaaacctcggagattatcatacggggaacgtaatcaagtgaaggaaatcgtttcaaacttactt

aaagaaaaggttataagacctagcaattctccttatgcttcggctctggtactcgtgcgaaagaagaatg

gagaggttcgaatgtgcgtggactatcgaccgttaaacaaaattactgtgagggataattacccactacc

gttgattgaaacatgtttagagcatcttagcaacaaaaagtttttcagtttactagatctcaaaagtggc

ttccaccagatacggatggaagaatcttccattccctacacttctttcgtcacccctgatggtcagtacg

agtacaccaaaatgcccttcggtcttaagaatgccccctctgagttccagagatttattaactccattct

tcgtgaatttatcgatgatgaaaaagtggtcgtctacttggacgacattataatagcctccttggacttt

cagtctcacttaaaaacccttcaggcagttctagagagaatcaaacaatgtggccttgagcttcgcgttg

acaagtgtaaatttgcgcatcaagaacttgactatctaggatacaaagcaaatgctttcggtattcgacc

tagtgatcggcacattcaagccattagaaattatccgatgcctattaatgttaaacaactcagaagatgt

ttaggactcttctcttattttcgacgctttgtcccttcgttttcttgcattgctaaaccactcactaact

tacttcaaaaagatgaattatttaactttgatacaagctgttgtgaagcatttgaaacattgcgagagaa

acttactcaatcccctatccttgctatttttgatcccaaaaaggagactgagcttcattgcgatgccagc

tcgttcggctttggtgctgtattattacagaaacaagaagataataaacttcatcctgtagcttatttct

cgaagacaacatcaaaagaagaggcaaagttgcatagctacgagctagagacgctttccgttatctatgc

acttaaaagattccatgtatacgtacatgggttgcctttaaaaatctttactgactgcaattcattggtc

gaaaccttaaaaaacaggaatgcatcggctaaaatagcaagatggtctttgtttttagaaaactatgatt

acacaattcattaccgatctggaacttctatggctcatgtagacgctctaagccgaactgaagccgttgg

agcaatcagcgacttagatcttgattttcaacttcagattgctcaatcccaagaccccttaataaatacc

ctacgacaaaaacttgaagctggttctgttcaaggatttatcctccaagatggtttggtatatcgtcagt

cttccacaaatcatcttcaattatatgtgccacgagaaatggtagacaatattatccgacacaatcacga

aaagattggtcatcttgccatcagcaaaacttgccaaaccattagccaacactattggttccctcatatg

aaaccaagagttgagaacttcattaaaaattgtttaaaatgcatcgtctattcagcacctccaagaacta

acaaccgaaatatgtacagtattccaaagacacccgtaccattcgacacgttgcatattgatcatttagg

acccctacccaacattacatctcgcaagaaatatatattggttgtcatagatgccttcaccaaatttaca

aaactatacgctaccgctacaactaatactaatgaagtatgtgaagctctcactcaatatatgtcatatt

atagcagaccgaagcgaattataagtgaccgtgcaacatgctttacttcaaccgcattcaaagagtttgt

tgattctaatgatatcactcatgttcttaatgcaacgggttccccccaagcgaacgggcaggtggaacgt

gttaatcgtgtacttcgccccattttaagcaaattatgcaattcttctgaccattctgattggagttcgc

atttgagatccgctgaacatgccctcaacaatactgtccatagttccacaaactttcttccttcgattct

tcttttcggcattgagcaacgaggccagatcttagacgaattacatgaattccttaatgataagcatgtt

acaacaaaccgtgacttaaaccttcttcgttctgaagcgttatctaacattgaatattcacaatatcgaa

acgaacaatacgtagccaatagaaccaagccagcccctagtttctctgaaggagatctggtagctatcaa

atacacagattccactaatgcaaataagaaactcgtctgcaagttccgcggcccttatatagttcataaa

gtacttccccacgaccgttatgtaattagagatgttgatggatttcagatcacgcagatgccttatgacg

gagttttagaggtagacaaacttaggaaatggtctaataacatataacatgatgtattactagacagcat

cgtattgctttaattagaatagaattgaggtcaatcctaggtcaggatagccgagc

>gypsy32-ltr_ag gypsy anopheles gambiae str. pest

tgtaatatcttagacatatgaccacgatgcggccactgtgcttcgtgtgtcaaacttgatcatgaccgct

gtcatgatcatcgtgcgcgcggcaatgaacaataaacgtcattcgtttacggatcgtcgtaccggcaaca

tcttcagtcttcttctgtgtcggtttatttcttctgcctttattcttctgccttcggactgtgctcagtt

tgagttcatcggttgatttgaattaattcaaattaattctaatcattaca

>gypsy33-i_ag gypsy anopheles gambiae str. pest

tctcagaagtgggatacgacaaattatagagtgaaaattaatagcgttgtcaagcgtgtgttgtttagcg

tgtcggtcagaatgttgacaaaagaccaaatgctgtgcgccctggaatgcgctaatgttcctgtgtcgcc

gaatgccaccattgcgcaaatacgtaagatgtacgaaactaccttcgaaagcgtacaaaatatgaacaat

ccatatgatgttccatcggttagtggaaagcctaaggaagaaactgcacttgctctacaaagcgagacac

atgttagtgagaacaatgccaccaccatgacgcatgctgagaacgacgtaaccgccattttgcgcaatga

acacaataccgctacgcatgattcaactatagctggaaatccgcaaagcgacacacccaatttaaacgat

gagattgaaatgctaaaaaaaaagcttgaaattcttgagctcaaacaacgaattacagccctagaagctc

caaccgttcctttcccggggccacaaccaccgatattcaattttgaagaaattgtcgataaatttagcgg

tgacaataatgatcacatcgagcgttggttcaaggaactggagcgtgcgtttctcccatacaatatggat

aatacaatgaagctccactatactcgtcgattgctcactggtacggctgcaaaatttgcaaaatcactag

attttacagcctatcatgaattgaaagatagtttgatagaaactttcagcgaaaccaaatcgcttgaaaa

tgtatacaagcagctcagatcacgacaacttggaagaaacgagtccatcacacggtacgttctagatatg

caagccttagcatggggaactccagtaccagaagaagatctggtaaatatcatcatcgatggaatcaatg

atcctatcaacacagcatctattcgattcgctgctcgttctttaactgatttgaagcgactattgaaacg

gtacgagcaaatccgccctcaacacattgcaacaccatcatcatcagggaccatccgccaaccaagtaat

gtgtcaaaaacatcggataaccagcaggcacccgctgtagtgagatgctacaactgttctcaatttggac

actatcagaattcctgccctatgccacgtcgaccaccaggatcgtgctttaagtgtcatcaggttgggca

tgctgctcgaaactgcccaattaaaataatcatcccatcggctgccgcgcattataaagatcagggtaat

gaagatcagggtacaactttagacgaatttgaacaggtgagtgttgccttccaaaatcaaaatgacccga

gcaaagtgttgacatgcgtgcgttctctccttgatacaggaagcccagtgagctttatcgactcttctat

agttccaaaaactttggttaaaccacctctgacatcaagatatcagggtctcggaaaccagatgcttgtt

gtctgtggagaggtgtcttgcaaaataaatctgcgaaaggatgaggcagtacatacatttatcatcctcc

ctagcgatagtattgcctggcctatgattattggtcgtgacctattaaaaaaattcaatatctttctgta

taaaaacaaatcaaaaattgaacttaataaaggacctaccaatcttaacagagaaacatgcatgtcatca

ttggctcagagtattccgttgcctcaagacacattagatttagtaccattcaagttagaaagactatgcc

ctgcagaatatcacgacacccaaaatgaggcatttgaggaaatatgtgcgattgatttatctgatgatgt

ttcagaactccatataggtaaacatttaactggccatgaaaattcagctcttttatgttctataaatcaa

aattatttaaattatccatctgaaaaaattattccacccgatcacagcatgaaaataagtcttactcatg

acacacctttgttcacaaaaccccgtcgactatcatatggagagcgaaatcaagtacgtgaaatagttaa

tgacttattagaaaaacaaataattcgtccaagtaattcgccgtatgcttcgcctctagttttggtcaga

aagaagagtggggaaattcgcatgtgtattgactataggcctctcaataaaatcactgttcgagataatt

ttccgctgccgttaatagaaacatgtttggaacatttaagtaataagcgaatttttacattactagattt

aaaaagtggctttcatcaagtcaaaatgcacgatgattcgattaaatatacctcgtttgttacccctgat

ggtcaatacgagtatgtcaaaatgccattcggattaaaaaacgctccttctgaattccaacgatttatca

ataacatattacgtgaatttattgaggcagacaaattagtagtttatttagacgatatcatcattgcttc

agttgacttcaattctcatttgagcactcttagtgctgtccttacgaaaatacgccaaaatgggttagag

ctccgtttagataaatgtaggtttggtcaacaagaattagattatctggggtataaagcaaattcttgtg

ggatacgtcccagtgacaggcacatagcggttattaaaaattatccgataccgacaaacaccaaacaggt

acgcagatgtctagggcttttctcatatttccgtcgtttcgtcccttcattttcacacatagcgaaacca

ttaactaatttgttacaaaatgaaaagcggtttgatttcgattctacgtgtgaaaatgcattcaaatcct

tacgagaaaaactcattttatctcccgtattggcgatatttgatccaaaacgtgagacagaactgcactg

tgatgccagttcttctggtttcggtgcggttttgttacaaaaacaagatgatggcagattccatcccata

gcatatttttctaagagcacaaccacggatgaatctaagctccatagttatgaactagaaacattgtcga

tcatatatgctttgaagcgctttcatgtttatgttcatggaatacccgtaaaaattgttacagactgtaa

ctctctcgtggaaaccctcaaaaaccgtaacacctcagccaaaattgctcgatggtcgcttttccttgaa

aattacgaatattccattcaacatcgtgcgggttcctctatgaatcatgttgacgctttaagcagattgg

aatcaagttgcgctgtcaatgaaattgatttggactttcaactacaagtaactcaggcccgagattctgt

cattgaggaaataaaaaaaaacttagagttggggcctgttgcaggttttacattgcaggacgggttagta

tatcgcgtatcaccttccaagggactacaactatacgttccacgtgaaatgtcagaaaacatcattcgtc

atgttcatgaaaaaataggtcatctagctgtcgacaagacttatgacaaaattggtacacattattggtt

tccgtatatgaagtcaaaagttgaacatttcattcgaaattgtttaaaatgcataatttattcggctcct

actcgtattaataacaaaaacctacatagcatcccaaaagaaccagtaccgttccatacacttcatatag

accatttgggtcccttaccttcaattagatcacaaaagaaatacatattagtagtcatcgatgccttcac

taaatttatgaaaatgtatgcaactcgctcaacaaatgcccaagaggtctgtaatatcctcaaccaatac

atgtcatactatagccgcccaaagcgaatcattaccgatcgtgctacgtgtttcacttctaaccaatttg

aaaatttcctcgaacataatggtattcaacacatcttaaatgccacaagttccccccaagctaatgggca

ggttgaaagagtcaatcgcgttcttcgtcctatgcttagtaagctctcggactttcatgatcatagcgat

tggagttctcaattacgatcagcagaatatgctttgaacaacactaaacatgcgtctacaaactttactc

cttctattcttctttttggcatcgaacagcgaagttgcgaagtggatgaattggaggaatttctggatga

aaagaatattctaaatgtaaatcgaccactgatcgagatccgtcataaagcatcagaaaatataaaacga

tcacaggagataaacgaaaattacttcaataaaaatcataaacctgctaccaaattccaaaaaggtgatt

ttgtagttatacgtaacgttgacactaccacgaacactaacaaaaaactgattccaaaatataaagggcc

atatgtcattcataaagttttaccaagcgatcggtacgttatcagagatatagatggttgtcaggtaact

caaatgccatacgatggtgtgttagaagcgaataaactaaagaaatggatagaaccatgcttgcgtgttt

aggctaatgatgattaacggcaacatttaccttagattagtttaagaactaggctaggcttgaattgagg

gcaattaaatgttcaggatggccgagc

>gypsy33-ltr_ag gypsy anopheles gambiae str. pest

tgtaggcgaatgtggatcataaacgtcaacctcatacatcaactgtcaaccgaataatgtccaaaccttg

tacaaatgtaaaatcttggcaacccggccgagctgtcagaaaaagagaataaaaaggggaattcacgttg

tgtacgcagttggaaaagcacgtgtaattctgtgtcttatgtccttcagtccttaagaatattttatatg

acgttgtttaca

>gypsy35-i_ag gypsy anopheles gambiae str. pest

tttggtgtcagaagtgggatgaaaatccagcaaacagtggttttacgtgtagcttgtggaatgaacaaga

gtgattagaacgttgagtagattgatctaagaaaatcaattgaagtgagggaaaagtgatcccgtgacta

attgatttgaggtgatcagagttagactcaaatttctgtggagtgaaagtcagtcaaagtgaaggaaagt

gttcgttcctgtgaccgattgattttcgccaaaaaagttgactgatcaaaaatgaatgagctggagcagc

gcattcttgatctgtgtgaagaattcacgaagactggcctagcgaaaaggtgccagcaaaaagggttgcc

ctcaacgggaaccaaagaagaaatggcaagatcactagtggaaagtgaagaaatgtccatcgcggatgat

tctacaatggatcaatttcatgacatggaggaaaaatctgaagcggcagctagcagtgcagcagcgaaag

tggagccgagcatagtacagcagccctactcctttagggacgtggaagagggaattgaggcgttttgtgc

agatggctcggtggattttgctgcgtggctgaccgattttgaggatgttgcgaccatggcgggatggtca

gatgagcaaaaatatgtgatgtgtcgaaaaaagttggtgggcacagcgagaagttttttgctaacgatgc

gtggggtctcatcgttcggggctcttcgcaaggcgctagtagcagaattcggagaaaaagtgcgtccgat

ggacattcatcgtcaacttgcatcacgacgacggaaaaaaggggaatctgctttggatttcatttactct

atgcagcgaattgccaaacagattgaccttgacgaagaaagtgtgtgtgagtacattgttgatggtatcg

cggagagcgaagcgcaacgcgcggcattgtacgaagcgcgcacggtgagagagttgaaagaaaaaattgc

ctggcaagagcgtgcagcgcagaaacaaacacgtgcggtgagatctgcatggcacgagcgcgcggggcag

aaagagaacaaaagtttgccggttcaagacacaattcctgtgaaaaatgcaacaaaagcacgttgtctta

attgtggtgtgatgggacatatggtacggaattgtccagaacaaagaagtggaccacggtgttttcagtg

caacgagtttgggcatcgtgcgaatcagtgtgggcaaaggaataaacaacaaccgggtacaagtacaaac

ctgataaaccatgtccaagctgagctaccgacaaaaatggtccagttggggggaaaaaatataaaagcgg

ttattgacacaggcagcgaagtgtcgattgtccgtcaagacacattgcaagatttagggtgtgtacaaca

aaggatggaacaatccatacaacaattgcgtggttttggtggaatcatacaaaagccgataggcgaattg

gtaacaaacattggcatcgatgacgttgaatacgaaatcggtttgctggtggtctctccaaattcgatga

aggttccaatgctggtaggcatgaattttcttcgaagtgtttgttacgctataaccaacgatggagtgca

gatttttaagaaaaaaagtgacaatgaggaatcgggtagcgatcacgaaagtgctgtgtataagattgat

tgtgaacaaatccaagaactggaagtgccggagaagtttcgagacaaggtacaagcgctgaaaagtcaat

acgaagcttcagtggtggataattacgaagacaattgtcccatacaaatgaagatagtgttggaagaaaa

cgcgtcaccttttcggcatacgccccgtcgcttgcctttaatggaggaagaagcagtcgaaaaccatgta

ggggaatggttgaggaagggaattgttcgtccatcgacgtcagactttgcaagtcgcgttgtggtagtga

aaaagaaagacggcactagtcggatttgtatcgactttcggaaactgaatacaatggttctaaaggatgg

gtttccagttccattgttagaagaagttttggagcagcttcagagtgcaaaggtgtttactgttatggat

ttggaaaacgggttttttcatgtgccgatagaagaagctagccgcaagtataccgcattcgttacaaaat

cgggattatttgaatttaatcgtgtgccatttggactatgcaactcgccagcggtgttcataaggtttgt

gaattatgtttttcaaaatttgttaagagagaatgtgttgcatatttacatggacgatattgtggtttgt

ggtagtacggctgaggagtgtttagaaaaaatggggaaagtgtttgaagtagcagcccaaaatggattaa

aaataaagtggaaaaagtgtcgtttccttcaaacatcgattgagtttttgggacatcatattgaaaacgg

atcgatttggcctggacaagaaaaagttagtgcggtgaaaaatttccctataccgaaaaacataagagca

gtgcaagcgtttttgggtttgacgggattttttaggaaatttataaaaggatattctattatggccaagc

ctcttactgacttgttgcggaaggaggtagattttaaaatgggtagtatagagatcgaagcctttaatga

tttgaaaagtgcgttagtgaaagaaccggtgctcaaaatatacgatcgaaatgctaaaacagaagtgcac

acggatgcttccatcaaagggtttggggcaacaatgttgcaatggtttgatggagagctgcatcccgtat

atttttggagcaagaaggcaaccgatgcagaggccaaacatcacagctatgtgttggaagcaaaggctat

ttatcttgcgttgaaaaaattcaggcattatctcctggggataaagttcaagcttgtgaccgactgtaat

gcattcaaacaaacattgaagaaagcagacgttccgcgagaggtggtgcagtgggttatttgcttggagg

agtttagttttgaggtggaacatcgtgccggagaccgattgaaacacgtcgattgtctcagtcgatatcc

acagttaacaatgatggtgacatgtgaagtgacggctcgtgttaaaagaagtcaacaaagggacgattca

ataaaagcgatagttgaaattcttaacaccagaccgtacgaagattacaaaataaaaggagaacttttat

acaagtgtgtcgaaggacaagaccttttagtgattccgcgtgatatggaaaaacaaataatttcggacat

tcatagtgagggtcattttggactatgcaaaaccatgcacgccataaaacaacggtattttataccgcat

ttagagcgaaaagtgaaactgattctcaacagctgtgtgaagtgtatcatccacaataagaagttaggac

gaaaggaagggttcctgcaccccattgacaagggagatcagccactgcaaacccttcacctcgatcatgt

agggccgatggacgccacgggaaagcagtacaaatacgttttgactgtagtggatggtttttccaagttc

gtctggttgtacccgacaaaaacgactggagcggaggaaacgttgaggaagctggaatgctggtcgacaa

tttttggctatccggctcgtatcgtaaccgacaggggctcagctttcacagccaacgcatttggtgagta

cgctaatcgccatggtatacagcacgtcgtttgtacaacaggagtgccgaggggcaacggtcaagcggag

agagtgaaccgcaccatgttatcggttttgacgaagttgtcatcggaagatcctggaaagtggttcaagg

tggtgccttctgtgcagcgagcgattaactctcatattcacgtagcagctggaaagtcgccattcgagtt

gatgttcgggacaaagatgaggacgtcggaggtggaagacttgtcgcggttactggaggaggaagcgtat

gatcgttttgattgtgaacggcaggaaatgcgaaaaggagcgaagaatgacatttgtaatgcgcaaaatg

tatataaaaaacattatgatttatcacgaaagccggaatatgcgtacgtggtaggagatttggtagccat

caaaagaacacaatttgtagccggaagaaagcttgctagcgaatttttgggaccgtatgaagttattaaa

attaatcgtaacggacgatacaaggttaaaagggcagccaattgcgagggaccgaacataacaacaacaa

gttgcgacaacatgaagttatggtcgtttgccatcagtaacgacaaagtattttcggaagatgaagcaga

atcggaaaatgaataaaatgaattagaagaaaaatcttcaggggctgaagatagggtaggacggccgaa

>gypsy35-ltr_ag gypsy anopheles gambiae str. pest

tgtaagaaaggaaaagcgctagtggtagtgagagatagaaataacggtagcgagatcaggagaacgagag

cgtactaggaacgagttgcgagagagagaacgcgcagcgttaaattcgggagcgcgggttaagcgcgagg

agttgaattcggaccgcgaagcgaataacatgttgtgaactaaagtaatcaataaagcgttatttgtctt

aaaccgaataaaaaactccaca

>gypsy36-i_ag gypsy anopheles gambiae str. pest

tttggtgtcagaagtgggatcacgacgaacgcgctttgggacaagtgtgaatttcctggaaattcgattg

cgatcgacgttgtgtggagtgagacagtgaggcatcaaagcgctgggtgcgctggacggagcacaaaaga

acagtgtacgagtgtagcggcgtttcgtacgggtgttcggtgcgatcaggcgctgggtgcgcaagttttg

gcggtgtgaaagaaggcgctgtgtgcgcgagtagttgtgcgtgtttatgcgttggtgcgctgtgtgcgcg

tgaagttgcggcttgcaacaaaaaggcgctgggtgcgcgagaagctgtgtgcgttggtgcgctgtgtgcg

cgggaagttgcggcttgcaacgaaaaggcgctgggtgcgcgagaagctgtgtgcgttggtgcgctgtgtg

cgcgtgaagttgcggcttgcaacgaaaaggcgctgggtgcgcgagaagctgtgtgcgtttttttttgcgc

tgggtgcgcgagtgtgctaggtgaaaaaaggattagcgctgtgtgcgcgtgaagttgcggcttgcaacaa

aaaggcgctgggtgcgcgagaagctgtgtgcgttggtgcgctgtgtgcgcgtgaagttgcggcttgcaac

gaaaaggcgctgggtgcgcgagaagctgtgtgcgttttttttgcgccgggtgcgcgagtgtgctaggtga

aaaaaggattagcgctgtgtgcgcggtgaagttgcagttagcatcgaaaaaggcgcggggtgcgcgagaa

gctgtgtgcgtttgtgcgctgtgtgcgcgtgaagttgcggtttgcaacagaaaggcgctgtgtgcgcgag

tggctttcggcgtttctgcactgggttcgcaagttaagcggtaggcaaggagattggtgcggtgggcccg

aaaagctgcggtttgaaagaggtgtgctgcataactaggcgtcgtgcttgtaagtgctgggtgcctgagg

agcagacgctgtgcgtgtgtgtgtgtgaggaactaacgctgtgcgtttggggtgcgaaaaagagttgcgt

tttctagcattttggcgcgttatatacaaaaaaaaatggaacgttttctaagcgaatatacgcgtgcggg

attggttcggatgtgcgaaacggcgggactagcactgtccggaaccaaggaagaattagctaagcgttta

ttagaagcaggggtaacaagcgacggccagcgcgaggaaaatatcggcgaaataaacgcgtctggatatt

ttgacgtggatgcggattcagaaactggcagcaatggtgcgactgcggcgggtggtgctatggctgttac

cgcggaatcgagagcaattgtgacagaagtggtgtcacaaaagatggatgtgcctagaagtatagaaaca

catgcaccgtttgtgcagccctattcgttccgcgacgtggaagaaggcattgacccttacggcaatgacc

ttaccaaagacattggggcatggtttgacgatttcgagggagtggccaacatggcgcactggacagacga

gcaaagatttattatgtgccggagaaaaatggtgggagttgcgcggagttttctctcgactgaaagaaac

ataatatcgtacgcagctttgcgatcaaagctgatgaaggaatttggtgaaatagtgcggtcgagtgatg

tgcataggcggcttatgtcacgggtaaaacgtccccaggaaacaatgctcgaatacgtgtatgagatgca

gcgtatagctcgagatacggaaatcgatgttgagagcatcatcgagtatatcgtggacggtgtggcgtgc

gacacaaaagtgcgtgcatccctttatcgagcgcgcagtatggccgagctgaaagaagagctgttgcgca

tggagagagcggagaagaaaagtgttgtgaagaggaacaatggtcaacaagatcaaaggaatggtggggt

aggtttgcggaagtgttatatttgcggggagttagggcatgaagcacaaaagtgtgttaagtcaacaaaa

tgttttgaatgcggtagatcggggcatcgggcgaaggattgttctatcaagaagcgactggtagctgaca

cgcgcgctattgtaccgagtacaaatgagcgatctccggtaaagtgtttcaaatgtggaggcgtggggca

tatcgctcgaaactgtaataaaaatagctacagcgtgatgggaggtcagatccaggaatcaaataaagaa

ttaccaacaaaacaagtgagcgtgtgcgggaaggaatatgcggctctgatcgacaccgggagcgaggtat

cactcatgcgtgaagatgttttcgcgggacttccaccgcagttcaaacaatggaaacaatcgaaccacat

tttgagagggctaggtggagtgccccaaaatggattcggcgaagccacacttgccgtaaacatagacaat

tgggtatatcaggtgcagtgggttctggtaccatacgaggcgattagaacaccccttatcgtaggtatgg

attttttgcattctgttaattacaatattaacaacgaaacggtcatgatcgagccccgaagaaacgagca

tgtgttggggatacagagaaatgaagaagaacacgaacgacaacagcatgtgaacaatattttaagcgaa

gtgatgaacacaatgtgcgccagcgagacaatggaaccggttatcccgcctcaatttcggggcgagataa

tgacaatgatcgagacatacaataataccagctgcagtgagagaaaagaaacgtgtccagttaagttgga

aattgttccagacggacagataatgccgttccgccacccaccaagcagattgtctttttcggaatctgaa

gcagtggatgcgcaagtggatgagtggcttaaagagggtattgttaggccttctgtgtcgaattttgcga

gccgcatagttatagtgaagaaaaaggacgggagcaaccgggtgtgtgtcgattatcggaaattgaattc

gatgattttgaaagacggattcccgataccagtaatcgatgatgtcgtgcaaaaactacaatgtgcacaa

tggttcacggttatggacctggagaatggatttttccatgtgccggtagcagagaatagtaaaaaatata

cagcgtttgcaacgagacgagggttgtacgagtttaatcgcgcgccgtttggtttgtgtaattcgcccgc

agtgtttatccggtatgttaatcatgtgtttcgggatcttattacaaacaacctactggatttgtacatg

gatgacatggttatccatggctcaaccgaacgagaatgtttatggaaaacggaaagagtgttaaaagtgg

cagcagagcacggtttgaacattaagtggaaaaagtgccagttcatgcaaagggaaataacgtttttggg

tcatcgggtgaaaactggacagatatcgccaagtgtggaaaaaataaatgcagtgaaacattttcgtatt

ccgcagaacgttaaagcagtgcaagcgtttctgggattaacggggtttttccggaagtttgtaaaagact

attcgaaaatcgccaggccgcttacggatttgttgaagaaagatacatctttcgaaataacgggaagtgc

attggaagccttcaaacggctaaaagaggaattaataaaagagccagtgttaaggttatttgatcccgaa

gcaaaaacggaacttcatacggacgcttcaaaagcagggtttggtgcaacattgttacagtgggtagaag

gaaaattgcatccagtgtatttttggagtaaaaagacaagtgaaccggaatccaacaaacatagctacgt

gctcgaggcaaaagcagtgtttctagcggttaaaaagtttcggcagtatcttttgggtcgtccttttaaa

ttagttactgactgtgcagcgtttaagtgtacattgaaaaaatcggaagtgccgcaggaagtgttaccat

gggtgatgttcctacaagattttgtgtttgaagttgaacaccgatcgggtaaaaaaatgcagcacgtgga

ctgtttgagccgatatccgacggaagtgatgactgtgacaaatgaactaacggcacgtattcgtaagaac

caacaacaggacgagatggtgaaagcaattagtgaaattctattggataggccctacggatcgtataaat

tgaaaggtggacttttatacactgtagtggatggaaacgaactattagtgattccacgaaacatgaggaa

gcagatcatagaaaacgcacataacgacgggcattatggggcacagcgtactatgcataccataaggcag

aagttttggattccacatctagaggtattggtgaagcaacatatctcgaactgtgtgaagtgcatcttac

acaacaaaaagttaggacgacaagagggatgcttgaacccaatcgacaaaggagatgcgcctctacgcac

attgcatttggaccatgtggggcccatggacgccacttcaaaacaatacaggtatattttaaccgtggtg

gatgggttttctaaatttgtttggctttatcctactagaacaacgaatgcggaggaagtgttgcaaaaac

tggagagttggtcgtcgatattcggatatccgacccgagtaataacggacagaggggcagcgttcacggc

taaggtatttgccgaattcgttcagaagcaaaacatcgagcatatcgtctccactacgggagtaccacga

ggaaacggccaggcggaacgtataaatcggacggttttatcagtgctgggaaaactatcgatgggagata

ccgctaagtggtataaacaagtcaacagagttcaacggtcggtaaacgggcaccttaattcgtcaacagg

gcgatctccatttgaattaatgtttggggtcagaatgagacaggcatctgatgatttattacacgaaatg

ctggaaaaagaatggtatgaagaatacgaacgggatagacagggtatgagacaagaggccagaaaagaaa

tagagttggcgcaaaagagatataaggagcaatttgataagaaaagaaagccggaacacgggtataagat

tggtgatttggtagcaataaagagaactcaatttgtggcaggaagaaagttagctaatgaatttttgggt

ccgtacgaaataacgaaggtaaaaagaaatgggcggtatgacgttaaaaaggcagctagttgcgaagggc

ctcaagttacgagtacgagtgacgacaatatcaaactatggtcatatgctacgattgaatcgtctgacga

agacgaattagatgaagatcaggaattgaaggaataaggataagtaaactaggtgtaggacatcgaggat

cgaagtccatcaggaagggccgag

>gypsy36-ltr_ag gypsy anopheles gambiae str. pest

tgtagaattgtatgtaaagttgaaagctagtattgcctgatgcaatcgggataatattataaagaagaga

acttgggctgagcagtcagtcgttgatcagaactcaataaagaaacaccgtcaaaagagaaaaagtctgt

gtcaacgttctccctcattcggtccacctcctccaccccctcggttggttttgcggttgtcgtgatccgg

ttgtgtcgagtattgttctccagtgttggagtattcggcgccttccctcattcctttccgctctcgtgtc

tcccctttcttaca

>gypsy37-i_ag gypsy anopheles gambiae str. pest

tgggggctcgtccgggaacgggctaagtacggtgataaagtgaaacgacgaaaaaccaagagtgaataat

gacgacaatcgaagagctgtgcgaaaaccttacgaagatcggcctcgcacgcaagtgccagcagcttggt

ttggcgacgtccggcggtaagcaggagatggccaaaaggattattgaccatacgacgatggccacaaacg

acgagaacgacggtgatcatcagaatcgtgaaattcgtgaagccgtgaacgctagagagcagaacgccgt

gctgcaaaacgacgacggtgtgagtgagaacgacgacttacacacacacaacgacgacaacgacgatcga

gaagatggcggcgcgcgtgcaaaatgttcacgcaacgacgacgacgaacgatacgatgatgatggtgttg

gtgacaacgacgacggcggcaacgatgattacaacgatgatgacgacgatgaagacaacgatgatgacga

tgatgacgacgcattattgttccaaacggcgatcagaacttcgaccccagcggcaaggaaggaacggaaa

tttcatggaacatcgatggtttattctttccgcgacatggaagaaagcatcgacacttttggtgctgatg

aaggtgaggacgtacgattatggctaaaacaacttgaaatgatttctaggtcggcacgatggaataatga

gcaaatgctgattatgtgtagaaaaaaactgacaggtacggcgagacgttttgtgttttccttacgcgat

gctagcagttatagtgttgtgaagaaagcgctaatcaaagagttcgctccttttgttcgagcaactgacg

tgcatagggcattagctaatcggaagaagaagccaacggagacgatgcgtgattacgtgtatgaaatgca

gcgcattgctttgcctattgagttagatgaacctagtttatgtgagtatattgtggatggcgttactgac

gacgaattttatcgctccacattatatgaagccaatacggtacaaaggctgaaagagaaactaagcattt

ttgagaaggcaacaaagaataccaaggtagctaagaagaatcgacacgatgacaatgctgacgttaagaa

agaaaggcaacgctttgaagggaagtgtaaaggtgaagtgaaacgtaaatgtttcaattgcggcggtacc

acacatgttgcttcagagtgtccgaagaaaaacgatggtccgaagtgctttagatgcaacgattttggac

atctttccaaagagtgtcccaaaacacaaaagacgaacaagaaagataatgacgcacgaatcaatgtagt

gaaagcggctgttaatgaaaaactcagcgtatcagtggaattgtttggacagaacatcaaagcagttgtg

gatacaggtagcgatatttctctaatgagatttgatctttttgaagaattgaaaagtaaccacattcaga

tgcatgaatcgaacttgaaggtacgaggatacggtggcggcgtaagctcagtacgtggtaaaacgacgat

atctgcaacaatcgacgcagagatttttgatattcaattctatgtcgttccatgtgaagccattgattca

ccaatgttgatcggaatggaatttcttagttcagttgactattctatcactccagaaggagtaacgataa

agaaatatcgaacagacgagacagttggtatagagacgaaatggattcggcgtattgcaggatacattga

tgaaaatgaattattggttccaaccaagtatcgcgaagaagtaatgacgctaattgaagactataagcca

cagaagaatatgactgatagaaatcagctgacgattactttatcagatttgaatgttgtttgtgagaacc

ctcgacgattggctctgttggagaaagaggtagttagaaagcaaatcgatgaatggcttgacgaaggaat

catacaaccatcgcaaagtgaatatgcaagtcctattgtcgttgtgccaaagaaggatggttcgtaccgc

gtttgtgttgactaccgtgaattgaataagaagatcgtgcgtgacaaatttccaatgccaaatgttgaag

agcaagtcgatcagctcgctgaagctcgagtttacaccactctcgacttaaagaattcctattttcacgt

accagttgatgaaagtagcaggaaatatacggcattcgtggcggatagtggtcagtatgaatttttaaga

gcaccatttggactgtgcattagtggaagcggttttggtagatttattaacgatgtgcttcgagaattta

ttcgagatggaacagttttggcgttcgtagacgatataatcattccgtctaaaactgaagaagatgggct

gaatgcgatgaaacgtgtatttgaagtagcagcgaaggctggattgacgtttaactggaaaaagtgcgta

tttctacaacgacgggtagagtacttggggtacacaatttacgatggaaaaatagaaccagcacccgcga

agatcgaaaagttgaagcagtttccacaaccgacgaccgtgaagcaactgcaacgattctatgggttagc

aagctattttcgaaagtttgtcccatcatttgctggtatcgcacgaccgttatcggaattattgaagaaa

gatcgttttactgaattgaacgacgaagcgatgaattctttcctatgtttaaaggatatacttgcagctt

atccagtactacgcatttttagagctgacggagatgtagaattgcatacagatgctagtaaaacagcgat

agcaggcattttaatgcaacgagcagaagacgatggtaaatttcatccgtgttattactttagtcgactc

acaagtagtgctgagaagaattatcattcgttcgagttggaggcattagctgtagtagagtcagtacgga

agttcagatgctatttgcttggacgtacatttaagatcgtgactgattgcatggcattcaaggactcagt

taagaagaagaagttgaacgcaagaattgccaagtacgtattagctctttctgaattcgattacgtaatg

gaacatcgaccaggagaaaagatgccgcacgttgacgcattgtcaagagctaatgtgatgatcatttcaa

caaccatcttatcgaagatacgaatggctcagaataaggatgaccgagctaaggctattatagcaacatt

ggaacgaggagatacggttgacaaatttattttgaacaatggcgttatttacgaaaacgatggcgataaa

cgacgactttacgttccaaaatcaatggagatagacatcataaggtctgctcatgaacaaggacattttg

gcgtccgcaaaacaaaagaacgcattaacgcagattattttattgttggattagacgaaaagattaagag

ttgcattgacacgtgtgttccatgtatcatcggcgaaaagaaaagaggtaaaccagaaggtgaactacaa

ccgatacctaaaggagacgtaccattggatacgttacacgttgaccacttaggaccgatgccgtcgacaa

agaagtcatatggatatattcttacagtcatagacgctttcacaaagtttgtctggttgtttacgactaa

atcaacaactgcagaagaggtcgtaaagaagcttcaggtgatcacgagtacgtttggcaacccacgacga

atcatcagtgatcgaggttctgccttcacgtcaggacatttcaccaggttttgtgaaaacgaaggcattg

agcatcacacgatagcaacgggagttccgcgaggaaatggacaagttgagcgtgtccatcgtatcatcat

cccaatgcttacgaagttgtccatggagaaaccggaggaatggtttaagcacgtagcgcgagttcaaaaa

tgtttgaacaatagctggcagagaacaattaacatgacaccgtttgagctaatgaccggtatcaagatgc

gtacgaaggaagatgctgtactacacgaactactatcacgcgagatacagaatgatttcactgaaggaag

agatgagttgcgcaggacagcgaagcgtaatattgaaaaaatgcaagaggagaattgtaagtactacaat

ctgcgtagaagaccctcacgacagtttaagatcggagatcttgtagctataccgaagacgcagtatggag

tagggcaaaagtttaaaccgcgcttttatggtccgtatgaaatcacgcgtattttagataacgatcgata

tgaagtaaagaagttagatgaggagacagaagggcctaagaaaacattaacagcaggaagttgtattaag

acgtggatacttccggggcggaagtaatgtcaggaaaggccg

>gypsy37-ltr_ag gypsy anopheles gambiae str. pest

tgtaggatgagagggctaccccccgaaaacgtcaaaatgacagctagatagcgagggcatgacagcaggc

gatgagcgacacaatcttaccgcgagcgtcaccgtgaacagaagcaagaatatacgaatttcctttaatc

acctacaccgctcgtttccttatttcttaca

>gypsy38-i_ag gypsy anopheles gambiae str. pest

tgggggctcgtccgggaagaggctaggcgatgcaaagtgatgagtttacagttatgaaaagttgcataat

gtgttaaagttgataaagttgttcaagtcgtaaagttgataaagttgtaaagttgttaaagtcgttcatg

tcgtaaagttgataaagttgtaaagttgttaaagtcgttcaagtcgtaaagttagtaaagttgttaagtt

gcagtagtggttcgaattggaaaagttgacttagtcggtgtttcacctgatgtgaaagtgtgaatttgtg

acgaaaagttgacattgtgacgcataaaggttgttaaagttgcgattgtgacgttgtaaagtgtgtttaa

ataaaagctaaagttgtgcaaaacgttaatgagaaaatggagaacgcgttgattgcagcgatggtcgaag

accatacgcggttgggtttggtgaaaaaatgtgaagagcgtgaattaagtacggtcggaacaaaagagga

gctggccgcacgcgtcgttgcgtatgatcgaaccgatgcagctgattttgcggatgctgagttgcccacg

gttgacagagtgcaaccatactccttccgcgatgtcgaagatggtgttgatgcatttggcgccggatcta

cgcaagacgtgcgcgcatggatcagaagttttgaggagataagtacgatggctggatggaacgaggatca

gaaattgatcatgtgccgtaaaaagttgaccggagtcgcacgtggttttctaaatacgctgaaaggtgtg

acgacatatgcaaatctacgtagagcgttgattaacgagtttgctccaagtgtgcgtgcaagcgacatac

acaggcagctatcgacgcgtcgcaagaaaaaagaggagaccgggttagaatttgtttatgccatgcagca

aattgcgcagcaaatcgatttagacgaagccagcctcgtagaatatgtggcagatggaataactaacgac

gaaaggcagaggtcaatgttctacgaggcaaagaatattaacgaacttaaggagaaaatacgtttgttcg

agagagcggcgaacaagtttcaacaggaacgcccaaaagttgtcgggcaacgaattccggataagttaaa

acaacgccattgtttcaattgtggcagcgcttctcatcgacttatcgaatgtccaacaaaacaagaaggt

ccgacttgtttcaaatgtggaacaaaaggacatagtgcaaaagattgtacgacagcgcaaggaagaagac

cgcattgttatgcttgcggcgaagttgggcatgttgcaacacgatgcccgaataacagtaataataatag

agttcaaacaatgacgagctcatttccaattgtctgtgtgtcagttggaaaccagttgtgcgaagcggtg

atcgatacgtgcagtgatgtgacgctcatgcgctgggatttgttccaggaattaaaactcaattgtaatg

agttgaaaccgtctgcacgggtaataaaaggctatggtggaaaacaaagcaacgtgtgtggtgagctgac

gatccgtgctaccatcgatgaagttgaggaaataattcgtttcgtggtggtaccttcgaattctatcgac

accaaaatgctgataggaatcgatgtgctcaaaagggttaactacgttatcacaaacggtcatgcaaaga

taacaaagcaacaggaggaagaggaaaaacccacgggtgttgatgatcgttgggtgtatcgtatagtttg

tgaaagtcatcaagaaatagacgcaccgtcaaattatcgtcgcgtgatttgtgatatggtgaataattat

gaaccagcaaagatttctaacgttgaaagtgagatgaaaatattagtgaacaacgaggaggtagtgcgta

cgttgccaagaagaatagctccgctagagaaagaggtggtaaggaaacaagttcgcgaatggttaaacga

cggaataattcagccatcgcgaagcccttacgctagcgcagttgtggttgtaccgaagaaggatggatct

caccgtgtgtgtgtggattatcgtgagatgaataagaggatcgtgcgggactcttatccgatgcccaata

tcgaagaccagattgatcagttggcagatgctcgtgtgtacagtgtattagatctgaaaaattcgtattt

tcacgtcaaggtggagaaggaaagtcgccagtacacatctttcgtaacaacggatgggcagtatgagttt

ttacgtgctccgtttggattgtgtctcagcggcaatgcgtttggacgcttcatcgatgcagtacttcatg

agttgatcatagacggtaccgtgatggccttcgtagatgacatcatcattccgtctcgagacgaggagca

tggtttggcgtctttgcggcgagttttggaagtggcacagcgggcaggacttcacttcaattggaaaaag

tgttcgttcctcaaacggcgtgtagagtatttggggtataccgtctatgaaggtaagattgaaccagcac

cccaaaaaatagaaaaagttaaatattacccgcagccaaaaacggtgaaacagctgcaatgttttatagg

gctagctagttattttagaaagtttatagaaggttttgcgcgtattgccaggccattgaccacaatgtta

aaaaaagagagtgtttttgaatttaatgaagaggcaagggcagcgtttgaatcgattaaagaaaaattag

tggaatatcctgtattacatattttcaaggcgagtttggttaccgaattgcatactgatgcgagtaaaga

tgctctggccggcatattgttgcagcgttctgaggaggatggtttattacatccttgctattattatagt

aggttgactaataaggccgagaaaaattatcactcattcgaattagaatcattagccgttgtggaatcca

taaagaagttcaggtgttacttgctaggtaagaaatttaaagttgtgaccgattgtatagcgtttaaaca

atccttaaacaaaaaagttcccaatgcgcgagtgagtcgttggtttgtaacgttgtccgagtttgatttt

gaagtagaacatcgttcaggtgaaaagatgaaacatgttgacgctctttcaagagcaaacgtattgaaaa

tatcggcaggtgtatgtgagaaaataatacaaggacaattgcgcgatgaagatcttacggcaattaggac

ggcgcttgatcagggcgaagaaagaaatggtttttgtttgataaatggagtactttataaaaatgagaat

gctcataacaaaatatgtgtacctcgagcaatggagacgggtatgataagagaagtacacgaacgaggac

attttggaattaagaaagtgaaagaacaactaacagaggattattttattcctgaagtagaaaagaagat

aaaatattgtattgataactgtgtccgatgcatagtgagtgatagaaaacgaggcagggcagagggagaa

cttcatccaataccgaaaggagaggttccgttggatacctttcatgttgatcatattggccctatgccat

ctactcgcaaatgttacaattacattcttaccgttgtcgatgcgtttacgaagcatgtttggctatttgc

tacaaaaactactagtgcggaagaggttataaaaaaattgacgattatatctgatacgtttggaaatccg

cgaaggataatttgcgatagaggatctgcttttacctctagtttatttacaaaattttgcgaagaagcca

atatagaattgcatcacattgttacaggagtcccacgagggaatggccaagtagagcgtgtacaccgcat

aattatccctacattagcgaagttgtcgtatgaaaatcccgaaaattggtttaagtttgtaaataatgtt

caaaaggcgataaataatagttggcagcgatcgattaggatgacaccttttgaattgatgatcggagtga

aaatgaaagagaaggaagatattcgaattaacgatattttgcaagaagaaatccaaaagatgtttacaga

tgatcgtaatgaaataaggaaaatagcatgcaaagggattgccagaatccaagatgaaaatcgtaggacg

tacaatttgcggcgtaaatcgattcaaaagtataagataggagatatcgtagcgattccagtaactcagt

tcggaataggacgaaaaattaagaggaaattctttgggccgtataagattgtcaaaacattttcaaatga

gcgttgtgaggtactgaagctagatgagcaaactgagggaccaatgagaacgacaactgcattcgatcaa

ataaaaatatgggcagattcaggagaatcatgatgtcaggaaaggccg

>gypsy38-ltr_ag gypsy anopheles gambiae str. pest

tgtaggaaataggagacagttgacagttgtcagacaagggatgtaaggcgaatgtcaaggaatgaattta

ggatcaccctgtgctggtgtcaatcgaactgacgacgcagtaggtttagctagagcgaaacattcacgcg

ggagaagccaagcgaaccagacgaaagtgaataaagtggattgtaaaaccatcgctttccgcgtatcctc

ttttcttcatcgttctaca

>gypsy39-i_ag gypsy anopheles gambiae str. pest

ggcgcagtcggctaggcccaattcacgttcagtgacaagtgttcaagtacagtgcagtgaaataacggaa

ataaatctgtgtatcgcccggactttgctccatcgcccgatccgtggttgactcgtgacgatcgcgtgaa

tttcattatttcataaagtgtaagaacaagtgcacgaacggttggtttcaactatttagaattggaagag

aacattcgaacaaaagctagtggcaagactccccagccagcgttactgggtaaactaatcaacgcaggac

aatcgctgacccaacccgtggacaacgagtgaccaaagaagaaaaggaaggacaatcgctgacccaatcc

gtggacaacgagcgaccaaggaagaaaaggaaggacaatcgctgacccaatccgtggacaacgagcgacc

gaagaagaagaggaaggacaaagcaaaggacaggtaacggaaaactctaatttcccgacacgtggataaa

aaaggtgagttacatcctaaagcataagttcccctactcttataataaaaaggcggtaggcaacaaacaa

tttttatcattgaatgccaaaatatacccctttcccaaaagttcgttctttgaacttcactaaggacaag

aaaattccagtgaaaatggaacaaaatatacacgcattgattgaatctatgcgctcgttagaggaacgcg

ttaacacactccaagcggaaaacgatagtttacaacgcttccaacaacaacaacagccaacgacctcgag

tcgcagtgctgactattttcgtatcccggatccaattagggtcattcccggattcgacggaaacaaaaaa

cagttaatcggttggttaacaacagttaaaaaaacactagacctcttcaaaaacaatgtggcaccagaaa

tattcagtgtctacgaacagaccgtaattaataagatagagggcaaagcacgtgacactatttgtgtgaa

cggaaaccctactacttttgatgaagtcgcagatattttgcagaacgtttatggcgatcgaaacaacatc

gcaacgtatcaaacacaactgtggaacctaaaacaaaacgaatctttgagccttcactatagaaaaacaa

aagaaattttgatcaatatgaaatcagtagcaagacagaatacggtatacgcttcacattgggaggcaat

taacctgtttttagaacaagaatgcctcgctgcgttcataaatggtctgagcaaaacatattttggatat

gcacaaaccgcacaaccagaagacttggaatcagcttatgcctttctatgcagattccaaaacgctgaaa

gaaccaaatcaaacaccaataatgggttcgaaaaacatcctagtgtcgacaaaaatgtaaaaagggataa

taaattttcacaacctaaacaccttgaaaatagaccattcaataacatgaaaccatcttcagatcgtaca

aaaattgttcccatggacgtcgatcaatccttacggtccaatatgagaaacaaaattttctcgcatacag

ccgaagaagatgacggaaaaacaatatcagaagaatccgattcagacgaagaaaccgataatgaagaaga

tgtaaattttcaaattactgcaaacttaaacccaccgagttaaacgcaattaacggactacccttttaca

caacaaaattaaatggttcaaacatcaaactcttagtagacagcggagcgaacaaaaactttttaaatcc

agaattagttccactaactcaaagagtgaaatgcgaaacaatcaccataaaaaataaaaatggtaagttc

aaatcacaagaatgcacattcataaccattctagataaaaaattaaaattttaccttttcaaatgtcata

attattttgacggaatactcggatacgaatcactctccgatatggaaacactaattgatacaaaaaatca

taaacttattctacccgatcgtacaatcgacttagaaataagaacattagaacccccttcaattacatta

aatgctaataattctcaaattataaagcttcctgtttctcaaacaaaaggaaacatttttcttccaaaag

acattcaaataaaagatgcaataattccttcaggcgtttataaagcaaaacaaggatatgctaaagttct

tgccaggaattacggcgacacattaacatttcattggactaaaccagcaaaaacatatgaattagacaaa

atgatcgaaataaatcacatgagcacacacccatataatgatcatccgcaaaattacatagcaaatctcg

aaaatttaattcgcaccgatcatttaaacaaagaagaaaaacataaacttttcgaaattcttaaacaaaa

tctaggcatcatccatcaagaaaaagaaaaactttcatgcaccaccgctattagacatcgaataaaaact

aaagacgatatacccattcacacaaaaacttatagataccccaatattcataaagcagaagtgaataggc

aaatcgaagaaatgattgctgacggtattatccaacattctatatccccatggacatccccaatctggat

agtccctaaaaaatcagatgcaagtggaaaggagaaatggcgcatcgtggttgactatagaaaattgaat

gaaaaaacgatcgatgatcgataccctattcccaatatagaagaaattttggacaagttaggtagaagca

tgtacttcactacacttgatttaaaatccggctttcatcaaattgaagttgataaaaaggatagaccaaa

aacagcttttagcacagaaaaaggacacttcgagtttatacgaatgccctttggcttgaaaaacgcacct

gctacttttcaacgagccatgaataatattttaggtgaccttgtaggaagaaactgtctcgtatatctag

atgatattattatttttggtaaatcactccaacaacatttggacaatttgaataaagtgttgaaaaaatt

aattgaatcaaatttaaaggttcaattagacaaatgtgaatttttaagaaaagagtgtgaattcttagga

cacatcgtaactcaagatggcatcaaacccaatccaaacaaaatagaaaaaatattgcattggcccatac

caaaaacaacaacccacattaaaggcttcttaggaatccttggttattacagaaaattcataaaagattt

ttcaaaactgaccaaacctctcacaaaatgtcttaagaaaggttctaaaataacacataatgacgaattt

ataaattgtttcaatgattgtaaacaaatgctcactactgatccaattttaaaatatcctgattttagca

gaaaattcattttagaaacagacgcaagtgacttcgcccttggcgctgtactttcacaaaaattcgaaga

tggtaaggaacacccaatcgcttacgcttctagaacgcttaacgaaacagagtgcaactattcagctacc

gaaaaagagctactcgccattgtttgggcaaccaagcatttccgaccgtacattttcggaacagtttttg

aaataagaactgaccacaaacctcttgtttggctaagacagaaaaatgatttaaatagaagacttcttca

ttggaaactggccttggaagaattcgaatttgaaattaagtataaaaaaggtactcttaatggaaatgca

gatgcactatcccgcataacggaaaaccctgctttaacatccgaattaaatgcaaatactgcctctaaca

atactgacactatgacgcaacactcagcagatactaatgatgacgaattcatcccagcaactgaaaaacc

actaaatgaatttagaaatcaaataatactagaagaaaataataatgattctcgagaagcaattacactt

ttcggaaattatcatagaataacaataaaaacacccctttttggaccagctatgttgatcaacataatca

aagaatatgcatctcccaaatgtgttactggcatttgttgcagcgaaaaaatcttacaaaacttacaaat

tatctacaaaaactatttctctcgcgcaaaatcgatcaaactattttggacaaaaacaattttagaagat

gtaactgatgaaattgaccaggatttgattattgaacgacaacataatgcaaaccatagaggaattattg

agaccaaactacatattgctagaaaatattattttccaaacatgaaaactaaaattactaaatttattaa

catttgcaaaatatgtcaaaaagctaaatatgaaagacgcccatataaacaaaaatatcaaattactgcc

actcctaaaaaacctttggaaatcgttcatatggacatctttataattagtgataaacattttctaaccc

tttgcgataagttctcaagactaaccatggcgattcctttacaatcaagaaacgctatacatatactaaa

agcactaactcaattcttcgctaacgttggaagaccttgtttgcttgtgatggaccaagaatgttcgttt

aaatccaccacaatcaaacaatttcttgaagaaaataatgtagaattccattatacaagtgttggtcaat

cctcttcaaatggaacaattgaaattgtccatagaacaataagagaattgcacaacataatctcactacg

agattctacaaaaaatctatcaactacttcaaaaattaatttggcagtctcaatttacaatgattctata

cactctcaaacaaacatatcccctaaagaactatttttcggcttcaaaaacgaagatcctatccctgagg

accttcaagaaagaattaaacaaaaagaggaactttatagagtgtatgctacaaaacaaaaagaaaaaaa

atccaaatacatagaaaaattaaacaaaacacgagaagaaccagaactattcaaagacaatgacacgatc

ttcgaaaggaaaagaaataatttaaaacacgaagaaaggtatcgtgaaacgaaagttttcgataacctag

ttacaaatattttagaccgaaatggaagaaaaattcataaatcaaaattgaaaaggaaacgaaaaaccta

acaaaccatttaagttctttctcatccttacggaaatcacagctgaatactataaaaaactaaaaatgaa

atttggaatgattcatttcctcataatagttttaacaaattcaaaacttataacagcaacaatttataga

gactaaccttaactatattcagaatttagccaataatattagcctaaccaaccatcttaaggacacctta

gactataaaatcctatcagcttataaaaaatgaggaaatataagacctagaaaacaaaagagaggcataa

taaatgcaggaaatcgcagatcagaaaacacttagaacattcagaatttagaacattcagaaaacaaaac

catatccaacattaacgctcaagttagaattaattccgaaatagaaaaatcagtgaacaaaattacacaa

actctaaaaaaaaaaaaatcgaaaatcaaatgaataatcagataaaagatcagatcagatcaaatgaata

atcgtaaaaactgaaatagaacaagtaaatcttatcctgaacattgataacattgtacaactgtagaaga

catagaagaatagacggatgtaaaatttgtaaccaagcataaaaacaccattttcaatgttaacgaagag

tgcacgatttgcaacaacccacaaccgctaaacgacgagtgtatctcaaatatcatcagaaatcatccat

ctaaatgtaccaccaccacgagctccgagcatacgataatacgagaaatgaaaccaggagtcattctaat

cgataccacccttggagtgccagtcattgattcatgttcaaacagccaaataatagcagtacccacacta

attgaaaccggcaattgcaccgtaaaaatcttaaatagcacatttactgcgcatattgatgtgttagacc

aagaagactacttcctacccttaactggcagcaaaacagaaataactcttaacaagccaagtatacaaga

tctgcacacaatgcatattagtaacatacatcaactccatacaataacactccgcttacgcacgcataca

attgcaggagggatattaattgttgttctaacaggctttcttataactgcattttgcatctacaaacaca

aaaccaaacatgcagagaagaaaatgtctaccgaagctattcacaacatcatcccactgcaaacgtcggt

aagtcgatcgaggacgctcgacgtttaaggagggagg

>gypsy39-ltr_ag gypsy anopheles gambiae str. pest

agttatgtacacatgcgctacgcgctatgatacaggtgctgagtaagaaaacggtcgcaagcgagccatc

gatgttactccacgcgcggagtcaagttccaacgggaactccactggaagcaggttcccacgaccaggtg

tggtgtcgtatgttcagacggaccgaggcaacatgatcatcataaacgtggacgacaacgtgaccggcaa

acctggcagcaccgaaaacaccagcctagctaaatagtaccgaatccagaagttagctttagtcttagtt

tagcagttcgcaaataaagatccccagtaatgtttttttttaaaacttactccgggctatcgtaaacata

att

>gypsy4-i_ag gypsy anopheles gambiae str. pest

tctcagaagtgggattaccaacaaaaatcgcctacaaaaccgcctgcaagccgcctaaccagcctgtatc

cgtgtgtacctgtgtacgtgtgtgtatgacacaacgccattttccatcatgctgaggaaaaaagaacttc

gtcgggcgcttatcgtcgacgtgccggataccgctaccgtcacacaactccgacagctttatgcctccca

cgagccggtcgctcgttccccccgtgcggcgccgcccaccacctcagcgacgacaccggctcccgcttgt

gcaaaccaccaagatgccgccattttgtgccttccacattacaatggcgacgacgattttgcgcatcacg

aaaatgttgcgaacgctgccgcttcgacgaacaataccactgatgccgtttccgcccttccttctgccca

tggtgtggccgccgcccttccccgcggccctgacgacatcgaggcccaatttgagaagctgcgacagcag

cagcagctagctgaattacgccaaaaggtgcaccaacttgaaacgcagcagccagccgccctttgcgtaa

aggactttgaagctttcatcgagccactcgacgccgataagaaccccaatgtcatccgatggttccgcga

cttggagcgtctctttgcactttaccgagtgcgcgatgcagataaatttttcttcacccttcggctcctc

accggcacagccgctaacgtcgcaaaagaacttgttgtcaccacttatgatgagttgaagaaagagttga

tcgacaatcttcacgtcgttgctacgcccgaatctgtttatcgccaactccgtaaccgtcgattgcggcc

ccaggaatccgccctgcattacttgtttgacatgcagcgcatcgcaggccaagccagtatcgccgattca

gaactgatcccgatcgtcatcgacggcttgggaagcccgtcaattacgtcgagtctgcatttcatgcctc

ttacgatggacgacttccggaagaaattgaaacttttcgaatcttgccgtcatctttgcaccacccagcc

cccttccgctgatgcccgggccacaacgaacagccgtatggaacggccccgcccatcgcaggaacccatc

cgctgcttcaactgctcccgattcggacaccttcagaacgcgtgcccgcggccgaagcgcccacccggcg

gatgttttcgttgtttccagactggacacgtctaccgtaactgccctgaacgtcgggccaacgccactgt

cgagggcaatactagttcggacgaagctctcgccacaaatcaagaggtgagtttgacatttttccaccct

tctgctaagcgtaccacccttccctgcgttcgttcccttctcgacacaggaagtcctgtgagcttcatta

gcgacacgatagtaccagttaagatgctaggacctctttccgctaccgaatactgcactatgattaaggg

accactttactctcgaggaaaaatcgattgtactatccgatttaagaatcattccgttcgacactctttt

attatattacctggaattgcgtggccagtcattatcggtcgcgatttactgaactcacttaatatttttc

ttacgtattcatctcttacaacttcatgtattactaaacctctatcgacggaacttaaagaagtagatac

gattcttccagaaaaattagacgatgctattaggagtatttgtgcgctcgatgtggctgaagccgataat

gaactggatttaggaaaaacactatctttggaacaacgttcaatagtcaattctattgttgaaaattcat

acctcaactatacttcagatgttataccgctcaaacaccctatgaaaatcaatctgactcatgatacacc

aatatttactaagccgcgaagactctcttatggtgaaagacagcaggttaagcaaattgttgataaactg

ttagcagaaaacatcatccggcccagtaattctccttatgcttctgcgcttgtcctcgttaggaaaaaga

gtggcgaggttcgtatgtgtgtggattaccggcccctcaacaaaattacagttcgggacaattaccccct

accccttatcgaaacttgtttggagcatctgtgtggaaaaaaattcttcagtttgctggatttgaaaagc

ggattccatcaagtcccaatgagtgaggagtctatcccctacacttcttttgtgaccccagatggtcaat

ttgaatatctgaaaatgccattcggtcttcgtaacgccccttccgaattccaacgttttattaattctat

cttaagggaattcattgatgatggcagaatagtagtgtacctcgatgacatcatcatcgcttctaccgat

cttagctctcacttcagtacccttcggtccgtattagaaaagattaagcagaataatttagaacttcgtc

ttgacaagtgcaaatttgtccatgaagaaatagaatacttgggctacaaagctaacttttctggaattca

gcctagtgataggcacattaaagcacttactaattaccctatgcccactaatttaaagcaactcagacgt

tgtcttggtctgttttcatacttccgacggtttgttccatctttctcttgcatcgctaaacctatgacaa

acttcttcagaaggacgaagtatttaacttcgattcaaattgcgtgcatgcttttgaaaccttacgtgac

aaacttgtgcattctcctatcctttccatattcgacccaaaacgggaaaccgaattacactgtgacgcaa

gttcctttggttttggcgctattctccttcagaaacaggatgacaataagttgcaccctgttgcttactt

ttccaaaaccacttcaaaagacgagtccaagttacacagttatgagcttgaaactctttccatcatttac

gctcttaagcgcttccacacttatgttcatgggctccccattagttactgactgcaactctctggtcgag

acccttaagaaccgtaatgcttccgctaagattgccaggtggtccttgtttctggaaaattacgactata

ccatctgtcatcgctcaggcacttctatgccccatgtcgacgcactgagtcgcaccgaagctgtgggtgc

catcggtgagattgaccttgacttccagcttcaagtagctcagacgcgtgacccatctatcgaagctctc

aaacatcggttagaatcagaagaagttgacggattcttacttcaagatgggcttgtctatcgcgacatac

ctgatggtcaacctcaattgtatgtcccttcggaaatggtcgacaacgtaattagacacactcacgagcg

aattggccacctgggcataaacaaaaccttcagcaaaatcagtcagcattactggttcccccacatgaag

cccactatcgacaaattcattaagaactgcctcaagtgcattgtttattctgcacctcatcatactaatg

cccggaatatgtacagcatccctaaagagcccttaccctttgataccatccatattgaccatttaggtcc

gctccctagttcttccttacgcaagaagtatatacttgttgttatcgatgctttcactaaattaaccaaa

ctttacccaacctcctcaactaatgcgaaggaagtgtgttctgccctttcccaatatatgtcttactata

gccgccctaggcggattgttagcgatcgagctacttgtttcacctcaaccttgtttgaggacttcttgga

atcgcataacattagccatgtcctcaacgccaccggatccccacaagccaatggacaggtagaacgggtg

aaccgtgtgttgcgtcctatccttagcaaactatctgatgctccagaccagaccgattgggtatccaagt

tgcggtcagccgaatacgctttaaacaataccgtccacacatctacgaacttctgcccctctgtcctact

ctttggtgtcgagcaacgcggtaaagttccagacgagttagccgaatacctggatgagaaatttgatcga

gcctctagggacttagaagccattcgggctaaagcgttagaaaacatagaagagtctcaacggaagaatg

aggaatactttagcaaaaagcacaaaccaccacagtgctataaggaaggtgacttagtggctatacgtta

ctctgatacgaccgatagcggtaataagaagctcaatcctaaattcaggggaccttacgtcatccataaa

gtgttgccccatgataggtacgtggtacgcgatgtagaaggatgtcaactcacacaactaccctacgatg

gggttctagaagcgaataagttgcgacgttggaccgagtccagtgattaggaaattgagggcaatttatt

gttcaggatagccgagc

>gypsy4-ltr_ag gypsy anopheles gambiae str. pest

tgtaacgtccggactaatatcgcccactgtcactcaacccgaaccccgaacgcagcggtataaacgcatt

ataccttgaccgctggagcacccggtgtgctagatgaactgtcatagaataaagctctcttcttggcgcg

acattgaactgaacagacgtaagccactgacttctgcgtataattatttgtgtgctcttccgaattgtgc

taaatcttattaaaacggccaattaaccttccgccaaccgtaaaacgcttggtcgttaca

>gypsy40-i_ag gypsy anopheles gambiae str. pest

ggcgcagccgttcgggcctttcctaagtgtgttttagtttaccgagtgataagtgcagaaacctgcgatt

tactttgtggtatcatccggtgtaaaccatctcccgacccgtggacgacttgcggtgatattcaggtaaa

ctaaaacaccctggaggatttcaatccggagaattggcaccgtacctacttccactgaaataagcattgt

gttaaaagcttatgcctagattccaaccctttccgaaagtgaaagcattatcttttgtgaaaaaaatgga

accagccgatctccatacgttcatggctaatttttctgccatggagcagcgtttaaatgctcttcaatta

aaaaatggcgaattattcagacagcttgagcaacaaaccgcgttggcttcccccggaccttcgggtggtt

atcaaaacgatcttttcaaagttcctgatcctttgaaaagcattccattattcgatggaaataaaatgca

tttggcatcatggattgccacagcgaaaaaaacactgagcatgtatcagccgattgtttctaccgcagtt

tacgctatgtacgagcaaatagttataaataaagtagaaggcaaagctagagatagtttatgtgtgaacg

gtaatcctacatcatttgatgaggttatcgaagtgttgaatgctacgttcggtgacaaaaaagatatggc

tacctatcaaacgatgctgtggtctatgaaaatggatagttcgatccatttttactacaagcgtacaaaa

gaaattgcacacgatatgaagtgtttggcaagaaaaaaagacttgtattgtaaccattggcaggctgtaa

atgattttatagaccaagaatgtcttgccgcttttataaaagggctaaataaagaatattttggctacgt

gcaagctgctaaacccgaagatttggaatctgcatattcgttcctttgcaaatttcaaaatctggaacac

acaaataaaatactagcacctcgtgccgaaacgaagatttcttatcagaaacagtttgacccttcatcaa

aaaatcggaatacgaacgcattcactaacaccattagaaagccgaatagttccgagaagctaaacagcag

taggcaaaagcacacaccgatggaagtcgatacgacaagaacaggtgtaaaactattcacgcacactagc

gaggatactgaacagcactatgataacggagtaaattttcaagaggtctttgcacctctgcctcagaggt

aaacgcagtaaacggaatgccttacatattgttacatatcgataataaatgcgcaaaaattttggtcgat

agtggatcgaataaaaatttcatttcccctaacgtatttcccaaatcgatggaaaaaaaatgcaaaccaa

tatcaatagcaaatagtaacggcacgcattacgcaaataaaaaggtcgatacaaaaatgttcaattcgga

tgttacattttttttgttcaaatttcataattattttgacggcattctagggtacgaaagcctttctgaa

ctccaagctgttttggatacgggtaaacataaacttatctttcccaatacaagcatagacctgagtatac

gacaactcaacccagaaacgcacacagtggaagccaattcagtaacacaaatcaaggtaccggtttcaca

cgaaagtggcaatgtgtttttacccgatgatgttcgcattaacgatatcgttattccgtcaggactttat

gttgcaaaaaatagcaatattacagcacttgcaagcaattttggaagaaaaatggttttcttttttacca

aaccagtagtaactttcaaagtcgataaaattgccgaagttaacaatacgatacaaacacctgatactat

ttcgaaaactgaaatagaaaatatgattaaaacggataatttaaaccaagaagaaaaagaacatctgtta

aaagtgctttatgaaaacatttccatcattcccaggaacaacgaaaaactatcctgcgttacatccgtta

aacacattattaacacggtagatgagatacctgtgcattgcaaatcatacagatacccacacatccacaa

agctgaaattcaaaagcagataaacgagatgcttgctgatggcattatacagcattcgatatctccgtgg

acttcaccaatttggatagtgccgaaaaagcccgattcaaatggtgcaaaacaatggcgaatcgtcgttg

attatcgcaaacttaacgaaaagacgattgacgataagtatccgatcccgaatattgaggaaatcttaga

taagctaggtcgcagcatgtacttcacaacactagacctaaaatctggttttcaccaaatcgaggtagaa

ctaaaagacagaccgaaaactgctttcagcaccgagaaaggtcatttcgaattcattcgtatgccttttg

gtttgaagaacgccccttctacgtttcagcgagcgatgaataacatactaaacgaattgatcggtacatg

ttgtttagtctatttggatgacattatcgttttcggaagctcactgcagcagcatatcgaaaatttacag

aaagttcttcacagattgaaacaggccaatttaaaaattaaaatagataaatgcgagtttttgcaaaagg

aatgcgaatttttgggacatattgtgacgcaggagggtatcaagcctaatccgaataaaattgagaaaat

tgtatcgtggcctcttcctaaaactgttacacaaattaaggcatttcttggaattttagggtattaccgt

aaatttattaaagattttgctaagttaaccaagccattaacaaaatgcctaaaaaaagattctaagatag

tacacgatgaaattttcataaactgctttagggattgtaaacaaatgttagttatggatcctattctcaa

atatcctgatttttctaaaaaattcattcttgaaacagacgcaagtgattttgcgttgggtgcagttctc

tcgcaacgattcgaagattcaaaagaacaccctatcgcgtatgcttccagaactctcaacgatacagagt

gtagatattctgccacagaaaaagaattgctcgccattatttacgctataaaacatttcaggccatacat

ttatggcaataagtttgaaatccgtacagaccataaacccttattatggcttaggcagaaaaacgattta

aataggaaattattacgctggaaattagagctggaagaattcgaattcgatataaaatacaaaaaaggaa

ctacaaatagtaacgctgactcactctcacgaatagaaccagcaataaatgctaatgtatctgaaagctt

aatgactcaacattcatctaacacagatgataatgattacattccaagtacagaaagaccgatcaacgaa

ttcagaaatcaaattattttagaacaaactaatgaaagtagtacagaaacgttaaaacaatttccaaaat

attctaaaataataattaagaaaccctcttttactacagataatttagttcagataattcaaaaatatgc

cgcacccaattgtttaaatggaatatattgtgacaagaacattttgaaaatgctgcacgaagtatacaaa

aaatattattctagggctaaatcattgaaatttcgctggactacaaaaatgcttgtagatgtaacagatg

aactagagcaagatcaaataattcaaacgtatcacgataccaaccatagaggagtaacagaaagtacaaa

acatcttcagagaaagtattttttcccccaaatgaaatccaagattactaaatacataaacctgtgcgcg

ttgtgtaaaaaatctaaatatgaaagacacccgtataagatcaaatacaaacaaacggatacacctaaaa

ggcctttagaaatcgttcataccgacatatttatcatgaaggataaacattacctcacattttgtgacaa

gttctcacggttagctctggcagtccctataaaaacacgatatactgtgcatatactgaaaggaatatcc

acatttatcgccacagtaggaaaacctttgctactaataatggaccaagaatgcagcttcaaatcaattg

cagttcaatcgtttttgaacgacaatttgataaaataccactacacctctgtagcgcaatcatcatctaa

tggcacggttgaaattgtgcatagaacaataagagaaatacacaacatattatctcagaaggaaagcaca

aaagacctatcggaatcaacaaaaattaatttagcagtagctacttataacgattctaatcattccgaaa

ccaacctcacgccgaatgaattgttccgtggttttagaaatgatcaaccgatatcatcgattctcgatga

gcacatacaggcgaaggagaaactttacgcagtagtccatcataaaatgctagagcaaaaagaaaagagg

attgctaagttgaacgaaaaaagagaagaacctataggcctttcagagggagaaaccgtgtttctgagga

aaaaaacaatttgaaacatcaagagcgttataaagaaatccaggtattagaagataaagacgttaacttt

atagattattgcggtagaaaatatcataaagaaaaactcaaaagaagatgtatgaactaaaatattttgt

aaaaataaccattagtaatacgcagcaatatagttagggttagataggtctccaatattatgttatagtc

ctaagatttgatttataacgttttaagaaaaaaagatgagaaagaaccacaagcgggacgaagatagcaa

acctacatgccgaactaattgcatacggtgaaactccaaagtgccgaggacgacacttttctaccccccc

gaga

>gypsy40-ltr_ag gypsy anopheles gambiae str. pest

agttatatgcgtggaacacaaatacaggtgcgacatcactgcaacagcctcgcacccatgccgatacgat

aggagccgattccgtcgcgtcagatcggtatcagcatccaagcgataagcacgcaataatcattttaaat

aaaagctgtggttccatactcataact

>gypsy41-i_ag gypsy anopheles gambiae str. pest

ggcgcagccgtccggagtgatttaagtgagtgtaattgtgtcaaaagtggaaaagtaagagactggaaaa

actctaactaaagtgatcccgcaagtgataaaagttatccaacgcatccggagcaacacaggaagacaac

atcgatcgaagctgtattccgccgaggagttgaggatcccccacgcagttacccacctggaaggaaaacc

agcatcgttaaatcacgaacccgcgacacgacagcaagaaaaaaaaatcgtaagtaattcttttattttc

ataaaaagtgcaacagtgtttcagtggaaatcagtgcttgaaaaaccagttttactcgcgtccttaggaa

atactaccagataggtttcttaggtagaaagtgacttactaccagataggagtagctttttctcatcgtg

ggagtactaccagatagggcccgtgatacaaagaaaagaaagagatcaacccagtgtcgtacacaacgaa

aaattcgtacatgaaagcaactgaaaggttcgattctcacagagatctttcaacctccgataatcttgaa

acagaaggagacaaaatggaagagatcgcaacacaattggtcgagatgatgcgagccatcacttctctcc

agaaccaatacacagcgctaagcgcaagtacctcatcaagtaatgcaggtaataatagggcatttgacga

ttattttcgcattcctgaccctataaaatcattgccaacttttgagggcaatcggaaacaactagcatca

tggttatcaaccgccgataacacactagctttgtttaaagacctagtacctgcagcagtgtaccaaatgt

acgttactgcagtaacaaacaaaatttgtgggaaagcaaaagacatcctatgtctttcaggaagtccaca

aaatttcgacgaaattaaagaaattttaatttcttcgctaggtgaccgacaagaattgtccacctataaa

tgccaaatgtggcaaaataaaatgaccgatgggatgagtattcacaaatattaccaccaaactaaagaaa

ttgtgcaaaatattaaaacccttgcaaaacaaaacgaacaataccgcacaaattgggttgcaatcaatgc

attcattgatgaagacgcacttgctgcattcatcgctggacttagaggaaattactttgggcacgcccaa

gccgctcgacctaaagacattgaggatgcgtatgcattcctttgtaagttcaaagcgacagaacaaaatg

caggcagccttaccaaaaatgttcaaaccccatccaataaaccaccttttaagaataaattcaaccaaaa

tgaaagtaccagttataataaaccaactaaagccatttcagaaaagaaattttcaatcaaaaactcagat

aaacctgaaccaatggatgtagatgcttcaatgcgcagtaaatacgcacaaaataaaaagcaattccaca

acaacgaggttgaaacggaacaagagtccaatgacagtgacagtgatgatgaaactgatcattttaacga

agtaaattttcgcctagcaggaagtctgaaaaacaatacttaaattccaaaaagaaatacaactatctcc

catatttacgcactaaacaaggtctcaatctattgatcgattccggcgcaaataagaatcttatccaacc

aggtgttttaaaaacaaaaaaggaaatcaaacagatcgaaatcactaacatagtgggaaaacaaattata

gatacctgcggaaaaacaaaccttttatacaaggaaattccctcacaaaaatattacgaattaaaattcc

acaattttttcgatggattgattggctcacaatttttagcagaaaatgaagctattctcaattaccgaaa

acaaacacttgaaatttcaaaagtaattatgccatttgaaaaatatttccctaacgagaaaaactataac

catgtagtaactctcccaaccaatacggacggagaatggattgtttacgaaccgactaaactttgtaaaa

aaattacagttcagccaggcgtatactcagccaaaaataaaaaaactacaattttactacagacaaatag

accaaaaccccctaatatacaacataaagcgttagaaattacagtaaataatttcgaaactgttactcct

ttacccatgaaacccgaatcaaaaattacaagcgaaatgctgagcgagataattcgtacatctcatcttt

caactttggaaaaagaccatctttttagaacaattattaaaaatcagaacgttctattaaaagcaggaga

aaaactttcagctacaccagatgttaaacacaaaataactaccactaatgatgcaccagtttttacaaaa

tcatatcgatatccacacgcatttaaaaatgacgtagaggaacagattaatgaactactacgaaatggta

tcataacccattcgacaagcccctattcctcgccaatatgggtagtccccaaaaaggttgatgcctcggg

aaagagaaaaataagagtagtaatcgactaccgcaagttgaatgaaaagaccattgacgaaaaattcccc

atcccacaaattgaagaaatactagacagtttgggtaaatcagtttattttacaacacttgacctcaaat

caggattccaccaaatcgagatggattctaacgataaagggaagacagcgttttctacagcacaaggcca

ctttgagttcaatcgaatgccttttgggttgaaaaacgccccagctgcttttcaacgcgctatgaacagt

gtgctaacaggactgataggaaatatttgtttcgtgtacctcgatgacattataatcatcggtaaaaact

tggaaaaccacatagaaaatctaaacacagttttagaaaggctttcaaaatttaacctcaaaattcaact

agataagtgcgaattccttagaaaagaaacagaatttttaggacatgttattactcaggaaggtataaaa

ccgaatccagataaaatcaccaaaatcctagaatggaaactgccatcaacacagaaagaaatcaaacaat

tcttaggtttatcaggctactatcgcagattcatcaaagactattcaaaattaacaaaacctctttcaaa

atgcctaaaaaaggatactaagataaacacgcaagatgaagagtataaaacatctttcaatagtctaaaa

caaattatcgcttcggatcagatattagcataccctgatttcgaaagaccgttcattctaacaaccgatg

caagcaactacgctcttggcgcagttttatcgcaaatccaagaaggaaaagagcgaccaattgcattcgg

aagcagaacattaaacgaagccgaatccagatactccactacagaaaaagaagccttagcgattatttgg

tctgtccaaaagtataaatcttatttgtatggtcataaattcacactcgttactgaccataaacctctta

cattcatcaaaacatccactaaaaactcaaaaattcttcgctggcgcctagaactcgagaatttcgattt

cgacatccaatacaaggaaggaaaggccaacgtagtagcagacgcgctaagcaggaaaacggaaattctt

acaaataccaatattaaccaagattcttccatttcaggcactcctaagaacaatgtttccaatacaattg

acgtcaacttcgaagaatcatcatctatttcagaatcccttcagcataataacccctcaccgaacaatac

taactctgattcacaaaccatgcattcagctgatacatccgacgattattttatccatttctccgagaga

ccaatcaattactacagaaatcagataattttccgaaaatcccatataacaactgacattacggaaaccc

cttttaacaactataaaagagcaattatttgcagaaatgattttgacgaattaacaatactagattctct

aaaaaatttccacaataacaaacaaaccgcgattatggcaccggatgaatcaactatttcactcattcaa

tccgtttatcgacaatacttcaaccaacatggacattttgttcttacacacttacaagtagaagacgtta

gtaacgaacaacgccaagacatcatcatagctaaagaacacgaacgcgcccatagaggtattcacgaagt

tcataatcaactaacaagatgctatttctttccacacatgatgacaaaaattaaaaaactaattaatctt

tgcaagatctgtaacgtacacaagtatgaacgcaaaccgtataacatcaaaatcacaccccgacctattg

aaacaacccctttcagtcgcgttcacatcgacatctttggaatcgacaaacacaactacttaacgttcgt

atgtgctttttcaaaatttttacaaaccatagaaattccgtccaggaacttgacagacataagaaaagct

cttgctcattttattacaacgtttggcgcaccgagaaaaattatttgcgatcacgaaaccacatttagaa

gtcttcaacttcaatcatttttagccaatttaggaacagaattagaattttcttcatcctccgaaaccaa

tggacaggtagagcgaacacacagtacaattatagaattgttcaacaccaataaacacaagttcagagat

ttaagctctccggagatcataaaagtagtgacagcactatacaacgaaacagttcactcctcaacaggat

tcacgcccaacgaaatcatttttaacagaactagcaatcggaatccagaacaaataattcaaaccactag

aaacatttacgaaaaagtatcccaaaagctccacaatgcaagtagaaacatgcagaaatataacgatgaa

aaagaaacaccaccggaaatagagacaggtaaacagatatttgtaaagaaaggcgttaggaaaaaattag

acccgcgattcaacgaaaaaacttgtcttaatgcgaatgataaaacagttacaatggcaagaaatatcaa

gaggaacaaaaacaagttaaggagaatcaagtcccagtaatacgttctttccgttttctttatctttccc

aggcttggagatcgaaaatgcaatactttccgataacattcatgctaatcctgcttacacaactctcgca

aagtaaagaattagaaatcatagatttgaacaggcaaccgatattttttcttaaaaccagaacttgtaga

ttacaaacaggaagcataaaattcatacatcctataaacatgttaacccttgaaaatgctattaacacca

ttacacacttttcgtacgaaaacattaataacgaacttaaagaaattgtcagactaaaagtaaaattatt

gtactcaaacttccaacaattaaaacctaaacatagaacagctagaagtctagaaatcttaggaacagct

tggaaatggataggaggaagtcctgacgccgacgacctcagaatcatcaacaccacaatgaacgagctaa

ccgagaacaacaacaaacagtaccgaatcaacaaacagttcgatcacaggctacgaaccctcactgacac

cataaatcaattgacaaaagaacgagaacaagtaatgctgaatgaactagaaaccattaaaaccataatg

aacatcgatatcatcaaccatgttttagaagaaattcaagaagcaatcagttggactaaagtatcagtag

ttagcaacaagattctatcatcaccagagataaactccatcaaaaccatactagaagaccaaggagtaaa

agtcgaattaccagatgaagcgctaaagctagtacaaccaattatcgcgatcaactcgaattcgatactc

tacatactgaagatccctcagctagctgatgaagaagcaacaatgcttgaagtatttcctctcagtatcg

ataacagaatcatagtagagactccgacgcaccttataaaaacacggaacaaagtgttcaaaccagccaa

acctgatgaatacatccaaaaccaatacagggagtacatcgataaatgcacatccaatctcatcctaggg

agaaaaagcgattgttctactgcaagaaagaacaacacgacgataaagctaatatccgatggacttatca

ttgtcgacaacgcaaaaggagcagccctgagttcaagctgtggacccgatgataaactcgtctccggaaa

tcttctgatacgcttcaacgattgcgaggtaacgataatgaatcaaacattttcttctaagactatctcc

agcacagtagagccatattggggagcagtatccatcaccgaagtcagatggcaacaccataaaccgatga

taaggcaagacgcattcgagaacattggaacgatgcagcattcttatctccagcagttcaacagcgcatg

gaattggagcctacttggaggagtattggtctcaacaatcttcacgttatccctggcgatcttcgttttc

acgttctacaaacgatcgatacggaccatcgcagacgttctaccgaaaatagcggacgcatgaggacacg

ctcttcttcacccccccgagg

>gypsy41-ltr_ag gypsy anopheles gambiae str. pest

agttacgtagaccgaatagctgagtccacgtaataagaagccaacacgtggacacgcgcaccgagcaacg

accgcatcaccggaacacgcgcgcatagcaacatgacgcgcatagcaacgggaaccggcacgtggacacg

cgcataccggatatgcagagtcagcagctatagggtagaatcgaaggctaggtgcattgtagaatttaag

aaataagttagttgtaatttggatcagctcagtgtaagaagcgcttgcgcttaagcaataaagttttttt

ttatgaaacgtgaagccttgcacttataatt

>gypsy42-i_ag gypsy anopheles gambiae str. pest

ggcgctgtagacaatccagagtgcaaaaatagtggcaaataacgaattaatcctcgttacgtaagtggtt

cccgcattcagcatcaacagctataggattacatccgctgttcgatttgagggccccccggcacacacct

ggtaccggaccatcggatttttttttatatccaaagcacgtgagtacgtttcatcttttcatagtgaatg

tgtattcggttagtttcaagcactaccagataggcttgaaataactacagtgtatttgtgtccatgtgag

tttgcgatcgattttttatttatcgtcgtaaactatctgtattgatactaccagataggtcaacagatta

tcgaagaagaatcgtcctcgtgttgtccgcgagtgaacttattgttagtagtgtgcgttgtgtgtattgt

gcgaatagcaagagcacgaggcatcggttgccgctgcttataacaattgtatcgacacgcatgcatgtga

acagttcagctaacgtaaagcctcgaatcgagcttagcgttttgttcaaaaatctatcggcttcagaaga

gagttcagattcggaattatccggagaatattcgaatatacctttacaagacagttcacttcctaattta

gaccaactcaatattaacactatagaaatggaaccaacagagcagcttaaaattatgaatcaaacgatag

cagatttgcaagaaaaaattgcagttttgacaatccagcaaagccaaccatctatcgatgtagccagttt

ttttcgcatccccgaccctataaaatcattgccatcatttgacgggaatcgcaaacaattatccacatgg

ctcaccactacagaagaaacacttaaccttttcaaagatagggttactggtgaggtttttaagatgtact

taacggcagtaataaataaaatcgaaggtaaagctagggatattctttgtttagccggaagtatcaacga

ttttgaatcattaaaagaaattctttttgatgcatttggggatcggcaggaattgtcgacttataaatgt

aaattgtggcaaaacaaaatggtcgacggaatgacaatacataagtactaccaaaaaacaaaagaaataa

ttcaatgcattaaaactattgctaagcagacccaagcttataaagataactgggctgtaattaatcaatt

tattgacgaagatggattagctgcttttatctcaggattgaaaggaatgtattttggccatatacaggca

gcccgccccaaagacattgaagaggcttatgcttttctttgtaaatttaaatcacacgaaataacagcag

actgcatggtccagaaaccacccaaccaacaaaaaaatagtttctttcaaaataatagaacagctaccac

tcaaaaaagccattttgctcaaaatcaaaacatcaatagggaaccttatccacaacctatggaagtggat

aattcaatgcgaagcagactcacattgaataaaagaacgattaataattttgaggttgcttcacaagatg

ataactctgcaaattgtgaacaaaattttcacttggattcgccatcaaccagcataacataaaatactgt

tctaattttttaccttacataaaagtgaaggacagcaaaaccaacaggcaaatccgtatgctcatagata

caggagcaaataaaaatattataagacctggtattatcaaaaacacaattaaaacagaacaagtgagtat

taaaaatatttttggtactaaaattattcaagaaaaagctatttgtaagcttttaggcccaaacatacca

gcacaaacttattacattatggaatttcacgatttttttgacggcataataggtactgaatttttgagtc

aaacaaacaccgtcatagatttcaaaaacaatgtcgtagttatcaacgaaacaaaaatttggttcgaaaa

attattttcctcaaaaaaattttaccaccacacaatatcaattgaaaccgatcaaaatggtgattggtgt

gttccaacttttgaaaatttatcagaaaatataattatagaacctgggttatattcttctatagataata

aaactttcgttaaagtattatctaccagtaaaacaactccccacatacctaaattgcatttcactgtaaa

taatttcgaaacattaacgcctattccatcggcttgtaatgacattccaacaaaaaaaataatcgaaaca

ttaataagaacagatcatctatcattttacgagaaatcgaaattgttcgaaactgtcatcaaaaaccata

acgtccttttgaaattaaatgaaaaactaactagtactacaataattaaacataaaataaacactaccga

tgacttgcctgtttatactaaaacataccgctacccacatgtctataagcaagatgttgaaacccaaatc

agggatatgctcgactctggcattattcaaccttcaacaagtccttattcatcgccaatttgggtggtgc

aaaagaaaatggatgcatctgggaaaaagaaagtacgggttgtcatagactacaggaaattaaatgacaa

aacgataaacgataagtttccaatgccagaaattgaagatattcttgatagcttaggtaaatcacaatac

ttcacgatattagatctgaaatctgggtttcaccagattgagatgcaccctgagcatcaagaaaaaactg

ctttctcgacaagccatggccattttgaatttactagaatgccatttgggctgaaaaatgcgccagctac

atttcaacgtgccatgaataacattttagctgagcttattggcaaaatctgttatgtttacttggacgat

atcgtaattgtaggaacaaatttagaagatcacttgaaaaacgtctcaacagtactcggaagattggcac

aatttaatttaaaaatacagctagacaaatgcgaattcctaaaaagggagaccgaatttcttggtcatat

aatatctcctgatggtataagaccaaaccccgaaaaagtgaaaaaaatattggactggcctataccttcc

aatgaaaaacaaatccgacaatttcttggattatcaggatattatcgtcgtttcatcaaagattattcaa

aaattactaagcatttaactaaatacttaaaaaaagatcagactattaatatcaatgacccggagtatat

cgattcattttctaaattaaaagaaacaatagcatcagaccaaatactagcatatccggatttcaattta

cctttcgttcttaccaccgacgcaagtgattttgctgttggtgcggtactttcgcaaatacaaaacaaag

ttgaaaggcccattgcctttgccagtaggacattaaataaggcagaaattaattactctactatagaaaa

ggaggcactagcaataatttgggcaatccgcaaatacaaagcttatctttatggaaatgagttcaaactt

ttcactgatcataaacctctaacgtttattaaaacctctatcaaaaataatagaatcttaaattggagac

tagaattagaaaactatcaatattctgtagaatacaaagagggaagagctaatgttgtagcagatgccct

tagcagaaaaactgaaaatactaacgaaattaattcaaccaatacaacaattttagctacaaatcattca

ggaagtacgtcagacgacttttatattaaatctagcgaaaggccattaaattactaccgcaatcaaatag

tttttgaattagttgctcaacatgaagatttaatagaaataccatttccaaattacaaaagaaccattat

acgtagaaccgactacgatgaatcaaagataacagacatattacgtaaattccataacgggaaacaaacg

gccattcttgcttcacaaaacctaatacaaataatacaaaactcttataaaaatcattttagctgcagcg

gttacatcgttatgacacactcccaagtcaaagatgtggcatccgttgaagagcaaaaccagctaataac

aagagaacacgagagagctcaccgaggtatacacgaaatcgaaaatcagatgaagaggtcatactttttc

ccaaagatgcatgatcgaatcaaatctgctatcaacgcatgccctgtgtgcaacatgcacaaatacgaac

gaaagccgtataacatcaagatctcaccgagggcagccaccgataaaccgatggagcgtggtcatatgga

tatattttccataaactcgaaaagcttcctttcattagcagattcgttctcaaaattcgcgcaaatgatt

cctatagatacaaaaaacctggtggacgtaaagaatgcgttagccaaatattttagtacttttgggattc

cattacaaataattaccgatcacgaaacaacgttcagatctatccaacttaaaaatttcttatgcaattt

aggttgctcgctgacatacgcatcatcatcagagagcaatggccaagtagaaaaaacgcactcaacaata

atagaaatttataatacaaacaaacataaattcgtggacatggacacagaagcgcttataccaatagcag

tttcattatacaatgcaactgtccactcagctactgggtatacgccgaacgaaatattgttcaatcaaac

aaatgaaatgaggcccataactatacacgaacaagcggaaaaaatatttgcgaatgcgaaaactaatatt

gaacgatctaggcaaaaccaaatgaaaggaaatattagaaaagaaactccacctttgattcgagaaggtc

aagaagtttatgtaaaacctaacataagaaaaaaattagaccctagagcaagaaatacaacagtaaacaa

tgtaacagatagaacttttgagaattcgagacacataaagcgacataaaaataagattcatcgtattagg

tcatagtaatacgtccggggacgtccgtcttttccccccgtgacg

>gypsy42-ltr_ag gypsy anopheles gambiae str. pest

aattatgtggtataaccattattaaccttggtactggtataacggtgatacgcgtggctgtagtggcagc

tttgtgacggtgacattcgatcgagagacgatctgataactctctttttccgttatctcccgagcagtac

agacgtctaggacgagataggttttttttcttatttttttatagttaggagttaggatttaggttatgtt

agaataagttagtttatttttttgtaaataaaaacttaatt

>gypsy43-i_ag gypsy anopheles gambiae str. pest

ggcgcagccggtagtcgttgattagaaaaaaaaaacagtgctagtgagccgtaatacgcttacgtagttt

gtccggatttgtagctactacaagatagggttacgtattcatctccgtgcaattttaaaagtgcggacga

tttgtggcaacgccgttttctgttcatcgacaggaccatcctcttctccctttttcatcaccaaacgtcg

gattgaggttaggatcctcacgtgttcttcaacaccaacacacaggtaagcaaacttttggtttttaatt

tctcttgtttgtctgagtgacagagatagaaaaacttttgctactgtgtttcggatagtcactataaaat

agaactattcgatttcttaaatttgaaaaattaaaaacgtgttcttagagagacgtttataaccgaagca

ttttatgtggagaaattcaaccattgaggctttctgttcggatacagattcaatatcgaacgattccggc

agcctctcatacgtgcccaccaacgttgatattcctctcgagcgcttatctttaaccgataacatggaac

aaatacaggcgcaacttgagcagcttacgcatctcgtacagactctcgctgtctctcacgagcaacagtc

tcaaaaaatactcacacttgagagtaatactcagcaggctagtagtagtgtagcgagtagcgtagcgcag

ggcgctaaccttagcgttaatttggatgccttttataaaatccctgacccgataaaggcagtccctgtgt

acgatggaaacaaaaaacagctttttgcatggcttaaaacagcagagaacgcactgaacatttttaaggg

aaacgtgcattatgccgtttaccaaatgtatgaggatgctataagcaacaaaatacaggggcgtgcaaaa

gatgcattgtgcttagaaggtaaccctactgattttcaagatatcaaagggattttgacaaaaactttca

gcgatagatatgatctatcaacgcacatgtgccaattatggcataataaaatgaacaatagtaccaattt

aaaaggctattacatgcagacaaaagaattaatacaaaaaattaagcaaatagcaaggcaaaacgaaaaa

tataatgagagctggtcagctataaaccacttcattgacgaaacaagcctagctgcatttattgcagggt

tacatgaaccatattttggttatgtacaagcagcacgacctgaggatttggaaggggcatatgcgttttt

atgcaagctgaaatccaacgagtccacagcacaaaattcaaatgttagaaaagtgaccgaaacaaagcat

tacacttcacacaatgaaggtttgcagaagcgatttgattgcaacgcaaatagaccaaggatgacaaatc

aagcttcgacatcgagtttcatgaaccgtaacacgagaaacgtgcactcttctaatagggattctccagt

tccaatggaaactgagtctagtagaacaagattaaccttgaatcaaaaaaatttgaatacaaccgaaaca

ttcaattcaaattgtccacgaaattttgaaaatgaggaggatgatattttggtaaatttttgggaagctg

tagaaacaactttaccgatttagattttcttccatacataacagggacacatcctaaaacttctcaacgg

tttaaattacttttggatagcggtgcaaataagaatattttgaaaccgggaataattcagtcattacaac

ctgttaataccgtaataaaaaatgcatcaggttgccataatgtttcacaaaaagggtcaataaatttgct

tggaccagaattgccaaatcaaatgtactatgaatatgatttccataaattttttgatggaattattgga

tcgcaatatttagctagatgcaattctataattaactatagcaacgaaacagttactatcgctggtaaaa

ttattccatttgttaaatattttccaaccaacaaatttttccatcacatagtttcaatcgacactttaga

aaatggtgattggtttgttccatgtcatcaattactctgtaataatcttataatagaacctgggctctac

aaatcagaaaataataagtccatcgtaaatattataagccaatcccatattgcaccaacaattacaacca

gttttcatctaaatgtaaataattttgaaacaattaaccccattccattaaaggtcgatgagccactcaa

taaaaaagcagtagaaattttgataaggtcggaccatttgacaccttatgaaaaagataatctttttgat

gtaatattgaataaccaacaagtactccttaagagaaatgaaaaactttcttcaacaaccgtagttaaac

ataaaattataactaaagacgatgagccaacgtacagcaaaacttatagatttccaaatcattttaaaaa

agacgtagaagagcaaatcttagaaatgctcgaagatggagtaattattccctcgaatagtccatattcg

tctcccatttgggtagttcccaaaaaaccggatgcttcacaaaaaaggaaaataagagtcgtaatagatt

ttcgcaaactgaatgaaaaaactattaatgataaataccctattccccagatagaggaaatcttggacag

tttgggaaaatctacatacttctcgacattagatctgaaatctggcttccaccagatagaaatggaccct

gcacatcgcgaaaaaacggccttctcgacatctcagggacacttcgagttcacgaggatgccgttcgggt

taaaaaatgccccagcgacgtttcaacgtgcaatgaaccatgtactaagaggatacataggatcgatttg

ttttgtctacttagacgatattattataattggtaataatttaacatcacatttagaaaacctggataaa

gtgttgaaaagattagcagcttgtaatttaaaaattcaagtaaacaaatgtgaatttctgaaaagagaaa

cagagttcctaggtcatttaataactcaagatggaataagacctaatcccgataaaattaaaaaaatcct

cgattggagtctacctatgaatcaaaaggaaatcaaacaattcctagggcttactggttattaccgtaag

tttgtgaaagactatgctagattaacaagacccctttcaaaatgtttaaagcaaggtgctaaagtagcgt

atgacgaggaagattataaaaaggcatttgaagagctaaaacatattattgcttctgatcaagtccttgc

ttatccggactttgaactgcccttcattctcactacagacgcaagcaactttgcattgggagcggttttg

tctcaagttcaagataattgcgaacggcctatagcattttctagtagaacactgaatagtacagaatcca

actatccagcaacagagaaagaggccctagctataatttgggcggttaaaaagtttaagccctaccttta

cgggaaaaagtttacgttaatcacggaccataaacctcttacgtttataaaaacatctttcaaaaattcg

aaaatactgaattggcgtttagagctagaaaacttcgactatgaagttaaatacaaagaaggaaaaacta

atgtggtagcagatgcgttgagcaggaggactgaccctgacaaatctatacgagatattaatgcttcctc

catttcagactcgccaagaagtggaacaaaccgaagtagcgaaacgattcattctggggatatatccggc

gattactttgttcactacacaacaagacctgtaaatttgtatcgcaatcagcttatattcaaattaggag

acacaaatgcgattgttaaagaaacaccatttgtaaattttcaaagaactgttatcactcagaacaattt

cgattcaagtaatgtcagttggtttttaagcatgtaccacaacggaaagcagaccgctatttttgctgca

gaaaatcttatgcaaacgattcaggaaacatttagtagccataatttcacaaaaggtcattttgtagtta

caccaaatatggtagaagatgtaacaagcattgagagacaaaattttctcgttacatcggagcatgacag

agcccatagaggtatatgtgaagtagaatatcagttacggcgatcatatttctttccctgcatgctaaaa

atgattagaagttatgttaacagctgtgaaatttgtagtagtcataagtacgaacgcaagccttacaata

taaaaatttcacccagaccgattacccataaaccattagatcgcgttcatatggatatttatatcattaa

taagtgtagtttcttatcaataattgattcatttacaaaacatctacaaatgatgtaccttaaaaacaag

aacatagtacaagttcaaaagaagttagctacttatttttcaatcataggattgcctaaagaaatcataa

cagaccatgagaccacgtttatgtctgtccaactaaaaagttttttgtcgtcgttaggagtcttgttaca

atatgcatcttgttcagaatcgaatgggcaaattgaaaaaacacactgcacaataacagaaatcattaat

acaaataagtataagtatgaaggagcagatacaagatctttaacaaagattgctgttaccctttacaaca

atagcgtgcatacggcaacgaaatttacacccaacgaattgttgtttaacaattctaatagtgtgaatcc

accagaaatatctggaaaagcacaaatattatttgctacggcatataagaatatgacaaaagctgccaag

cgccaaaccaataataatgattcaaaatgtgctcctccaacattggaggaaagggaagcagtgttcatca

tgcctaatataagaacaaagatgcaaccaagagcaacaaaactcattgtaagaaacgtactggacaaaac

atttgaaaatgctagaggagttaaaagacataagcaaaaaatcaagaggttgaagaagtacaattagcga

attcttttgaacaaaatttgaatattgataaacataccatacttttctagtgaacgtaatttaaaggtac

ttaatcacttgaaactaataaaaagcgaatgaaattctgttgtaaggctgcgtaaggttaggtaatacta

agttgactttattataacgtttttatactgtcctattatctttgtactgcctatcctaatcggacgagga

cgcccgagctttaccccccggaga

>gypsy43-ltr_ag gypsy anopheles gambiae str. pest

agtaatagatagtgtgtttattattatatgtttacatataaaacgttcaatcacacctcaccatcatata

aaaaccttattcaactgacgtgttgcgctgtcagtttgatgataaacgcaggctcctcaataaagtcatt

attattccgatcgttaaagagaaaggacacaaacacaacacccacgacttgtaatt

>gypsy44-i_ag gypsy anopheles gambiae str. pest

ggcgcccgaatcaggagagccctagacgggaagagataagctcagagcagaaaaaaaaatcttcacaaat

ctcagagagcaatactctctgaaacagtggtccaaagagccactactgcaaaagagcgaagcgaagcttg

ggacactgtggttatcacccgatcgctgtacgagacccctagtaaccggaccggtaaaaaggattcccct

ccaccagctgcctaaccttcccccgaacggagtatcaccaacgagttacaacggagcatcaccgacggag

cacgacgaaaatcagcgttggtaagcaataatagtgtctgtgtgagtattattttgtaagtattcatagt

gtgtggtgcaaagtgacagtgtcgaagtgaaaaaatgttcaacggcagccagagttttgttcaaccggct

ccaaatattctggagctcggtaaggtgcccgattttgtgagagatcttcgcaatttcgatggacaaccaa

acgagttgaataactggatagatgatgtagaaagcatcatgaatctttgcgaacggtgtcgagtaggaga

aacttctttaatgtatgagttgatccaaaaaacaatccgacgtaaaatttgcggccaagctgccgacgtc

ttaaattctaacaatattacttctaaatggtcagagattaaagaaacgttgttattatattattgcgata

aacgagacttaaaaacattagattttgagctgacaaccgttaaaagaggaaaaagtgaaccattaaattc

atactatggcagggttaacgaactattagcatctttggtaacacaagtgcagacacaagatagttataaa
[truncated: 1,952,444 more chars]
