# Supplementary material for: Global Analysis of Small Non-Coding RNA Populations across Tissues in the Malaria Vector, Anopheles gambiae
Source: Insects. 2020 Jun 30;11(7):406. doi: 10.3390/insects11070406 (PMC7411766; doi:10.3390/insects11070406)
Supplement: Supplementary file 1 [file insects-11-00406-s001.zip › insects-827855-suppl/insects-827855 - supplementary.docx]

**Supplemental Data**

**Figure S1. Fragments of tRNAs and rRNAs across mosquito tissues.** Read annotation percentages and corresponding histograms for tRNA- and rRNA- fragments across mosquito tissues. Data graphed as average +/− SEM from three biological replicates. Mosquito tissue groups include the fat body abdominal wall (FB-Ab), midgut (MG), ovary (OV), and remaining head and thorax tissues (R).

**Figure S2. Differential abundance of rRNA- and tRNA- fragments across mosquito tissues.** Heatmap of (A) rRNA- (B) tRNA- fragments across mosquito tissue groups. Data graphed as average log_10_ transformed RPM values with color scheme denoting minimum (blue) and maximum (red). Mosquito tissue groups include the fat body abdominal wall (FB-Ab), midgut (MG), ovary (OV), and remaining head and thorax tissues (R).

**Figure S3. Mapping properties of *AGAP006442*-derived small ncRNAs from midgut small RNA libraries.** Figure illustrates the mapping properties of sense and antisense *AGAP006442*-derived small ncRNAs in midgut tissue (MG) from three biological replicates (br1-3). Biological replicate 1 (br1) is shown in Figure 5E of the main manuscript.

**Figure S4. Mapping properties of *AGAP003387*-derived small ncRNAs across tissues.** Figure illustrates the mapping properties of *AGAP003387*-derived sense small ncRNAs in all mosquito tissues, each with three biological replicates (br1-3). Mosquito tissue groups include the fat body abdominal wall (FB-Ab), midgut (MG), ovary (OV), and remaining head and thorax tissues (R). Biological replicate 1 (br1) across tissues is shown in Figure 5C of the main manuscript.

**Table S1. Bioinformatic data for read abundance per small ncRNA group across tissues.**

**Table S2. miRNA annotation update.**

**Dataset S1. Conversion of raw reads to RPM across small RNA tissue libraries for rRNA- and tRNA- fragments.** Data is split between two tabs, (1) **rRNA** and (2) **tRNA**, each tab contains raw reads to RPM conversion and corresponding log_10_ values. Only values with log_10_ >1 were used for abundance analysis across mosquito tissues.

**Dataset S2. Conversion of raw reads to RPM across tissue libraries for *miR-956.*** Reads mapped to *miR-956* loci on chromosome 3L interrogation were converted to RPM across mosquito tissues. Mosquito tissue groups include the fat body abdominal wall (FB-Ab), midgut (MG), ovary (OV), and remaining head and thorax tissues (R), each with three biological replicates.

**Dataset S3. Transposable Elements retrieved from databases.** Consolidated list of LTR, non-LTR, and DNA transposons from Repbase and TEfam databases. Sequences are grouped according to TE class and database.

**Dataset S4. Reads for piRNAs across Repbase and TEFam annotated transposable elements.** Number of mapping reads across LTR, non-LTR, and DNA transposons in three biological replicate ovary (OV) small RNA libraries. Read data with average and median values are grouped according to TE class and database.

**Dataset S5. Reads for mRNA-derived small ncRNAs across tissues.** Data include (i) read abundance mapping across mRNA transcripts, (ii) read abundance for one specific sequence, (iii) percentage of specific sequence over total mRNA-mapped reads, (iv) top mapping sequence, and (v) read sequence mapping orientations. Mosquito tissue groups include the fat body abdominal wall (FB-Ab), midgut (MG), ovary (OV), and remaining head and thorax tissues (R), each with three biological replicates.
