# Supplementary figures and images for: Global Analysis of Small Non-Coding RNA Populations across Tissues in the Malaria Vector, Anopheles gambiae
Source: Insects. 2020 Jun 30;11(7):406. doi: 10.3390/insects11070406 (PMC7411766; doi:10.3390/insects11070406)

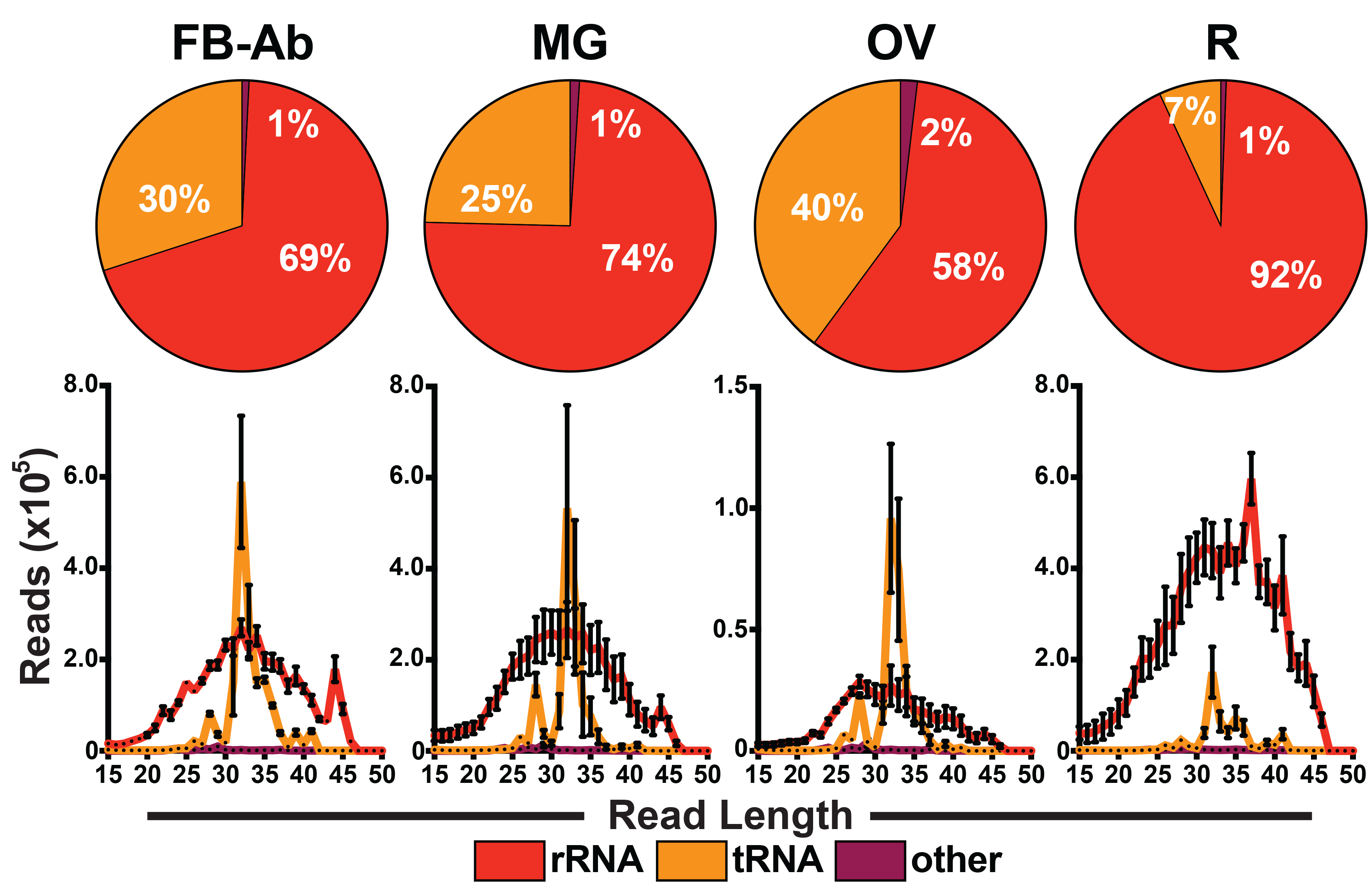

Supplement: Supplementary file 1 [file insects-11-00406-s001.zip › insects-827855-suppl/Figure S1.tif]

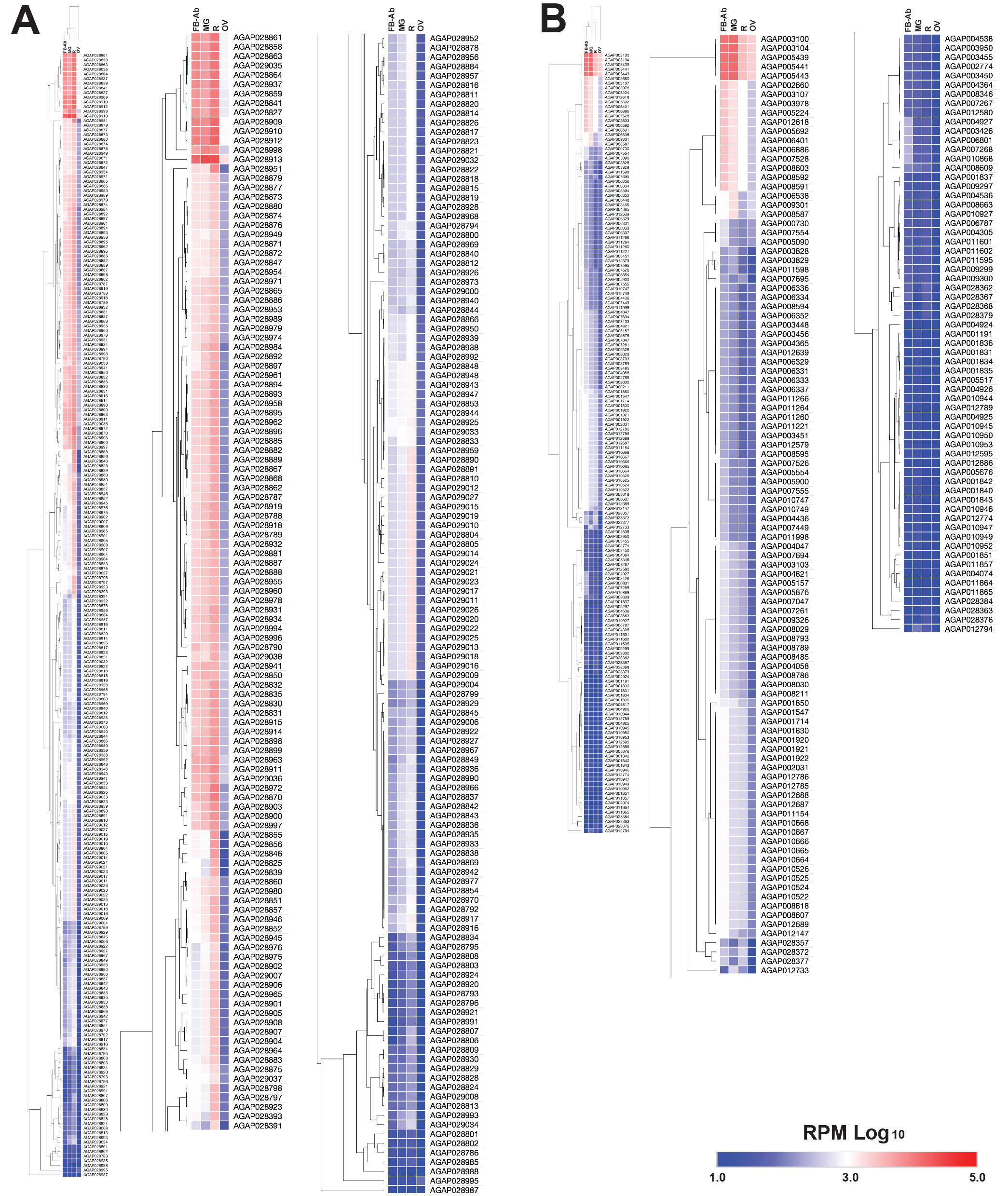

Supplement: Supplementary file 1 [file insects-11-00406-s001.zip › insects-827855-suppl/Figure S2.tif]

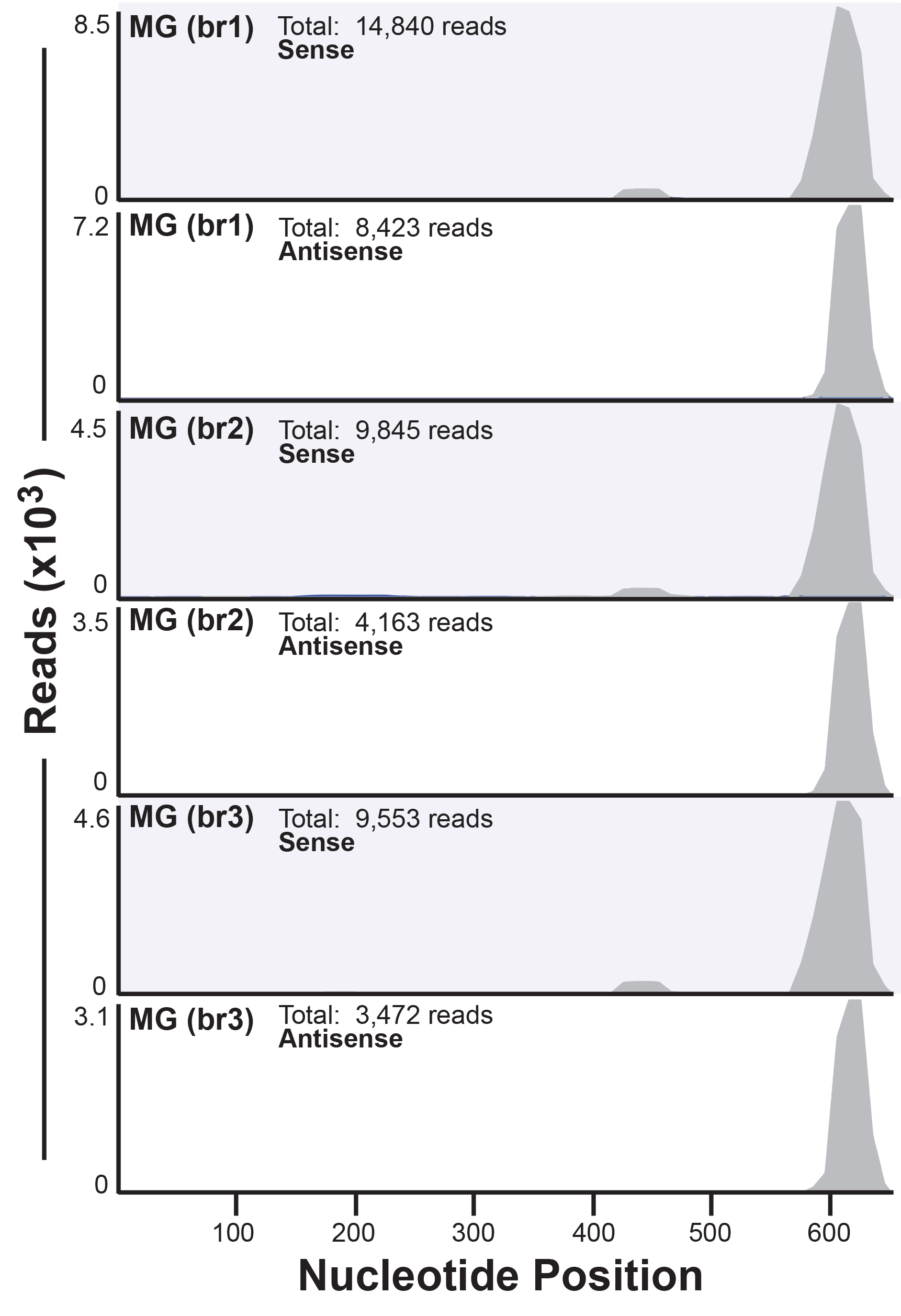

Supplement: Supplementary file 1 [file insects-11-00406-s001.zip › insects-827855-suppl/Figure S3.tif]

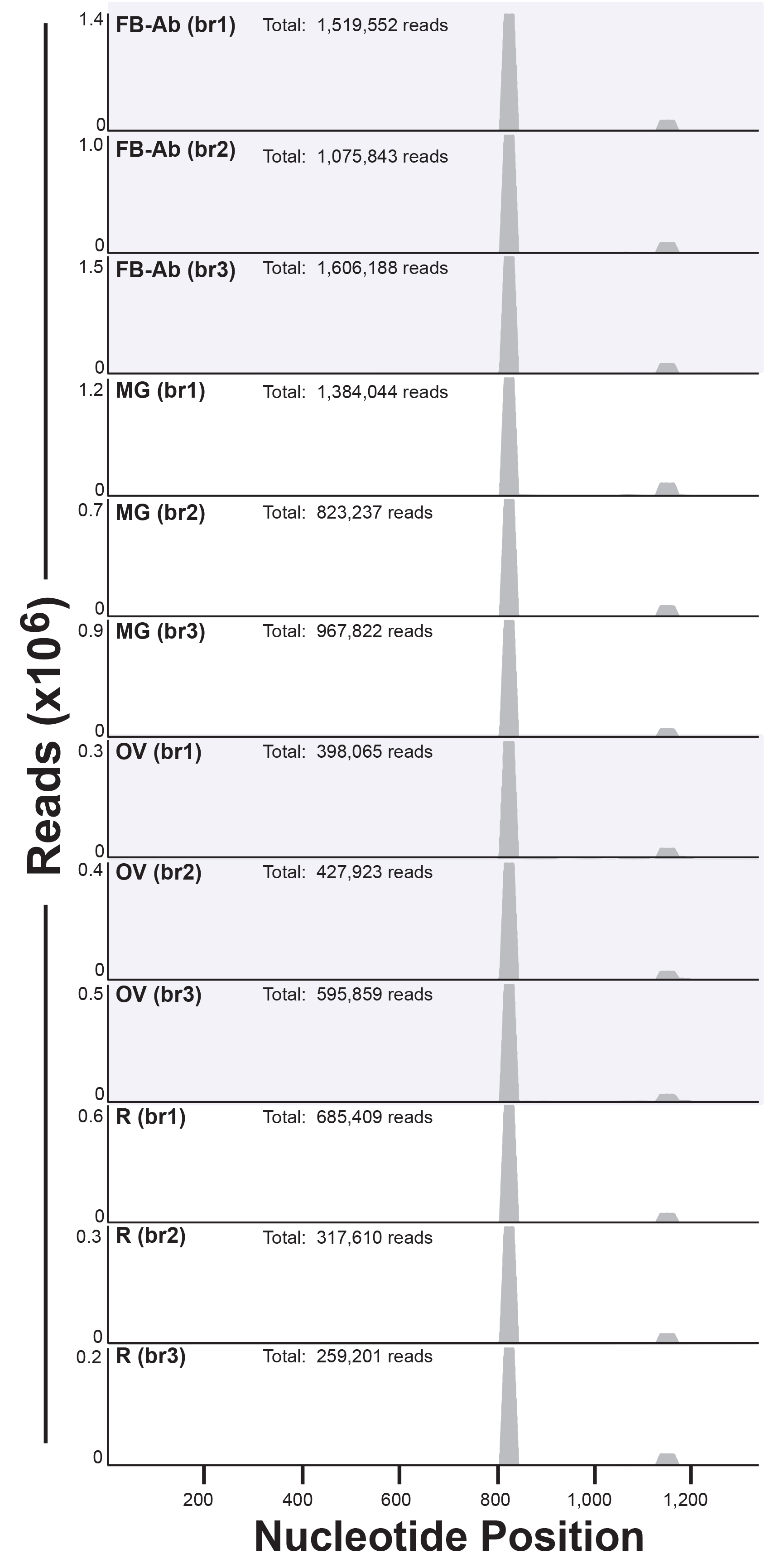

Supplement: Supplementary file 1 [file insects-11-00406-s001.zip › insects-827855-suppl/Figure S4.tif]
